# Digital twin-driven fault diagnosis of power substations by multi-modal fusion learning

**DOI:** 10.1038/s41467-026-73483-5

**Published:** 2026-05-20

**Authors:** Yulun Wu, Ying Chen, Tannan Xiao, Lifu Ding

**Affiliations:** 1 Department of Electrical Engineering, Tsinghua University, Beijing, China; 2 State Key Laboratory of Power System Operation and Control, Tsinghua University, Beijing, China

**Keywords:** Energy grids and networks, Electrical and electronic engineering, Computer science

## Abstract

Substations are critical infrastructures for ensuring reliable power system operation. With increasing digitalization and system complexity, the rapid growth of multi-source data poses significant challenges for accurate and timely fault diagnosis. Existing approaches often struggle to effectively integrate heterogeneous data or adapt to varying operating conditions. To address these limitations, this study proposes a digital twin-driven fault diagnosis framework incorporating a multi-modal fusion model that integrates system topology, alarms, fault waveforms, and SCADA data through Graph Attention Networks and self-attention mechanisms. In this work, the method is validated on a 110 kV substation using 11,597 training and 3,890 testing scenarios generated in CloudPSS. Experimental results demonstrate over 95% accuracy in fault location, 97% in fault type identification, and 90% accuracy in protection failure detection under 30% data loss conditions. The deployed digital twin system further verifies the practical feasibility of the proposed approach, highlighting its robustness in complex operating environments.

## Abstract (web summary)

The study builds a digital twin of a power substation and combines topology, alarms, waveforms, and measurements using attention-based graph models to diagnose fault location, fault type, and protection failures with robust performance under noise and missing data.

## Introduction

Substations are crucial nodes in power systems, and their operational safety directly determines power supply reliability. Rapid and accurate fault diagnosis is therefore essential for maintaining secure and stable operation of power systems. As substations evolve toward digitalization and intelligence, the increasing number of secondary devices and growing system complexity introduce new challenges for fault diagnosis^1^. During fault events, substations generate massive heterogeneous data—alarms, fault waveform recordings, and Supervisory Control and Data Acquisition (SCADA) data—that reflect system conditions from multiple perspectives. Effectively utilizing these multi-modal data to enhance diagnostic accuracy and robustness has become a key research focus.

Traditional fault diagnosis approaches in power systems are generally categorized into rule-based and data-driven methods. Rule-based methods rely on predefined expert knowledge and logical rules, such as expert systems^2,3^, Bayesian networks^4,5^, fuzzy sets^6,7^, rough sets^8,9^, analytical models^10,11^, and Petri nets^12,13^. They primarily use the status data of protective relays (PRs) and circuit breakers (CBs). Although multi-source fusion strategies combining electrical and status data have been explored^14–16^, these methods remain limited by PR and CB malfunctions, false alarms, and missing data. Moreover, they depend heavily on prior knowledge, making model construction and updates costly, while their generalization to unseen or complex faults remains poor.

Data-driven methods employ machine learning or deep learning for automatic fault detection and classification using real-time data. Early works applied wavelet transforms combined with deep neural networks (DNNs) to detect faults in microgrids^17^, and Graph Convolutional Networks (GCNs) were introduced to enhance fault diagnosis in distribution networks^18^. In addition, recent advancements have further integrated cutting-edge architectures, such as transformers^19^, Recurrent Graph Neural Networks (R-GNNs)^20^, and Message Passing Neural Networks (MPNNs)^21^, to enhance fault detection performance. However, despite the promising results, these methods generally fail to leverage multi-modal fault data, which could provide a more comprehensive understanding of the fault conditions. Additionally, they often lack robustness and adaptability in handling different topologies, which restricts their performance across diverse operational scenarios. Furthermore, data-driven methods face challenges such as insufficient training samples due to the rarity of fault events, particularly for complex fault patterns, impacting their effectiveness and generalizability.

For substation fault diagnosis, traditional rule-based approaches are widely applied, and many studies have attempted to enhance them with more flexible reasoning mechanisms. For example, researchers have introduced a Bayesian network–expert system hybrid to enhance coordinated analysis of primary and secondary systems^22^, a Petri net–based method to support integrated fault identification and protection verification^23^, and a rough set–based bio-inspired model that combines rough set reduction with spiking neural P systems to better handle uncertainty in alarm information^24^. With increasing data availability, research has gradually shifted toward data-driven methods. Recent work includes deep reinforcement learning models that learn fault patterns from historical data^25^, as well as ANN- and RNN-based approaches that leverage communication, protection, and secondary system data for fast diagnosis and improved fault localization^26,27^. These data-driven studies, however, primarily focus on the secondary systems of smart substations. Beyond these developments, multi-source information fusion has also been investigated. One representative study combines time–frequency features with Dempster–Shafer evidence theory and BP neural networks for busbar fault diagnosis^28^. However, this method focuses solely on busbar faults and does not incorporate alarm information, thereby limiting its diagnostic scope.

Digital twin (DT) technology provides a promising pathway to address several inherent limitations of existing fault diagnosis approaches. As a high-fidelity virtual representation of the physical substation that integrates real-time operational data with computational models, a DT enables intelligent monitoring, simulation, and optimization of system behaviors^29,30^. In current substation practice, DTs have already been applied to enhance real-time fault monitoring, fault localization, and predictive maintenance across both primary and secondary systems^31–33^. By simulating diverse and realistic fault scenarios, DTs can generate abundant, balanced, and topology-aware training samples to mitigate the data scarcity and class imbalance commonly encountered in data-driven methods^34^. Moreover, the ability of DTs to provide consistent modeling of topology, device interactions, and protection logic greatly facilitates the alignment and fusion of heterogeneous data. Additionally, DTs support flexible model deployment and online updating, and their efficient data interaction within a coherent cyber–physical environment enables timely access to multi-source information, thereby enhancing the practicality, adaptability, and scalability of intelligent fault diagnosis solutions.

Multi-modal fusion aims to integrate heterogeneous data, exploit their complementarity and correlations, and thereby enhance the performance and generalization of downstream tasks. A recent work^35^ categorized fusion models into encoder–decoder-based^36^, attention-based^37^, GNN-based^38^, generative neural network-based^39^, and constraint-based^40^ frameworks. These models have shown strong potential in industrial fault diagnosis—for example, deep coupling autoencoders for multi-sensor data fusion in rotating machinery^41^, time–frequency transformers for bearing fault diagnosis^42^, and graph-based fusion models for root cause localization in large-scale microservice systems^43^. Motivated by these advances, this study aims to design a substation fault diagnosis framework that leverages diverse data modalities and combines the strengths of attention and graph-based fusion for accurate, robust, and topology-generalizable performance.

In this work, we develop a digital twin–driven multimodal fault diagnosis framework for substations that integrates heterogeneous data sources, including system topology, alarms, fault waveform recordings, and SCADA data. The proposed method employs Graph Attention Networks (GATs) combined with self-attention to effectively fuse multimodal data and capture spatial–temporal dependencies across primary and secondary systems. To highlight the conceptual advantages of the proposed approach, we provide a comparison with representative existing methods (Supplementary Table 1). The developed approach is further implemented and verified on a real 110 kV substation system. A total of 11,597 training and 3890 testing fault scenarios are generated on a digital twin platform—CloudPSS. The proposed framework demonstrates strong performance, achieving 95% accuracy in fault location, 97% in fault type identification, and 90% in protection failure detection under 30% data loss. Moreover, the deployed digital twin system achieves an end-to-end latency of approximately 1 min for IEC 61850-based data communication and 0.5 s inference time on an NVIDIA RTX 3090 GPU, confirming its high diagnostic efficiency and potential for real-world online deployment.

## Results

### Case setup

**Fig. 1:**
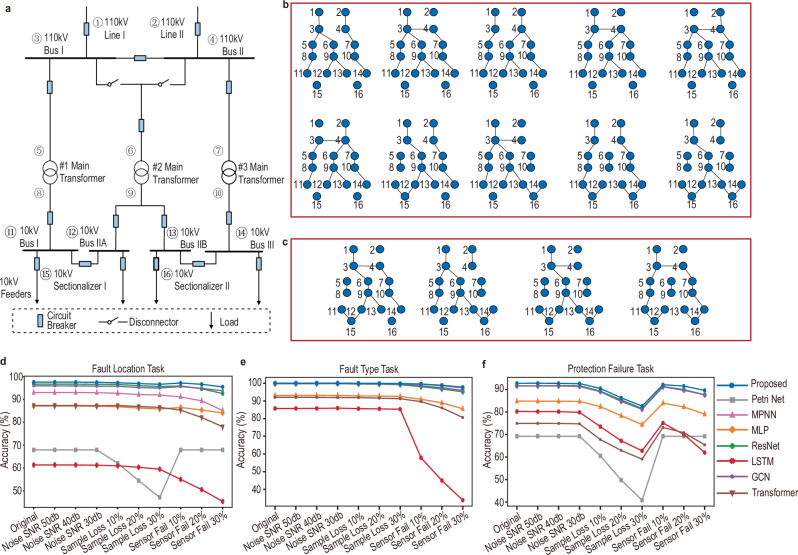
Overview of the substation system, topology configurations, and diagnostic model performance under diverse test conditions. **a** Overall layout of the studied substation. The system comprises two 110 kV incoming lines, two 110 kV busbars, three main transformers with both high- and low-voltage terminals, four 10 kV busbars, and two 10 kV sectionalizer switches, defining 16 physically meaningful fault locations within the primary network. **b** The ten topology configurations used for model training. Each graph contains 16 annotated nodes representing the same set of primary-system fault locations—Nodes 1–2: 110 kV Lines I and II; Nodes 3–4: 110 kV Buses I and II; Nodes 5–7: high-voltage terminals of Main Transformers #1–#3; Nodes 8–10: low-voltage terminals of Main Transformers #1–#3; Nodes 11–14: 10 kV Buses I, IIA, IIB, and III; and Nodes 15–16: 10 kV Sectionalizers I and II. **c** The four topology configurations reserved for testing. Designed to introduce previously unseen structural variations and thereby assess the generalization ability of the diagnostic models. **d** Model performance on the fault-location task under a range of test conditions. Including the original dataset, noise-corrupted datasets (SNR = 50, 40, and 30 dB), sampling-loss conditions (10%, 20%, and 30%), and sensor-failure conditions (10%, 20%, and 30%). Line plots report the mean accuracy across 5 different random seeds. **e** Performance of the same set of models on the fault-type classification task across the corresponding test conditions. **f** Performance on the protection failure detection task, evaluated across all test datasets. In all diagnostic tasks, the curves highlight differences in model robustness as data quality degrades. Source data are provided as a Source Data file.

The case study and engineering validation are based on a real 110 kV smart substation located in Shenzhen, China (Fig. 1a), which operates in full compliance with the IEC 61850 standard (Supplementary Notes). The proposed method, however, is equally applicable to substations at other voltage levels (e.g., 220 kV, 500 kV) that adopt the IEC 61850 communication standards.

The substation includes two 110 kV transmission lines, two 110 kV busbars, three main transformers (high- and low-voltage sides), four 10 kV busbars, and two 10 kV sectionalizer switches, providing a total of 16 potential fault locations. The protection configuration includes various protection schemes and corresponding operation time delays for transmission lines, busbars, and transformers (Supplementary Table 2). A joint primary–secondary digital twin model is constructed on the CloudPSS platform^44^ (Supplementary Software), simultaneously simulating electromagnetic transients and data acquisition from both the primary and secondary systems.

To capture diverse operational conditions, ten topologies are simulated by varying circuit breaker open/close statuses (Fig.  1b), generating the training dataset through batch fault simulations. Each topology covers all 16 fault locations and 10 fault types (A/B/C single-phase grounding, AB/BC/CA two-phase short-circuit, AB/BC/CA two-phase-to-ground short-circuit, and three-phase short-circuit). Fault resistance is randomly set within 0–10 Ω to represent low-resistance faults. Based on these combinations, 1600 base fault scenarios are defined. To further consider secondary system uncertainties, multiple protection failure conditions are generated automatically using a fault enumeration tree traversal method based on Breadth-First Search (Supplementary Methods, Supplementary Fig. 2), resulting in 11,597 distinct fault scenarios for the training dataset.

For each fault case, alarm data are collected from PRs, including time-tagged event signals recorded during the fault process. SCADA measurements record the root-mean-square (RMS) values of three-phase and zero-sequence currents of each line via current transformers (CTs), sampled at 2 Hz, retaining 1 pre-fault and 19 post-fault data points within a 10 s time window. Fault waveform recordings capture the instantaneous values of the same current quantities at a sampling rate of 4 kHz, which is consistent with the requirements for high-speed fault recording in smart substations. Each waveform record contains 5 pre-fault cycles (0.1 s) and 20 post-fault cycles (0.4 s), yielding 2000 data points per channel. It is worth noting that the proposed method does not impose strict requirements on the sampling frequency of waveform recordings, as the proposed model extracts RMS features over multi-cycle windows. As long as the sampling rate is sufficient to capture the transient characteristics during fault events, the diagnostic performance of the model remains unaffected.

To enhance robustness and generalization, multiple data augmentation strategies are applied, including adding zero-mean Gaussian noise with signal-to-noise ratios (SNR) of 50, 40, and 30 dB; introducing random sampling loss by removing 10%, 20%, and 30% of time-series data points from alarm, SCADA, and waveform data; and simulating sensor failures by randomly dropping 10%, 20%, and 30% of entire SCADA or waveform channels. These augmentations generate nine additional datasets, which, together with the original data, result in a total of 115,970 training samples.

To evaluate generalization capability, four unseen topologies are selected for testing (Fig. 1c). For each topology, fault scenarios are generated using the same procedure as for the training set—including all fault locations, fault types, fault resistances, and the same protection-failure enumeration method—resulting in 3890 distinct base fault scenarios. Using identical noise, sampling-loss, and sensor-failure augmentation strategies, 10 corresponding test datasets are constructed, forming the final test set (Supplementary Table 3).

This dataset, incorporating diverse topologies, fault locations, fault types, fault resistances, and protection failures, together with multi-level augmentation strategies, comprehensively represents the complexities and uncertainties of real-world substation operation.

### Comparison of fault diagnosis models

The substation fault diagnosis problem is formulated as a multi-task learning framework (Supplementary Notes, Supplementary Fig. 1). The model takes system topology, alarm information, fault waveform recordings, and SCADA measurements as inputs, and jointly predicts fault location (multi-class classification), fault type (multi-class classification), and protection failure (multi-label classification).

The proposed model is implemented in PyTorch and trained on a workstation equipped with an Intel(R) Xeon(R) CPU E3-1230 v5 and an NVIDIA RTX 3090 GPU. Model training is conducted in two stages. In the first stage, a denoising autoencoder (DAE) is pretrained for 100 epochs to learn noise-robust representations, using the Adam optimizer with a learning rate of 1 × 10^-3^. In the second stage, the training strategy is task-dependent. For the fault-location and fault-type classification tasks, the self-attention module is first trained with Supervised Contrastive Learning (SCL) for 100 epochs, followed by 50 epochs of classifier training, both using a reduced learning rate of 1 × 10^-4^. For the protection-failure detection task, the self-attention module and classifier are jointly trained for 150 epochs with the same learning rate of 1  × 10^-4^. Early stopping and L₂ regularization are applied to mitigate overfitting. Multiple batch sizes (8, 16, 32, 64) are evaluated, and a batch size of 32 is selected based on the highest validation accuracy (Supplementary Table 4). Notably, each batch consists of one raw fault sample and its nine augmented variants (noise-corrupted, sampling-loss, and sensor-failure versions). This batch structure is deliberately designed to support both DAE pretraining—by providing paired clean and perturbed samples—and the SCL stage, where each raw–augmented pair forms a positive example while the remaining samples in the batch act as negatives.

Seven baseline models are implemented for performance comparison. Temporal constrained fuzzy Petri net (TCFPN)^12^ is a rule-based approach that relies solely on alarm information to identify fault locations and protection failures. It builds upon an improved Petri Net–based framework and represents a typical rule-based solution for substation fault diagnosis. However, the model requires manual updates whenever the system topology changes. The MPNN^21^ model is a data-driven method that utilizes system topology and fault waveform data to predict fault locations and types. Although originally designed for microgrid fault diagnosis, its modeling framework can be readily extended to substations and represents a state-of-the-art graph-based data-driven approach. The MLP-based, ResNet-based, LSTM-based, GCN-based, and Transformer-based models are constructed by replacing the GAT module in the proposed framework with the corresponding architectures, representing common deep learning baselines. For fair comparison, all data-driven models are trained under identical optimization settings and data inputs, and their hyperparameters and parameter counts are adjusted to maintain comparable model complexity (Supplementary Tables 5 and 6).

All models are evaluated on ten test datasets corresponding to different data degradation conditions, which emulate practical disturbances in communication and measurement commonly encountered in substations. For quantitative evaluation, three diagnostic subtasks are assessed independently. Accuracy is used as the primary performance metric in the main text, while additional metrics, including precision, recall, and F1-score on both training and test datasets, are provided in the supplementary material (Supplementary Data). Each experiment is repeated under five random seeds, and results are reported as mean ± standard deviation to evaluate model stability.

The proposed model consistently outperforms all baseline approaches under varying levels of noise, sampling loss, and sensor failure (Tables 1,2,3, Fig. 1d–f, Supplementary Fig. 3–5, Supplementary Data). While it incurs moderate additional computational overhead (Supplementary Table 7), the overall training and inference costs remain within a practically deployable range. From an operational perspective, the observed accuracy–efficiency trade-off should be interpreted in the context of practical substation workflows. Protection relays typically isolate faults within milliseconds, and subsequent diagnostic analysis is conducted after system stabilization using monitoring data collected from multiple sources. Under such post-event conditions, both the proposed model and the baseline methods operate within a sub-second inference range, which is sufficient for practical deployment. Given the safety-critical nature of substations, even moderate improvements in diagnostic accuracy can enhance operational reliability and reduce the risk of misdiagnosis or missed faults. Therefore, while computational efficiency remains an important consideration, the additional resource requirements of the proposed model are considered acceptable in applications where diagnostic robustness and precision are prioritized.

**Table 1:** Fault location identification accuracy (%)

Method	Original test dataset	SNR 50 dB	SNR 40 dB	SNR 30 dB	Sample loss 10%	Sample loss 20%	Sample loss 30%	Sensors failure 10%	Sensors failure 20%	Sensors failure 30%
TCFPN^12^	67.92	67.92	67.92	67.92	62.08	54.45	47.07	67.92	67.92	67.92
MPNN^21^	93.01 ± 0.42	93.02 ± 0.45	93.03 ± 0.44	92.95 ± 0.38	92.67 ±0.29	92.14 ± 0.36	91.94 ± 0.46	91.08 ± 0.26	89.39 ± 0.43	85.12 ± 0.46
MLP-based	0.8711 ± 1.19	87.09 ± 1.14	87.11 ± 1.17	86.99 ± 1.05	86.83 ±1.03	86.23 ± 0.90	85.79 ± 0.90	86.48 ± 1.04	85.33 ± 1.13	84.10 ± 0.89
ResNet-based	96.69 ± 0.37	96.70 ± 0.36	96.68 ± 0.34	96.59 ± 0.32	96.56 ±0.49	96.01 ± 0.42	95.63 ± 0.62	95.88 ± 0.71	94.65 ± 0.73	92.58 ± 0.96
LSTM-based	0.6131 ± 4.85	61.33 ± 4.87	61.30 ± 4.82	61.19 ± 4.94	60.92 ±5.07	60.30 ± 4.99	59.44 ± 4.92	54.98 ± 3.29	50.52 ± 2.91	45.34 ± 2.67
GCN-based	95.93 ± 0.68	95.93 ± 0.65	95.90 ± 0.63	95.77 ± 0.63	95.78 ±0.58	95.18 ± 0.80	94.95 ± 0.87	95.65 ± 0.67	94.83 ± 0.62	93.80 ± 0.65
Transformer-based	87.34 ± 2.43	87.34 ± 2.42	87.31 ± 2.40	87.23 ± 2.23	87.32 ±2.35	86.90 ± 2.68	86.51 ± 2.72	85.30 ± 2.01	82.10 ± 1.90	78.03 ± 1.77
Proposed	97.56 ± 0.23	97.58 ± 0.25	97.56 ± 0.25	97.49 ± 0.21	97.29 ±0.27	96.94 ± 0.23	96.61 ± 0.25	97.18 ± 0.22	96.62 ± 0.22	95.47 ± 0.22

**Table 2:** Fault type classification accuracy (%)

Method	Original test dataset	SNR 50 dB	SNR 40 dB	SNR 30 dB	Sample loss 10%	Sample loss 20%	Sample loss 30%	Sensors failure 10%	Sensors failure 20%	Sensors failure 30%
TCFPN^12^	N/A	N/A	N/A	N/A	N/A	N/A	N/A	N/A	N/A	N/A
MPNN^21^	99.99 ± 0.02	99.99 ± 0.02	99.99 ± 0.02	99.99 ± 0.02	99.9 ± 0.04	99.84 ± 0.04	99.57 ± 0.06	99.48 ± 0.12	98.61 ± 0.08	97.35 ± 0.20
MLP-based	93.10 ± 1.45	93.11 ± 1.43	93.12 ± 1.43	93.06 ± 1.46	92.85 ± 1.20	92.74 ± 1.15	92.55 ± 1.05	90.93 ± 1.22	88.90 ± 1.42	85.54 ± 1.57
ResNet-based	99.72 ± 0.23	99.72 ± 0.22	99.75 ± 0.21	99.79 ± 0.18	99.55 ± 0.15	99.47 ± 0.19	99.19 ± 0.25	98.05 ± 0.21	96.77 ± 0.41	95.17 ± 0.47
LSTM-based	85.74 ± 2.04	85.74 ± 2.03	85.78 ± 2.03	85.92 ± 2.03	85.65 ± 2.10	85.50 ± 2.18	85.32 ± 2.31	57.84 ± 2.35	44.98 ± 2.59	33.86 ± 2.41
GCN-based	99.87 ± 0.13	99.87 ± 0.13	99.87 ± 0.13	99.86 ± 0.11	99.76 ± 0.17	99.56 ± 0.19	99.54 ± 0.15	98.54 ± 0.11	97.46 ± 0.14	95.99 ± 0.29
Transformer-based	92.02 ± 1.17	92.02 ± 1.20	92.02 ± 1.26	91.90 ± 1.20	91.87 ± 1.19	91.59 ± 1.06	91.25 ± 0.93	89.60 ± 1.17	86.09 ± 1.13	80.62 ± 1.02
Proposed	100 ± 0.00	100 ± 0.00	100 ± 0.00	100 ± 0.00	99.98 ± 0.03	99.94 ± 0.05	99.85 ± 0.09	99.57 ± 0.11	98.85 ± 0.17	97.78 ± 0.14

**Table 3:** Protection failure detection accuracy (%)

Method	Original test dataset	SNR 50 dB	SNR 40 dB	SNR 30 dB	Sample loss 10%	Sample loss 20%	Sample loss 30%	Sensors failure 10%	Sensors failure 20%	Sensors failure 30%
TCFPN^12^	69.23	69.23	69.23	69.23	60.54	49.77	40.82	69.23	69.23	69.23
MPNN^21^	N/A	N/A	N/A	N/A	N/A	N/A	NA	NA	NA	NA
MLP-based	84.82 ± 0.95	84.81 ± 0.95	84.77 ± 0.94	84.71 ± 1.01	82.38 ± 1.16	78.35 ± 1.09	74.42 ± 1.28	83.94 ± 0.65	82.25 ± 0.58	79.03 ± 0.52
ResNet-based	91.63 ± 0.43	91.65 ± 0.42	91.71 ± 0.41	91.69 ± 0.41	89.26 ± 0. 41	85.32 ± 0.61	81.52 ± 0.70	91.28 ± 0.50	89.96 ± 0.58	87.64 ± 0.72
LSTM-based	80.25 ± 2.24	80.17 ± 2.28	80.16 ± 2.24	79.86 ± 2.17	73.51 ± 1.21	67.19 ± 1.05	62.77 ± 1.35	75.12 ± 1.44	70.03 ± 1.11	61.96 ± 0.96
GCN-based	91.60 ± 0.53	91.60 ± 0.53	91.55 ± 0.50	91.36 ± 0.45	89.04 ± 0.64	84.69 ± 0.87	81.20 ± 0.63	91.17 ± 0.59	89.65 ± 0.54	87.65 ± 0.63
Transformer-based	74.93 ± 0.92	74.93 ± 0.94	74.92 ± 0.90	74.75 ± 0.91	67.76 ± 1.67	62.93 ± 2.82	59.07 ± 2.74	73.12 ± 0.96	70.83 ± 1.08	65.40 ± 0.91
Proposed	92.75 ± 0.30	92.77 ± 0.38	92.72 ± 0.38	92.62 ± 0.38	90.46 ± 0.45	86.30 ± 0.56	82.61 ± 0.59	92.13 ± 0.25	91.51 ± 0.31	89.62 ± 0.54

The comparative results can be interpreted from both structural modeling capability and architectural design perspectives.

TCFPN relies solely on alarm information and performs rule-based reasoning without learning from multi-modal data. Although it reflects predefined protection logic, multiple fault scenarios may produce highly similar alarm patterns, and rule-based inference cannot resolve such ambiguities. Consequently, its outputs remain largely invariant across certain test conditions, indicating limited discriminative capability and weak adaptability to noisy or incomplete inputs.

MLP-, LSTM-, and Transformer-based models do not explicitly encode electrical topology. MLP treats inputs as flattened feature vectors, LSTM emphasizes temporal dependencies, and Transformer models token-level relationships through self-attention. While these architectures possess strong representation capacity, they lack structural inductive bias reflecting electrical connectivity. Therefore, when system topology changes, their learned correlations may not generalize effectively, resulting in reduced robustness under unseen topology configurations. ResNet-based model enhances feature extraction through convolutional operations and achieves relatively competitive performance. However, convolution is defined on regular Euclidean grids and cannot accurately represent the irregular and non-uniform connectivity of substation components. As a result, although ResNet captures local spatial patterns, it cannot explicitly model topology-dependent fault propagation paths.

Among the graph-based models, MPNN explicitly incorporates topology through message passing between connected nodes and utilizes waveform information for diagnosis. This structural modeling enables better performance than non-graph models in topology-sensitive tasks. However, MPNN does not incorporate alarm information and is trained in a single end-to-end manner without staged pretraining, which limits its ability to diagnose protection failures and reduces robustness under noisy or incomplete inputs. The GCN-based model also encodes topology via graph convolutions and achieves a stronger overall performance than MPNN. By aggregating neighborhood information layer by layer, GCN more effectively captures structural dependencies among components. Nevertheless, its aggregation mechanism is homogeneous, applying fixed normalization and uniform weighting to neighboring nodes. This limitation makes it less effective than GAT in modeling the directional and heterogeneous dependencies among substation components. In contrast, the proposed GAT-based fusion mechanism performs adaptive, node-wise attention and integrates topology, waveform, SCADA, and alarm information more effectively, yielding the highest overall accuracy.

In addition to architectural differences, it is important to analyze how different data degradation mechanisms affect model performance. To further understand this effect, we compare model performance under sample loss and sensor failure conditions. The impacts of sample loss and sensor failure differ due to their distinct degradation mechanisms. Sample loss removes partial time-series data across alarm, SCADA, and waveform modalities, whereas sensor failure drops entire electrical measurement channels while preserving alarm information. Therefore, the performance variation depends on the modality importance of each task. For fault-location, most models rely more on electrical measurements, making sensor failure more detrimental, except for the TCFPN model, which depends solely on alarm data. For fault-type classification, electrical measurements dominate, and sensor failure leads to greater degradation. In contrast, protection failure detection relies primarily on alarm signals, making it more sensitive to sample loss.

### Ablation study

To further assess the contribution of each data source and architectural component to the overall performance, an ablation study was conducted. Specifically, the effects of removing individual modules are examined, including the alarm, fault waveform, and SCADA feature extractors, as well as the self-attention layer, DAE pretraining, and SCL training stage, to quantify their influence on downstream fault diagnosis tasks.

**Fig. 2:**
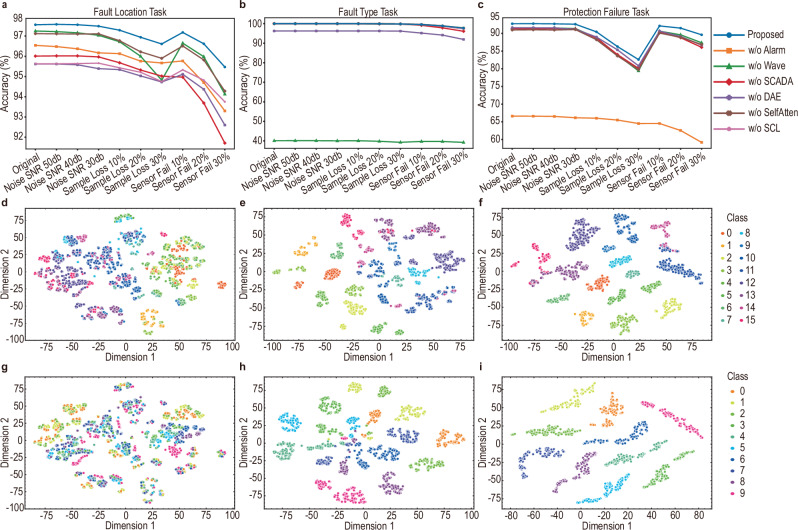
Ablation study results. **a** Performance of the ablation variants on the fault-location task. The variants include without Alarm, without Waveform, without SCADA, without DAE, without self-attention, without SCL, and the Proposed Model. **b** Performance of the ablation variants on the fault-type classification task under the same evaluation conditions. **c** Performance of the ablation variants on the protection-failure detection task. All models in (**a**–**c**) are evaluated on the original test dataset, noise-corrupted datasets (SNR = 50, 40, 30 dB), sampling-loss conditions (10%, 20%, 30%), and sensor-failure conditions (10%, 20%, 30%). Line plots report the mean accuracy across 5 different random seeds. **d** t-SNE visualization of the encoder-output feature embeddings for the fault-location classification task. **e** t-SNE visualization of the feature embeddings after self-attention (SA) fusion for the fault-location classification task. **f** t-SNE visualization of the feature embeddings after applying self-attention with supervised contrastive learning (SA + SCL) for the fault-location classification task. In panels (**d**–**f**), labels 0–15 correspond to the 16 fault-location categories. **g** t-SNE visualization of the encoder-output feature embeddings for the fault-type classification task. **h** t-SNE visualization of the feature embeddings after SA for the fault-type classification task. **i** t-SNE visualization of the feature embeddings after SA + SCL for the fault-type classification task. In panels (**g**–**i**), labels 0–9 correspond to the 10 fault-type categories. All t-SNE plots (**d**–**i**) are generated using identical hyperparameters (perplexity = 30, learning rate = 200, iterations = 1000). The use of self-attention improves cluster compactness, while the addition of supervised contrastive learning further enhances intra-class compactness and inter-class separability, leading to more structured and distinguishable feature clusters. Source data are provided as a Source Data file.

The results demonstrate that all data sources contribute positively to diagnostic performance across different tasks, enhancing the model’s overall accuracy and robustness (Fig. 2a–c and Supplementary Figs. 6–8). For fault location, the diagnostic process relies on a more comprehensive integration of heterogeneous information sources. Removing any single data source leads to a noticeable decrease in accuracy (Fig. 2a). This indicates that accurate localization requires both the transient electrical characteristics captured by waveform data and the complementary contextual information provided by alarm and SCADA signals. For fault type diagnosis, waveform recordings remain the most influential factor. Excluding waveform data results in a dramatic performance reduction (Fig. 2b), reflecting that transient fault waveforms contain the most discriminative temporal–spatial signatures for distinguishing fault types. In contrast, removing alarm information or SCADA measurements produces only minor accuracy changes (Fig. 2b), suggesting that these sources have a limited direct impact on identifying fault type. For protection failure diagnosis, alarm information proves to be the dominant source, as removing it causes a significant degradation in accuracy (Fig. 2c). This highlights that alarm signals, which reflect the operational status and timing behavior of the secondary protection system, are essential for accurately identifying malfunctioning protection devices.

The results also show that all architectural components contribute to performance improvement, confirming the effectiveness of each module in enhancing feature quality and diagnostic robustness (Fig. 2a–c). Removing the DAE pretraining leads to a noticeable decline in accuracy, as the DAE facilitates robust feature learning by mitigating the effects of noise and missing data through denoising and high-dimensional representation extraction. This pretraining step enhances the model’s fault tolerance and overall feature consistency. Eliminating the self-attention layer also results in degraded performance, indicating its essential role in capturing cross-domain dependencies among multi-source features and selectively emphasizing the most informative representations for fault diagnosis. The SCL stage, which is applied only to fault location and type classification tasks, further improves diagnostic performance by enhancing feature discriminability across different fault modes. Removing this stage reduces model adaptability and classification accuracy (Fig. 2a, b).

To investigate how self-attention fusion and SCL influence the structure of the learned feature space, we visualize the embeddings using t-SNE with fixed hyperparameters (perplexity = 30, learning rate = 200, iterations = 1000) (Fig. 2d–i). It is important to note that t-SNE^45^ is a nonlinear, locally preserving embedding method; therefore, the distances in its 2D projected space do not reflect the true geometry of the original high-dimensional feature space^46^. Consequently, t-SNE is used only for qualitative visualization, whereas all quantitative metrics are computed in the original feature space.

To rigorously evaluate clustering quality in the two multi-class tasks, several metrics explicitly supporting multi-class settings are adopted, including the silhouette score, intra-class distance, inter-class distance, and their ratio (Supplementary Methods, Supplementary Equation (3)-(11)). The quantitative results reveal consistent trends across both tasks (Table 4). Self-attention fusion improves cluster compactness compared with the encoder output, while introducing SCL further produces substantially more compact and better-separated feature clusters. For the fault location task, the silhouette score increases from 0.15 (SA) to 0.76 (SA + SCL), and the inter/intra-class distance ratio increases from 1.35 to 7.43. Similar improvements are observed in the fault type classification task. These results demonstrate that SCL significantly enhances the discriminability of the latent feature space, enabling the model to more effectively separate fault categories.

**Table 4:** Quantitative clustering metrics for multi-class feature embeddings under different model configurations

Task	Feature representation	Silhouette score	Intra-class distance	Inter-class distance	Inter/Intra
Fault location	Encoder	0.0269	4.5099	5.2125	1.1558
Fault location	SA without SCL	0.1548	109.4283	148.1606	1.3540
Fault location	SA with SCL	0.7600	15.7616	117.0393	7.4256
Fault type classification	Encoder	0.0009	4.9346	5.1503	1.0437
Fault type classification	SA without SCL	0.1697	89.6750	124.9386	1.3932
Fault type classification	SA with SCL	0.7309	17.0048	85.7115	5.0404

### Digital twin application platform

A digital twin system of the 110 kV substation is developed using the CloudPSS platform for intelligent fault diagnosis. The digital twin is deployed on a station-level workstation, where data are collected from intelligent electronic devices (IEDs) via the IEC 61850 communication protocol (Supplementary Note). This configuration enables dynamic interaction between operational data and the digital twin system, enhancing diagnostic efficiency and reliability.

**Fig. 3:**
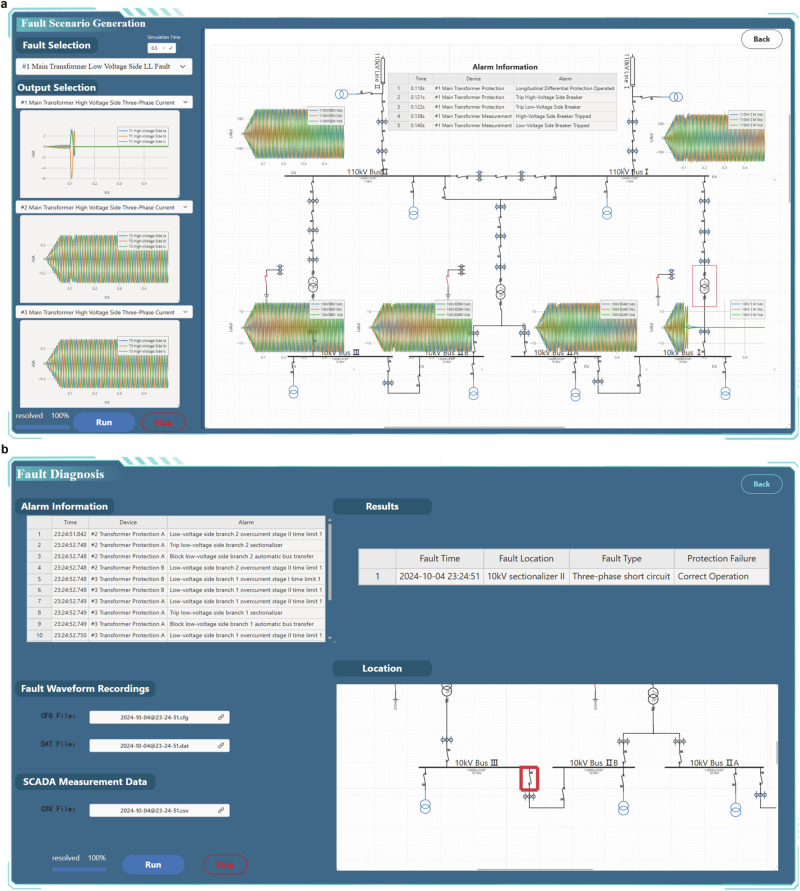
Data generation and fault diagnosis modules of the digital twin platform. **a** Automated fault scenario generation module used to create diverse fault cases for training and validation, ensuring broad coverage of operating conditions and fault patterns. **b** Fault diagnosis module integrating the proposed learning framework to perform fast fault localization, fault type classification, and protection failure analysis within the digital twin environment.

The digital twin platform consists of four core functional modules that correspond to different submodels within the CloudPSS environment (Fig. 3 and Supplementary Fig. 11). The primary system model reproduces the electrical network configuration, including power sources, busbars, main transformers, loads, capacitors, grounding transformers, circuit breakers, and instrument transformers (CTs/PTs), to simulate the operational behavior of primary equipment (Supplementary Fig. 11a). The secondary protection system model is built according to the actual relay configurations and protection logic used in the substation, enabling accurate replication of secondary device interactions (Supplementary Fig. 11b). The fault scenario generation module automatically produces diverse fault cases for model training and validation, ensuring wide coverage of operating and fault conditions (Fig. 3a). The fault diagnosis module integrates the proposed learning model for rapid and accurate fault localization, type identification, and protection failure analysis, thereby enabling fast intelligent diagnosis within the digital twin environment (Fig. 3b).

To enhance diagnostic reliability and support effective human–machine collaboration, a confidence-based alarm verification mechanism is integrated into the overall diagnostic framework. In this mechanism, the model outputs the predicted labels together with the corresponding confidence scores produced by the final softmax or sigmoid layers for the three diagnostic tasks of fault type classification, fault location, and protection failure detection. A unified confidence threshold of 0.90 is applied. Predictions with confidence values higher than this threshold are regarded as reliable and do not trigger an alarm. Predictions with lower confidence values are treated as uncertain and will trigger an alarm so that the case is escalated for operator review. The outputs from the three diagnostic tasks are further ranked according to their confidence scores, with higher-confidence predictions assigned higher inspection priority, thereby guiding maintenance personnel to prioritize inspection actions accordingly. This mechanism ensures that potentially unreliable inferences receive human attention and thus improves the operational dependability of the diagnostic process.

In addition to confidence-based verification, the robustness of the diagnostic model under incomplete measurements was evaluated through a stress test using nineteen datasets with 10%–90% sampling loss and 10%–90% sensor failures (Supplementary Table 8). The results show that the three diagnostic tasks have distinct tolerance limits. Fault type classification is the most resilient, maintaining above 95% accuracy even with 90% sampling loss and above 90% accuracy under 40% sensor failures. Fault location is moderately sensitive: its accuracy stays above 90% when sampling loss is within 60% and above 85% when sensor failures are within 40%, but drops sharply beyond these levels (e.g., 63.2% at 90% sampling loss and 59.4% at 80% sensor failure). Protection failure detection is the most vulnerable, remaining stable only when missing data is below roughly 30%, but falling below 50% under extreme degradation, such as ≥60% missing data. These differences can be attributed to the nature of the tasks: fault location diagnosis focuses on the spatial distribution and inter-node relationships of electrical features across the network, and therefore requires sufficient measurements from multiple nodes around the faulted area to maintain spatial observability; fault type classification mainly relies on the characteristic patterns of three-phase and zero-sequence currents, which are global fault signatures and remain distinguishable even when a large portion of measurements is missing; in contrast, protection failure detection depends primarily on protection-related alarm and event information, and any loss of such alarm signals is likely to directly result in misdiagnosis, making this task inherently more sensitive to missing data. Overall, the observations indicate practical robustness thresholds of approximately 60% sampling loss or 40% sensor failure for fault location, approximately 90% sampling loss or 40% sensor failure for fault type classification, and roughly 30% missing data for reliable protection-failure detection.

A real fault event that occurred at the substation was used to validate the proposed fault diagnosis method. During operation, the station level continuously received the real-time switching states of circuit breakers, disconnectors, and grounding switches from bay-level IEDs, ensuring that the pre-fault topology was already available when the fault occurred. Following the fault, the station level immediately obtained the sequence-of-events (SOE) alarms (Supplementary Table 9) and SCADA analog measurements, both of which were transmitted within milliseconds and therefore did not contribute noticeably to the diagnostic latency. In contrast, the acquisition of fault waveforms was substantially slower. The protection IEDs and fault recorders needed to capture the pre-fault window and the complete transient before generating COMTRADE files, and this file-generation step dominated the overall delay. In the 110 kV smart substation used for validation, generating the waveform files from multiple triggered recorders required approximately 50 s, after which the files (about 20 MB each) were uploaded to the station level within roughly 10 s. Once all required waveform files were available, the digital twin system reconstructed the pre-fault topology, parsed and organized the SOE, SCADA, and waveform data, and produced the model inputs, all within about 1 s. The diagnostic model then performed inference on the workstation within approximately 0.5 s. As SOE and SCADA reporting overlap with waveform acquisition, the end-to-end diagnostic latency is dominated by the recorder-side waveform file-generation time (Supplementary Notes).

The model identified the fault as a three-phase short circuit at the 10 kV sectionalizer II, with backup protection on the low-voltage side of transformers #2 and #3 operating correctly to isolate the faulted section. On-site inspection confirmed the diagnosis results. To further validate the model’s reliability, a digital twin simulation of the same event was conducted on the CloudPSS platform. The simulated current waveforms closely matched the measured recordings (Supplementary Fig. 9), demonstrating both the fidelity of the digital twin model and the robustness of the proposed diagnostic framework. The recorded real-world fault data have been archived and incorporated into the dataset for subsequent model fine-tuning and performance optimization, ensuring continuous improvement of diagnostic accuracy under practical operating conditions.

## Discussion

This study developed a digital twin–driven multi-modal fusion fault diagnosis framework for power substations, integrating system topology, alarm information, fault waveforms, and SCADA measurements to achieve accurate and comprehensive diagnosis. The proposed model employs GATs and the self-attention mechanism to improve feature extraction and fusion, resulting in more informative and robust representations of substation fault scenarios. A systematic sample generation approach ensures robust training, while DAE pretraining and task-specific downstream learning substantially improve generalization and robustness to noisy or missing data. The digital twin framework enables seamless integration with real substations, supporting fast fault diagnosis and decision assistance. Experimental validation confirms the model’s high diagnostic accuracy, adaptability to topology variations, and resilience under noisy and incomplete data conditions. The successful deployment at a 110 kV substation further demonstrates its engineering feasibility and practical value.

The proposed framework also exhibits strong transferability across substations. When applied to a new substation, the main tasks include constructing or adapting its simulation model, generating batch training data, and performing model retraining and validation. Owing to the high standardization of smart substation designs, most existing digital twin models can be reused with only minor adjustments to electrical parameters and protection configurations. With the CloudPSS platform, large-scale training datasets can be efficiently generated, enabling rapid adaptation of the fault diagnosis model to new substations. Thus, the proposed framework offers a scalable and generalizable solution for smart substation fault diagnosis and maintenance.

Future work will focus on two main directions. First, we aim to enhance the scalability of the proposed method to accommodate larger and more complex power systems, such as DC converter stations and distribution networks. This will involve adapting the digital twin modeling and training workflow to support more diverse system topologies and protection configurations, ensuring efficient deployment across different grid levels. Second, we plan to extend the multi-modal fusion capability by integrating visual data collected from substation inspection robots. This will provide complementary spatial and contextual information for enhanced situational awareness. The visual data can be incorporated into the existing framework by adding a visual feature extraction branch parallel to the existing input encoders. This extension will require additional DAE pretraining to adapt visual features to the shared latent space. To support training, visual datasets will be generated from a 3D digital model of the substation, dynamically linked with the electromagnetic simulation in CloudPSS to ensure physical and temporal consistency between visual scenes and system states.

## Methods

### Digital twin-driven fault diagnosis framework

**Fig. 4:**
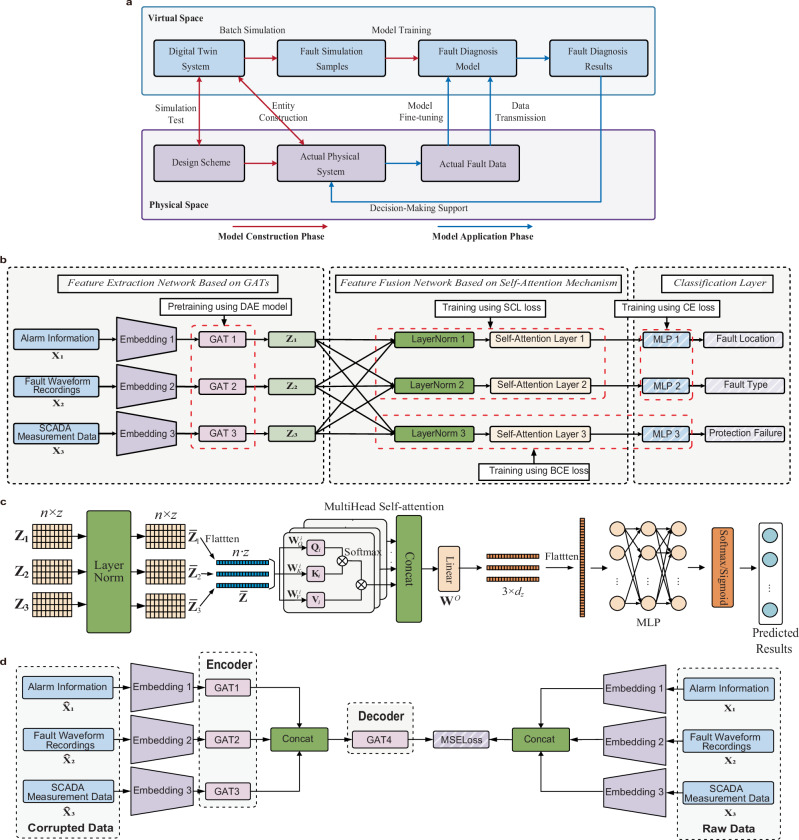
Overall framework and model architecture of the proposed method. **a** Digital twin–driven fault diagnosis framework. In the model construction phase, a digital twin is built to simulate operating and fault scenarios and to support training of the multi-modal fusion model; in the model application phase, real-time substation data are synchronized to the virtual space for fault inference, decision support, and continuous model refinement, forming a closed-loop cyber–physical system. **b** Architecture and training strategy of the proposed model. The model comprises a GAT-based multi-modal feature extractor, a task-specific self-attention fusion module, and MLP classifiers. After DAE pretraining for noise-robust initialization, downstream training optimizes the self-attention layers with SCL and the classifier with CE loss for fault location and fault-type tasks, while the multi-label protection-failure task is trained directly using BCE loss. **c** Self-attention-based feature fusion network. Alarm, waveform, and SCADA features are normalized, concatenated, and fused via multi-head self-attention to model global cross-modal dependencies, followed by an MLP for task-specific prediction (Softmax for location/type; Sigmoid for protection failure). **d** DAE pretraining structure. A GAT-based encoder–decoder DAE is used to learn noise-robust latent representations from augmented multi-modal inputs.

The proposed digital twin-driven fault diagnosis framework aims to achieve accurate and adaptive fault diagnosis in power substations through the integration of virtual simulation and multi-modal fusion learning, thereby facilitating practical engineering applications. The framework operates in two main phases: the model construction phase and the model application phase, forming a closed-loop cyber–physical system for continuous model optimization and fast fault diagnosis (Fig. 4a).

In the model construction phase, the foundation of the fault diagnosis framework is established. A virtual simulation environment is developed using digital twin technology to replicate the substation’s primary and secondary systems. This digital replica enables comprehensive testing of system behavior and performance evaluation under diverse operational and fault conditions. Based on this virtual model, a variety of fault scenarios are systematically generated to construct a diverse and representative training dataset, ensuring the model’s robustness and generalization capability. Subsequently, a multi-modal fusion fault diagnosis model is built according to the substation’s system configuration to effectively capture correlations among heterogeneous data sources. The model is then trained using the generated dataset, enabling it to perform accurate fault inference across complex operating scenarios. Finally, the virtual model is seamlessly integrated with the physical system, establishing a cyber–physical collaborative environment that supports real-time monitoring and feedback.

In the model application phase, the framework transitions from the virtual domain to real-world operation. Real-time operational and fault data from the physical substation are continuously transmitted to the virtual model through the digital twin system deployed at the station level (Supplementary Fig. 1a). At this level, real-time data are collected from both process-level and station-level networks via various sensors and IEDs. The trained multi-modal fusion model processes these streaming data to infer potential fault causes and provide accurate diagnostic and decision support for maintenance personnel. Moreover, the model’s robustness to noisy or incomplete data ensures reliable fault identification in complex environments. As real-world fault data accumulate, the model is further fine-tuned to enhance diagnostic accuracy and adaptability to evolving operating conditions.

Overall, this digital twin-driven framework provides a systematic, scalable, and adaptive solution for fault diagnosis in substation engineering applications. By integrating digital twin technology with multi-modal fusion learning, it enables precise, data-driven decision-making and continuous performance improvement in real-world operations.

### Multi-modal fusion fault diagnosis model

To overcome the limitations of single-modality diagnostic approaches, a multi-modal fusion fault diagnosis model is developed based on GATs and the self-attention mechanism. This model effectively integrates heterogeneous data sources to achieve comprehensive and accurate fault diagnosis in power substations.

The proposed model comprises three core components: a fault feature extraction network based on GATs, a feature fusion network employing the self-attention mechanism, and a classification layer (Fig. 4b). The feature extraction network processes raw data from multiple modalities, embedding and extracting discriminative features specific to each data type. The feature fusion network then integrates these features through self-attention, enabling cross-modal interactions that retain critical information and enhance diagnostic precision. Finally, the classification layer receives the fused feature representations and performs fault classification. By systematically integrating complementary information across modalities, this architecture significantly improves the robustness and accuracy of fault diagnosis, providing a unified framework for multi-source data analysis in substation systems.

### Feature extraction networks based on GATs

GAT is a type of graph neural network that integrates the attention mechanism into the message-passing process^47^. GATs are inherently suitable for processing graph-structured data, enabling efficient modeling of complex topological relationships and inter-node dependencies. Their adaptability to dynamic topologies allows the model to effectively utilize both the substation’s primary topology information and diverse fault data, thereby extracting high-quality feature representations. This enhances the topological generalization capability of the model and improves fault diagnosis accuracy.

In a GAT, each node updates its own feature representation by aggregating information from its neighboring nodes. The updating formula is as follows:

1 $${{{{\bf{h}}}}}_{i}^{(l+1)}=\sigma \left( \, {\sum }_{j\in N(i)}{\alpha }_{ij}^{(l)}{{{{\bf{W}}}}}^{(l)}{{{{\bf{h}}}}}_{j}^{(l)}\right)$$

In Eq. (1), ${{{{\bf{h}}}}}_{i}^{(l)}$ represents the feature vector of node *i* at layer *l*; $N(i)$ is the set of neighboring nodes of node *i*; ${\alpha }_{ij}$ is the attention weight, indicating the influence of neighboring node *j* on node *i*; ${{{\bf{W}}}}$ is the learnable linear transformation matrix; and $\sigma$ is the activation function, typically using the ReLU function.

The attention weight ${\alpha }_{ij}$ calculation is as follows:

2 $${\alpha }_{ij}=\frac{\exp ({e}_{ij})}{{\sum }_{g\in N(i)}\exp ({e}_{ig})}$$

In Eq. (2), ${e}_{ij}$ is the attention coefficient, calculated by the following formula:

3 $${e}_{ij}={{{\rm{LeakyReLU}}}}\left({{{{\bf{a}}}}}^{T}\left[{{{\bf{W}}}}{{{{\bf{h}}}}}_{i}\parallel {{{\bf{W}}}}{{{{\bf{h}}}}}_{j}\right]\right)$$

In Eq. (3), ${{{\bf{a}}}}$ is a learnable attention vector; ${{{\bf{W}}}}$ is a learnable linear transformation matrix consistent with the one in Eq. (1); $\parallel$ denotes feature concatenation; and LeakyReLU is the activation function. To improve robustness and expressiveness, GAT employs a multi-head attention mechanism, where features are obtained by concatenating or averaging the outputs from multiple heads.

Based on the primary topology of the substation, multi-layer GATs are employed to extract structured features from three heterogeneous data sources: alarm information, fault waveform recordings, and SCADA measurements.

For alarm information, the Temporal Constrained Fuzzy Petri Net (TCFPN)^12^ is first employed to infer a confidence vector representing the actions of the *p* protection devices. This vector is expanded into a matrix ${{{{\bf{E}}}}}_{1}\in {{\mathbb{R}}}^{n\times p}$, where *n* denotes the number of primary-system nodes corresponding to the possible fault locations. The matrix **E**_1_ is subsequently fed into the GATs to obtain the alarm-based feature matrix **Z**_1_ (Supplementary Fig. 12a).

For the fault waveform recordings, the RMS values of the three-phase currents and the zero-sequence currents are computed over a window consisting of 5 pre-fault cycles and 20 post-fault cycles. After normalization, these values form the matrix ${{{{\bf{E}}}}}_{2}\in {{\mathbb{R}}}^{n\times 100}$, where each of the *n* nodes is associated with 100 temporal features. Processing **E**_2_ through the GATs yields the waveform-based feature matrix **Z**_2_ (Supplementary Fig. 12b).

Similarly, SCADA measurements—three-phase and zero-sequence currents at each primary node, including 1 pre-fault and 19 post-fault sampling points—are normalized into the input matrix ${{{{\bf{E}}}}}_{3}\in {{\mathbb{R}}}^{n\times 80}$. The GATs then transform this matrix into the SCADA feature representation **Z**_3_ (Supplementary Fig. 12c).

Through these specialized GAT-based extraction networks, the proposed framework effectively captures spatial-topological dependencies and modality-specific characteristics, providing rich and discriminative representations for subsequent feature fusion and fault classification.

### Feature fusion network based on a self-attention mechanism

In the feature fusion network, a self-attention mechanism is employed to model global correlations among multi-modal fault features and adaptively adjust their significance according to the diagnostic task. By leveraging this mechanism, the proposed approach ensures flexible and effective feature fusion, enhancing the accuracy of each fault classification task.

For each fault classification task, the fault feature matrices **Z**_1_, **Z**_2_, and **Z**_3_ from the GAT networks, each of size $n\times z$, are normalized using LayerNorm^48^ to produce ${\bar{{{{\bf{Z}}}}}}_{1}$, ${\bar{{{{\bf{Z}}}}}}_{2}$, and ${\bar{{{{\bf{Z}}}}}}_{3}$ (Fig. 4c), ensuring stable feature distributions across data sources:

4 $${\bar{{{{\bf{Z}}}}}}_{i}=\frac{{{{{\bf{Z}}}}}_{i}-{\mu }_{i}}{{\sigma }_{i}+\varepsilon }\odot \gamma+\beta,i=1,2,3$$

In Eq. (4), ${\mu }_{i}$ and ${\sigma }_{i}$ are the mean and standard deviation of **Z**_*i*_; $\gamma$ and $\beta$ are learnable scaling and bias parameters; $\varepsilon$ prevents division by zero; $\odot$ represents element-wise multiplication.

The normalized matrices ${\bar{{{{\bf{Z}}}}}}_{1}$, ${\bar{{{{\bf{Z}}}}}}_{2}$, and ${\bar{{{{\bf{Z}}}}}}_{3}$ are flattened and concatenated into a single matrix $\bar{{{{\bf{Z}}}}}\in {{\mathbb{R}}}^{3\times (n\cdot z)}$. This matrix is input to a self-attention layer^49^, where it undergoes linear transformations to generate Query (${{{{\bf{Q}}}}}_{i}$), Key (${{{{\bf{K}}}}}_{i}$), and Value (${{{{\bf{V}}}}}_{i}$) for *h* attention heads:

5 $$\left\{\begin{array}{l}{{{{\bf{Q}}}}}_{i}=\bar{{{{\bf{Z}}}}}{{{{\bf{W}}}}}_{Q}^{i}\\ {{{{\bf{K}}}}}_{i}=\bar{{{{\bf{Z}}}}}{{{{\bf{W}}}}}_{K}^{i}\\ {{{{\bf{V}}}}}_{i}=\bar{{{{\bf{Z}}}}}{{{{\bf{W}}}}}_{V}^{i}\end{array}\right.\\ i=1,2,\cdots,h$$

In Eq. (5), ${{{{\bf{W}}}}}_{Q}^{i}$, ${{{{\bf{W}}}}}_{K}^{i}$ and ${{{{\bf{W}}}}}_{V}^{i}$ are the parameter matrices for the *i*-th attention head.

Attention weights for the *i*-th head are calculated and applied to ${{{{\bf{V}}}}}_{i}$:

6 $${{{\rm{head}}}}_{i}={{{\rm{Softmax}}}}\left(\frac{{{{{\bf{Q}}}}}_{i}{{{{\bf{K}}}}}_{i}^{T}}{\sqrt{{d}_{k}}}\right){{{{\bf{V}}}}}_{i}$$

In Eq. (6), ${d}_{k}={d}_{z}/h$ is the feature dimension per head, with ${d}_{z}$ being the output feature dimension. The outputs of *h* heads are concatenated and projected to form the final self-attention output:

7 $${{{\rm{MultiHead}}}}({{{\bf{Q}}}},{{{\bf{K}}}},{{{\bf{V}}}})={{{\rm{Concat}}}}({{{\rm{head}}}}_{1},{{{\rm{head}}}}_{1},\cdots,{{{\rm{head}}}}_{h}){{{{\bf{W}}}}}^{o}$$

In Eq. (7), $hea{d}_{i}\in {{\mathbb{R}}}^{3\times ({d}_{z}/h)}$ is the output of the *i*-th attention head; ${{{{\bf{W}}}}}^{o}$ is the output projection matrix.

The final output of the self-attention layer is flattened and passed through a Multi-Layer Perceptron (MLP) to generate the diagnostic results. Depending on the task, different activation functions are used to produce the final prediction probabilities: Softmax is applied for the multi-class tasks of fault location and fault type, whereas Sigmoid is employed for the multi-label protection failure classification.

### Dataset generation

To ensure the generalization and robustness of the fault diagnosis model, the training dataset is systematically generated through a series of processes involving topology selection, fault scenario generation, and data augmentation.

To enhance the model’s adaptability to diverse network configurations, multiple substation topologies are incorporated into the dataset. These topologies are derived from commonly observed configurations in historical operation data, representing various connection structures and equipment layouts. By including multiple topological variations, the model is trained to recognize fault patterns across different network structures, preventing overfitting to a single configuration and ensuring strong adaptability in complex and unseen operational environments (Supplementary Discussion, Supplementary Fig. 10).

To address the scarcity of real-world fault data and expand the dataset’s coverage, a systematic fault-scenario generation procedure is adopted. For each feasible network topology, the full set of primary-system fault configurations is generated, covering all potential fault locations and fault types. The fault resistance is randomized within 0–10 Ω to introduce variability consistent with typical low-impedance fault conditions. To further account for uncertainties in the secondary system, multiple protection failure conditions are automatically generated using a fault enumeration tree traversal method based on Breadth-First Search (Supplementary Methods, Supplementary Fig. 2). In this approach, a fault enumeration tree is constructed in which each node corresponds to a possible combination of PR failure states. The BFS traversal updates the PR states of child nodes according to the simulated behavior of their parent nodes, thereby producing an exhaustive set of protection-failure combinations. This mechanism effectively emulates fault propagation under diverse PR failure scenarios. Through this systematic process, a diverse and realistic collection of simulated fault cases is obtained, comprehensively capturing potential operational anomalies across both the primary and secondary systems.

To further enhance the realism and robustness of the dataset, multiple data augmentation strategies are applied to the simulated samples, ensuring that the training data better reflect real-world operating conditions and mitigating the risk of model overfitting. Three augmentation mechanisms are designed. First, random Gaussian noise is added to both fault waveform and SCADA measurements to emulate environmental disturbances, sensor precision limitations, and communication noise commonly observed in field deployments. This enables the model to learn noise-tolerant representations and maintain diagnostic reliability under uncertain signal conditions. Second, sampling-loss simulation is performed by randomly removing a subset of time-series data points at each measurement node. This mimics transient communication delays or temporary signal interruptions, compelling the model to infer missing information and improving its resilience to incomplete measurements. Third, sensor-failure simulation is introduced by completely eliminating the measurements from selected sensors to represent sensor malfunctions or disconnections. This ensures that the model remains functional and reliable even when partial sensing devices fail in practice. Finally, the augmentation pipeline is designed to be extensible: real fault cases occurring during actual operation are archived and later incorporated into the framework for fine-tuning and iterative model enhancement. This enables the model to progressively adapt to real operating environments as more field data becomes available.

By integrating diverse topology configurations, exhaustive fault scenario generation, and comprehensive data augmentation, the constructed dataset becomes both rich and representative. This ensures that the proposed fault diagnosis model can generalize effectively across varied system configurations, accurately identify different fault scenarios, and remain resilient to noisy or incomplete data in practical engineering applications.

### Model training strategy

The training strategy of the proposed model is designed to achieve robust feature extraction, effective feature fusion, accurate fault classification, and reliable adaptation to diverse operating conditions. The overall training process consists of two sequential stages (Fig.4b). This training design is motivated by two key considerations. First, substation fault data are inherently noisy, incomplete, and heterogeneous across modalities. Direct end-to-end supervised training under such conditions may lead to unstable optimization and modality bias. Therefore, an unsupervised pretraining stage is introduced to learn noise-robust and topology-aware feature representations before task-specific optimization. Second, the three downstream tasks exhibit different label structures and modality dependencies, requiring tailored optimization strategies to ensure discriminative and stable learning for each task.

In the first stage, a DAE is employed for pretraining to learn robust, noise-tolerant feature representations from multi-modal data. By reconstructing corrupted inputs, the DAE enhances the model’s ability to capture essential fault characteristics and improves its resilience to noise and missing data.

In the second stage, the pretrained feature extraction layers are frozen, and only the feature fusion network and classification layers are trained for specific downstream diagnostic tasks, including fault location, fault type, and protection failure classification. Each task is optimized using a task-specific loss function to ensure targeted performance improvement while leveraging the robust features learned during the pretraining stage.

This two-stage strategy enables the downstream training to start from noise-robust and well-structured feature representations learned by the DAE, leading to faster convergence, improved generalization, and higher diagnostic accuracy under complex and varying substation operating conditions.

### Denoising autoencoder pretraining

To enhance feature extraction from heterogeneous fault data and mitigate the adverse effects of noise and data loss on diagnostic performance, a DAE is introduced to pretrain the feature extraction network. As an unsupervised model capable of learning latent representations through reconstruction^50^, the DAE effectively derives robust features even when inputs are noisy or partially missing. Compared with purely supervised pretraining, the DAE enables the encoder to capture intrinsic structural dependencies and cross-modal correlations without relying on labeled fault categories. Freezing the pretrained encoder during downstream optimization prevents catastrophic forgetting and preserves the learned topology-aware and noise-robust representations, ensuring stable transfer to task-specific learning.

The DAE consists of an encoder and a decoder, both implemented using multi-layer GATs (Fig. 4 d). The encoder receives augmented training samples, which naturally contain corrupted or incomplete data introduced during the augmentation process. It comprises three independent multi-layer GAT branches, each dedicated to a specific data modality. The outputs of these branches are concatenated to form a unified latent feature matrix $\hat{{{{\bf{Z}}}}}=[{{{{\bf{Z}}}}}_{1},{{{{\bf{Z}}}}}_{2},{{{{\bf{Z}}}}}_{3}]\in {{\mathbb{R}}}^{n\times 3z}$, which encodes fused high-dimensional representations across modalities.

The decoder, also GAT-based, takes $\hat{{{{\bf{Z}}}}}$ as input and performs multi-layer feature aggregation to reconstruct the original data, producing $\hat{{{{\bf{E}}}}}$ as an approximation to the complete input matrix **E**. This reconstruction process not only preserves the structural dependencies imposed by the substation’s primary topology but also captures the complementary relationships across different data modalities, ensuring that information from alarms, waveforms, and SCADA measurements can be jointly exploited in a topology-aware manner. The DAE is trained by minimizing the Mean Squared Error (MSE) between the reconstructed and original inputs, defined as follows:

8 $${L}_{{{\rm{MSE}}}}=\frac{1}{N}{\sum }_{i=1}^{N}{\left|\!\left| {{{{\bf{E}}}}}_{i}-{\hat{{{{\bf{E}}}}}}_{i}\right|\!\right| }_{2}^{2}$$

In Eq. (8), ${{{{\bf{E}}}}}_{i}$ and ${\hat{{{{\bf{E}}}}}}_{i}$ denote the original and reconstructed feature matrices of the *i*-th sample, respectively, and *N* is the total number of training samples.

The pretrained encoder parameters are subsequently frozen and reused in the downstream training stage, providing a robust feature representation that accelerates convergence and enhances diagnostic resilience.

### Downstream tasks training

This study focuses on single-point faults, formulating both the primary fault location and fault type diagnosis tasks as multi-class classification problems. To enhance feature discrimination and improve the stability of model optimization, the training of the self-attention layers and classification layers is organized into two consecutive optimization procedures. In the first procedure, SCL^51^ is applied to train the self-attention layers, enhancing the discriminative capability of the learned feature representations. SCL promotes feature separability by pulling samples of the same class closer together while pushing samples of different classes farther apart in the feature space. The SCL loss is defined as:

9 $${L}_{{{\rm{SCL}}}}=\frac{1}{N}{\sum }_{i=1}^{N}\frac{-1}{\left|P(i)\right|}{\sum }_{p\in P(i)}\log \frac{\exp ({{{{\bf{z}}}}}_{i}\cdot {{{{\bf{z}}}}}_{p}/\tau )}{{\sum }_{a\in A(i)}\exp ({{{{\bf{z}}}}}_{i}\cdot {{{{\bf{z}}}}}_{a}/\tau )}$$

In Eq. (9), N is the sample size; *P*(*i*) is the set of positive samples associated with sample *i*; *A*(*i*) is the set of all other samples in the batch excluding sample *i*; **z**(*i*) is the feature representation of sample *i*; $\tau$ is temperature coefficient.

After this discriminative feature enhancement stage, the model proceeds to the classification optimization stage, where the MLP classifier is trained using the Cross-Entropy (CE) loss to enable accurate prediction of fault locations and fault types. The CE loss is expressed as:

10 $${L}_{{{\rm{CE}}}}^{{{{\rm{Y}}}}_{1}}=-\frac{1}{N}{\sum }_{i=1}^{N}{\sum }_{j=1}^{n}[{y}_{1}^{i,j}\,\log (\,{\tilde{y}}_{1}^{i,j})]$$

11 $${L}_{{{\rm{CE}}}}^{{{{\rm{Y}}}}_{2}}=-\frac{1}{N}{\sum }_{i=1}^{N}{\sum }_{j=1}^{m}[{y}_{2}^{i,j}\,\log (\,{\tilde{y}}_{2}^{i,j})]$$

In Eqs. (10) and (11), ${y}_{1}^{i,j}$ and ${y}_{2}^{i,j}$ denote the true class labels for fault location and fault type, respectively; ${\tilde{y}}_{1}^{i,j}$ and ${\tilde{y}}_{2}^{i,j}$ represent the corresponding model-predicted class probabilities. Here, *i* is the index of the training sample, *j* is the index of the class, *n* is the total number of primary nodes in the substation (i.e., the possible fault locations), and *m* is the total number of fault types considered.

For protection failure classification, the task is modeled as a multi-label classification problem, since a single fault event may involve simultaneous failures of multiple protection relays. In this task, the multi-label nature introduces label overlap and dependency among protection relays, making it difficult to define unambiguous positive and negative pairs required for contrastive learning. Therefore, directly optimizing the model using Binary Cross-Entropy (BCE) loss provides a more appropriate objective for capturing independent yet potentially correlated relay failure events. The self-attention layer and classifier are trained directly using the BCE loss, defined as:

12 $${L}_{{{\rm{BCE}}}}^{{{{\rm{Y}}}}_{3}}=-\frac{1}{N}{\sum }_{i=1}^{N}{\sum }_{j=1}^{p}\left[{y}_{3}^{i,j}\,\log \left({\tilde{y}}_{3}^{i,j}\right)+\left(1-{y}_{3}^{i,j}\right)\log \left(1-{\tilde{y}}_{3}^{i,j}\right)\right]$$

In Eq. (12), ${y}_{3}^{i,j}$ denotes the true label for the *j*-th protection relay in the *i*-th sample (with ${y}_{3}^{i,j}$=1 indicating a failure and ${y}_{3}^{i,j}$=0 indicating normal operation), and ${\tilde{y}}_{3}^{i,j}$ represents the predicted failure probability, and *p* is the total number of protection relays in the system.

## Supplementary information

Supplementary Information

Description of Additional Supplementary Files

Supplementary Data

Supplementary Software

Transparent Peer Review file

## Source data

Source Data

## Data Availability

Source data are provided with this paper. The data generated in this study have been deposited in the Code Ocean repository and are publicly available at 10.24433/CO.6548109.v3^52^. Source data are provided with this paper.

## Code availability

The core code is provided with this paper. The code supporting the findings of this study has been deposited in the Code Ocean repository and is publicly available at 10.24433/CO.6548109.v3^52^.
