# Supplementary material for: Digital twin-driven fault diagnosis of power substations by multi-modal fusion learning
Source: Nat Commun. 2026 May 20;17:6628. doi: 10.1038/s41467-026-73483-5 (PMC13381694; doi:10.1038/s41467-026-73483-5)
Supplement: Supplementary file 1 — Supplementary Information [file 41467_2026_73483_MOESM1_ESM.pdf]

Supplementary Information for

Digital twin-driven fault diagnosis of power substations by multi-modal fusion learning

Yulun Wu[1], Ying Chen[1*],Tannan Xiao[2], Lifu Ding[1]

[1] Department of Electrical Engineering, Tsinghua University, Beijing, China

[2] State Key Laboratory of Power System Operation and Control, Tsinghua University, Beijing, China

[*] Corresponding author. E-mail: chen_ying@tsinghua.edu.cn

This file includes:

Supplementary Figures 3

Supplementary Figure 1: Illustration of the fault diagnosis problem in substations 3

Supplementary Figure 2: Example case of the fault enumeration tree traversal method based on breadth-first search 4

Supplementary Figure 3: Test results of models on the fault location task 6

Supplementary Figure 4: Test results of models on the fault type classification task 8

Supplementary Figure 5: Test results of models on the protection failure detection task 10

Supplementary Figure 6: Ablation study results on the fault location task 12

Supplementary Figure 7: Ablation study results on the fault type classification task 14

Supplementary Figure 8: Ablation study results on the protection failure detection task 16

Supplementary Figure 9: Comparison of partial real fault waveforms and simulation waveforms. 17

Supplementary Figure 10: Physical layout and feasible topology space of the studied 110 kV substation 18

Supplementary Figure 11: Primary and secondary system modeling of the digital twin platform 19

Supplementary Figure 12: Detailed feature extraction networks for multi-modal inputs. 20

Supplementary Tables **20**

Supplementary Table 1: Comparison of existing fault diagnosis methods for power systems 20

Supplementary Table 2: Brief protection configuration of a 110kV substation in Shenzhen 22

Supplementary Table 3: Summary of the dataset 23

Supplementary Table 4: Performance of the proposed model in test dataset with different training batch sizes 23

Supplementary Table 5: Hyperparameters of the proposed model 24

Supplementary Table 6: Architecture and parameter counts of all baseline models 25

Supplementary Table 7: Training time, inference time and GPU memory usage of each module across all baseline models 25

Supplementary Table 8: Diagnostic accuracy of the proposed model under increasing sampling-loss and sensor-failure conditions 26

Supplementary Table 9: Alarm information of the real fault case 26

Supplementary Methods **27**

Fault enumeration tree traversal method based on breadth-first search 27

Quantitative clustering metrics for multi-class feature embeddings 31

Supplementary Notes **32**

Overview of the IEC 61850 communication architecture in smart substations 32

Description of fault diagnosis problems in substations 35

Latency analysis of the digital twin system 37

Supplementary Discussion **39**

Generalization saturation and mitigation of topology overfitting 39

Supplementary References **42**

Supplementary Figures

Supplementary Figure 1: Illustration of the fault diagnosis problem in substations

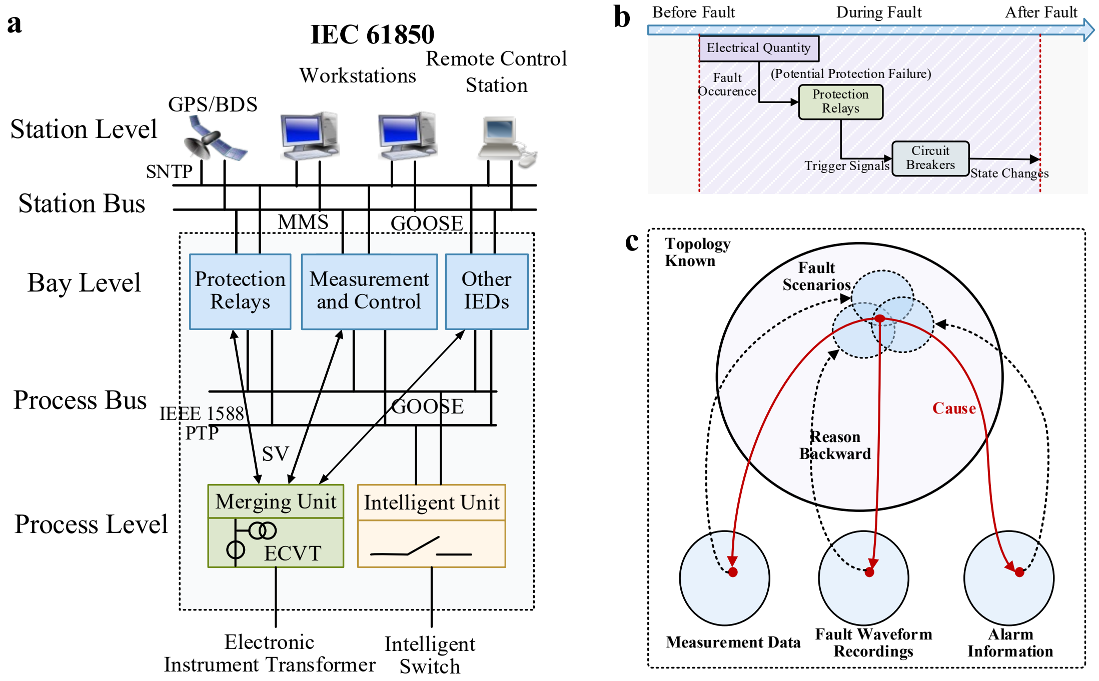

Supplementary Figure 1. **Illustration of the fault diagnosis problem in smart substations.** **a** IEC 61850–based digital communication architecture of a smart substation. The system follows the standard three-level, two-network framework, where the process, bay, and station levels are interconnected via a process bus and a station bus. Process-level merging units and intelligent terminals interface with primary equipment and publish sampled values and switching status to bay-level IEDs. Bay-level protection and control IEDs subscribe to SV streams and exchange fast GOOSE messages for protection and automation. Station-level systems provide supervisory control and data management, communicating with bay-level IEDs through MMS and GOOSE. Time synchronization is sourced from BeiDou/GPS, with SNTP used at the station level and IEEE 1588v2 PTP at the bay and process levels. **b** Schematic of the fault process in a substation. When a primary-system fault occurs, secondary protection devices detect abrupt changes in electrical quantities. Protection relays (PRs) operate according to preset logic and issue trip commands to circuit breakers (CBs); backup protection acts if the main protection fails. During the process, PR and CB actions generate alarms, fault waveform recordings capture transient features, and SCADA data reflects pre- and post-fault steady-state conditions, together depicting the full fault evolution. **c** Schematic of fault scenarios and multi-source data mapping. Integrating multi-source data is essential for accurately determining fault location and type, understanding PR status, and characterizing fault mechanisms and propagation.

Supplementary Figure 2: Example case of the fault enumeration tree traversal method based on breadth-first search

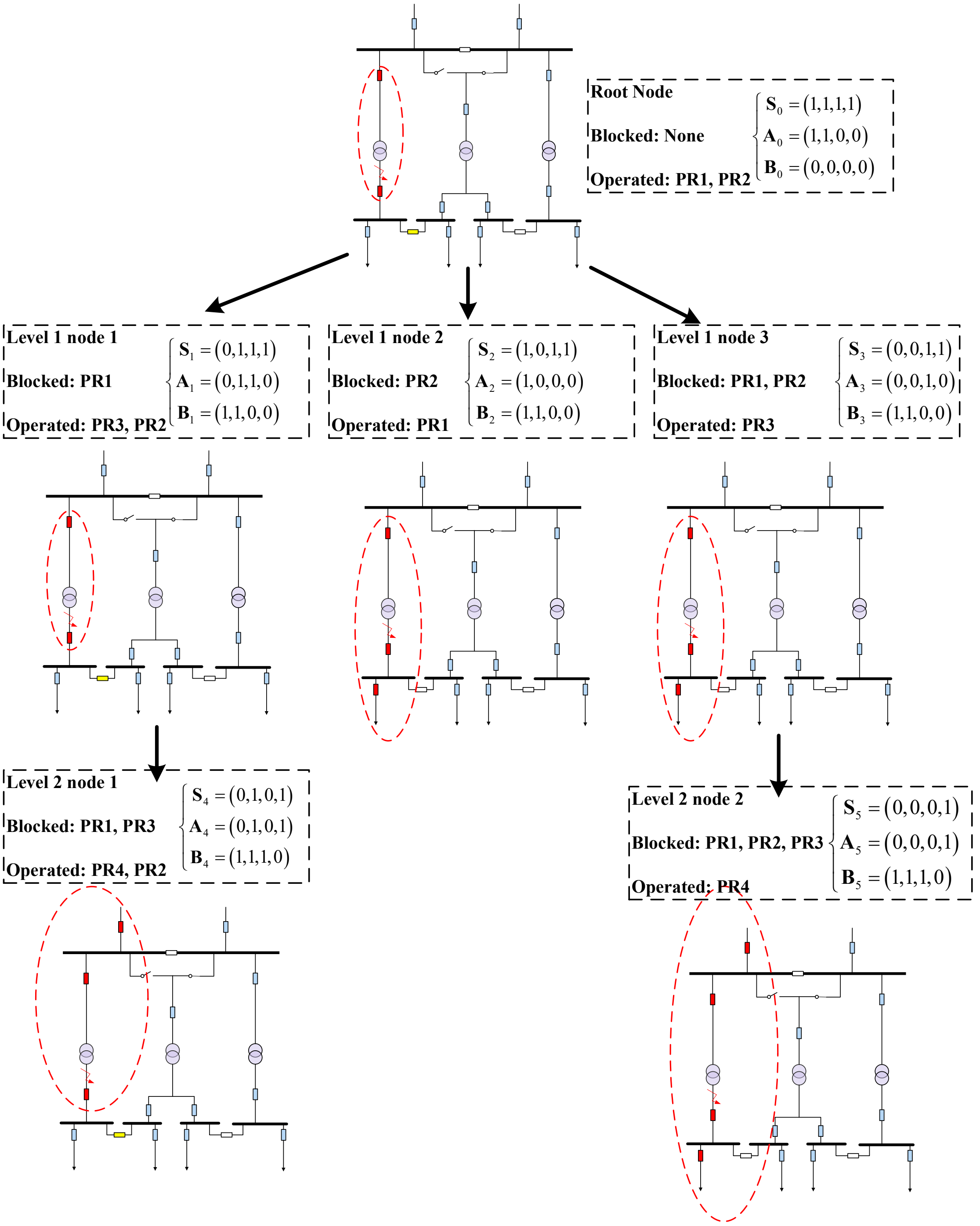

Supplementary Figure 2. Example case of the fault enumeration tree traversal method based on breadth-first search. Illustration of the fault enumeration tree traversal using breadth-first search (BFS). Taking the low-voltage inter-phase fault of #1 main transformer as the root scenario, the corresponding relay operations in the base case-#1 main transformer differential protection (PR1) and 10 kV bus transfer scheme (PR2)—generate three first-level child nodes (PR1 blocked; PR2 blocked; PR1 and PR2 blocked). BFS then traverses each node

to evaluate the resulting fault evolution and newly triggered protections. For example, blocking PR1 leads to #3 main transformer high-voltage overcurrent protection (PR3), which forms the next-layer child node; blocking PR2 yields no new protections; blocking both PR1 and PR2 again triggers PR3. The traversal proceeds layer by layer: in the second level, blocking PR1 and PR3 causes the fault to propagate to the 110 kV system, activating Line I distance protection stage III (PR4) together with PR2, while blocking PR1, PR2, and PR3 also results in PR4 operation. Further enumeration produces no additional relay actions, indicating completion of the BFS-based fault enumeration tree.

Supplementary Figure 3: Test results of models on the fault location task

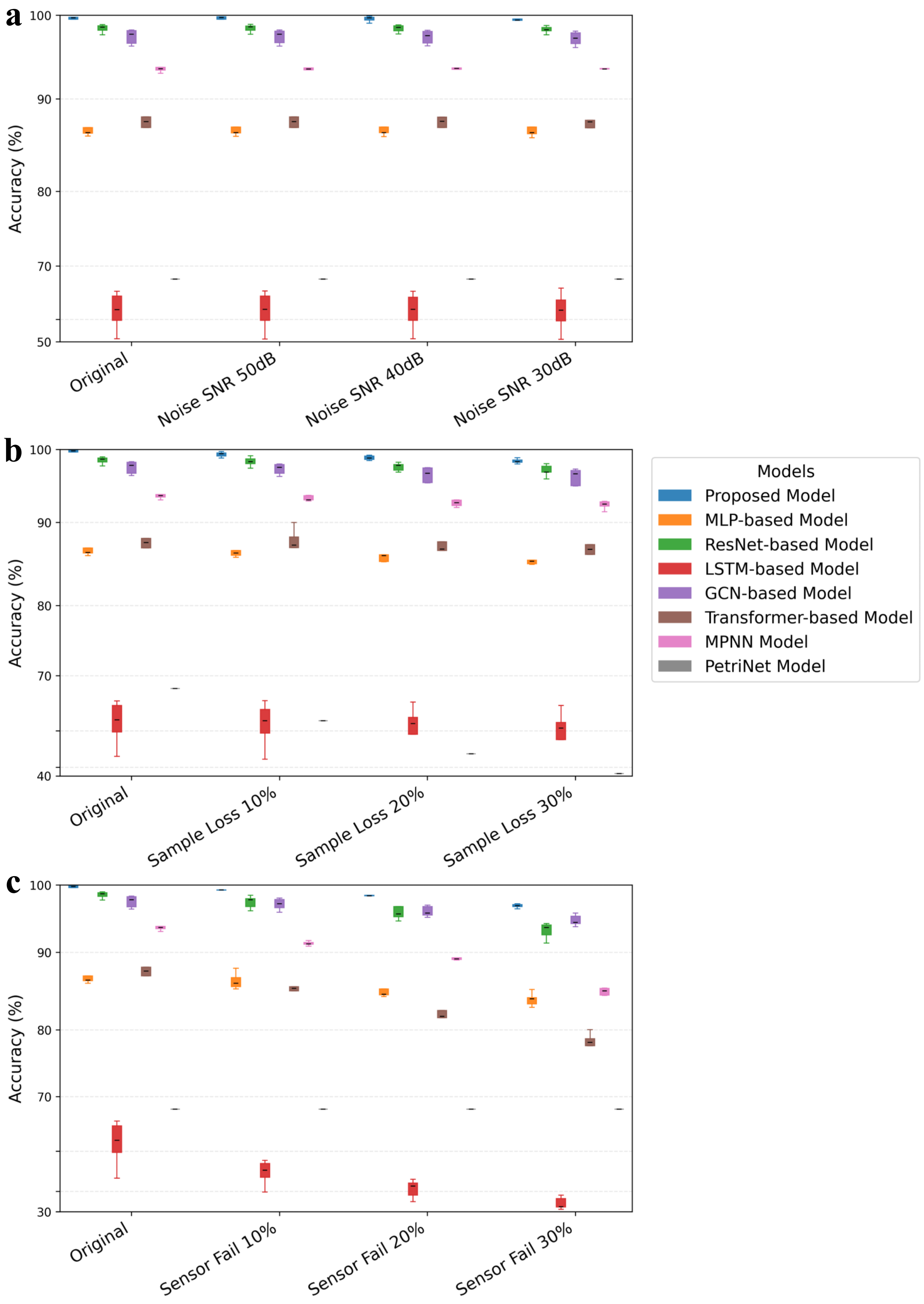

Supplementary Figure 3. Test results of models on the fault location task. Box-and-whisker

plots present the distribution of test accuracy across five independent training runs with different random seeds for eight models: TCFPN, MPNN, MLP-based, ResNet-based, LSTM-based, GCN-based, Transformer-based, and the Proposed model, evaluated on multiple test datasets, including the original dataset, datasets with SNR levels of 30 dB, 40 dB, and 50 dB, sampling-loss rates of 10%, 20%, and 30%, and sensor-failure rates of 10%, 20%, and 30%. For each dataset, the box represents the interquartile range (IQR, 25th–75th percentiles), the central black line indicates the median accuracy, and the whiskers extend to the minimum and maximum values within 1.5×IQR. **a** Fault location test accuracy under different noise levels (SNR). **b** Fault location test accuracy under different sampling-loss rates. **c** Fault location test accuracy under different sensor-failure rates.

Supplementary Figure 4: Test results of models on the fault type classification task

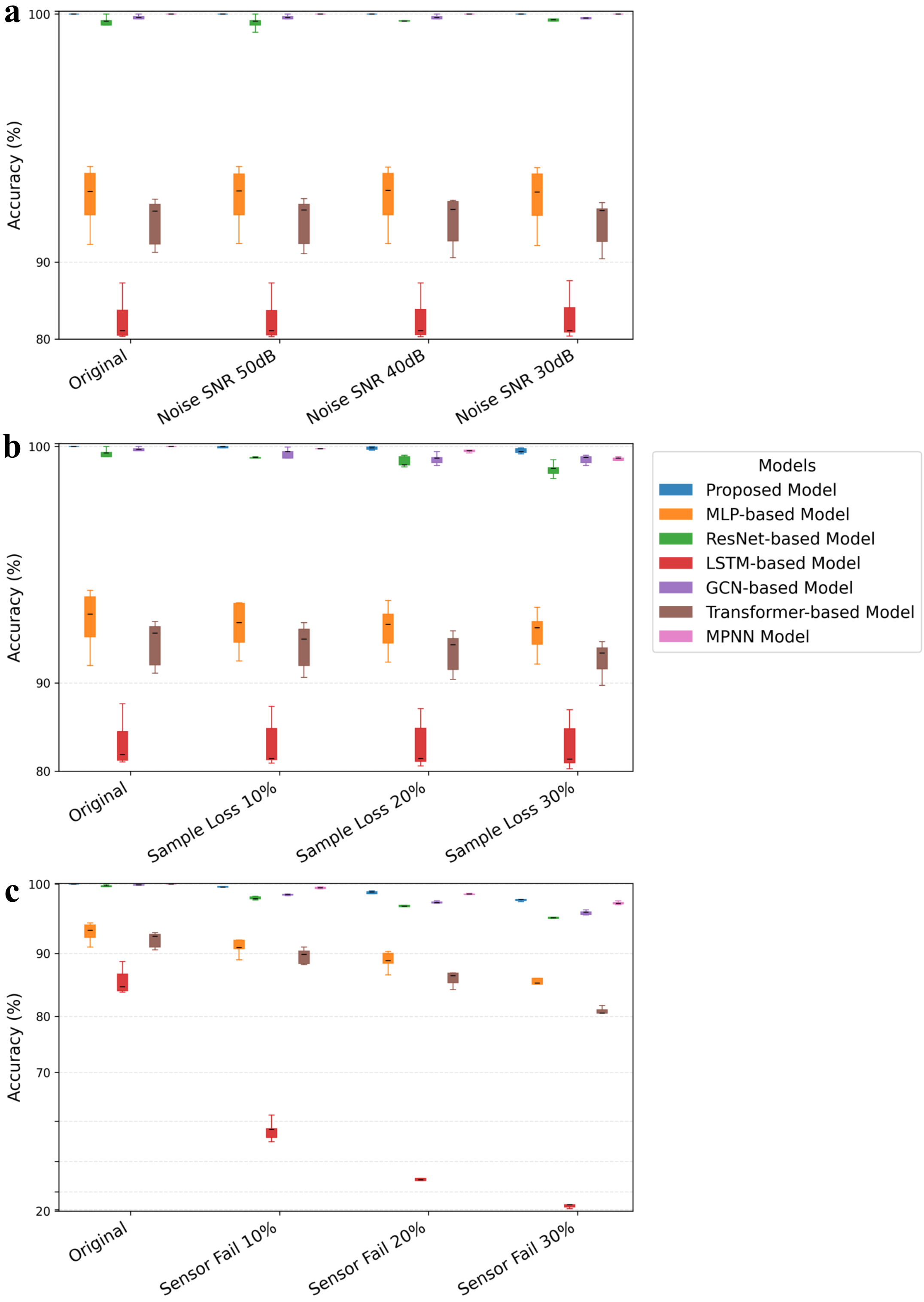

Supplementary Figure 4. Test results of models on the fault type classification task. Box-

and-whisker plots present the distribution of test accuracy across five independent training runs with different random seeds for eight models: TCFPN, MPNN, MLP-based, ResNet-based, LSTM-based, GCN-based, Transformer-based, and the Proposed model, evaluated on multiple test datasets, including the original dataset, datasets with SNR levels of 30 dB, 40 dB, and 50 dB, sampling-loss rates of 10%, 20%, and 30%, and sensor-failure rates of 10%, 20%, and 30%. For each dataset, the box represents the interquartile range (IQR, 25th–75th percentiles), the central black line indicates the median accuracy, and the whiskers extend to the minimum and maximum values within 1.5×IQR. **a** Fault type classification test accuracy under different noise levels (SNR). **b** Fault type classification test accuracy under different sampling-loss rates. **c** Fault type classification test accuracy under different sensor-failure rates.

Supplementary Figure 5: Test results of models on the protection failure detection task

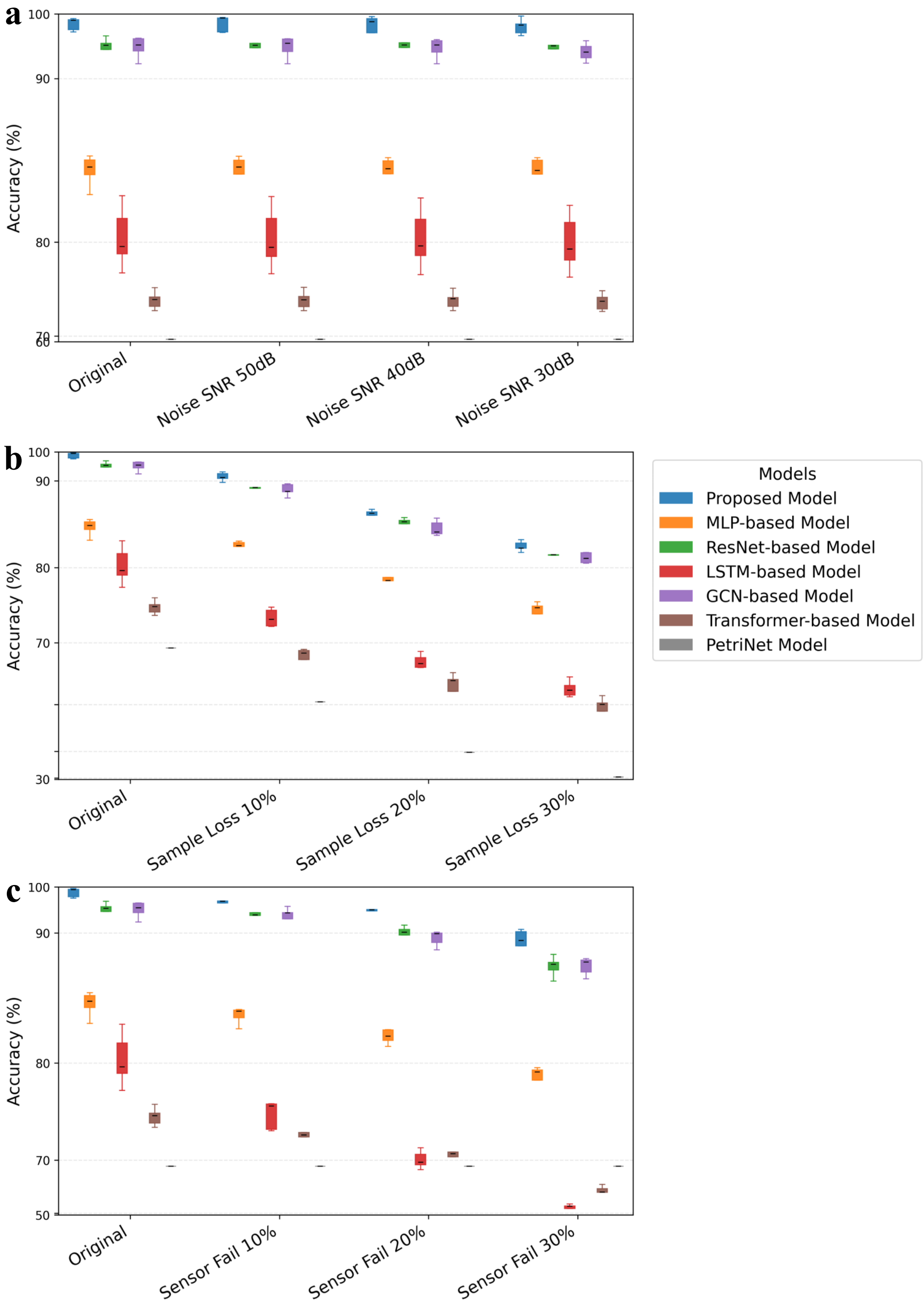

Supplementary Figure 5. Test results of models on the protection failure detection task.

Box-and-whisker plots present the distribution of test accuracy across five independent training runs with different random seeds for eight models: TCFPN, MPNN, MLP-based, ResNet-based, LSTM-based, GCN-based, Transformer-based, and the Proposed model, evaluated on multiple test datasets, including the original dataset, datasets with SNR levels of 30 dB, 40 dB, and 50 dB, sampling-loss rates of 10%, 20%, and 30%, and sensor-failure rates of 10%, 20%, and 30%. For each dataset, the box represents the interquartile range (IQR, 25th–75th percentiles), the central black line indicates the median accuracy, and the whiskers extend to the minimum and maximum values within 1.5×IQR. **a** Protection failure detection test accuracy under different noise levels (SNR). **b** Protection failure detection test accuracy under different sampling-loss rates. **c** Protection failure detection test accuracy under different sensor-failure rates.

Supplementary Figure 6: Ablation study results on the fault location task

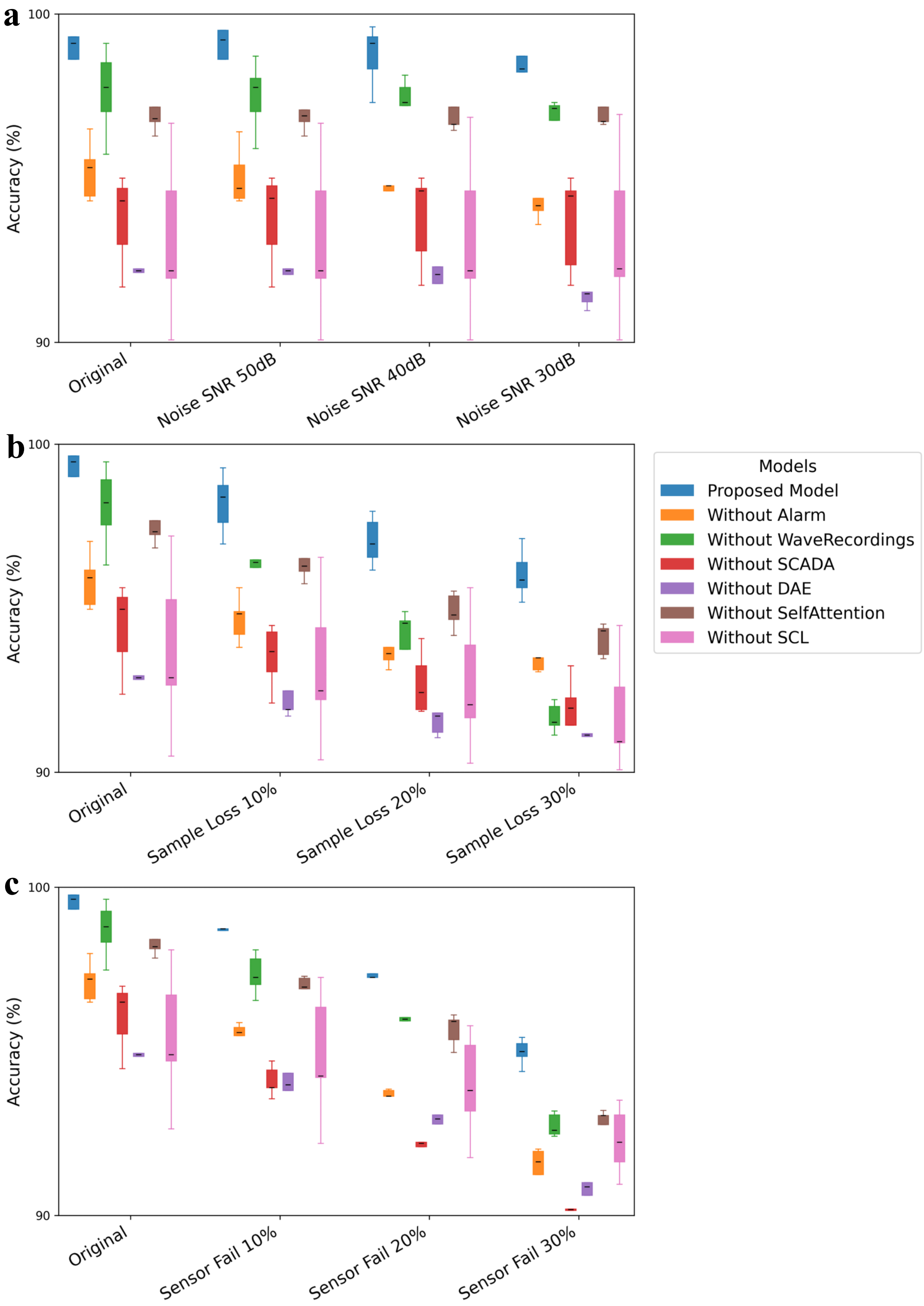

Supplementary Figure 6. Ablation study results on the fault location task. Box-and-

whisker plots present the distribution of test accuracy across five independent training runs with different random seeds for the ablation variants—w/o Alarm, w/o Waveform, w/o SCADA, w/o DAE, w/o Self-Atten, w/o SCL—and the Proposed Model, evaluated on multiple test datasets, including the original dataset, datasets with SNR levels of 30 dB, 40 dB, and 50 dB, sampling-loss rates of 10%, 20%, and 30%, and sensor-failure rates of 10%, 20%, and 30%. Here, w/o Alarm, w/o Waveform, and w/o SCADA denote removing alarm information, fault waveform recordings, and SCADA measurement data from the model input, respectively; w/o DAE removes the denoising autoencoder pre-training module; w/o Self-Atten removes the self-attention layer; and w/o SCL removes supervised contrastive learning during training. For each dataset, the box represents the interquartile range (IQR, 25th–75th percentiles), the central black line indicates the median accuracy, and the whiskers extend to the minimum and maximum values within 1.5×IQR. **a** Fault location test accuracy of ablation variants under different noise levels (SNR). **b** Fault location test accuracy under different sampling-loss rates. **c** Fault location test accuracy under different sensor-failure rates.

Supplementary Figure 7: Ablation study results on the fault type classification task

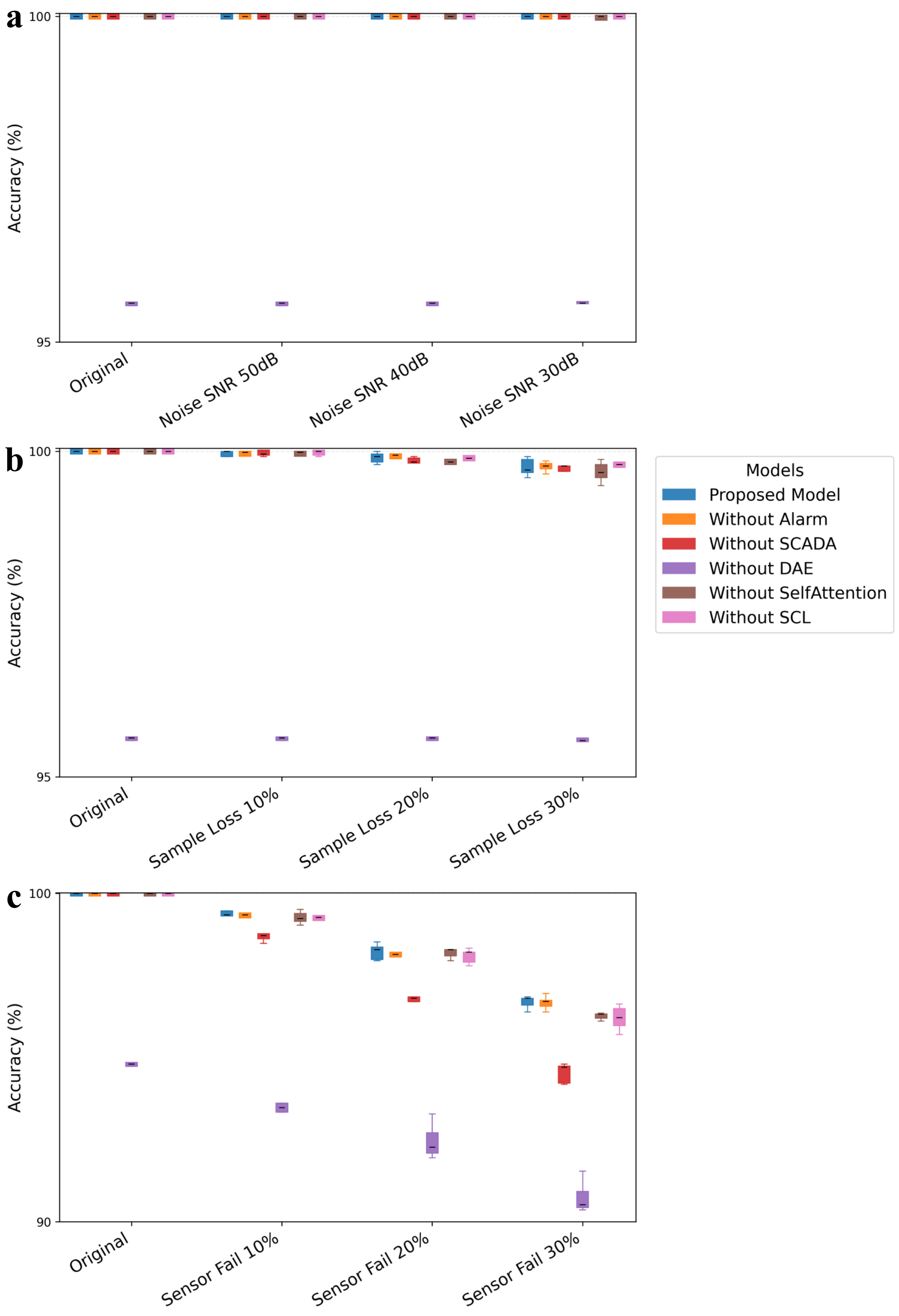

Supplementary Figure 7. Ablation study results on the fault type classification task. Box-and-whisker plots present the distribution of test accuracy across five independent training runs with different random seeds for the ablation variants—w/o Alarm, w/o Waveform, w/o SCADA, w/o DAE, w/o Self-Atten, w/o SCL—and the Proposed Model, evaluated on multiple test datasets, including the original dataset, datasets with SNR levels of 30 dB, 40 dB, and 50 dB, sampling-loss rates of 10%, 20%, and 30%, and sensor-failure rates of 10%, 20%, and 30%. Here, w/o Alarm, w/o Waveform, and w/o SCADA denote removing alarm information, fault waveform recordings, and SCADA measurement data from the model input, respectively; w/o DAE removes the denoising autoencoder pre-training module; w/o Self-Atten removes the self-attention layer; and w/o SCL removes supervised contrastive learning during training. For each dataset, the box represents the interquartile range (IQR, 25th–75th percentiles), the central black line indicates the median accuracy, and the whiskers extend to the minimum and maximum values within 1.5×IQR. **a** Fault type classification test accuracy of ablation variants under different noise levels (SNR). **b** Fault type classification test accuracy under different sampling-loss rates. **c** Fault type classification test accuracy under different sensor-failure rates.

Supplementary Figure 8: Ablation study results on the protection failure detection task

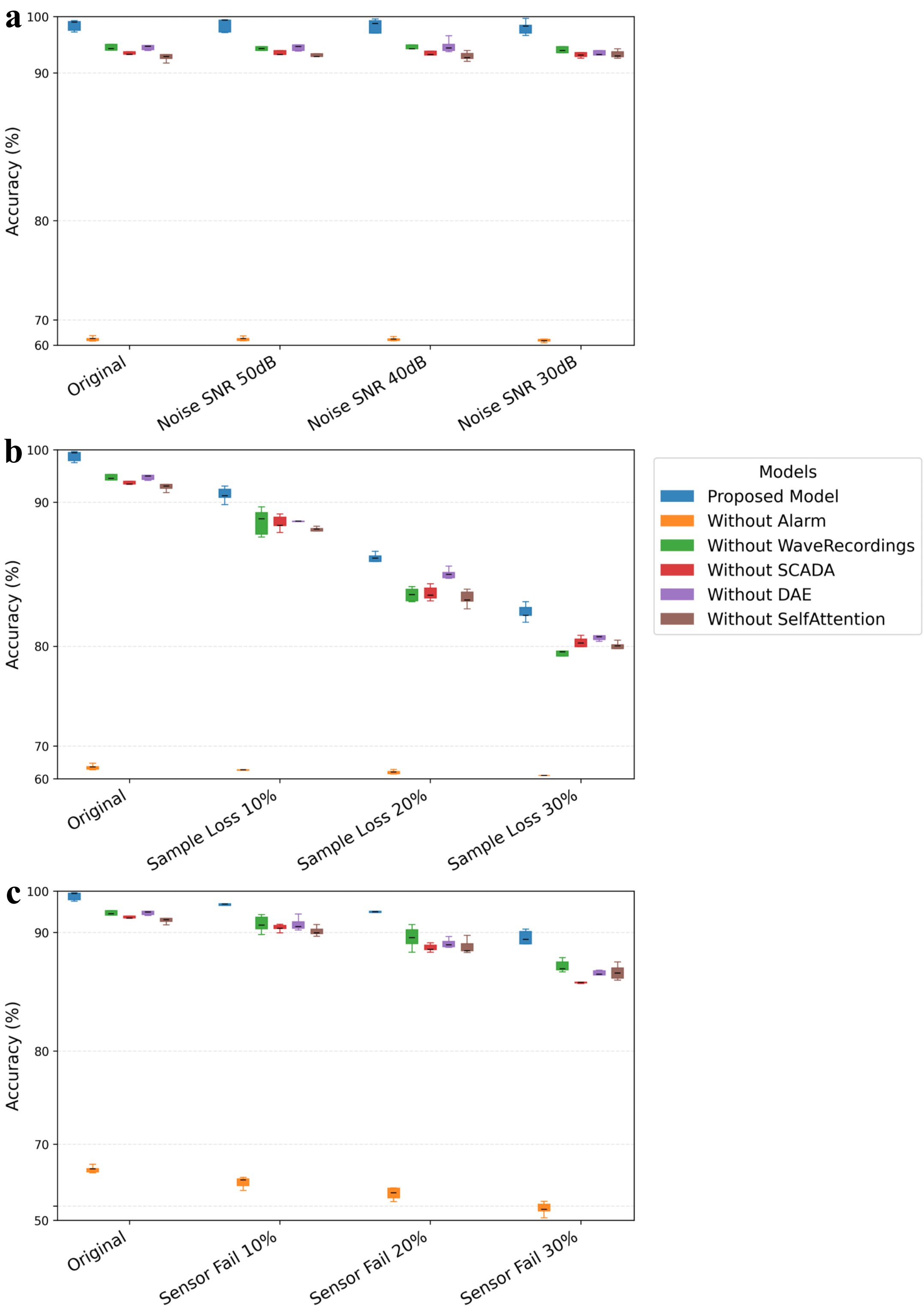

Supplementary Figure 8. Ablation study results on the protection failure detection task.

Box-and-whisker plots present the distribution of test accuracy across five independent training runs with different random seeds for the ablation variants—w/o Alarm, w/o Waveform, w/o SCADA, w/o DAE, w/o Self-Atten, w/o SCL—and the Proposed Model, evaluated on multiple test datasets, including the original dataset, datasets with SNR levels of 30 dB, 40 dB, and 50 dB, sampling-loss rates of 10%, 20%, and 30%, and sensor-failure rates of 10%, 20%, and 30%. Here, w/o Alarm, w/o Waveform, and w/o SCADA denote removing alarm information, fault waveform recordings, and SCADA measurement data from the model input, respectively; w/o DAE removes the denoising autoencoder pre-training module; w/o Self-Atten removes the self-attention layer; and w/o SCL removes supervised contrastive learning during training. For each dataset, the box represents the interquartile range (IQR, 25th–75th percentiles), the central black line indicates the median accuracy, and the whiskers extend to the minimum and maximum values within 1.5×IQR. **a** Protection failure detection test accuracy of ablation variants under different noise levels (SNR). **b** Protection failure detection test accuracy under different sampling-loss rates. **c** Protection failure detection test accuracy under different sensor-failure rates.

Supplementary Figure 9: Comparison of partial real fault waveforms and simulation waveforms.

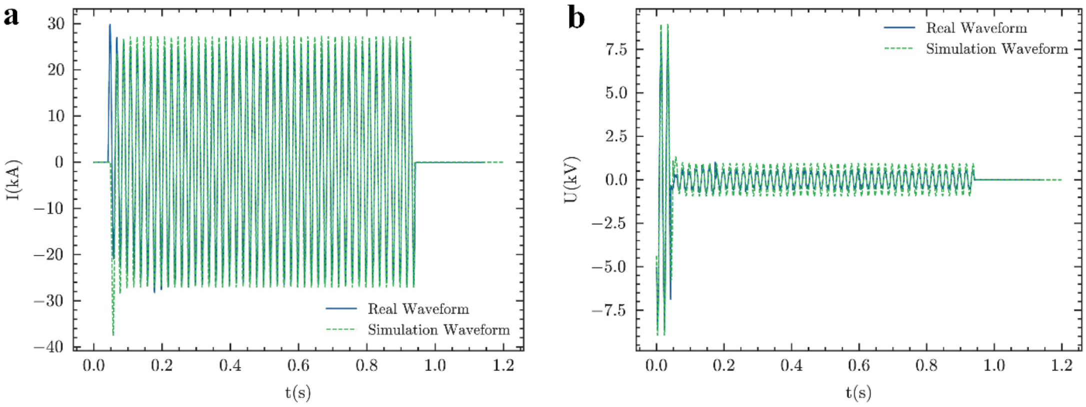

Supplementary Figure 9. Comparison of partial real fault waveforms and simulation waveforms. a Current waveform of phase A on the low-voltage side of the #3 main transformer during a three-phase short circuit occurring at the 10 kV Sectionalizer II. When the fault occurs, the current rises sharply and remains at a high magnitude until 0.9 s, when the low-voltage overcurrent protection operates and trips the low-voltage circuit breaker, causing the current to drop to zero. The green dashed line represents the simulation waveform, and the blue solid line represents the real recorded waveform. **b** Voltage waveform of phase A on the 10 kV Busbar III. Before the fault, the voltage remains within the normal operating range (the rated line voltage is 10 kV, corresponding to a phase-voltage peak value of $10/\sqrt{3}\times\sqrt{2}=8.165\ \text{kV}$). During the fault, the three-phase short circuit causes the voltage to collapse sharply, leaving only residual voltage. After the protection system isolates the fault, the bus voltage drops to zero. The green dashed line represents the simulation waveform, and the blue solid line represents

the real recorded waveform.

Supplementary Figure 10: Physical layout and feasible topology space of the studied 110 kV substation

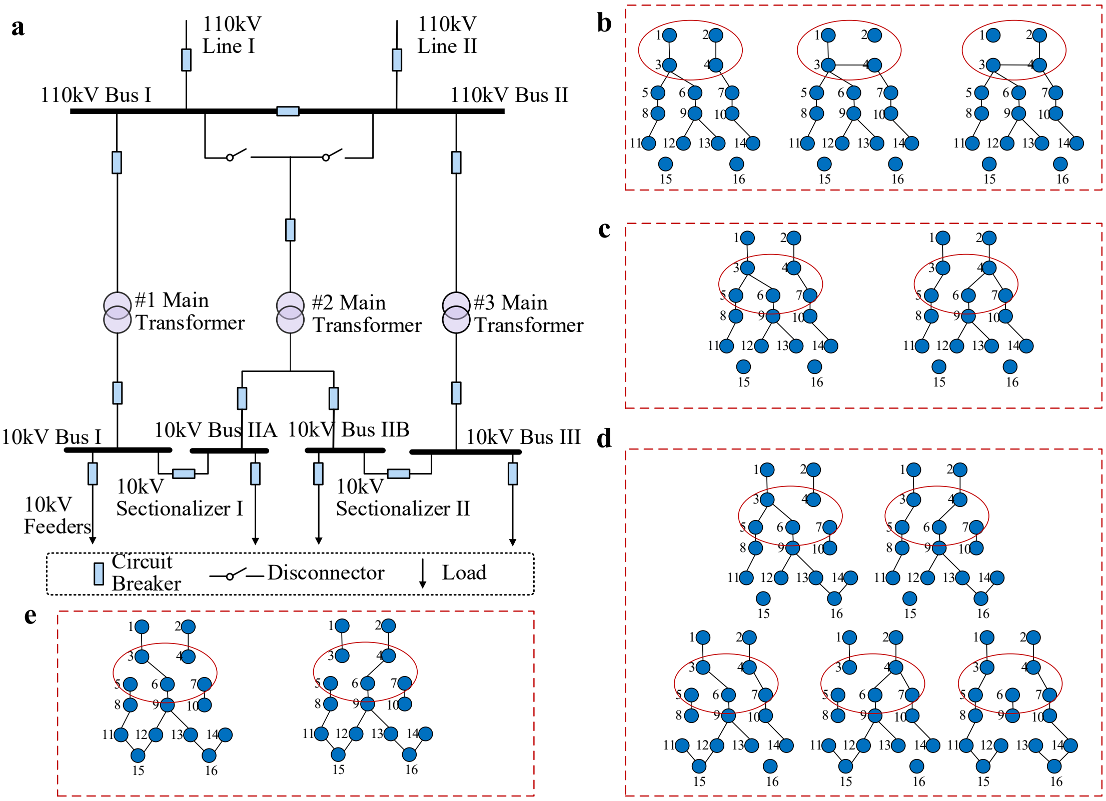

Supplementary Figure 10. Physical Layout and Feasible Topology Space of the Studied 110 kV Substation a Overall layout of the studied 110 kV substation. The system comprises two 110 kV incoming lines, two 110 kV busbars, three main transformers with both high- and low-voltage terminals, four 10 kV busbars, and two 10 kV sectionalizer switches, defining 16 physically meaningful fault locations within the primary network. **b–e** Illustration of all physically feasible topology categories in the substation, determined by permissible combinations of 110 kV busbar configurations and transformer operating modes. **b** Three admissible 110 kV busbar configurations arising from the double-bus scheme: (i) two bus sections operating independently (bus-tie open), (ii) bus-tie closed with Line I energized and Line II on standby, and (iii) bus-tie closed with Line II energized and Line I on standby. **c** Two feasible three-transformer operating modes, where Transformer #2 can be connected either to Bus I or Bus II. **d** Five admissible two-transformer operating modes. Any two of the three transformers may operate; if Transformer #2 is included, it again has two possible wiring options, yielding five distinct configurations. **e** Two feasible single-transformer operating modes. Only Transformer #2 can operate alone, as the single operation of Transformer #1 or #3 cannot supply all four 10 kV bus sections simultaneously. Together, these constrained combinations form a total of 27 physically realistic topologies for the substation.

Supplementary Figure 11: Primary and secondary system modeling of the digital twin platform

a

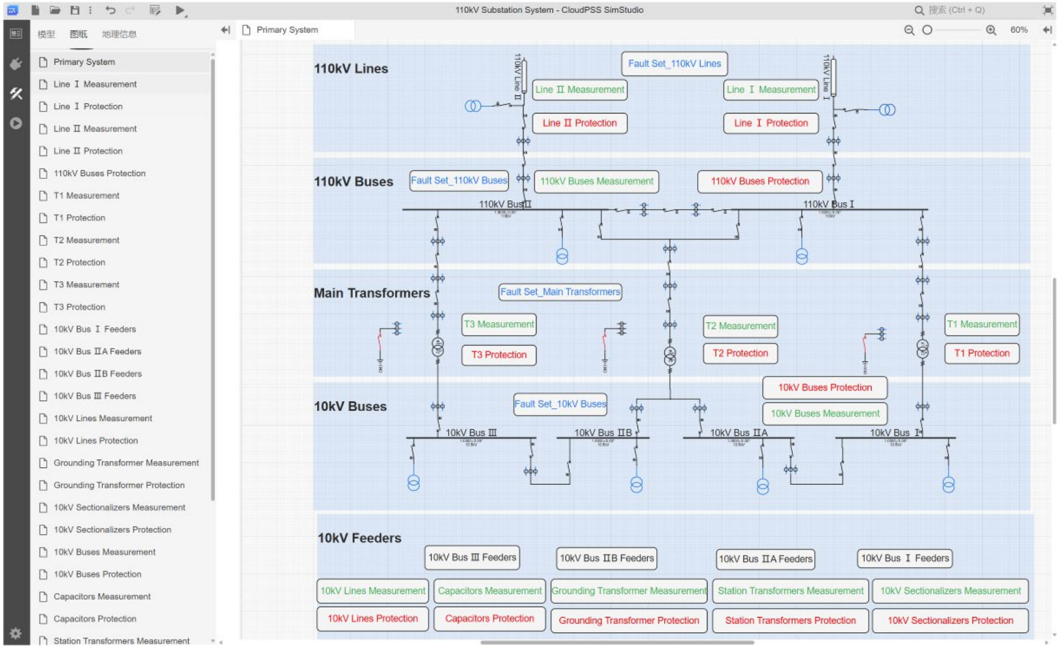

b

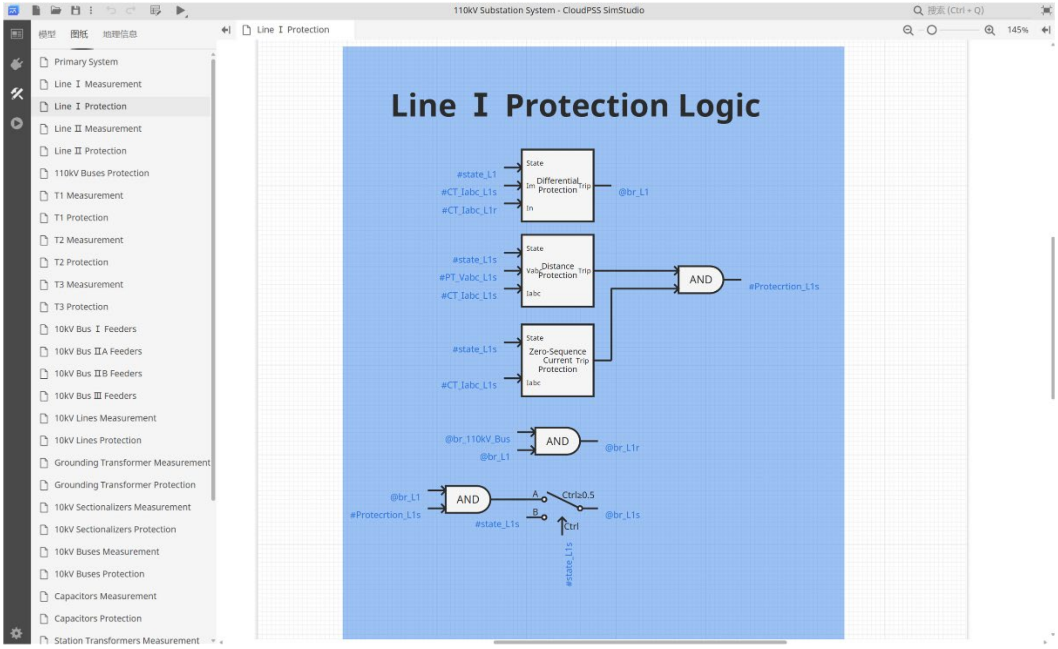

Supplementary Figure 11. Primary and secondary system modeling of the digital twin platform a Primary system model constructed in CloudPSS, reproducing the electrical network configuration of the substation, including power sources, busbars, main transformers, loads, capacitors, grounding transformers, circuit breakers, and instrument transformers (CTs/PTs), to emulate the operational behavior of primary equipment. **b** Secondary protection system model

reflecting the actual relay settings, device configurations, and protection logic deployed in the substation, enabling realistic simulation of interactions among secondary devices.

Supplementary Figure 12: Detailed feature extraction networks for multi-modal inputs.

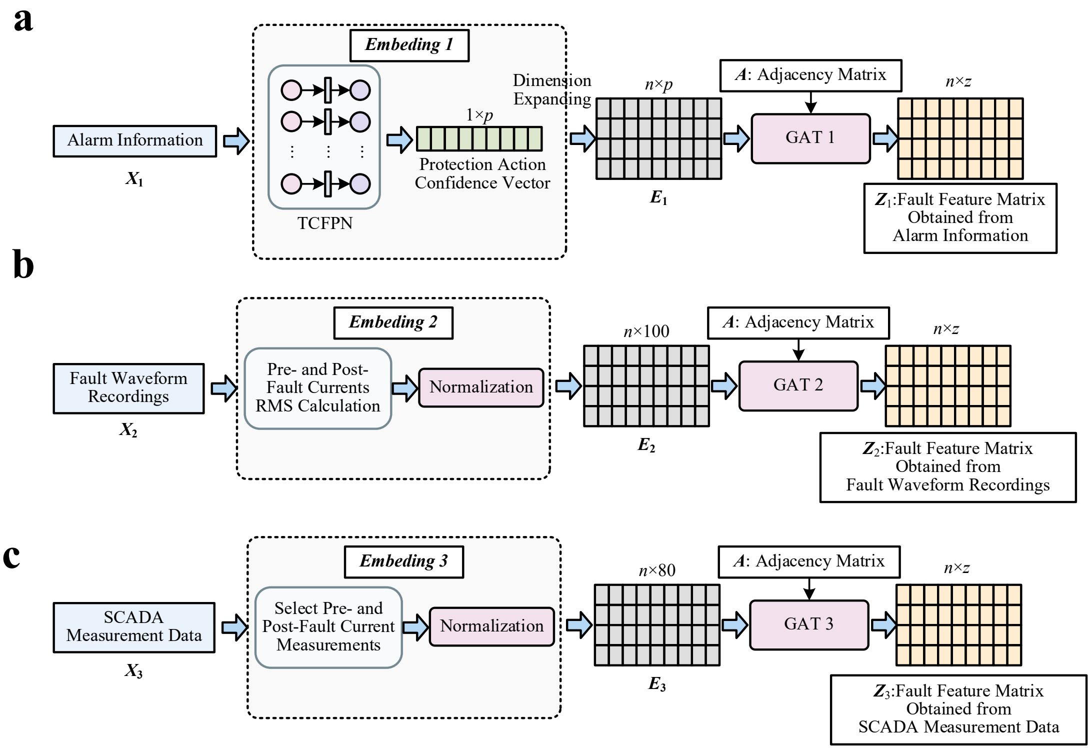

Supplementary Figure 12. Detailed feature extraction networks for multi-modal inputs. a Feature extraction network for alarm information. Alarm data are processed using a TCFPN to obtain relay-action confidence vectors, which are expanded and encoded by GATs to produce alarm feature matrices. **b** Feature extraction network for fault waveform recordings. RMS values of three-phase and zero-sequence currents over pre- and post-fault windows are normalized and passed through GATs to extract waveform-based features. **c** Feature extraction network for SCADA measurements. Normalized current measurements at each primary node, including pre- and post-fault samples, are encoded by GATs to obtain SCADA feature matrices.

Supplementary Tables

Supplementary Table 1: Comparison of existing fault diagnosis methods for power systems

Method	Type	Input Data	Main Task	Accuracy	Robustness	Generalization	Model Construction Cost	Model Update Cost

Expert Systems[1]	Rule-based	Alarm	Fault location	Moderate	Moderate–Low (sensitive to missing alarms)	Limited (rules tied to specific operation conditions)	High (manual rule-based construction)	High (frequent manual updates required)
Bayesian Networks[2]	Rule-based	Alarm	Fault location	Moderate	Moderate (probabilistic reasoning handles uncertain alarms)	Moderate (structure fixed; parameters adaptable)	Medium (requires expert-defined BN structure)	Medium (parameter tuning needed)
Fuzzy Sets[3]	Rule-based	Topology + Alarm	Fault location	Moderate–High	Moderate–High (tolerant to vague or missing alarms)	Moderate (adapts to topology through incidence matrices)	Low–Medium (simple fuzzy matrices)	Medium (rules easy to adjust)
Rough Set[4]	Rule-based	Alarm	Fault location + fault type classification	Moderate–High	Moderate–High (rule reduction enhances stability)	Moderate (rules discovered from data, not topology-dependent)	Medium–Low (automatic rule extraction)	Medium (compact rule-base, low maintenance)
Analytical Models[5]	Rule-based	Alarm	Fault location + protection failure detection	Moderate–High	Moderate–High (robust to redundant alarms; timestamp utilization)	Moderate (supports varied protection schemes & bus configurations)	Medium (analytic formula only)	Medium (only configuration-related adjustments)
Petri Nets[6]	Rule-based	Alarm	Fault location + protection failure detection	Moderate–High	Moderate–High (handles fuzzy evidence)	Moderate (temporal + fuzzy transitions work in many scenarios)	Medium (Petri net modeling required)	Medium (net structure updates moderately costly)
Hierarchical Diagnosis[7]	Rule-based	Alarm + SCADA	Fault location + protection failure detection	High	High (multi-source fusion mitigates conflicting information)	Good (layered use of switching + electrical info)	Medium–High (two-layer diagnostic modeling)	Medium (both layers must be updated)
DS Evidence + BP NN[8]	Data-driven	Fault waveform recordings	Busbar fault type classification	High	High (DS fusion reduces uncertainty before NN training)	Good (learns busbar patterns independent of topology)	Medium (requires DS preprocessing + NN training)	Medium (NN retraining needed for new waveform patterns)
GCNs[9]	Data-driven	Topology + SCADA	Fault location	Very High	Very High (robust to noise, data loss,	Very good (graph learning generalizes across networks)	Medium–High (graph modeling + GNN training)	Medium (moderate retraining for

					topology variations)			topology changes)
CNN-based Transformer[10]	Data-driven	Fault waveform recordings	Fault location + fault type classification	Very High	Very High (attention mechanism resists waveform distortions)	Good (long-term temporal modeling adapts across conditions)	Medium–High (complex hybrid deep model)	Medium (retraining needed for distribution shifts)
R-GNNs[11]	Data-driven	Topology + Fault waveform recordings	Fault location + fault type classification	Very High	Very High (captures spatial–temporal dependencies reliably)	Very good (generalizes across operating modes & topologies)	Medium–High (recurrent GNN architecture)	Medium (requires updates for major topology changes)
MPNN[12]	Data-driven	Topology + Fault waveform recordings	Fault location + fault type classification	Very High	Very High (resilient to topology variations & missing data)	Very good (Graph-Lasso auto-identifies topology)	Medium–High (joint MPNN + topology estimation)	Low–Medium (auto topology update reduces manual work)
Proposed Method	Data-driven	Topology + Alarm + SCADA + Fault waveform recordings	Fault location + fault type classification + protection failure detection	Excellent	Excellent (multi-modal fusion + attention; resistant to noise, missing data)	Excellent (digital twin + graph attention adapt to topology, data quality, and real scenarios)	High (digital twin modeling + multi-modal architecture)	Low–Medium (digital twin reduces update workload)

Supplementary Table 2: Brief protection configuration of a 110kV substation in Shenzhen

Device	Protection type	Time delay(s)
110kV transmission lines	Longitudinal differential protection	0
110kV transmission lines	Distance protection stage II	0.3
110kV transmission lines	Distance protection stage III	1.8
110kV transmission lines	Zero-sequence overcurrent protection stage II	0.3
110kV transmission lines	Zero-sequence overcurrent protection stage III	0.6
110kV transmission lines	Zero-sequence overcurrent protection stage IV	1.8
110kV busbars	Differential protection	0
110kV busbars	Automatic Bus Transfer	0.3
Main transformers	Longitudinal differential protection	0
Main transformers	High-voltage side overcurrent protection	1.5

Main transformers	High-voltage side zero-sequence protection stage I time limits 1	1.5
Main transformers	High-voltage side zero-sequence protection stage I time limits 2	1.8
Main transformers	High-voltage side zero-sequence protection stage II time limits 1	2.1
Main transformers	High-voltage side zero-sequence protection stage II time limits 2	2.4
Main transformers	High-voltage side gap overcurrent protection	1.5
Main transformers	High-voltage side zero-sequence overvoltage protection	1.5
Main transformers	Low-voltage side overcurrent protection stage I time limit 1	0.9
Main transformers	Low-voltage side overcurrent protection stage I time limit 2	1.2
Main transformers	Low-voltage side overcurrent protection stage II time limit 1	0.9
Main transformers	Low-voltage side overcurrent protection stage II time limit 2	1.2
Main transformers	Low-voltage side overcurrent protection stage II time limit 3	1.5
10kV sectionalizer	Overcurrent protection stage I	0.6
10kV sectionalizer	Overcurrent protection stage II	0.9
10kV sectionalizer	10kV automatic bus transfer	0.3
Grounding transformers	Zero-sequence overcurrent protection stage I	2.3
Grounding transformers	Zero-sequence overcurrent protection stage II	2.6

Supplementary Table 3: Summary of the dataset

Item	Description
Topologies	10 for training and 4 for testing (different operating configurations)
Fault locations	16 (transmission lines, busbars, transformers, sectionalizers)
Fault types	10 (A/B/C single-phase grounding; AB/BC/CA two-phase short-circuit; AB/BC/CA two-phase-to-ground short-circuit; three-phase short-circuit)
Fault resistance	Randomly sampled within 0–10 Ω to represent low-resistance faults
Protection failure conditions	Automatically enumerated using a Breadth-First Search–based fault enumeration method (Supplementary Method)
Base fault scenarios	11,597 for training; 3,890 for testing
Data modalities	Alarm events, SCADA measurements (2 Hz, RMS values of three-phase and zero-sequence currents from CTs), and fault waveform recordings (4 kHz, instantaneous values of the same current quantities)
Noise levels (SNR)	30, 40, and 50 dB (zero-mean Gaussian noise)
Sampling loss	Random removal of 10%, 20%, or 30% of time-series data points from alarm, SCADA, waveform data
Sensor failures	Random dropping of 10%, 20%, or 30% of the entire SCADA or waveform channels
Total fault samples	115,970 for training; 38,900 for testing

Supplementary Table 4: Performance of the proposed model in test dataset with different training batch sizes

Batch size	Fault location accuracy	Fault type classification accuracy	Protection failure detection accuracy
8	0.9621±0.0051	0.9956±0.0003	0.9015±0.0046

16	0.9678±0.0015	0.9960±0.0003	0.9000±0.0039
32	**0.9703±0.0020**	**0.9960±0.0003**	**0.9035±0.0031**
64	0.9662±0.0014	0.9946±0.0001	0.9003±0.0041

Supplementary Table 5: Hyperparameters of the proposed model

**Feature Extraction GAT 1/2/3**	
**Item**	**Parameter**
Layers	3
Node num	16
Input node dim	143/100/80
Hidden layer 1 node dim	256
Hidden layer 2 node dim	256
Output node dim	256
Attention Heads	4 heads per layer
Learnable parameters (Millions)	0.68/0.63/0.61
**DAE Decoder GAT 4**	
**Item**	**Parameter**
Layers	3
Node num	16
Input node dim	3×256=768
Hidden layer 1 node dim	512
Hidden layer 2 node dim	512
Output node dim	143+100+80=323
Attention Heads	4 heads per layer
Learnable parameters (Millions)	3.3
**Self-attention Layer 1/2/3**	
**Item**	**Parameter**
Sequence Length (Input Modality)	3
Input Dim	16×256 = 4096
Normalization Method	Layer Norm
Attention Heads	16
Output Dim	1024
Learnable parameters (Millions)	12.59
**Classifier MLP 1/2/3**	
**Item**	**Parameter**
Layers	2
Input Dim	3×1024
Hidden Dim	1024
Output Dim	16/10/143
Learnable parameters (Millions)	3.16/3.16/3.22

Supplementary Table 6: Architecture and parameter counts of all baseline models

Model	Encoder architecture	Encoder learnable parameters (Millions)	Decoder architecture	Decoder learnable parameters (Millions)	Self-attention layer learnable parameters (Millions)	MLP Classifiers learnable parameters (Millions)	Total learnable parameters (Millions)
MPNN	2-layer Conv1d + MLP + 4-layer MPNN	59.89	N/A	N/A	N/A	0.85	60.74
MLP-based	3-branch × 3-layer MLP	2.3	3-layer MLP	3.54	12.59× 3	3.22× 3	53.15
ResNet-based	3-branch × 3-layer Conv2d ResNet	2.22	3-layer ConvTranspose2d ResNet	3.68	12.59× 3	3.22× 3	53.21
LSTM-based	3-branch × 3-layer LSTM	2.05	3-layer LSTM	3.41	12.59× 3	3.22× 3	52.77
GCN-based	3-branch × 3-layer GCN	1.94	3-layer GCN	3.55	12.59× 3	3.22× 3	52.60
Transformer-based	3-branch × 3-layer Transformer Encoder	2.5	3-layer Transformer Decoder	3.59	12.59× 3	3.22× 3	53.4
Proposed	3-branch × 3-layer GAT	1.92	3-layer GAT	3.3	12.59× 3	3.22× 3	52.53

Supplementary Table 7: Training time, inference time and GPU memory usage of each module across all baseline models

Model	DAE Training Time (s/epoch)	DAE GPU Mem (MB)	SCL Training Time (s/epoch)	SCL GPU Mem (MB)	Classifier Training Time (s/epoch)	Classifier GPU Mem (MB)	Total Training Time (hours)	Inference Time (ms/sample)
MPNN (end-to-end training)	N/A	N/A	N/A	N/A	75.3	2183.9	5.23	622
MLP-based	13.62	178.5	10.01	311.4	27.92	198.0	2.87	155
ResNet-based	91.13	6153.4	37.01	1689.6	69.52	1594.9	9.41	337

LSTM-based	18.79	1943.4	11.62	781.7	28.42	684.8	3.14	157
GCN-based	89.68	438.8	39.84	361.9	88.31	262.7	10.84	563
Transformer-based	50.17	5540.8	21.29	627.8	41.30	531.1	5.44	181
Proposed	93.33	1132.0	41.4	383.7	100.90	286.6	11.90	560

Supplementary Table 8: Diagnostic accuracy of the proposed model under increasing sampling-loss and sensor-failure conditions

Test dataset	Fault location accuracy (%)	Fault type classification accuracy (%)	Protection failure detection accuracy (%)
Sampling loss 10%	97.35	100	88.15
Sampling loss 20%	96.79	99.97	84.01
Sampling loss 30%	96.32	99.92	80.62
Sampling loss 40%	93.19	98.33	69.43
Sampling loss 50%	92.16	98.41	60.62
Sampling loss 60%	90.72	98.53	50.64
Sampling loss 70%	86.38	98.66	40.13
Sampling loss 80%	80.23	97.63	26.12
Sampling loss 90%	63.21	95.4	13.57
Sensors fail 10%	97.25	99.64	90.26
Sensors fail 20%	96.61	98.87	89.2
Sensors fail 30%	95.48	97.63	87.17
Sensors fail 40%	89.33	91.18	78.61
Sensors fail 50%	84.68	86.38	73.52
Sensors fail 60%	78.17	78.33	65.14
Sensors fail 70%	70.72	68.64	58.51
Sensors fail 80%	59.43	49.23	45.14
Sensors fail 90%	46.97	29.61	33.93

Supplementary Table 9: Alarm information of the real fault case

Time	Device	Alarm
23:24:51.842	#2 Transformer protection A	Low-voltage side branch 2 overcurrent stage II time limit 1
23:24:52.748	#2 Transformer protection A	Trip low-voltage side branch 2 sectionalizer
23:24:52.748	#2 Transformer protection A	Block low-voltage side branch 2 automatic bus transfer
23:24:52.748	#2 Transformer protection B	Low-voltage side branch 2 overcurrent stage II time limit 1
23:24:52.748	#3 Transformer protection B	Low-voltage side branch 1 overcurrent stage I time limit 1
23:24:52.748	#3 Transformer protection B	Low-voltage side branch 1 overcurrent stage II time limit 1
23:24:52.749	#3 Transformer protection A	Low-voltage side branch 1 overcurrent stage II time limit 1
23:24:52.749	#3 Transformer protection A	Trip low-voltage side branch 1 sectionalizer

23:24:52.749	#3 Transformer protection A	Block low-voltage side branch 1 automatic bus transfer
23:24:52.750	#3 Transformer protection A	Low-voltage side branch 1 overcurrent stage II time limit 1
23:24:52.751	#3 Transformer protection A	Trip low-voltage side branch 1
23:24:52.751	#3 Transformer protection A	Low-voltage side branch 1 overcurrent stage I time limit 1
23:24:52.752	#2 Transformer protection B	Low-voltage side branch 2 overcurrent stage I time limit 1
23:24:52.754	#2 Transformer protection A	Trip low-voltage side branch 2
23:24:52.760	#2 Transformer protection A	Low-voltage side branch 2 overcurrent stage I time limit 1

Supplementary Methods

Fault enumeration tree traversal method based on breadth-first search

For a system with *p* PRs, each node *k* in the fault enumeration tree for a particular fault location and fault type is defined by the following three vectors:

$$\begin{cases} \mathbf{S}_k = \left(s_k^1, s_k^2, \cdots, s_k^p\right) \\ \mathbf{A}_k = \left(a_k^1, a_k^2, \cdots, a_k^p\right) \\ \mathbf{B}_k = \left(b_k^1, b_k^2, \cdots, b_k^p\right) \end{cases} \quad (1)$$

In Supplementary Equation (1), $\mathbf{S}_k$ is the PRs state vector for node *k*, with $s_k^i = 0$ indicating the *i*-th relay is blocked and $s_k^i = 1$ indicating it is in normal operation. $\mathbf{A}_k$ is the protection action vector for node *k*, with $a_k^i = 0$ meaning the *i*-th relay did not operate and $a_k^i = 1$ meaning it did. $\mathbf{B}_k$ specifies which relay states must match between node *k* and its child nodes. If $b_k^i = 1$, the *i*-th relay state for all child nodes must be the same as node *k*; otherwise, they may differ. Specifically, if node *q* is a child node of node *k*, the following condition must be satisfied:

$$s_q^i = \begin{cases} s_k^i & \text{if } b_k^i = 1 \\ 0 \text{ or } 1 & \text{if } b_k^i = 0 \end{cases} \quad (2)$$

Based on the above definitions, the steps for the fault enumeration tree traversal method based on BFS are as detailed as follows:

Algorithm 1 Fault enumeration tree traversal method based on BFS

1: **Initialize root node:**

$\mathbf{S}_0 \leftarrow (1,1,\cdots,1)$

$\mathbf{A}_0 \leftarrow$ simulation result of $\mathbf{S}_0$

$\mathbf{B}_0 \leftarrow (0,0,\cdots,0)$

while new child nodes are generated **do**

  for each node k at the bottom level **do**

    $z \leftarrow \sum_{i=1}^{p} a_k^i$

    $M \leftarrow \sum_{i=1}^{z} C_z^i$,where C_z^i denotes the binomial coefficient ("z choose i").

    $\{\mathbf{S}_{k+1},\mathbf{S}_{k+2},\cdots,\mathbf{S}_{k+r}\} \leftarrow$ eliminate state vectors not satisfy Supplementary Equation (2) from M

    $\{\mathbf{A}_{k+1},\mathbf{A}_{k+2},\cdots,\mathbf{A}_{k+r}\} \leftarrow$ simulation results of $\{\mathbf{S}_{k+1},\cdots,\mathbf{S}_{k+r}\}$

    Compute $\{\mathbf{B}_{k+1},\cdots,\mathbf{B}_{k+r}\}$:

    for $q = k+1, k+2, \cdots, k+r$ **do**

      for $i = 1,2,\cdots,p$ **do**

        $b_q^i \leftarrow b_k^i \mid a_k^i$, where | represents the logical OR operation.

      end for

    end for

  end for

end while

Data cleaning: remove samples with more blocked PRs but same simulation results

---

An illustrative example of the proposed fault enumeration tree traversal method is built on the studied substation, whose main protection configuration is summarized in Supplementary Table 2. A phase-to-phase short-circuit fault occurring on the low-voltage side of #1 main transformer is selected to demonstrate the fault enumeration tree traversal method based on BFS. Under the typical operating topology in Supplementary Fig. 2, a phase-to-phase short circuit is applied to the low-voltage side of #1 main transformer in the simulation model, with all protection devices in service. In the base case, the simulation results show that the longitudinal differential protection of #1 main transformer (denoted as PR1) operates first and trips both circuit breakers on its high-voltage and low-voltage sides. After a short delay, the 10 kV automatic transfer

scheme (denoted as PR2) operates, closes the 10 kV sectionalizer II, and restores the supply to the 10 kV Bus III. This fault scenario, with all relays available, is taken as the root node of the fault enumeration tree. Since two protection devices (PR1 and PR2) operate in this scenario, the root node generates three first-level child nodes, corresponding to: (i) PR1 blocked, PR2 available; (ii) PR2 blocked, PR1 available; and (iii) both PR1 and PR2 blocked.

Next, all first-level nodes are traversed in a breadth-first manner, and for each node a time-domain simulation is repeated under the same fault and topology, but with the specified relays blocked. For the first child node (PR1 blocked, PR2 available), the simulation shows that, after the fault occurs, the high-voltage side overcurrent protection of #3 main transformer (denoted as PR3) operates, tripping its associated circuit breakers. After a delay, PR2 still operates and closes 10 kV sectionalizer II, thereby restoring the supply to 10 kV Bus III. Because a new protection element (PR3) is involved in this scenario, a new child node is generated from this node by additionally blocking PR3 (i.e., PR1 and PR3 blocked, PR2 available).

For the second child node (PR2 blocked, PR1 available), the simulation results indicate that PR1 operates as in the base case, but PR2 does not. Consequently, 10 kV Bus III is not resupplied after the fault is cleared. No additional protections operate beyond PR1, so this node does not generate any further child nodes.

For the third child node (PR1 and PR2 both blocked), the simulation shows that PR3 operates after the fault, while PR2 remains blocked and 10 kV Bus III is not restored. Since a new relay (PR3) operates in this scenario, a new child node is again generated by additionally blocking PR3 (i.e., PR1, PR2, and PR3 all blocked).

After traversing all first-level nodes and analyzing their corresponding fault scenarios, the newly generated second-level nodes are appended to the BFS queue and evaluated in the same manner. For the first second-level node (PR1 and PR3 blocked, PR2 available), the simulation shows that the fault propagates to the 110 kV level. The distance protection stage III of 110 kV transmission line I (denoted as PR4) operates, tripping the line, and PR2 subsequently operates to close 10 kV sectionalizer II and restore the supply to 10 kV Bus III. For the second second-level node (PR1, PR2, and PR3 all blocked), the fault likewise propagates to the 110 kV system and PR4 operates, indicating a similar escalation of the fault to the transmission line.

From these second-level nodes, additional child nodes can in principle be generated by further blocking PR4 (e.g., PR1–PR4 all blocked). However, the subsequent simulations for these deeper nodes show no new protection operations, and the fault continues without any further relay response. As no additional relays are triggered and no new child nodes are created, the BFS traversal of the fault enumeration tree terminates at this point.

This example illustrates how the proposed BFS-based fault enumeration tree systematically explores all feasible combinations of relay failures (blocking scenarios) for a given fault and topology. Starting from the fully functional case as the root node, each layer represents an incremental blocking of protections observed in the parent scenarios, while the breadth-first traversal ensures that lower-order failure combinations (with fewer blocked relays) are examined first. The resulting set of scenarios provides a structured and comprehensive basis for analyzing fault propagation paths and protection malfunction behaviors in complex substation systems.

Quantitative clustering metrics for multi-class feature embeddings

Let the dataset contain K classes $\{C_1, C_2, \ldots, C_K\}$. For any sample $x_i \in C_k$, its intra-class distance is defined as

$$a(i) = \frac{1}{|C_k| - 1} \sum_{\substack{x_j \in C_k \\ j \neq i}} \|x_i - x_j\| \tag{3}$$

In Equation (3), $|C_k|$ denotes the number of samples in class C_k and $a(i)$ is the average distance between x_i and all other samples belonging to the same class. The inter-class distance for x_i is computed as

$$b(i) = \min_{m \neq k} \left(\frac{1}{|C_m|} \sum_{x_j \in C_m} \|x_i - x_j\| \right) \tag{4}$$

In Equation (4), the term inside the parentheses is the mean distance from x_i to class C_m , and $b(i)$ selects the smallest such value, representing the distance to the nearest different class. Based on these terms, the silhouette value of sample x_i is

$$s(i) = \frac{b(i) - a(i)}{\max\left(a(i), b(i)\right)} \tag{5}$$

In Equation (5), $s(i)$ measures how well sample x_i is clustered. The overall silhouette score is then calculated as

$$S = \frac{1}{N} \sum_{i=1}^{N} s(i) \tag{6}$$

In Equation (6), N denotes the total number of samples.

For each class C_k , the intra-class distance is computed as

$$\mathrm{Intra}\left(C_k\right)=\frac{1}{\left|C_k\right|\left(C_k-1\right)}\sum_{\substack{x_i,x_j\in C_k\\ i\neq j}}\left\|x_i-x_j\right\| \tag{7}$$

In Equation (7), Intra(C_k) represents the average pairwise distance among all samples within class C_k. The global intra-class distance across all classes is obtained by

$$\mathrm{Intra}=\frac{1}{K}\sum_{k=1}^{K}\mathrm{Intra}\left(C_k\right) \tag{8}$$

In Equation (8), Intra is the mean compactness of all classes.

Similarly, the inter-class distance between any two classes C_a and C_b is defined as

$$\mathrm{Inter}\left(C_a,C_b\right)=\frac{1}{\left|C_a\right|\left|C_b\right|}\sum_{x_i\in C_a}\sum_{x_j\in C_b}\left\|x_i-x_j\right\| \tag{9}$$

In Equation (9), Inter(C_a, C_b) denotes the average pairwise distance between samples from different classes. The global inter-class distance is computed by

$$\mathrm{Inter}=\frac{2}{K\left(K-1\right)}\sum_{a<b}\mathrm{Inter}\left(C_a,C_b\right) \tag{10}$$

In Equation (10), the summation term averages over all unordered class pairs. Finally, the inter/intra separation ratio is calculated as

$$R=\frac{\mathrm{Inter}}{\mathrm{Intra}} \tag{11}$$

In Equation (11), a larger value of R indicates better class separability in the feature space.

Supplementary Notes

Overview of the IEC 61850 communication architecture in smart substations

Modern smart substations built upon the IEC 61850[13] standard adopt a unified, hierarchical, and networked automation architecture that integrates primary equipment, intelligent electronic devices (IEDs), and supervisory systems through interoperable communication services and

deterministic Ethernet networks.

The system follows the conventional three-level, two-network framework, including process, bay and station levels interconnected by a process bus and a station bus (Supplementary Fig. 1a). At the process level, merging units and intelligent terminals interface directly with primary apparatus such as current and voltage transformers, circuit breakers, and disconnectors, acquiring analog electrical quantities and equipment status and converting them into digital data. The bay level hosts protection, control, and interlocking IEDs, which subscribe to real-time measurement streams from the process level and exchange fast event and command messages to perform protection and automation. The station level provides global supervision, human–machine interfaces, data management, fault recording, and communicates with upper-layer control centers; it also hosts high-level applications such as digital-twin models and advanced analytics.

Information exchange across these three levels relies on the three principal IEC 61850 service models: SV (Sampled Values), GOOSE (Generic Object Oriented Substation Event), and MMS (Manufacturing Message Specification). SV is used at the process level to transmit digitized current and voltage measurements from merging units to bay-level protection and control IEDs. In typical 50 Hz systems, SV streams operate at 80 samples per cycle (4 kHz) for protection-oriented applications and 256 samples per cycle (12.8 kHz) for enhanced measurement and waveform recording accuracy. The end-to-end transfer time for SV used in protection applications shall not exceed 3 ms, ensuring that protection algorithms receive fresh samples with negligible latency. GOOSE provides fast, reliable, peer-to-peer exchange of binary status

and control information among bay-level IEDs and between bay-level devices and process-level intelligent terminals. The end-to-end transmission time of GOOSE messages used for high-speed tripping, interlocking, and fast state exchange shall be within 3 ms. MMS provides client–server services over TCP/IP between bay IEDs and station-level servers, carrying supervisory, operational, and maintenance information such as protection operation reports, alarms, setting parameters, and fault records; it is not used for the most time-critical protection functions but for comprehensive system monitoring and control.

These communication services are mapped onto two deterministic Ethernet networks. The process bus interconnects process-level devices and bay-level IEDs, carrying high-rate SV streams and time-critical GOOSE messages, and therefore requires microsecond- to millisecond-level latency performance. The station bus links the bay and station levels, transporting MMS traffic for supervisory control and data acquisition, and—where required—GOOSE messages for inter-bay coordination, busbar protection, or fast station-level interlocking. In the substation considered in this work, both buses are implemented on a high-speed Ethernet infrastructure with link data rates not lower than 100 Mbps, ensuring adequate bandwidth for concurrent SV, GOOSE, MMS, and other auxiliary traffic.

A unified time-synchronization system ensures coherent timestamping and time alignment across all three levels of the digital substation. In typical deployments, station-level devices obtain time via the Simple Network Time Protocol (SNTP), which provides millisecond-level accuracy adequate for supervisory and other non-time-critical functions. In contrast, bay-level and process-level equipment are normally synchronized using either the Inter-Range

Instrumentation Group time code B (IRIG-B) or the IEEE 1588v2 Precision Time Protocol (PTP), both of which satisfy the microsecond-level timing requirements of protection and high-precision measurement applications. In the 110 kV Shenzhen digital substation investigated in this study, both BeiDou Navigation Satellite System (BDS) and the Global Positioning System (GPS) time references are available, with BDS used as the primary source, and the bay and process levels employ IEEE 1588v2 PTP as the main synchronization method, achieving a timing accuracy better than 1 μs across the IEDs.

Description of fault diagnosis problems in substations

With the digitalization of secondary systems in substations, real-time monitoring of system operating conditions and the collection of fault data have significantly improved.

When a fault occurs in the primary system, physical characteristics such as electrical quantities experience sudden fluctuations. These changes are detected by secondary protection devices, which activate PRs according to predefined logic and send trip signals to CBs to isolate the faulty equipment or line. If the main protection fails, backup protection devices take over (Supplementary Fig. 1b). During this process, PR and CB actions are recorded as alarm information. Fault waveform recordings capture high-frequency electrical variations, reflecting the transient characteristics of the fault. Additionally, Supervisory Control And Data Acquisition (SCADA) measurement data, with lower sampling frequencies, documents the grid's real-time steady-state conditions before and after the fault. Together, these multi-source data sources provide a comprehensive view of the fault process, supporting multi-dimensional fault diagnosis.

In the actual substation operations, factors such as equipment maintenance, operational adjustments, or other requirements result in dynamic changes in the primary system topology. The topology, which defines the connection relationships between substation equipment, directly determines the fault propagation path, the PR action logic, and the distribution patterns of electrical characteristics.

The mapping relationships between fault scenarios, alarm information, fault waveform recordings, and SCADA measurement data—given a known primary system topology—are described below (Supplementary Fig. 1c). A single alarm sequence may correspond to multiple fault scenarios because similar PRs can be triggered by different faults, making fault pattern differentiation challenging. Fault waveform recordings accurately capture changes in the primary system during a fault but provide limited insights into secondary system anomalies. SCADA measurement data offers steady-state information before and after the fault, but lacks the ability to capture transient fault characteristics and precise fault locations.

Therefore, relying on a single data source for fault diagnosis has significant limitations, as it fails to capture the complexity and diversity of fault scenarios. Integrating multi-source data is essential for accurately diagnosing fault locations, fault types, and PR statuses. These data sources complement each other, collectively providing a detailed understanding of the fault mechanism and propagation. Based on this integration, a mathematical model for fault diagnosis in substation systems can be formulated, as shown in Supplementary Equation (12):

$$\begin{cases} \tilde{\mathbf{Y}} = f\left(\mathbf{X}_1, \mathbf{X}_2, \mathbf{X}_3, \mathbf{A}; \boldsymbol{\theta}\right) \\ \tilde{\mathbf{Y}} = \left[\tilde{\mathbf{Y}}_1, \tilde{\mathbf{Y}}_2, \tilde{\mathbf{Y}}_3\right] \\ \tilde{\mathbf{Y}}_1 = \left[\tilde{y}_1^1, \tilde{y}_1^2, \cdots, \tilde{y}_1^n\right], \tilde{y}_1^i \in [0,1] \\ \tilde{\mathbf{Y}}_2 = \left[\tilde{y}_2^1, \tilde{y}_2^2, \cdots, \tilde{y}_2^m\right], \tilde{y}_2^i \in [0,1] \\ \tilde{\mathbf{Y}}_3 = \left[\tilde{y}_3^1, \tilde{y}_3^2, \cdots, \tilde{y}_3^p\right], \tilde{y}_3^i \in [0,1] \end{cases} \tag{12}$$

where $\mathbf{X}_1$ represents the sequence of alarm information after a fault; $\mathbf{X}_2$ represents the fault waveform recordings of each electrical node; $\mathbf{X}_3$ represents the SCADA measurement data; **A** represents the substation system's primary topology matrix; *f* represents the nonlinear mapping model from these data to the diagnostic results; $\boldsymbol{\theta}$ is the parameter set of the model; $\tilde{\mathbf{Y}} = \left[\tilde{\mathbf{Y}}_1, \tilde{\mathbf{Y}}_2, \tilde{\mathbf{Y}}_3\right]$ represent the diagnostic results for the primary fault location, fault type, and PRs failure, respectively; $\tilde{y}_1, \tilde{y}_2, \tilde{y}_3$ represent the predicted probabilities for corresponding faults; *n*, *m*, and *p* are the number of potential fault locations, fault types, and PRs, respectively.

Latency analysis of the digital twin system

The end-to-end diagnostic latency of the digital twin system can be understood as the cumulative duration of three sequential stages: (i) acquiring the required data, (ii) processing and organizing the data into model inputs, and (iii) executing the diagnostic model.

- Stage 1 — Data acquisition.

Before the fault occurs, the digital twin continuously receives real-time switching states of circuit breakers and disconnectors via IEC 61850 GOOSE and MMS services, ensuring that the pre-fault topology is already available at the moment the fault begins. During the fault, bay-level protection IEDs generate microsecond-synchronized Sequence-of-Events (SOE) logs, and SCADA analog measurements continue to be reported through MMS. These messages typically reach the station level within milliseconds and therefore do not contribute significantly to

diagnostic latency.

In contrast, the acquisition of fault waveform data is inherently slower. Protection IEDs and standalone fault recorders must capture at least 2 seconds of pre-fault data and the entire fault transient before a COMTRADE file can be generated, and this file-generation process is time-consuming. In the 110 kV Shenzhen digital substation studied in this work, generating the waveform files from multiple triggered recorders takes approximately 50 seconds, making this step the dominant contributor to the end-to-end latency. Once generated, the waveform files—typically about 20 MB each—are uploaded to the station level via IEC 61850 MMS file services in around 10 seconds.

- Stage 2 — Data processing.

After all waveform files are available at the station level, the digital twin reconstructs the pre-fault topology, parses the COMTRADE channels, extracts the necessary diagnostic features, and formats the combined SOE, SCADA, and waveform information into the model's input structure. These operations are computationally lightweight and require roughly 1 s.

- Stage 3 — Model inference.

The processed data are then fed into the diagnostic model, which runs on an Intel Xeon E3-1230 v5 CPU and an NVIDIA RTX 3090 GPU. The inference stage is fast, producing the final diagnostic decision within approximately 0.5 s.

Because SOE and SCADA reporting overlap with the waveform acquisition phase, the overall latency is determined primarily by the waveform file–generation delay. Let t_1 denote the time

for recorder-side file generation and upload, t_2 the data-processing time, and t_3 the inference time. The end-to-end latency is therefore

$$T_{\text{latency}} = t_1 + t_2 + t_3 \tag{13}$$

Given that $t_1 \approx 60$ s, $t_2 \approx 1$ s, and $t_3 \approx 0.5$ s, the overall latency is dominated by the waveform file–generation process.

Supplementary Discussion

Generalization saturation and mitigation of topology overfitting

The system analyzed in this study corresponds to a single 110 kV smart substation (Supplementary Fig. 10a). Due to its compact scale, fixed equipment configuration, and strict operational constraints, the number of physically feasible operating topologies is inherently limited. These admissible topologies arise from permissible combinations of busbar configurations and transformer operating modes.

1. 110 kV Busbar Configuration

The substation utilizes a double-bus structure with a bus-tie breaker, leading to three operational modes for the 110 kV busbar:

- Two bus sections operating independently (with the bus-tie breaker open).
- Bus-tie breaker closed, Line I energized, Line II standby.
- Bus-tie breaker closed, Line II energized, Line I standby.

Thus, the 110 kV busbar contributes three possible topologies (Supplementary Fig. 10b).

2. Transformer Operating Modes

The substation operates three transformers (#1, #2, #3), and the admissible operating combinations are constrained by system supply requirements:

- Three-transformer operation

  Transformer #2 has two valid wiring schemes: connected to 110 kV busbar I or busbar II, allowing for 2 configurations (Supplementary Fig. 10c).

- Two-transformer operation:

  Any two of the three transformers may operate, and if transformer #2 is included, it has two wiring options, resulting in 5 configurations (Supplementary Fig. 10d).

- Single-transformer operation:

  Only transformer #2 can operate alone, as the single operation of transformer #1 or #3 does not provide sufficient power to all four 10 kV bus sections simultaneously, leading to 2 configurations (Supplementary Fig. 10e).

This gives a total of 9 transformer configurations.

3. 10 kV Busbar Configurations

The 10 kV busbar configuration is determined directly by the operating transformer configuration, ensuring that power is supplied to all feeders. Therefore, the 10 kV busbar topology is entirely defined by the transformer operational modes.

4. Total Feasible Topologies

Combining the above operational constraints, 3×9=27 distinct operational topologies for

the substation, which form the complete and physically realistic topology space for the system. No additional topologies are permitted by real-world operational constraints.

5. Generalization Across Topologies

Although 27 distinct topologies are theoretically possible, in practice, the generalization of the model is more influenced by the structural diversity within the training data rather than the absolute number of topologies. To ensure robust model generalization, the training dataset includes a representative subset of these topologies that captures the most important structural variations:

- The 3 distinct 110 kV busbar modes
- Representative transformer operational modes (including three-transformer, two-transformer, and one-transformer configurations)
- Both wiring configurations of transformer #2

The ten topologies used in model training already cover the essential structural variations of the substation. Because the remaining feasible topologies can be viewed as minor variations or combinations of these structural motifs, the model learns generalizable topology-dependent patterns rather than memorizing specific configurations. Consequently, even though only ten topologies are included in the training set, they provide sufficient diversity for the model to generalize reliably to unseen topologies, effectively avoiding overfitting and ensuring stable diagnostic performance in new operational conditions.

Supplementary References

1. Lee, H.-J., Ahn, B.-S. & Park, Y.-M. A fault diagnosis expert system for distribution substations. *IEEE Transactions on Power Deliv*. **15**, 92–97, DOI: 10.1109/61.847234 (2000).

2. Yongli, Z., Limin, H. & Jinling, L. Bayesian networks-based approach for power systems fault diagnosis. *IEEE Transactions on Power Deliv.* **21**, 634–639, DOI: 10.1109/TPWRD.2005.858774 (2006).

3. Shao, N., Chen, Q., Dong, Y., Ding, W. & Wang, L. Power system fault diagnosis method based on intuitionistic fuzzy sets and incidence matrices. *IEEE Transactions on Power Deliv.* **38**, 3924–3938, DOI: 10.1109/TPWRD.2023.3294883 (2023).

4. Hor, C.-L., Crossley, P. A. & Watson, S. J. Building knowledge for substation-based decision support using rough sets. *IEEE Transactions on Power Deliv.* **22**, 1372–1379, DOI: 10.1109/TPWRD.2006.886783 (2007).

5. Zhang, Y., Chung, C. Y., Wen, F. & Zhong, J. An analytic model for fault diagnosis in power systems utilizing redundancy and temporal information of alarm messages. *IEEE Transactions on Power Syst.* **31**, 4877–4886, DOI: 10.1109/TPWRS.2016.2519452 (2016).

6. Xu, B., Yin, X., Yin, X., Wang, Y. & Pang, S. Fault diagnosis of power systems based on temporal constrained fuzzy petri nets. *IEEE Access* **7**, 101895–101904, DOI: 10.1109/ACCESS.2019.2930545 (2019).

7. Wang, S.-P. & Zhao, D.-M. A hierarchical power grid fault diagnosis method using multi-source information. *IEEE Transactions on Smart Grid* **11**, 2067–2079, DOI: 10.1109/TSG.2019.2946901 (2020).

8. Jiang, X. *et al.* Busbar fault diagnosis method based on multi-source information fusion. *Front. Energy Res.* **12**, DOI:10.3389/fenrg.2024.1443570 (2024).

9. Chen, K., Hu, J., Zhang, Y., Yu, Z. & He, J. Fault location in power distribution systems via deep graph convolutional networks. *IEEE J. on Sel. Areas Commun.* **38**, 119–131, DOI: 10.1109/JSAC.2019.2951964 (2020).

10. Thomas, J. B., Chaudhari, S. G., K. V., S. & Verma, N. K. Cnn-based transformer model for fault detection in power system networks. *IEEE Transactions on Instrumentation Meas.* **72**, 1–10, DOI: 10.1109/TIM.2023.3238059 (2023).

11. Nguyen, B. L. H., Vu, T. V., Nguyen, T.-T., Panwar, M. & Hovsapian, R. Spatial-temporal recurrent graph neural networks for fault diagnostics in power distribution systems. *IEEE Access* **11**, 46039–46050, DOI: 10.1109/ACCESS.2023.3273292(2023).

12. Ding, L. *et al.* Topology-aware fault diagnosis for microgrid clusters with diverse scenarios generated by digital twins. *Appl. Energy* **378**, 124794, DOI: 10.1016/j.apenergy.2024.124794 (2025).

13. Sidhu, T. & Gangadharan, P. Control and automation of power system substation using iec61850 communication. In *Proceedings of 2005 IEEE Conference on Control Applications, 2005. CCA 2005.*, 1331–1336, DOI: 10.1109/CCA.2005. 1507316 (2005).
