# Supplementary material for: Digital twin-driven fault diagnosis of power substations by multi-modal fusion learning
Source: Nat Commun. 2026 May 20;17:6628. doi: 10.1038/s41467-026-73483-5 (PMC13381694; doi:10.1038/s41467-026-73483-5)
Supplement: Supplementary file 2 — Description of Additional Supplementary Files [file 41467_2026_73483_MOESM2_ESM.pdf]

Description of Additional Supplementary Files

Supplementary Data:

Performance metrics of all evaluated models across all benchmark tasks presented in the **Manuscript**.

Supplementary Software:

The CloudPSS digital-twin simulation model of the referenced substation, including an instruction file detailing uploading and execution on the CloudPSS platform.
