# Supplementary material for: Digital twin-driven fault diagnosis of power substations by multi-modal fusion learning
Source: Nat Commun. 2026 May 20;17:6628. doi: 10.1038/s41467-026-73483-5 (PMC13381694; doi:10.1038/s41467-026-73483-5)

**Instruction for Using the**

**Substation Simulation Model on CloudPSS**

This document provides step-by-step instructions for loading, inspecting, and running the provided substation simulation model (model_wu-yl23_substation_model.zip) on the CloudPSS SimStudio platform. It also describes how to create and use an SDK for batch fault simulation via Python scripts.

1. **Create a CloudPSS Account**
2. Open the CloudPSS registration page at:
   [**https://cloudpss.net/account/simstudio**](https://cloudpss.net/account/simstudio)
3. Create a new user account following the on-screen instructions.


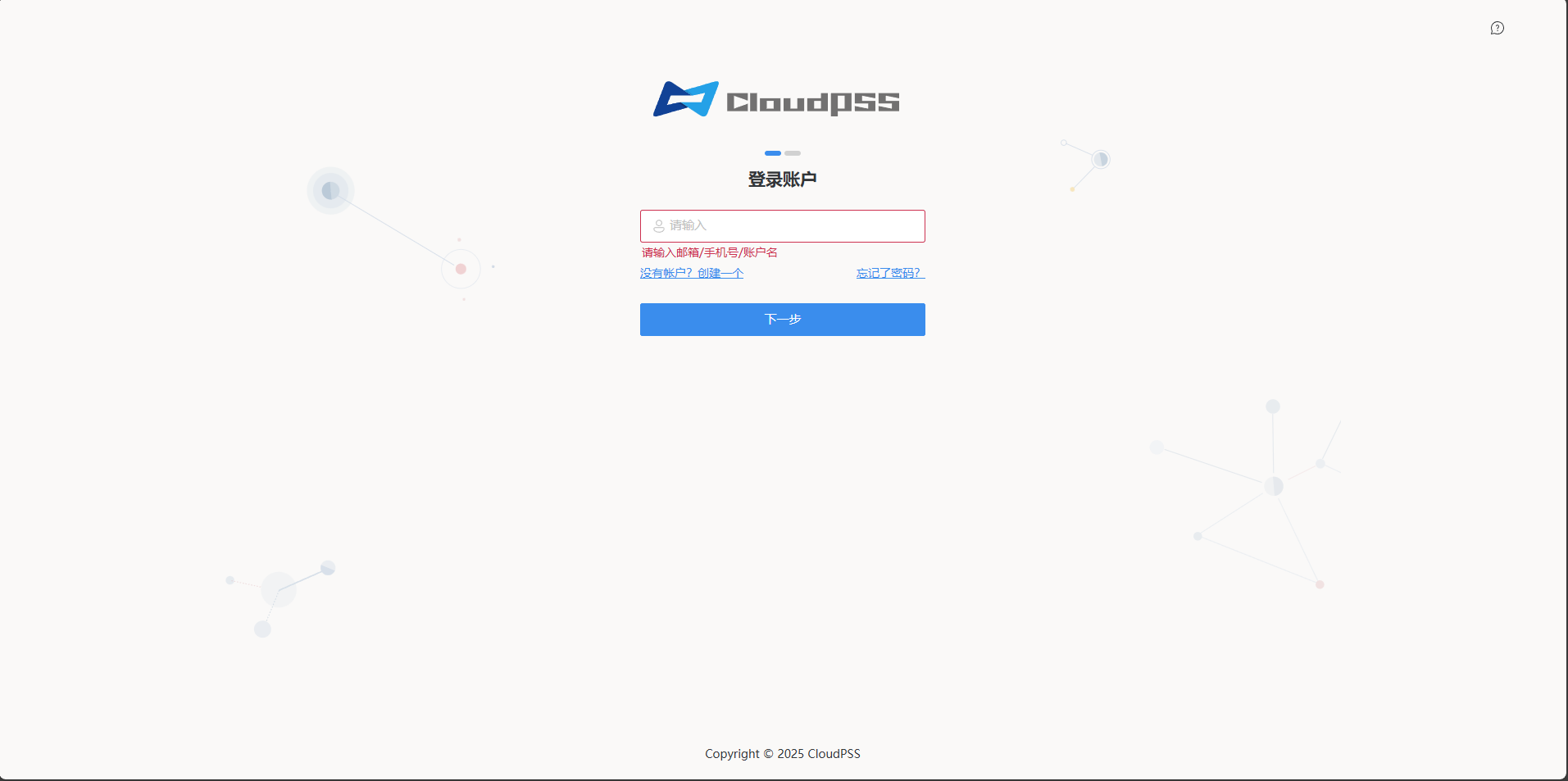


1. **Access SimStudio**
2. Log in to your CloudPSS account.
3. Navigate to SimStudio from the main dashboard.


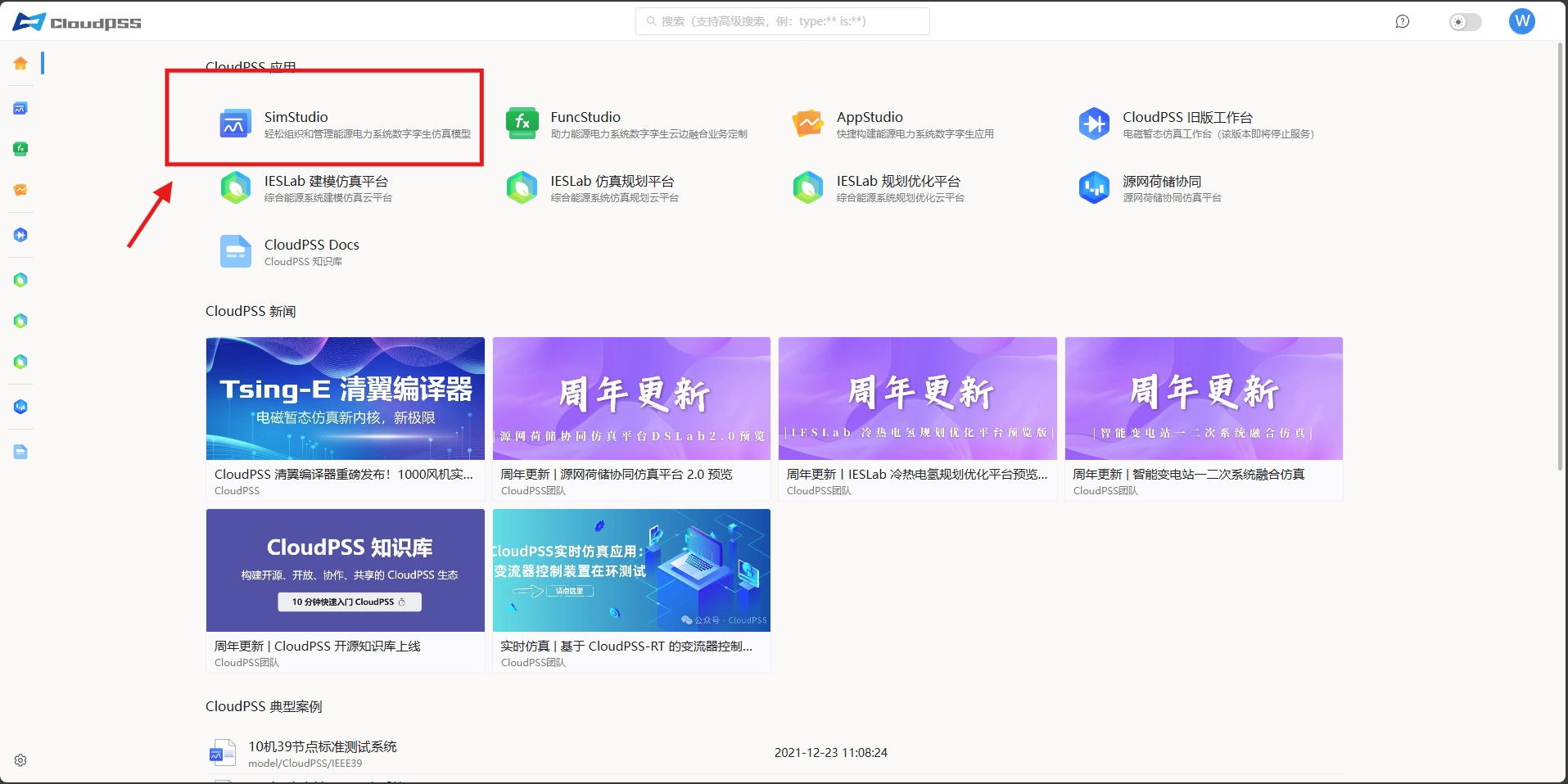


1. **Upload the Substation Model**
2. Download the supplementary software package and locate the attached model file:
   **model_wu-yl23_substation_model.zip**
3. In SimStudio, upload the model file using the **Upload** function.


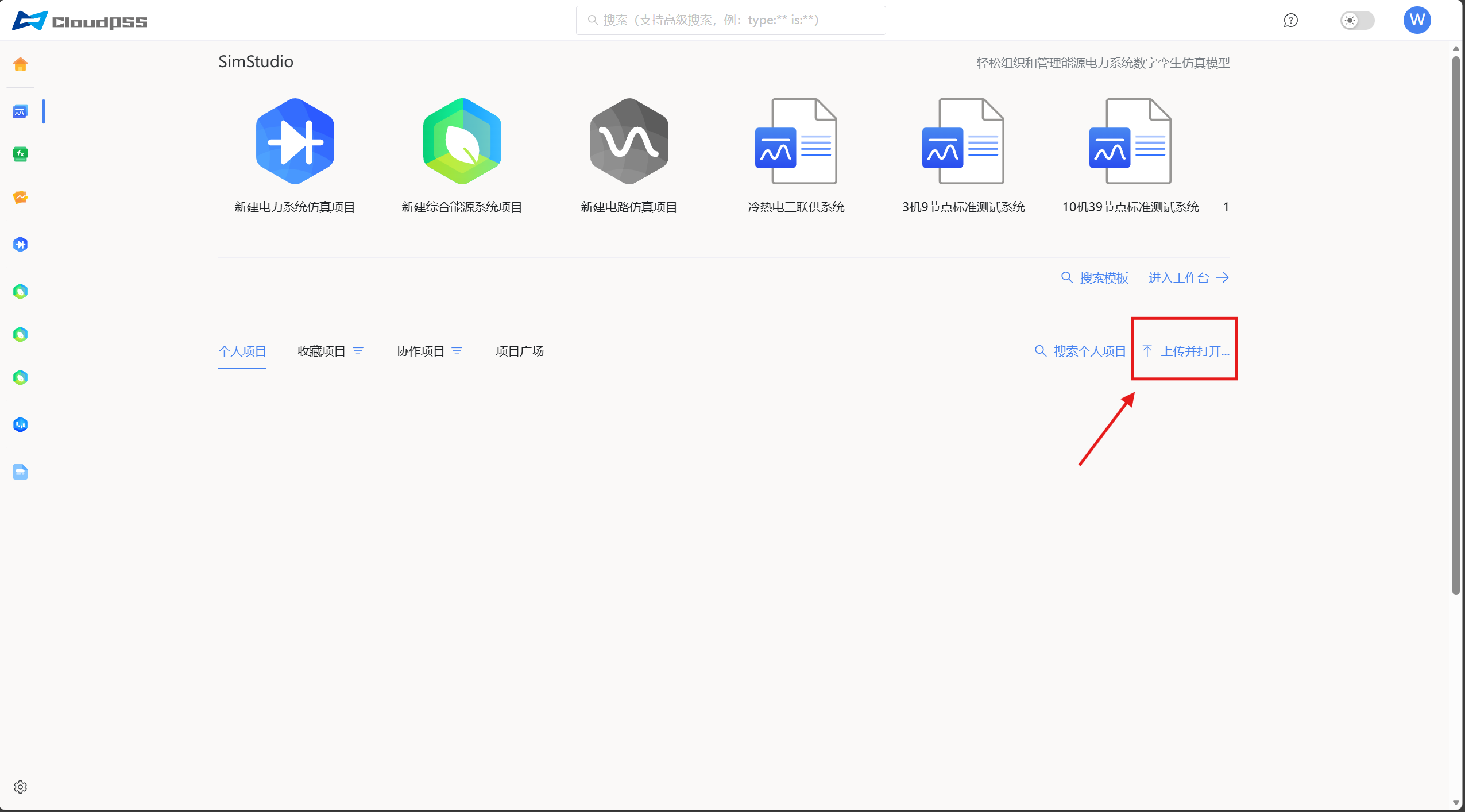

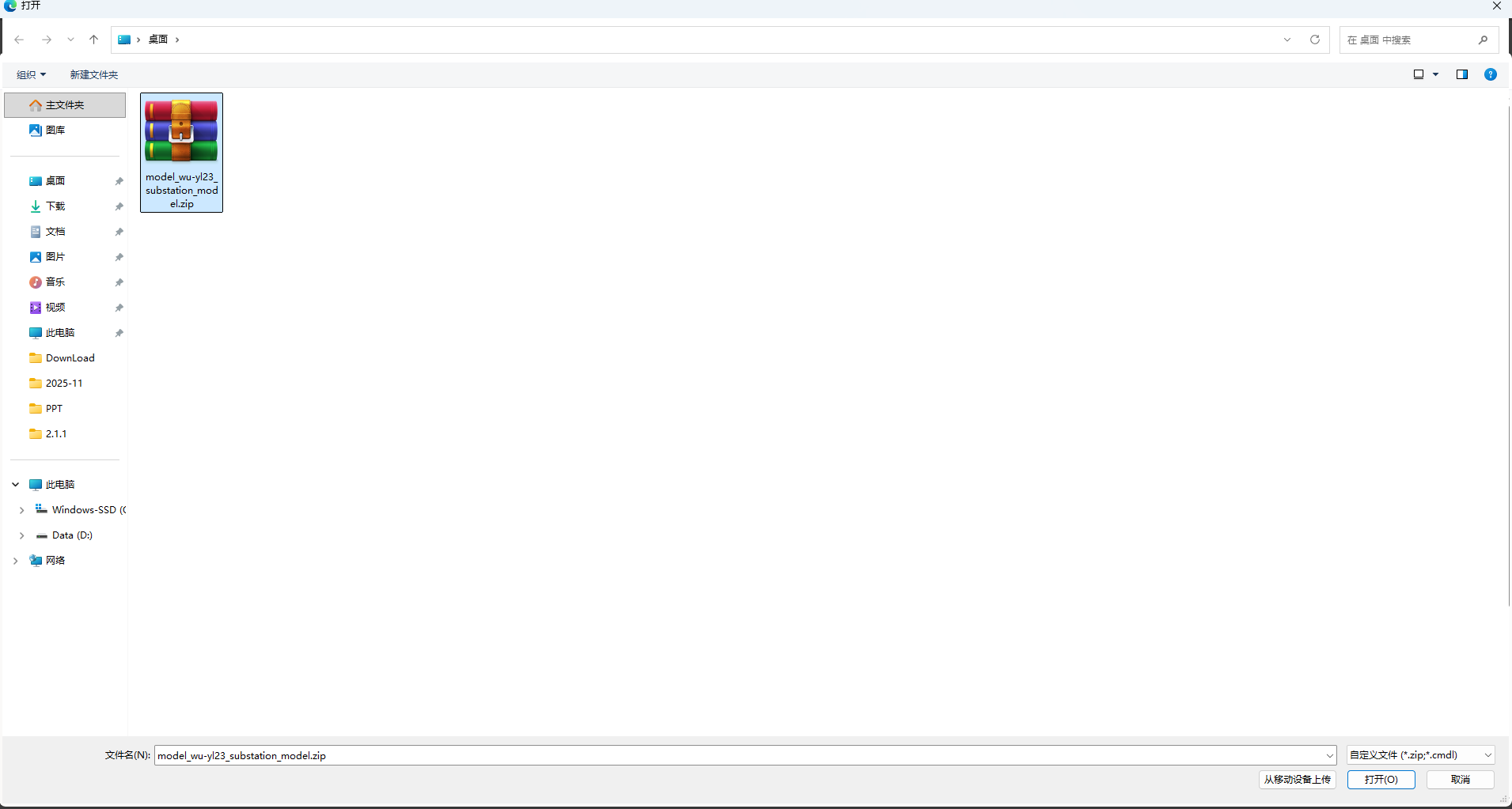


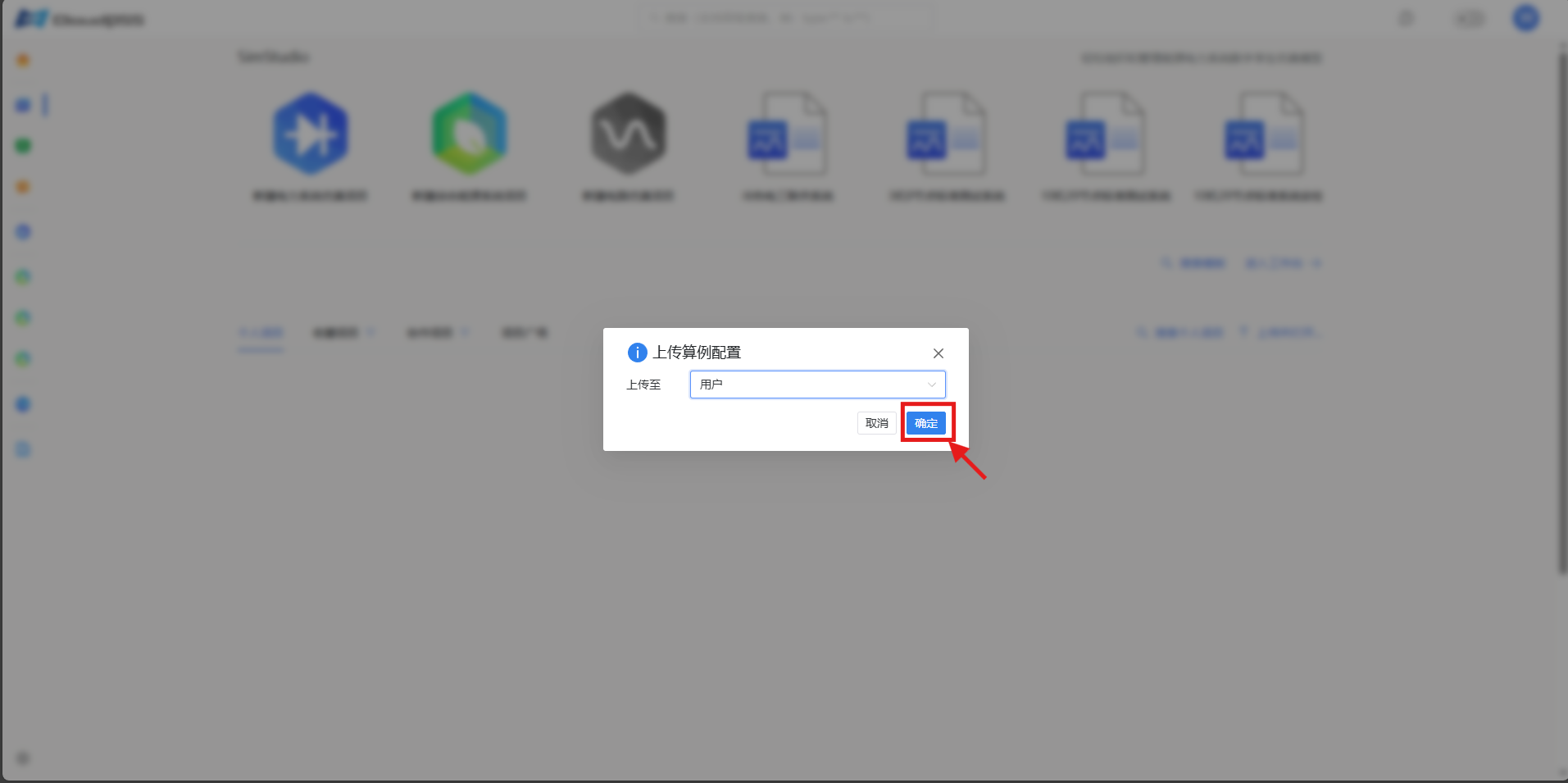

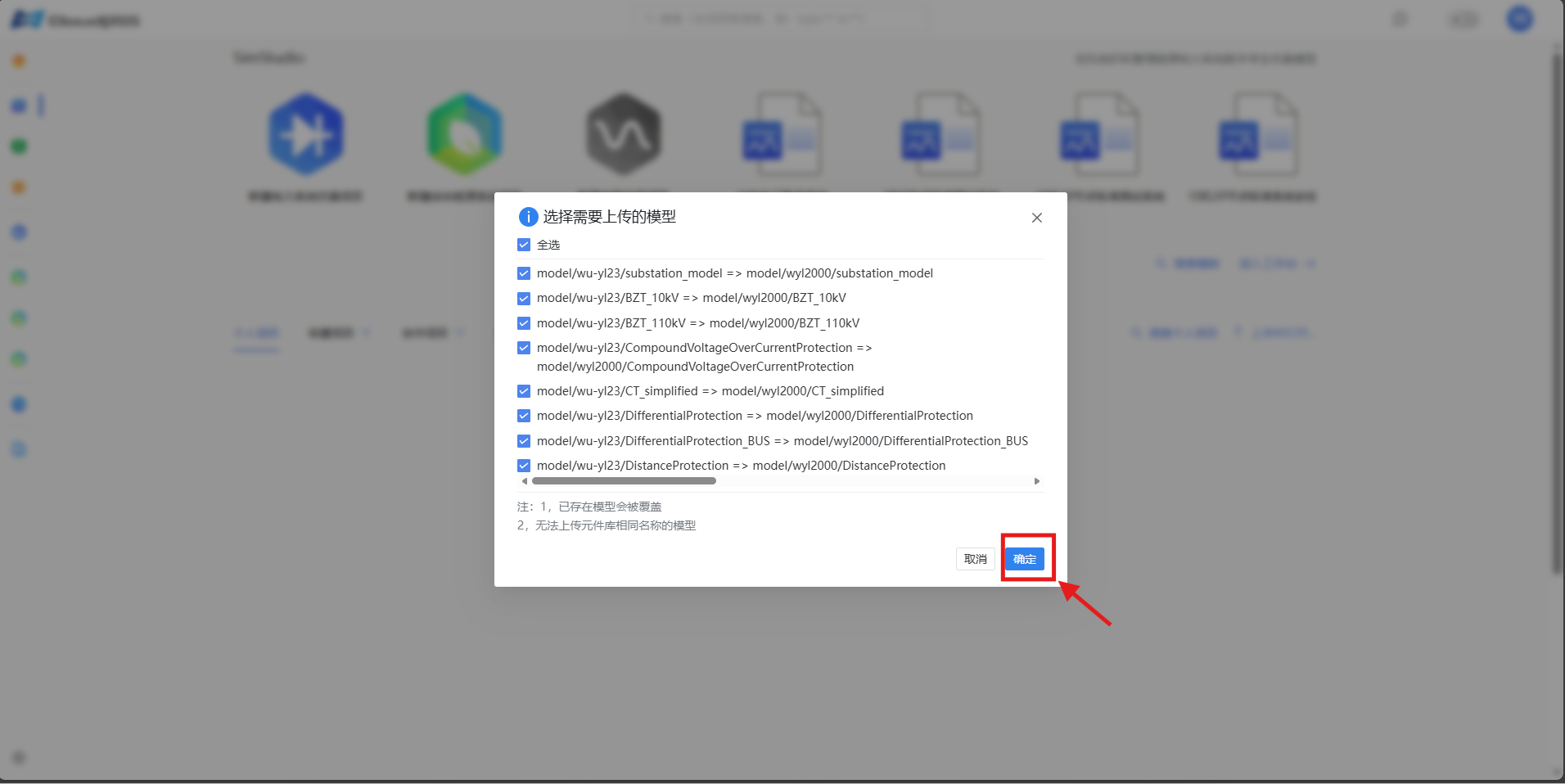


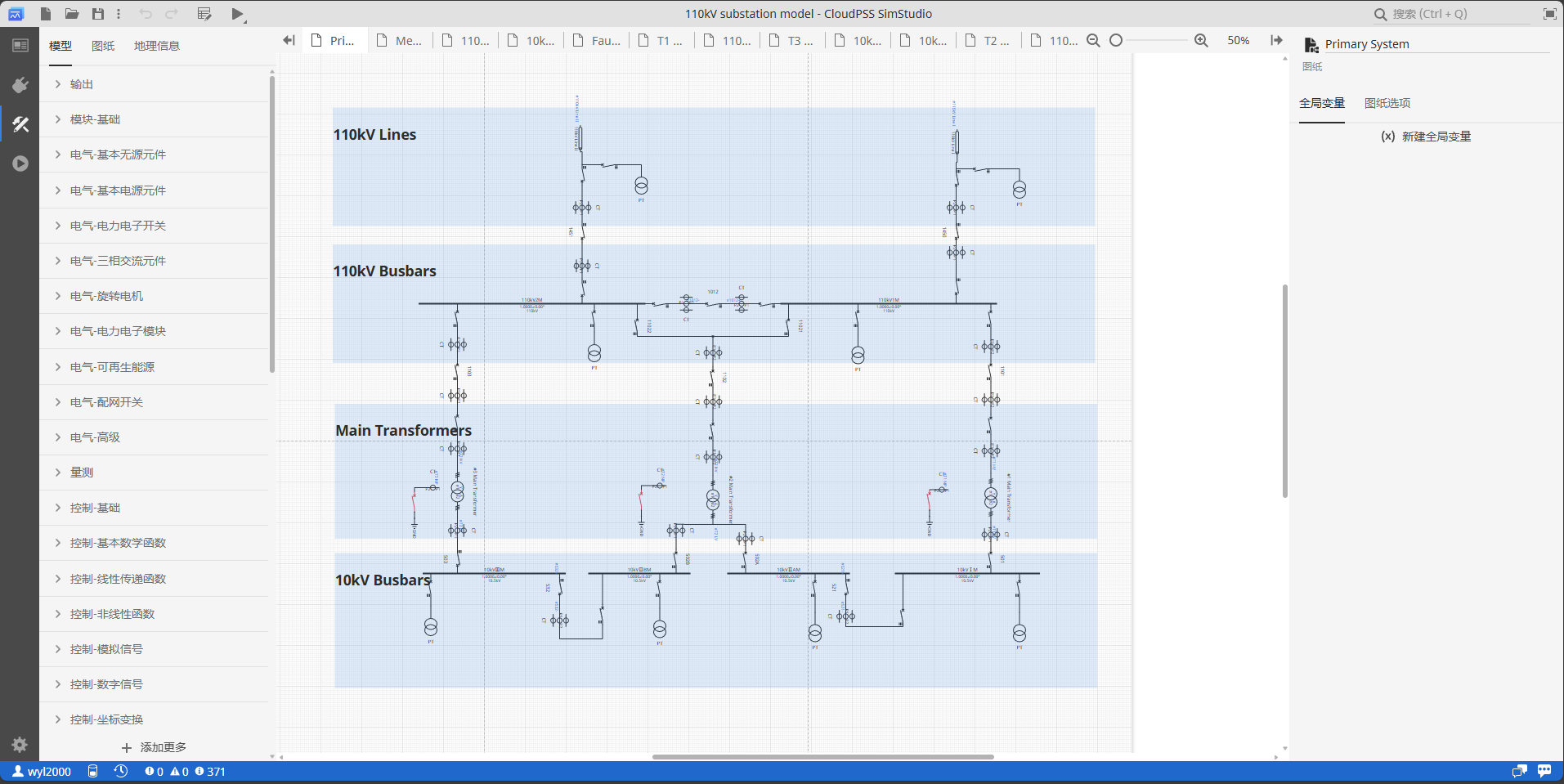


1. **View the Model Structure**

Once imported, the model can be viewed across multiple **Diagram** pages including Primary system model (one-line diagram) and Secondary protection and control model. Click the **“Diagram”** button to switch between different layers/pages.


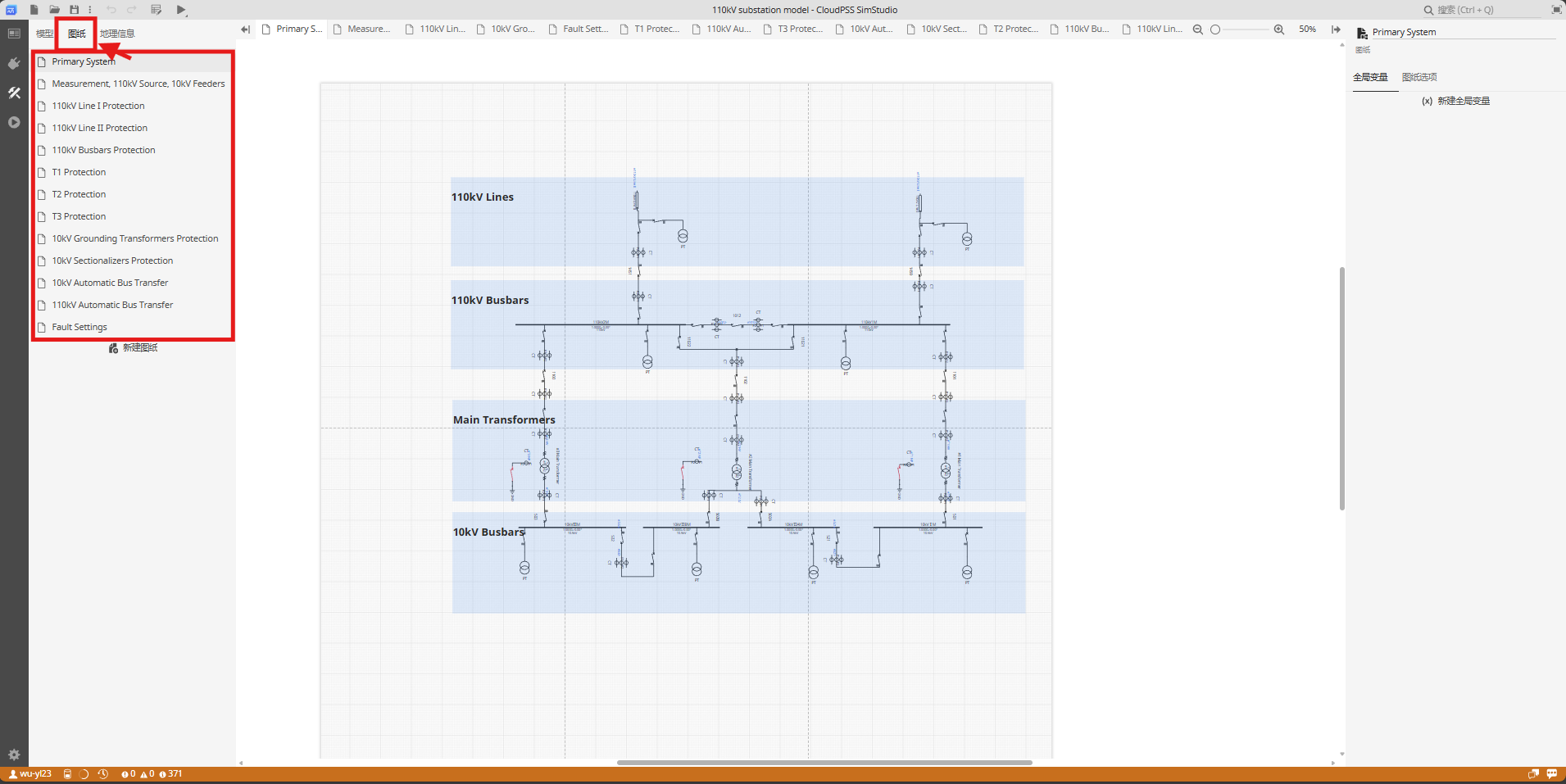

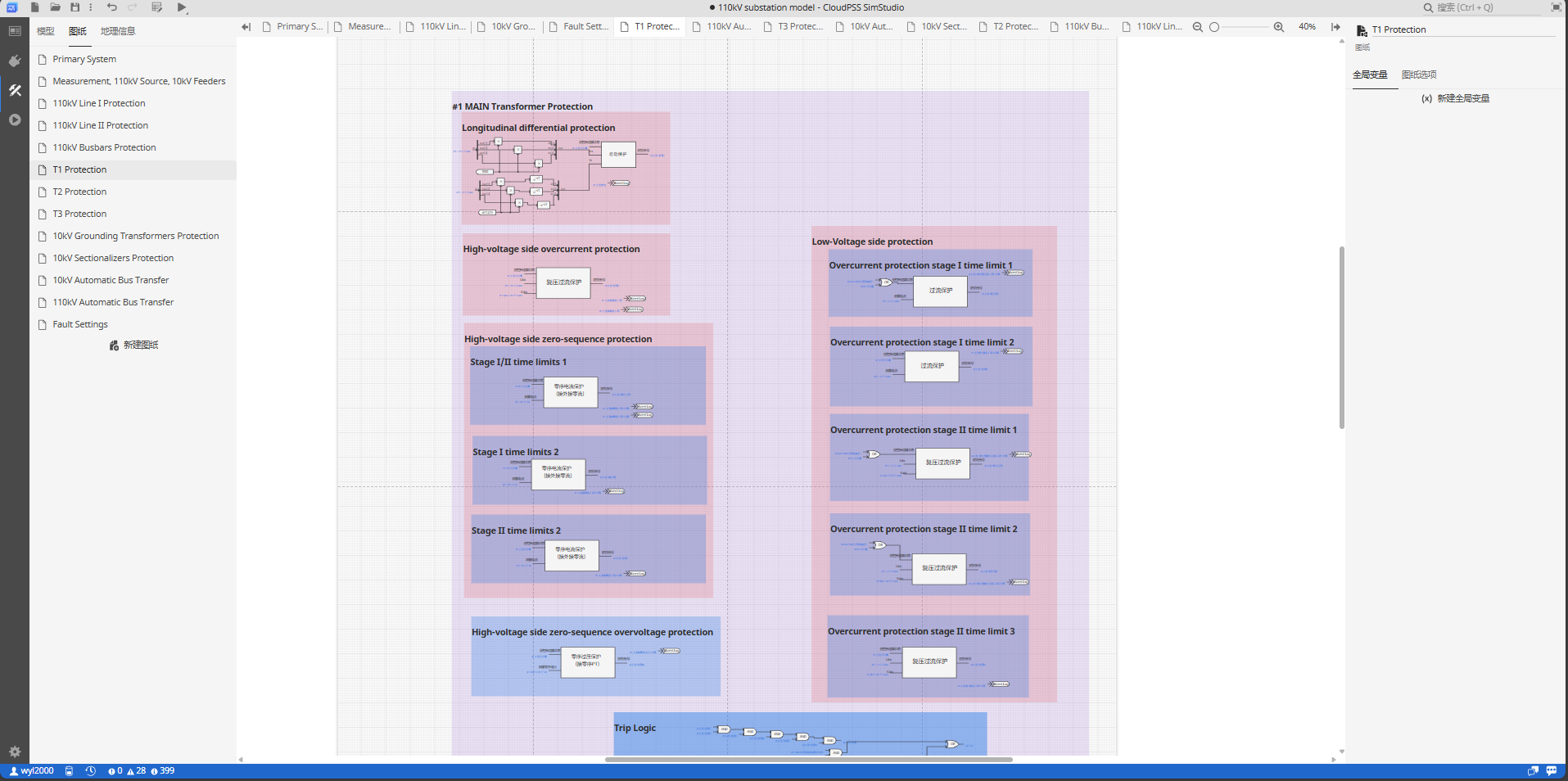


1. **Configure Simulation Parameters**

In the **Configuration (config)** panel, users may:

- Modify the initial state of circuit breakers to adjust system topology
- Set fault parameters (location, type, impedance, duration, etc.)
- Adjust other simulation options


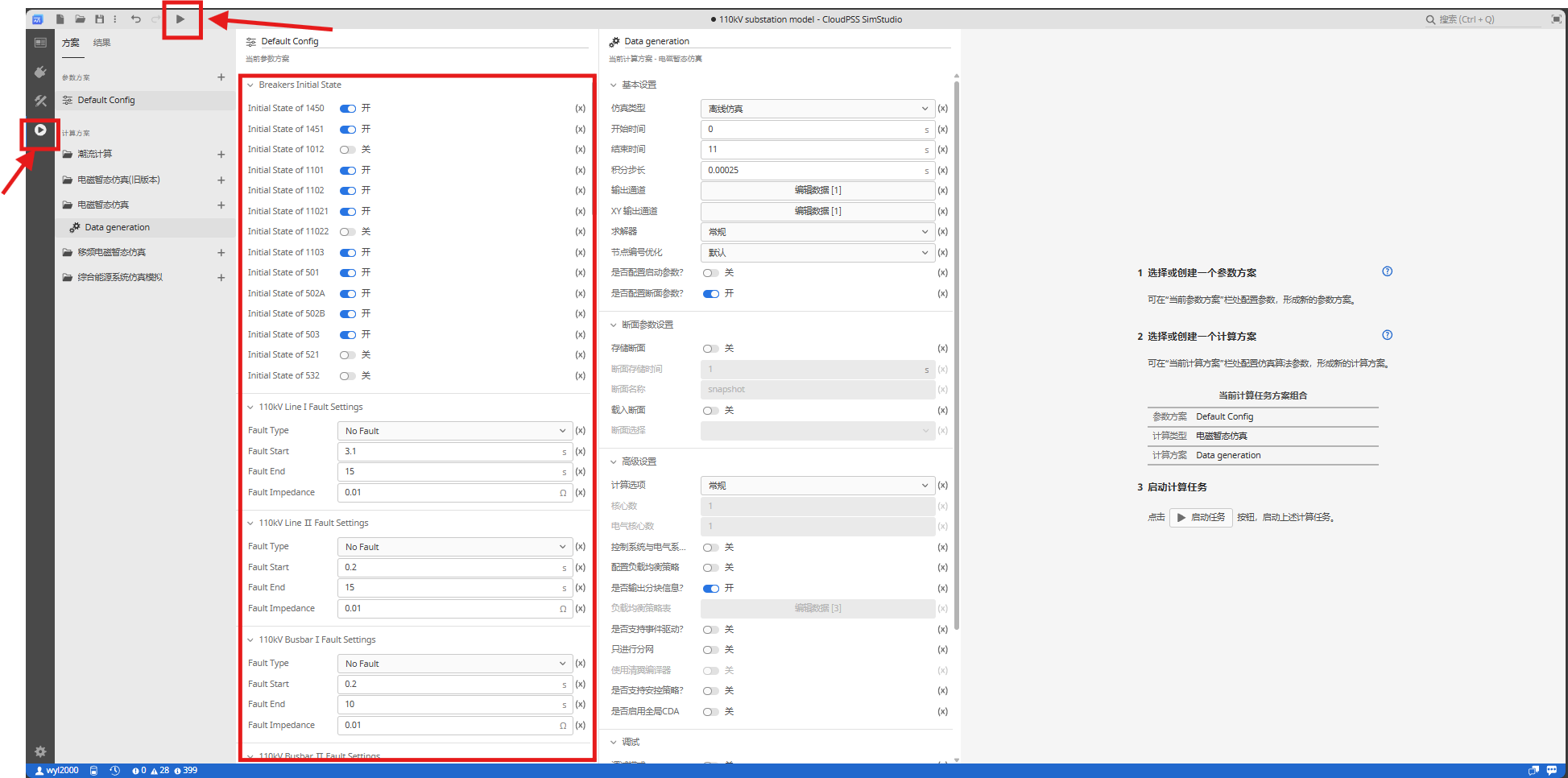


1. **Run the Simulation**

After configuration, click **Run** to execute the simulation. Upon completion, the system outputs:

- Alarm messages
- Waveforms and measurement results


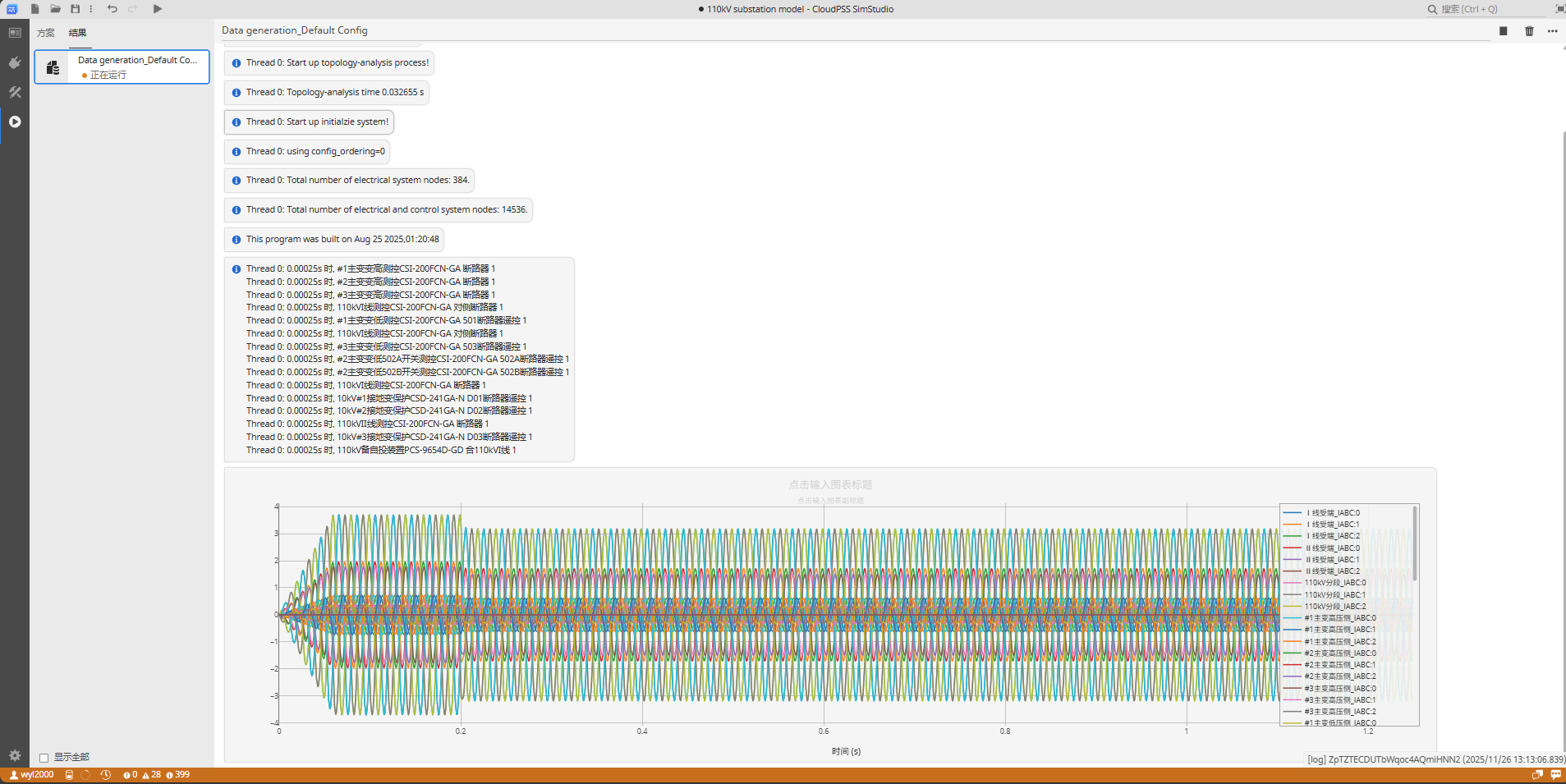


For more details, refer to the **CloudPSS Help Documentation**.


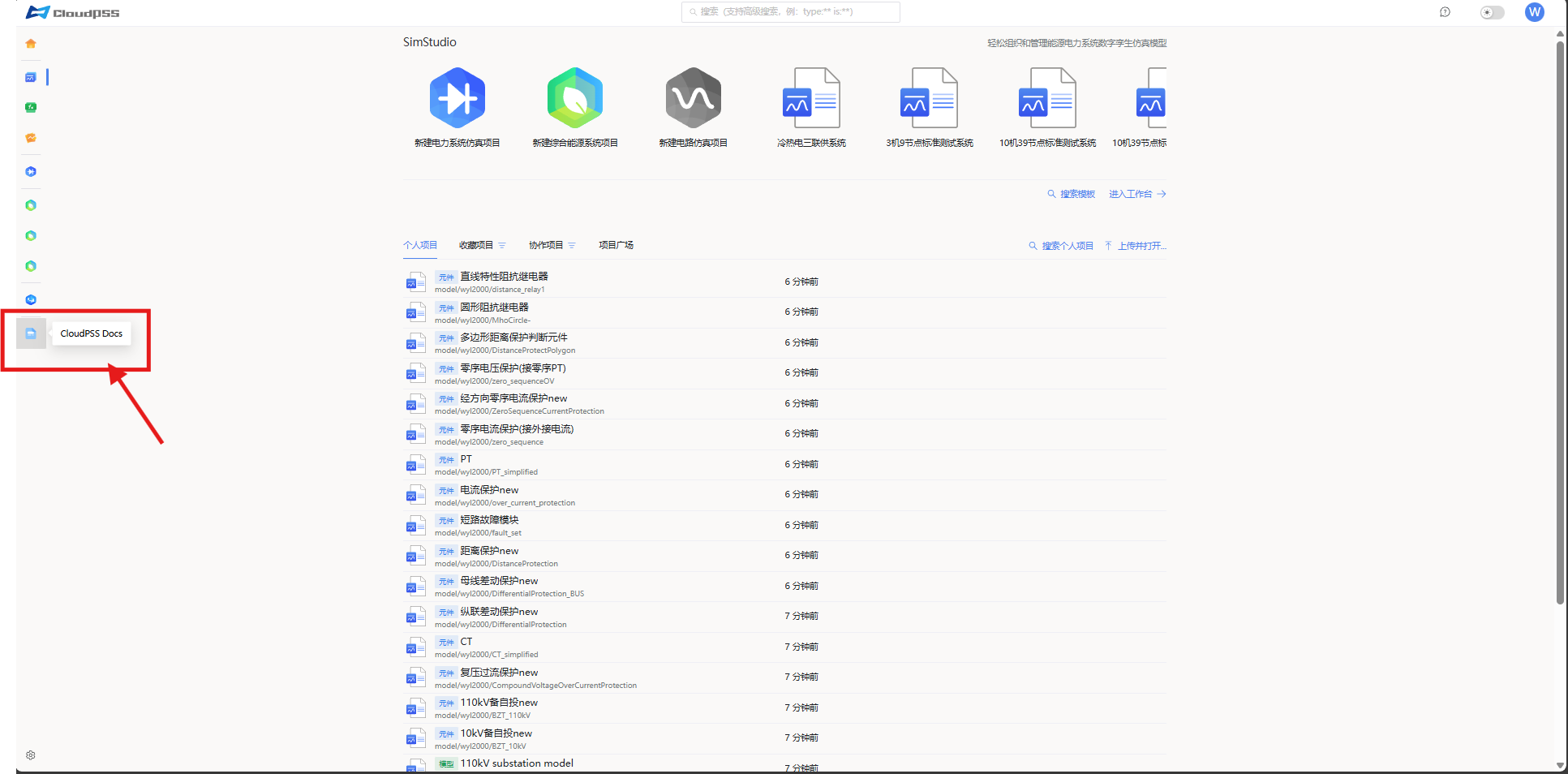


1. **Create and Use an SDK**

CloudPSS allows users to create a personal SDK (Software Development Kit) for programmatic access to the model.

1. Open the **SDK Creation** panel and generate an SDK for this project.


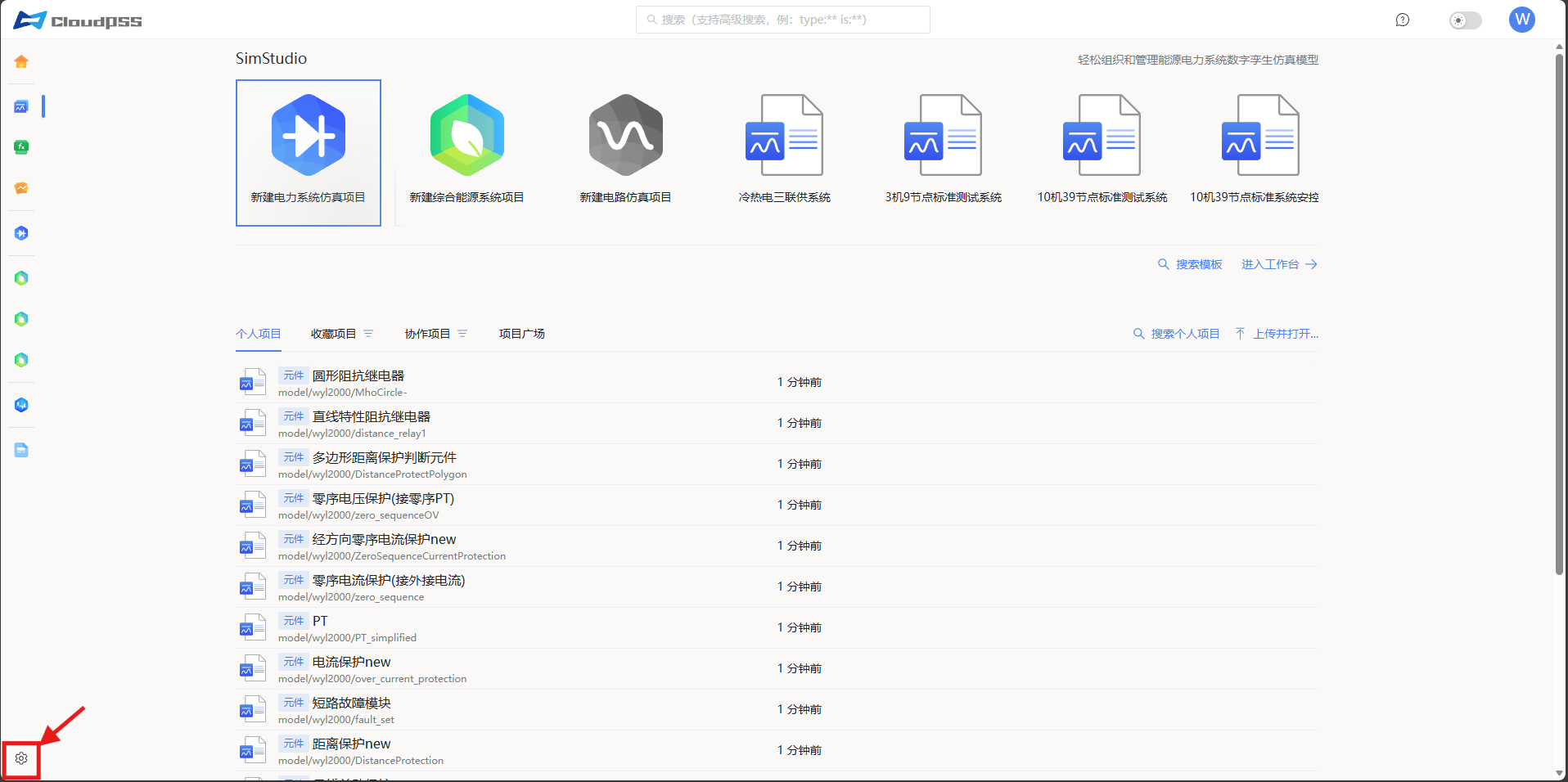

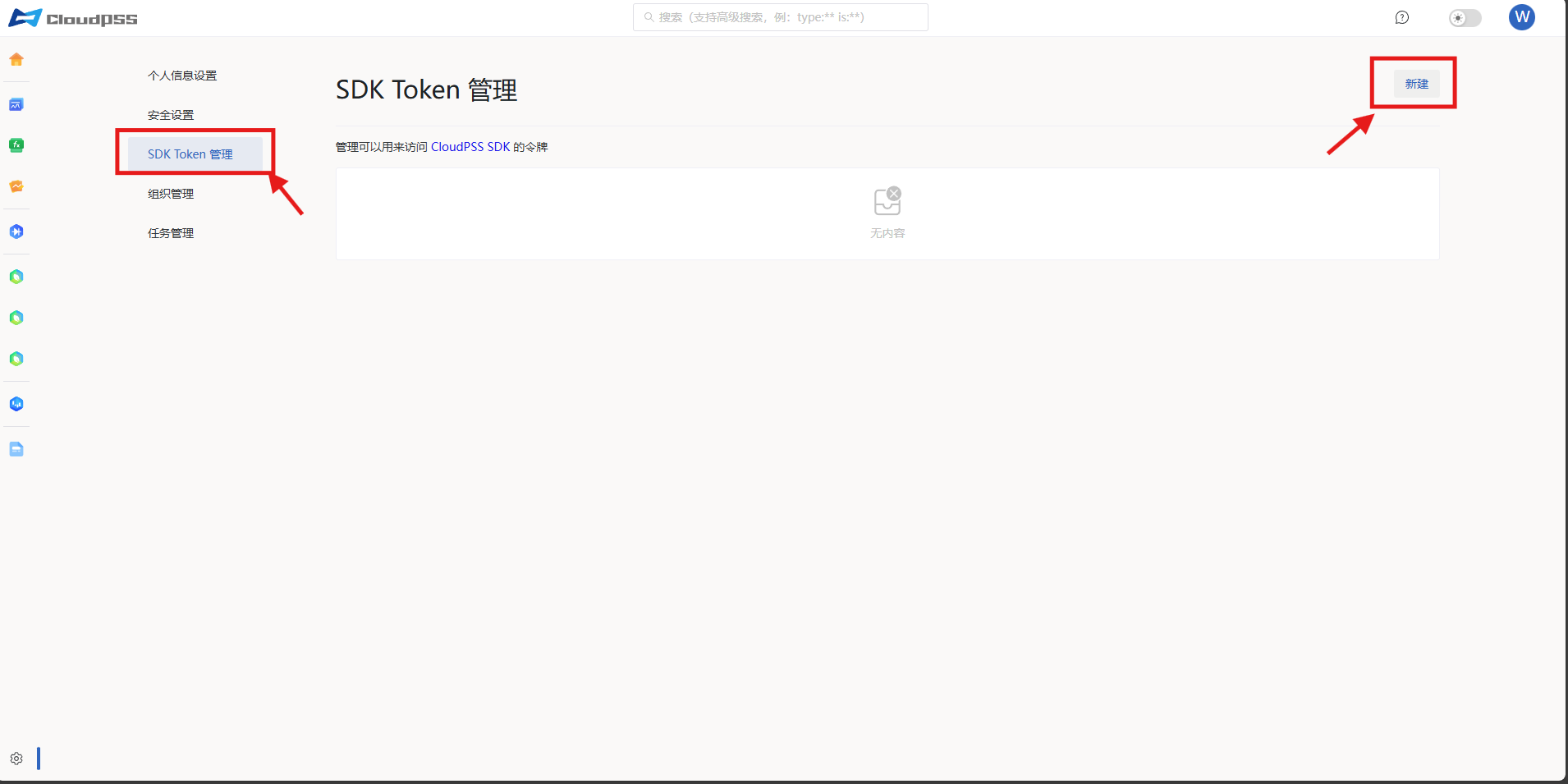


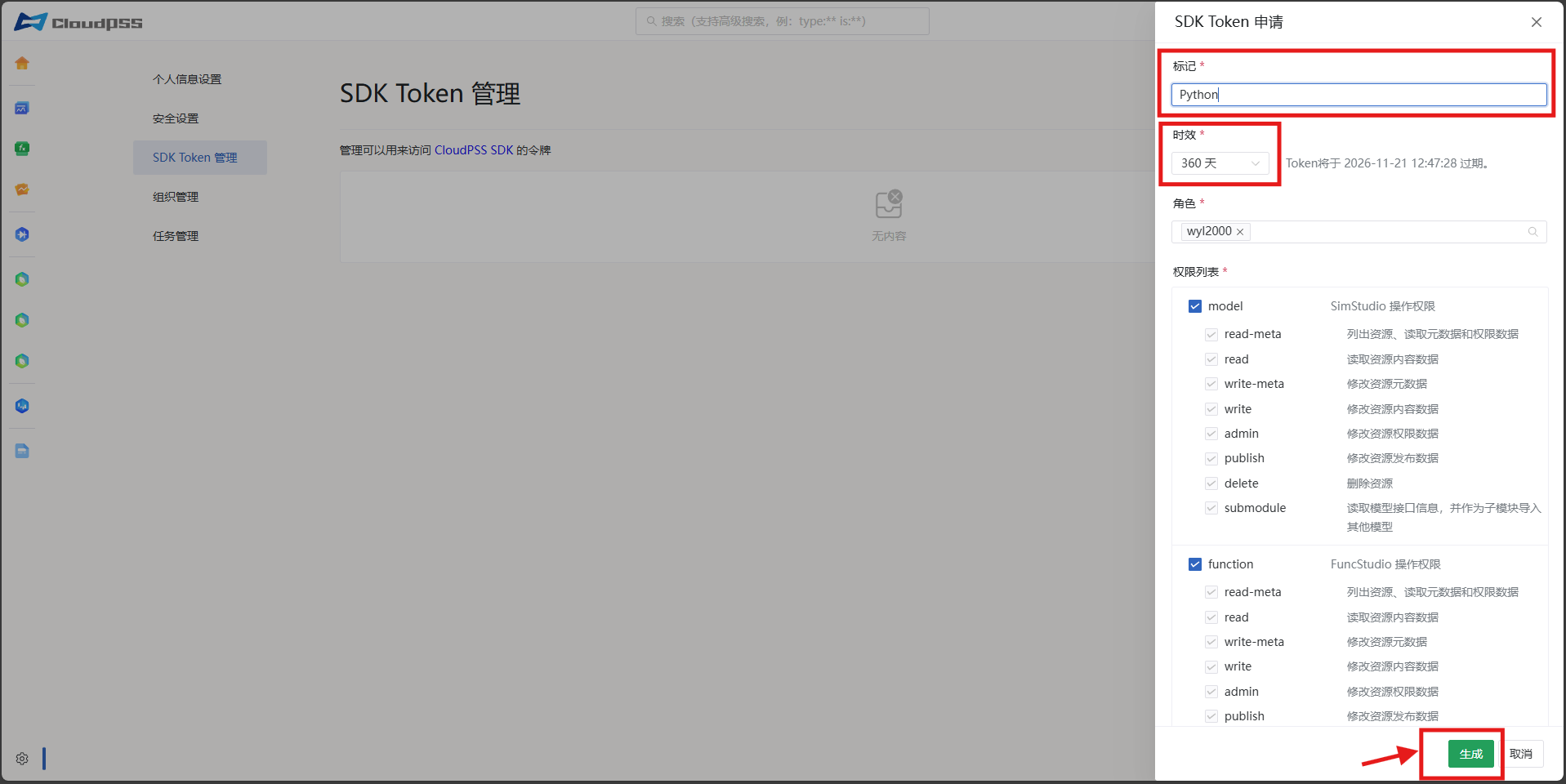


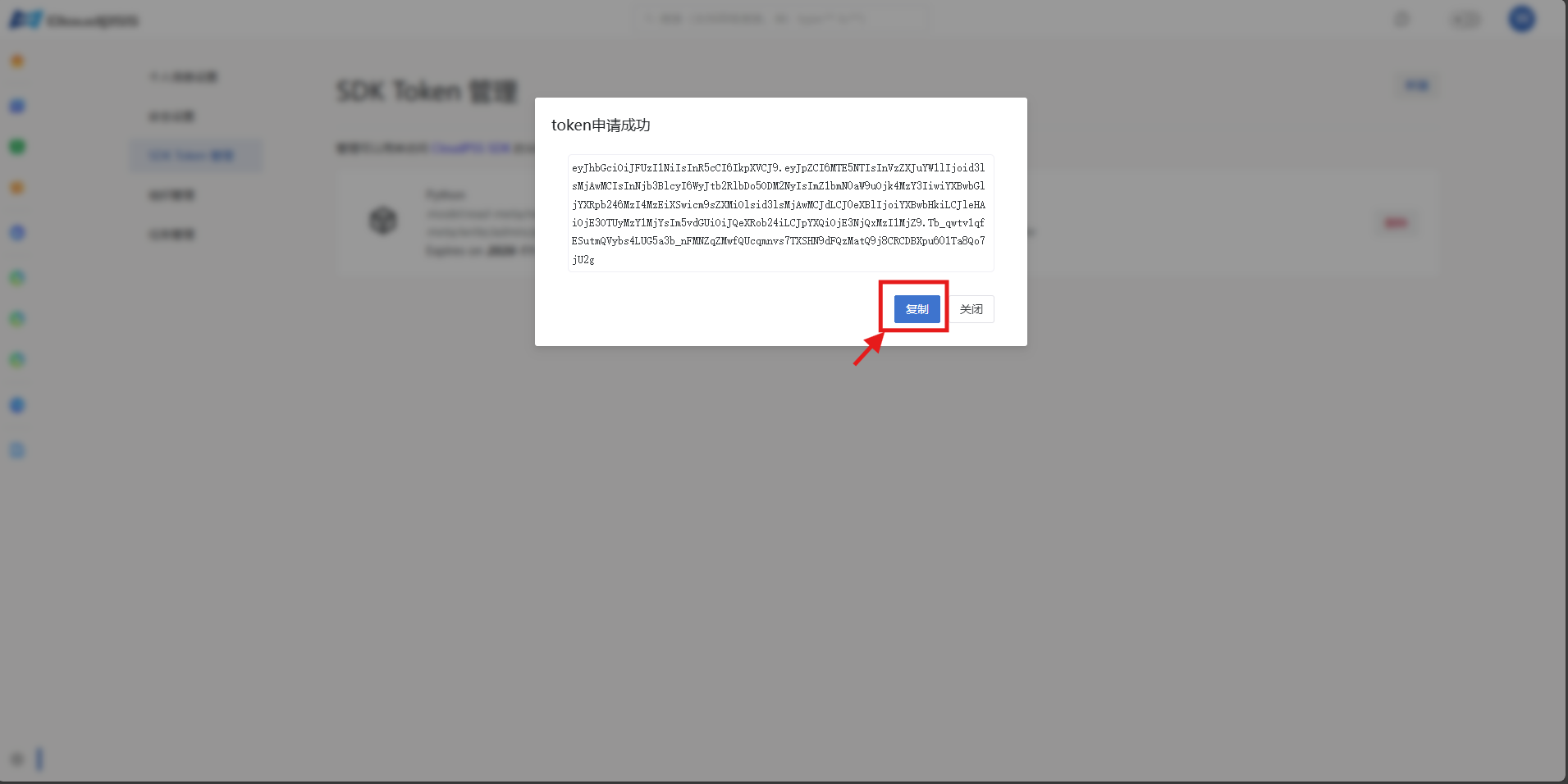


1. Copy the generated SDK code into the provided script:
   **Step1_data_generation.py**

This allows the Python script to:

- Invoke the uploaded substation model
- Perform **batch fault simulations**
- Automatically generate training or analysis samples


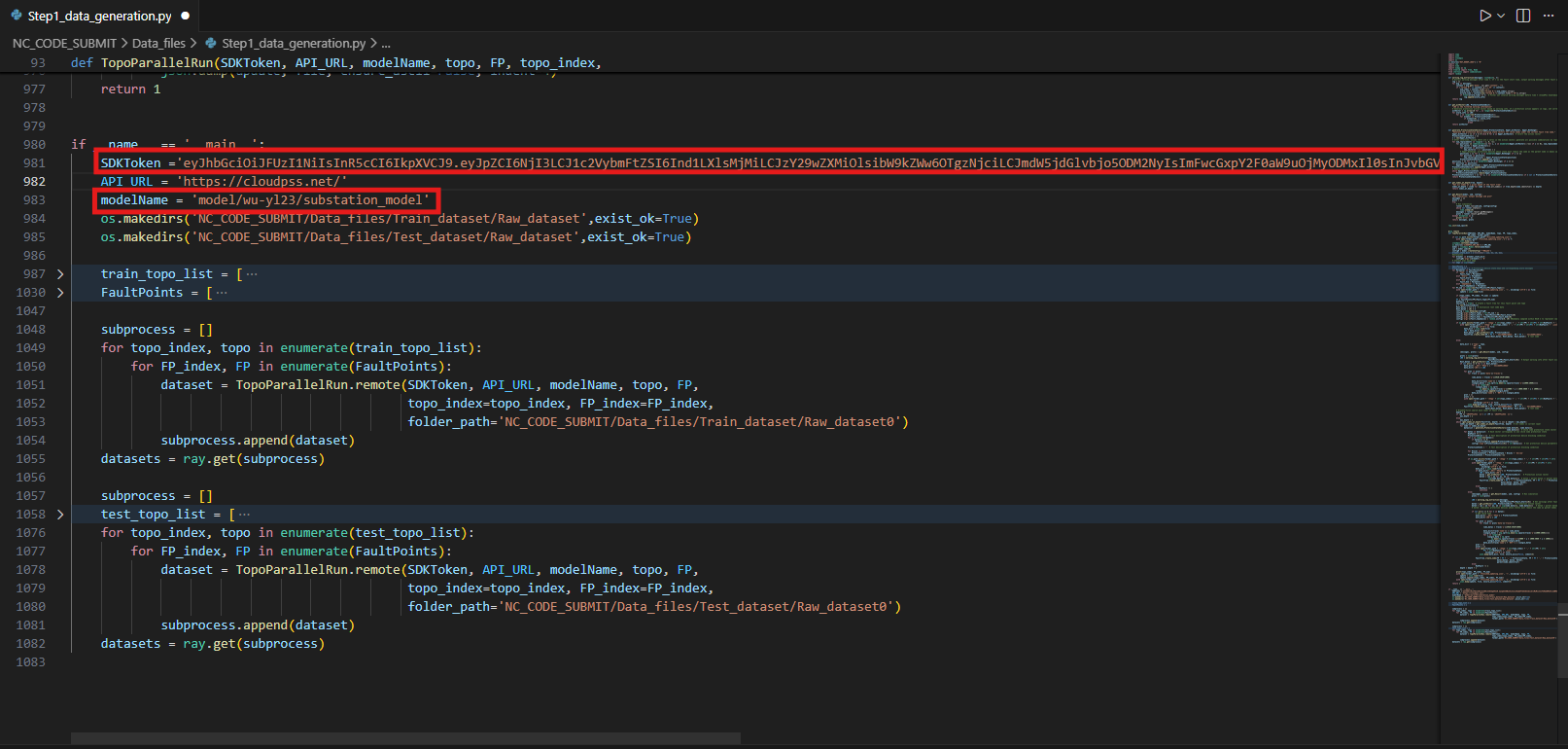


1. **Monitor Simulation Tasks**

During batch processing, you may check progress in the **Task Manager**:

- View running / completed tasks
- Examine logs
- Download results


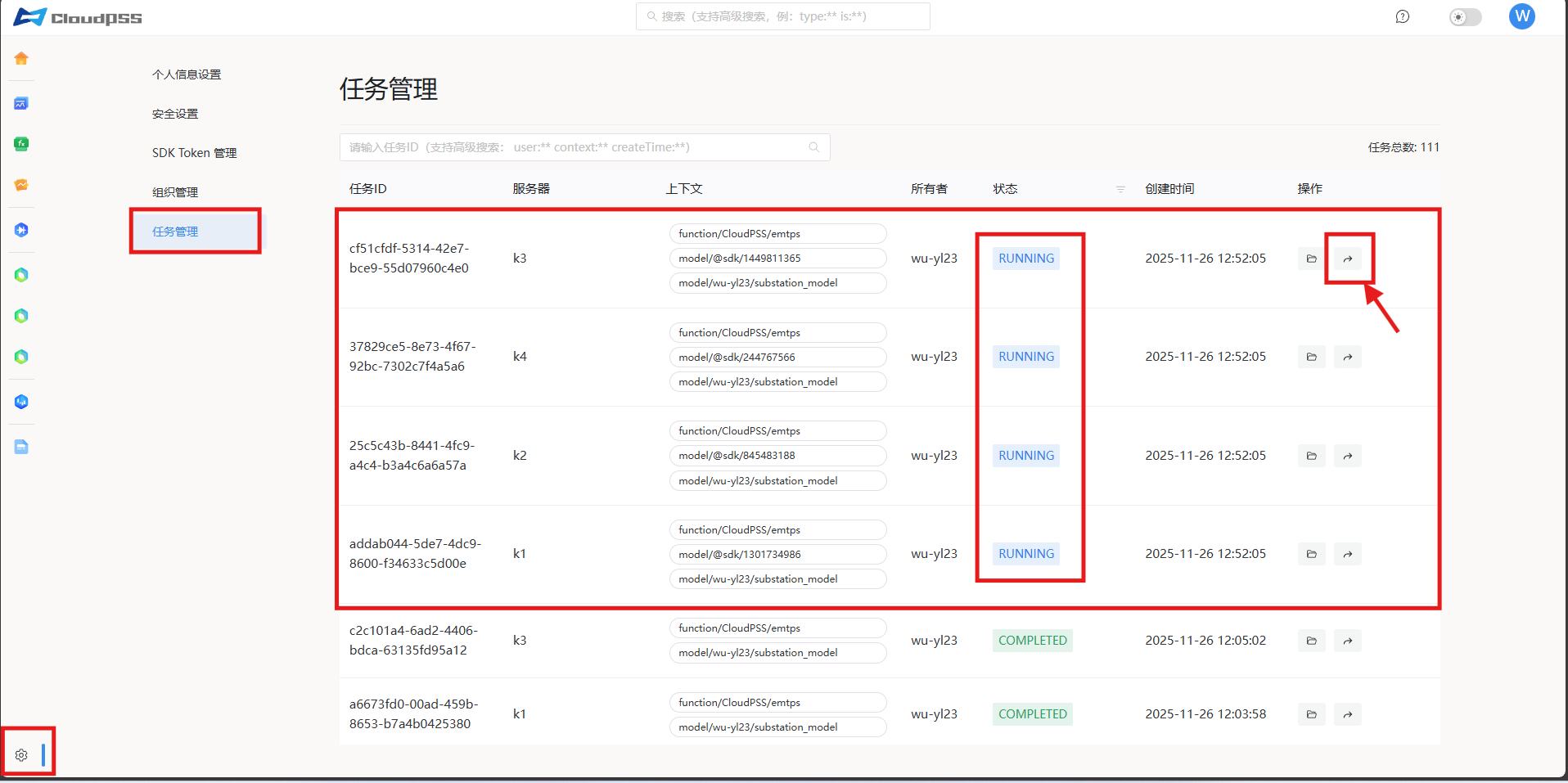


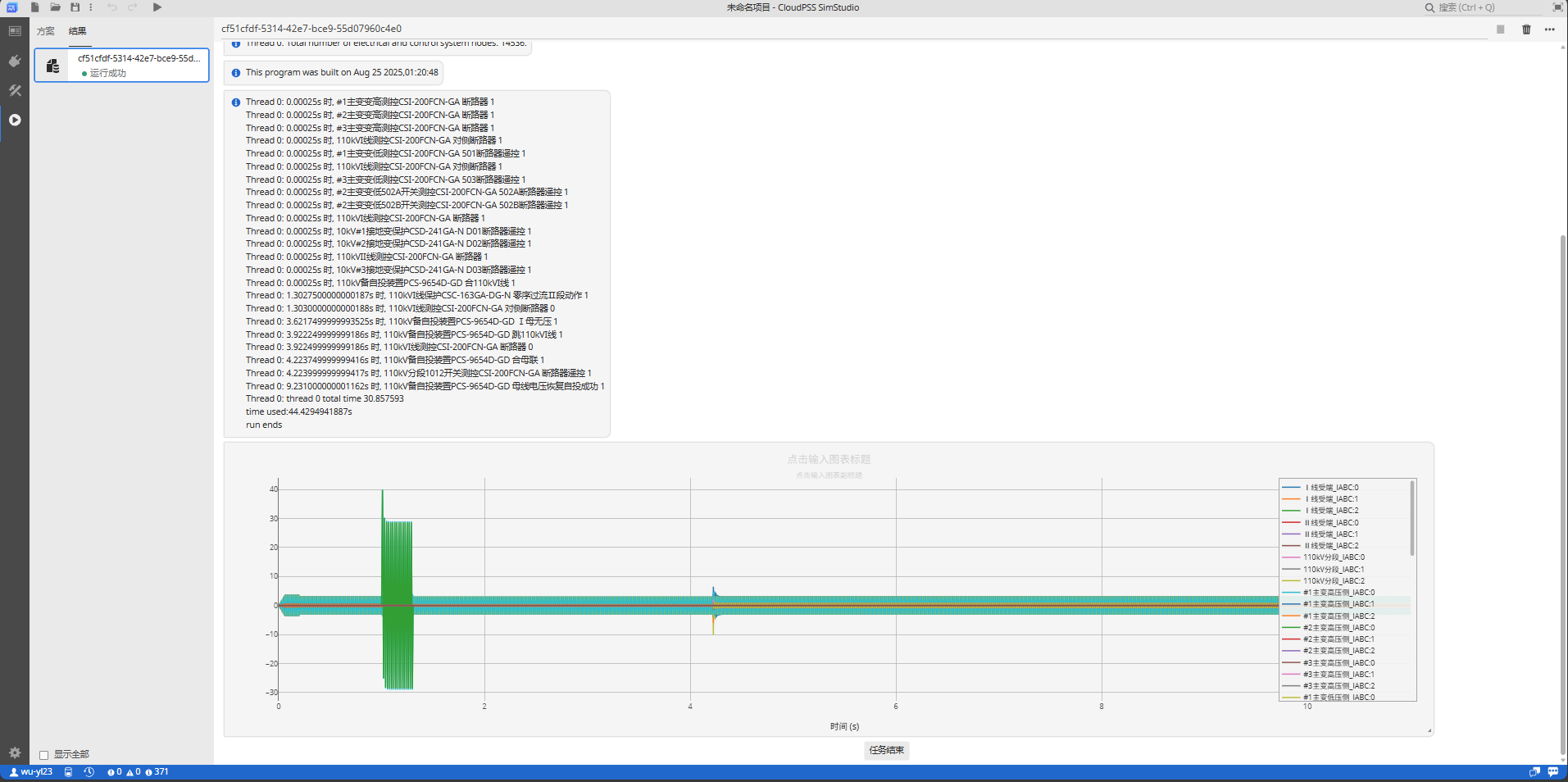

Supplement: Supplementary file 4 — Supplementary Software [file 41467_2026_73483_MOESM4_ESM.zip › Supplementary Software/Instructions for CloudPSS model.docx]
