# Supplementary material for: Digital twin-driven fault diagnosis of power substations by multi-modal fusion learning
Source: Nat Commun. 2026 May 20;17:6628. doi: 10.1038/s41467-026-73483-5 (PMC13381694; doi:10.1038/s41467-026-73483-5)
Supplement: Supplementary file 5 — Transparent Peer Review file [file 41467_2026_73483_MOESM5_ESM.pdf]

nature portfolio

Peer Review File

Digital twin-driven fault diagnosis of power substations by multi-modal fusion learning

Corresponding Author: Professor Ying Chen

This file contains all reviewer reports in order by version, followed by all author rebuttals in order by version.

Version 0:

Reviewer comments:

Reviewer #1

(Remarks to the Author)
1. Typically, dependance on the voltage level, the measurements and grid codes of a substation will be different. What is the voltag level focus of the work?
2. Line 156, what is the minimum requirements for sampling frequency of current waveform?
3. What are the features neccesary for the work? only the current waveform? therefore, typically by applying a signal processing tool, we could provide more features, concluding more accurate resutls.
4. How is the comparison of work with existing methods?
6. The operation time/delay should be highlighted for different scanrios.

(Remarks on code availability)

Reviewer #2

(Remarks to the Author)
The manuscript presents a novel digital twin-driven, multi-modal fusion model for fault diagnosis in substations, integrating GATs, self-attention, and synthetic fault generation. The results are promising, but several clarifications and revisions are needed to strengthen reproducibility and practical impact.

1. The authors report near-perfect accuracy for fault location and fault type classification in the paper. They need to clarify how much these improvements exceed transformer-based baselines (Refs. 25–27)?

2. In the abstract the authors claim “high diagnostic accuracy” and say the method has been “implemented in actual engineering applications”. Please quantify (train/test sample sizes, number of events/substations, hardware, end-to-end latency, and false-alarm/missed-alarm rates).

3. The authors need to add numerical benchmarks from literature (e.g., Bayesian networks, Petri nets, GCNs) to highlight the scale of their improvement?

4. On p. 2 - line 51, the authors mention digital twins generating diverse fault samples. They need to specify how many distinct fault types and variations were generated, and how they validated their realism?

5. Page 3: In the network description, specify SV/GOOSE sampling rates and typical end-to-end latencies, time-sync method (e.g., PTP/IRIG-B) and accuracy, and how the station/process networks feed the topology matrix used downstream.

6. On page 5, clarify how many topologies were required before generalization performance saturated, and how the authors avoided over-fitting to specific topologies?

7. Page 4: In Eq. (1): clarify whether outputs are probability-calibrated (e.g., temperature scaling), and state decision thresholds for $\hat{Y}3$ (multi-label).

8. On page 7, the authors selected RMS values for waveform representation. Please justify why this was preferred over other features like wavelets, and whether the architecture could adapt to other inputs without redesign?

9. On p. 9 - 4.2.1, the authors describe DAE pretraining. It'll be great to provide readers with the computational cost (epochs, runtime per epoch, GPU/CPU specs) if available?

10. Page 10 - Eq. (10): did the authors tune the τ temperature parameter, and if so what was its effect on performance?

11. Page 10: For protection failure classification (i.e., Eq. 12), what was the class distribution between failure and non-failure events, and how did the authors address imbalance?

12. Page 13, Line 300: Table 2 lists parameters, but why batch size 16 was chosen, whether larger sizes were tested, and the number of samples used for training vs. validation/testing?

13. Table 3 reports accuracy values. It'll be great to also provide mean ± standard deviation over multiple seeds to demonstrate stability?

14. Figure 13: Could authors quantify the additional computational cost of each module (DAE, self-attention, SCL) compared to a CNN baseline?

15. Figure 14 shows t-SNE visualizations. For better clarification, could authors supplement this with quantitative clustering metrics such as silhouette score or inter/intra-class distance ratios?

16. On p. 16, under the CloudPSS deployment, what is the communication latency between real-time data acquisition and the digital twin? please clarify.

17. In the real case study (page 17), please describe in a paragraph any situations where the model misdiagnosed or required operator correction, and how uncertainty is flagged to operators when confidence is low?

18. On page 18 - line 389: the authors emphasize robustness to missing data. Please specify the maximum tolerable missing data percentage before performance becomes unreliable in real deployments?

19. On page 18 - line 388: the authors mention integrating environmental and visual data. Explain better how these would be fused into the existing GAT/self-attention framework—would additional pretraining be required, and what data sources do you envision?

20. Under Appendix, the BFS-based fault enumeration algorithm is outlined, but providing a sample implementation would be ideal so readers could replicate author's training data generation process.

(Remarks on code availability)

Reviewer #3

(Remarks to the Author)
The manuscript with title “Digital twin-driven fault diagnosis of power components within substations by multi-modal fusion learning” presents a Graph Attention Network with self-attention to model power substations. The model uses alarm information, fault waveform recordings, and SCADA measurement data to mainly classify fault location, fault type and protection failure (ref Fig 8). A case study based on 110kV substation located in Shenzhen, China is carried out. In section 6.2, an ablation study was carried out for data loss. In general, the work is interesting and brings practical contributions.
Some comments and questions that arise:
1. The introduction/literature review on digital twins relating to substations requires more explanation and linkage towards this manuscript. There are good number of literature in the field which could be easily found.
2. Referring to Fig 4., the multi-modal fusion applies a direct mid-level fusion on the feature space (Z) of three GATs. The fusion approach seems very straightforward, has other data fusion strategy been explored? What is the rationale on selecting this option as oppose to other data fusion strategy?
3. In section 6.2, the comparison between the proposed method and the selected alternative method is limited. It is still lacking on more comparisons, for example, authors can consider deep learning techniques based on LSTM architecture, residual network etc.
4. Fair selection of alternative model hyperparameters: The selection of hyperparameters in the CNN layer is also not mentioned for study, which could significantly affect the performance. Such hyperparameter exploration will also be significant for other alternative models.
5. In figure 14, author performed t-SNE on data relating to the model. The classes are labelled from 0-15 on the top figures, and 0-9 on the bottom figures, however I do not find explanation of such classes. This needs to be properly explained.
6. Relating to the previous point, t-SNE is a dimensionality reduction technique that is stochastic and relatively difficult to interpret (see Wattenberg et al. below). In manuscript line 345, the author interpreted the separation of clusters within T-SNE. This is not quite appropriate for T-SNE, as distances between clusters might not mean anything.
Wattenberg, M., Viégas, F. and Johnson, I., 2016. How to use t-SNE effectively. Distill, 1(10), p.e2.

7. In the Table 4, the alarm information seems supervised. What would happen if there were a fault that was not within the database or existing distributions?
8. Relating to real-world application, most of the time, when data are missing in substations, they are rarely 10% or 20% missing, but fully missing due to sensor damages. Does the algorithm proposed deals with some variables being 100% missing? Perhaps authors can also justify the real-world potential relating to the ablation study of 10-50% missing data ratio. What happens when there are higher missing data ratio?
9. In Figure 12, it might be useful to label the nodes in the topology to demonstrate what these nodes represent.
10. Although the model is of a general deep learning framework, its deployability to other substations/case studies will still require effort. I do think authors should discuss such aspects together with further potential for improvement or wider application.

(Remarks on code availability)
The code was reproducible on codeocean. There is a README document that explains the code.

Reviewer #4

(Remarks to the Author)
The paper proposes a novel fault diagnosis framework that integrates digital twin technology with graph attention networks and self-attention mechanisms to enhance diagnostic accuracy and robustness under diverse conditions. The detailed reviewer comments are provided below.
- The literature review is informative but somewhat broad, as it mainly covers general fault diagnosis methods in power systems. It would be stronger if the review focused more specifically on fault detection and diagnosis in substations. For example, Jiang et al. (2024) proposed a busbar fault diagnosis method based on multi-source fusion, which is directly relevant to the current study but not cited. Including such works would better position the contribution of this paper within the state-of-the-art.
[A] Jiang X, Cao H, Zhou C, Ren X, Shen J and Yu J (2024) Busbar fault diagnosis method based on multi-source information fusion. Front. Energy Res. 12:1443570. doi: 10.3389/fenrg.2024.1443570.
- The evaluation is restricted to a single 110 kV substation. Broader validation across substations with different voltage levels, configurations, and operational conditions would strengthen claims of generalization.
- The paper addresses robustness against missing data but does not explicitly analyze the effect of measurement noise on diagnostic accuracy. Considering that measurement noise is inevitable in SCADA systems and waveform recorders, an explicit evaluation of noise robustness would strengthen the practical relevance of the results.
- While the paper provides a systematic strategy for training data generation through topology selection, fault enumeration, and data augmentation, the description of the simulated fault scenarios remains somewhat vague. It is not explicitly stated which fault types (e.g., single-line-to-ground, line-to-line, three-phase faults) or fault resistances were considered. Since the realism and diversity of training data critically affect generalization in practice, a clearer specification of fault categories, resistance ranges, and operating conditions would significantly strengthen the methodology.
- The combined use of GAT, DAE, Self-Attention, and SCL likely imposes a significant computational burden. The paper would benefit from a more detailed analysis of complexity, training time, hardware requirements, and feasibility for real-time deployment.
- The evaluation relies mainly on accuracy metrics. Adding precision, recall, F1-score, … would provide a more comprehensive understanding of model performance.
- The scope of the study is focused on diagnosing faults within substations, addressing fault location, type, and protection failures. Can external faults also generate alarms, waveform disturbances, and SCADA events in substations, resulting in mal-operation of the proposed scheme?

(Remarks on code availability)

Reviewer #5

(Remarks to the Author)
Summary:

The paper proposes a deep learning framework based on graph neural networks for the design of digital twin for fault diagnosis. Specifically graph attention networks (GAT) are utilized with incorporation of a multi-modal fusion strategy. The multi-modal fusion strategy allows to incorporate various data sources such as alarms, fault waveform recordings and supervisory control and data acquisition measurements.

Overall assessment:
The proposed approach is interesting in the context of a new application, but the methodological novelties and their added value compared to existing methods are not completely clear. Experimental setup and the description of the data are too limited to fully see potential benefit of the work.

Major comments:
- It is not completely clear how novel the proposed framework is in the context of graph attention networks and multi-modal

fusion. Different pipelines already exist in different fields and a more thorough review/clear novelty in terms of methodological contributions should be described.
- It is not clear what are the added benefits of attention features extractors. (Figure 6). Why can these not be simply replaced by MLPs? This would make the model simpler. Experiments to justify this block of the model are needed.
- It is not clear whether there is no edge information in the graphs and if not why not. At least this seems to be the case in the way the models are described.
- The correlation feature analysis should be provided to understand the structure of the data better. Do we really need to have separate blocks for attention based features extraction, even if the features are highly correlated?
- The baseline methods in the experiments are not well chosen, the proposed method already contains TCFPN in it, so it is not very surprising that the proposed method outperforms it. More conventional baselines are needed for proper assessment. This comparison should also include computational training and inference time.
- The data should be described in more detail with a table with summary statistics. In particular, how balanced is the data set, how diverse are the topologies in terms of structure and size. What are summary statistics of the features.
- Potential overfitting should be explored and mitigated. At what point would the model start overfitting?

Minor:
- All figures should be self contained. Currently the captions are very minimal.

(Remarks on code availability)
At this point I have not reviewed the code since the paper still requires quite some work in my opinion.

Version 1:

Reviewer comments:

Reviewer #1

(Remarks to the Author)
No further comment,

(Remarks on code availability)

Reviewer #2

(Remarks to the Author)
The manuscript presents several noteworthy results that advance understanding in the field, particularly through its key finding/method/result, which appears to extend existing work in a meaningful way.
Overall, the study has the potential to be significant both to the core field and to related disciplines, and it compares favorably with established literature, although clearer positioning relative to prior studies would strengthen the contribution.
The data largely support the authors' main conclusions; however, in some cases the claims appear broader than what is directly evidenced, and additional justification or clarification would be beneficial. The methodology is generally sound and consistent with current standards in the field, though certain aspects of the data analysis and interpretation would benefit from further explanation to rule out alternative interpretations. Importantly, while the methods are mostly well described, additional detail in specific steps would improve reproducibility. Overall, the revised revision is solid.

(Remarks on code availability)

Reviewer #3

(Remarks to the Author)
Authors have amended the manuscript with good effort. Nevertheless, there are still multiple aspects which needs more attention. My comments can be found below:
1. Although I appreciate that authors are providing some key results in the abstract, I find it too specific at the moment even to include inference time and GPU model. The abstract needs to be critically re-written to better reflect the work.
2. From the newly amended manuscript, I do not understand the motivation behind adding zero-mean Gaussian noise with specific signal-to-noise ratio. Also, looking at the performance results, it seems that zero-mean Gaussian noise does not do too much. It would be preferred if more irregular noises would be used as a study. Perhaps authors can justify.
3. Regarding Figure 1, it is appreciated that there are now numbers on the nodes in fig 1b. However, are these numbers corresponding to fig 1a? In this case, it would be best to also number figure 1a.
4. Regarding the accuracy in Fig 1d,e,f, I find it curious that accuracy due to sensor fail are in some case better than the accuracy of sample loss. This is not natural, could authors explain this?
5. There are a lot of problematic symbols in the pdf in Eq 3, 4 and their explanation. Please fix it.
6. Fig 4h also has some problem in the symbols. Please look into this.
7. Authors gave some very reasonable explanation on the rationale on the model architecture and data fusion approach, however this is not highlighted in the model training strategy section (and sections below). I would appreciate if authors highlight the rationale on the model training.

(Remarks on code availability)
The code is available on oceancode. It has been improved for clarity according to the revision.

Reviewer #4

(Remarks to the Author)
My comments were properly addressed.

(Remarks on code availability)

Reviewer #5

(Remarks to the Author)
The manuscript addressed some of the raised questions and concerns. However, some questions remain.

1) Related to Q3.

The authors' response mentions that edge connectivity is indeed used to define the graph structure via an adjacency matrix derived from switching states. However, this aspect is standard and essentially implicit in any GNN-based model: a graph neural network necessarily operates on an adjacency structure, and representing electrical connectivity through edges does not in itself constitute additional or novel edge information.

More importantly, the response does not fully address the fairness of the comparative evaluation. If topology and edge connectivity are central to fault propagation and protection-zone modelling, as the authors correctly argue, then comparing a topology-aware GAT against baselines (MLP, LSTM, Transformer) that do not have access to this structural information in the form of edge features is inherently unbalanced. The observed performance degradation of these non-graph models under unseen topologies is therefore expected and cannot be attributed solely to modelling limitations, but rather to the absence of critical topological input.

While the inclusion of graph-based baselines such as GCN and MPNN is appropriate and more informative, the manuscript should more clearly distinguish between:
- Performance gains due to explicit access to graph topology, and
- Performance gains due to the adaptive attention mechanism of GAT.

2) Related to Q5.

The expanded set of baselines improves the breadth of the experimental evaluation. However, the revised results raise an important issue that is currently not sufficiently analysed or discussed in the manuscript.

Notably, the ResNet-based baseline achieves performance very close to that of the proposed method across multiple diagnostic tasks, while exhibiting substantially lower computational requirements. Based on the reported results, the ResNet-based model demonstrates:
- Comparable fault localisation and type classification accuracy,
- Approximately half the inference time,
- Lower training time, and
- Reduced GPU memory usage.

This observation is particularly important in the context of real-time substation fault diagnosis and protection applications, where inference latency, computational efficiency, and system reliability are often as critical as marginal accuracy improvements. In such operational settings, faster and more resource-efficient models may be preferable if their performance is comparable.

The manuscript primarily emphasises accuracy gains, without addressing this accuracy–efficiency trade-off. A more critical discussion is needed to clarify:
- Under which operating conditions or fault scenarios does the proposed method provide clear and practically meaningful advantages over simpler architectures such as ResNet, and
- Whether the additional architectural complexity and computational cost of the proposed method are justified for real-time or near–real-time deployment in substations.

The authors are encouraged to explicitly discuss these trade-offs, particularly in the context of real-world deployment constraints. Without such analysis, the results may suggest that a simpler and more computationally efficient model could offer a more favourable balance between performance and practicality for operational use.

(Remarks on code availability)

Version 2:

Reviewer comments:

Reviewer #3

(Remarks to the Author)
My comments were addressed. I have no further remarks. Thank you.

(Remarks on code availability)

Reviewer #5

(Remarks to the Author)
No additional comments.

(Remarks on code availability)
I have no additional comments. Some analysis could have been more in depth, but the restructuring of the manuscript and some clarifications helped significantly as well.

Open Access This Peer Review File is licensed under a Creative Commons Attribution 4.0 International License, which permits use, sharing, adaptation, distribution and reproduction in any medium or format, as long as you give appropriate credit to the original author(s) and the source, provide a link to the Creative Commons license, and indicate if changes were made.
In cases where reviewers are anonymous, credit should be given to 'Anonymous Referee' and the source.
The images or other third party material in this Peer Review File are included in the article’s Creative Commons license, unless indicated otherwise in a credit line to the material. If material is not included in the article’s Creative Commons license and your intended use is not permitted by statutory regulation or exceeds the permitted use, you will need to obtain permission directly from the copyright holder.
To view a copy of this license, visit https://creativecommons.org/licenses/by/4.0/

Catalogs

RESPONSES TO REVIEWERS AND REVISIONS BASED ON THE COMMENTS 3
Response to Reviewer #1 3
Q1. Voltage level focus of the study. 3
Q2. Minimum sampling frequency requirement for current waveform. 3
Q3. Feature selection and waveform representation strategy. 4
Q4. Comparison with existing fault diagnosis methods. 6
Q5. Operation time and delay evaluation for different scenarios. 10
Response to Reviewer #2 12
Q1. Reported accuracy improvements over transformer-based baselines. 12
Q2. Quantitative clarification of system implementation and performance metrics. 12
Q3. Inclusion of numerical benchmarks from literature 14
Q4. Number and validation of generated fault types and variations 15
Q5. Communication configuration and sampling rate specifications 19
Q6. Model generalization across topologies and overfitting prevention. 21
Q7. Output probability calibration and decision thresholds. 25
Q8. Justification of RMS representation and adaptability to other features 25
Q9. Computational cost of DAE pretraining 26
Q10. Effect of temperature parameter τ in contrastive loss 27
Q11. Class imbalance handling in protection failure classification 28
Q12. Justification of batch size and dataset split details 28
Q13. Model stability over multiple random seeds 30
Q14. Computational overhead of each architectural module 31
Q15. Quantitative t-SNE clustering metrics 33
Q16. Communication latency in CloudPSS deployment 34
Q17. Operator intervention and uncertainty handling in low-confidence cases 36
Q18. Robustness threshold under missing data conditions 37
Q19. Integration of visual data and fusion within GAT/self-attention framework 39
Q20. Implementation details of the BFS-based fault enumeration algorithm 40
Response to Reviewer #3 44
Q1. Strengthening literature review on digital twins for substations 44
Q2. Rationale for mid-level feature fusion strategy 45
Q3. Expansion of comparative analysis with additional deep learning baselines 46
Q4. Fair hyperparameter selection across models 48
Q5. Explanation of class labels in t-SNE visualization 49
Q6. Proper interpretation and limitations of t-SNE plots 50
Q7. Generalization to unseen or out-of-distribution fault events 52
Q8. Handling of fully missing variables and high missing-data ratios 53
Q9. Node labeling in topology visualization 55
Q10. Discussion of deployability and transferability to other substations 56
Q11. Remarks on code availability and reproducibility 57
Response to Reviewer #4 58
Q1. Literature review needs more focus on substation fault diagnosis studies 58

Q2. Validation limited to one 110 kV substation; broader evaluation suggested 59
Q3. Missing analysis of robustness to measurement noise 60
Q4. Insufficient details on simulated fault types, resistances, and conditions 61
Q5. Computational complexity and feasibility for real-time deployment........................64
Q6. Lack of additional metrics such as precision, recall, and F1-score. 67
Q7. Influence of external faults on alarms and diagnosis results 68
Response to Reviewer #5 69
Q1. Need for clearer description of framework novelty within GAT fusion context. 69
Q2. Justification for using attention-based feature extractors instead of MLPs..............71
Q3. Clarification on edge information usage in graph representations 74
Q4. Correlation analysis to justify separate attention feature extraction blocks. 75
Q5. Selection of appropriate baselines and computational cost comparison. 76
Q6. Need for detailed dataset description and statistical summaries. 79
Q7. Discussion of potential overfitting and mitigation strategies. 82
Q8. Captions of figures are too brief; need to be self-contained....................................83
Q9. Remarks on code availability and documentation completeness. 88

RESPONSES TO REVIEWERS AND REVISIONS BASED ON THE COMMENTS

Response to Reviewer #1

Q1. Typically, dependance on the voltage level, the measurements and grid codes of a substation will be different. What is the voltage level focus of the work?

A1. We sincerely thank the reviewer for this thoughtful comment. The reviewer asked about the voltage level focus of the work and whether the proposed method is specific to a particular substation voltage grade.

In this study, the engineering validation and case analysis are performed on a 110 kV smart substation, which serves as a representative real-world application scenario. However, the proposed digital twin-driven fault diagnosis framework is not limited to a specific voltage level. Because it is developed based on the IEC61850 communication standard and a modular data-fusion architecture, it can be directly applied to other smart substations—such as 220 kV, 500 kV substations—that follow the IEC61850 communication standard.

To clarify this point, we have added the following statement in the ***Results - Case setup*** section:

The corresponding revisions in the Manuscript:

Results (Line 118-121)

Case setup

The case study and engineering validation are based on a real 110 kV smart substation located in Shenzhen, China (Fig. 1a), which operates in full compliance with the IEC 61850 standard (Supplementary Notes). The proposed method, however, is equally applicable to substations at other voltage levels (e.g., 220 kV, 500 kV) that adopt the IEC 61850 communication standards.

……

Q2. Line 156, what is the minimum requirements for sampling frequency of current waveform?

A2. We sincerely thank the reviewer for this insightful question regarding the sampling frequency requirement of fault waveform recordings. The reviewer is correct that sampling frequency may influence the fidelity of transient features, and we are happy to clarify this point.

In the proposed method, the model does not impose strict constraints on the sampling frequency. This is because the waveform features used in our framework are computed as the root-mean-square (RMS) values of the three-phase and zero-sequence currents over a fixed window consisting of 5 pre-fault cycles and 20 post-fault cycles. Such features are relatively insensitive to sampling rate variations, provided that the sampling resolution is sufficient to capture the fundamental transient behavior during faults.

In practical engineering settings, IEC 61850-compliant smart substations generally require the fault recorder's analog sampling rate to be no less than 4 kHz during high-speed recording. Following this requirement, the sampling frequency used in our 110 kV substation case study is set to 4 kHz, which reflects the actual deployment configuration and ensures realistic waveform representation.

To clarify this point, we have added the following statement in the ***Results* - *Case setup*** section:

The corresponding revisions in the Manuscript:

Results (Line 141–148)

Case setup

……

…… Fault waveform recordings capture the same current quantities at a sampling rate of 4 kHz, which is consistent with the requirements for high-speed fault recording in intelligent substations. Each waveform record contains 5 pre-fault cycles (0.1 s) and 20 post-fault cycles (0.4 s), yielding 2,000 data points per channel. It is worth noting that the proposed method does not impose strict requirements on the sampling frequency of waveform recordings, as the model extracts RMS features over multi-cycle windows. As long as the sampling rate is sufficient to capture the transient characteristics during fault events, the diagnostic performance of the model remains unaffected.

……

Q3. What are the features necessary for the work? only the current waveform? therefore, typically by applying a signal processing tool, we could provide more features, concluding more accurate results.

A3. We sincerely thank the reviewer for this insightful comment. The question addresses the essential issue of what features are required for the proposed method and whether waveform-only features would suffice for fault diagnosis.

We agree with the reviewer that advanced signal processing—such as wavelet transforms—can extract richer features from waveform recordings. However, even with such enhancements, waveform-only methods cannot replace the contextual information from alarms or the system-level measurements from SCADA, particularly for diagnosing secondary-system protection failures.

The proposed framework intentionally integrates three complementary data modalities—alarm information, fault waveform recordings, and SCADA measurements—because each captures a different aspect of the fault mechanism.

First, alarm information reflects the logical and temporal behavior of the secondary protection system. However, many different fault scenarios can trigger similar alarm sequences, especially when the same protection devices respond to multiple fault types or locations. Thus, alarm data alone often lacks discriminative power.

Second, fault waveform recordings accurately capture fast transient responses of the primary system and are highly effective for locating faults and determining fault types. Nonetheless, waveform data provide limited visibility into secondary system abnormalities, such as protection malfunctions or misoperations, which do not always manifest as distinct transient signatures.

Third, SCADA measurements offer steady-state or quasi-steady-state current information before and after the fault. While they help characterize overall system behavior and support topology-related reasoning, they lack the temporal resolution required to capture fault-induced transients and cannot determine fault types on their own.

These three data sources are therefore mutually complementary:

- alarms → secondary protection behavior
- waveforms → primary fault transients
- SCADA → network-level operating conditions

Together, they provide a holistic view of both primary and secondary system dynamics. This multi-modal integration also improves fault tolerance, as missing or degraded data from one modality can be compensated by information from the others.

As demonstrated in the ***Results - Ablation study***, removing any of the three data modalities leads to a noticeable decrease in diagnostic accuracy, underscoring that each modality contributes uniquely to the model's performance.

The corresponding part in the Manuscript:

Results (Line 262–276)

Ablation study

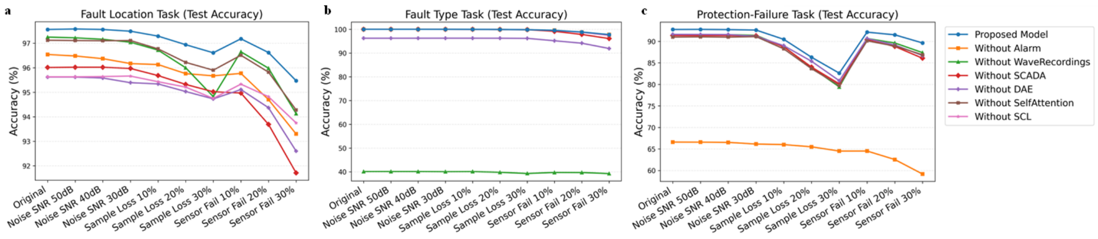

……

The results demonstrate that all data sources contribute positively to diagnostic performance across different tasks, enhancing the model's overall accuracy and robustness (Fig. 2a–c). For fault location, the diagnostic process relies on a more comprehensive integration of heterogeneous information sources. Removing any single data source leads to a noticeable decrease in accuracy (Fig. 2a). This indicates that accurate localization requires both the transient electrical characteristics captured by waveform data and the complementary contextual information provided by alarm and SCADA signals. For fault type diagnosis, waveform recordings remain the most influential factor. Excluding waveform data results in a dramatic performance reduction (Fig. 2b), reflecting that transient fault waveforms contain the most discriminative temporal–spatial signatures for distinguishing fault types. In contrast, removing alarm information or SCADA

measurements produces only minor accuracy changes (Fig. 2b), suggesting that these sources have a limited direct impact on identifying fault type. For protection failure diagnosis, alarm information proves to be the dominant source, as removing it causes a significant degradation in accuracy (Fig. 2c). This highlights that alarm signals, which reflect the operational status and timing behavior of the secondary protection system, are essential for accurately identifying malfunctioning protection devices.

……

Following the reviewer's constructive suggestion, we will explore in future work the integration of signal-processing-based feature extraction methods (e.g., wavelet or Hilbert-Huang transforms) into the waveform encoder. This enhancement will help enrich waveform representations and further improve diagnostic performance.

Q4. How is the comparison of work with existing methods?

A4. We sincerely thank the reviewer for this valuable comment regarding the comparison with existing methods. In response, we have substantially expanded both the conceptual and quantitative comparisons to clearly demonstrate the advantages of the proposed approach.

To strengthen the conceptual comparison, we added a new table (***Supplementary Table 1***) that summarizes representative rule-based and data-driven fault diagnosis methods—including expert systems, Bayesian networks, fuzzy and rough-set models, Petri nets, DS evidence-enhanced neural networks, GCN/GNN-based approaches, CNN-Transformer hybrids, and MPNN-based models. The table compares method types, input modalities, diagnostic tasks, accuracy, robustness, generalization, and model construction/update costs. This overview highlights that the proposed framework is the only one that integrates topology, alarm, SCADA, and waveform data simultaneously and that it covers all three diagnostic tasks (fault location, fault type, and protection failure) with strong robustness to noise and missing data.

To reinforce the quantitative comparison, we expanded the baseline experiments in the ***Results - Comparison of fault diagnosis models*** section. Seven baseline models were implemented, including the rule-based TCFPN model, the graph-based MPNN method, and five deep-learning baselines—MLP-based, ResNet-based, LSTM-based, GCN-based, and Transformer-based architectures. For fairness, each data-driven baseline was constructed by replacing only the GAT module in our framework while keeping all other components, data inputs, optimization settings, and model complexity comparable (details in ***Supplementary Table 5***). All models were evaluated on ten test datasets reflecting various practical disturbances such as noise, sampling loss, and sensor failures. Performance on the three diagnostic subtasks was measured using accuracy in the main text, with precision, recall, and F1 scores reported in the ***Supplementary Data***. Each experiment was repeated with five random seeds, and results are reported as mean ± standard deviation to assess stability. Across all conditions, the proposed method consistently achieves the highest performance and shows superior robustness compared with all baselines.

The corresponding revisions in the Manuscript and Supplementary Information:

Introduction (Line 89–91)

……

To highlight the conceptual advantages of the proposed approach, we provide a comparison with representative existing methods (Supplementary Table 1).

……

Results (Line 184–235)

Comparison of fault diagnosis models

……

Seven baseline models are implemented for performance comparison. Temporal constrained fuzzy Petri net (TCFPN)[12] is a rule-based approach that relies solely on alarm information to identify fault locations and protection failures. It builds upon an improved Petri Net–based framework and represents a typical rule-based solution for substation fault diagnosis. However, the model requires manual updates whenever the system topology changes. The MPNN[21] model is a data-driven method that utilizes system topology and fault waveform data to predict fault locations and types. Although originally designed for microgrid fault diagnosis, its modeling framework can be readily extended to substations and represents a state-of-the-art graph-based data-driven approach. The MLP-based, ResNet-based, LSTM-based, GCN-based, and Transformer-based models are constructed by replacing the GAT module in the proposed framework with the corresponding architectures, representing common deep learning baselines. For fair comparison, all data-driven models are trained under identical optimization settings and data inputs, and their hyperparameters and parameter counts are adjusted to maintain comparable model complexity (Supplementary Table 5,6). While the proposed model does increase the training time compared to some baselines, the overall training and inference costs remain reasonable, without introducing significant overhead relative to other deep learning models (Supplementary Table 7).

All models are evaluated on ten test datasets corresponding to different data degradation conditions, which emulate practical disturbances in communication and measurement commonly encountered in substations. For quantitative evaluation, three diagnostic subtasks are assessed independently. Accuracy is used as the primary performance metric in the main text, while additional metrics, including precision, recall, and F1-score on both training and test datasets, are provided in the supplementary material (Supplementary Data). Each experiment is repeated under five random seeds, and results are reported as mean ± standard deviation to evaluate model stability.

The proposed model consistently outperforms all baseline approaches under varying levels of noise, sampling loss, and sensor failure (Tables 1–3, Fig. 1d–f, Supplementary Fig. 3–5, Supplementary Data).

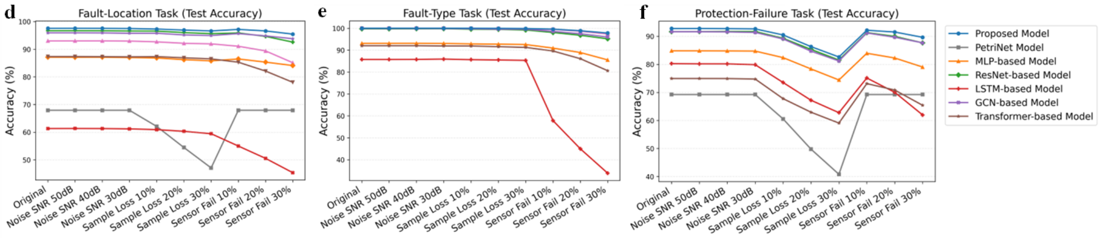

TCFPN relies solely on alarm information; however, multiple fault scenarios can produce highly similar

alarm patterns, and the rule-based inference cannot resolve these ambiguities. As a result, its outputs remain largely invariant across some different test conditions, reflecting the limited discriminative capacity of alarm-only reasoning.

MPNN utilizes system topology and fault waveform recordings, enabling competent performance in fault-location and fault-type identification. However, because it does not incorporate alarm information, it cannot diagnose protection failures. In addition, MPNN is trained in a single end-to-end manner rather than following the staged training strategy adopted in the proposed framework, resulting in less effective feature extraction and, consequently, lower diagnostic accuracy. This training scheme also limits its robustness when the input data are noisy, incomplete, or corrupted.

LSTM-based models emphasize temporal correlations and are not well-suited for capturing spatial or topological interactions, leading to reduced robustness when the input topology changes. The MLP-based and Transformer-based variants, though capable of strong representation learning when sufficient data are available, treat inputs as flattened or tokenized sequences and therefore cannot explicitly leverage the substation's graph structure. This limits their ability to learn inter-node dependencies, resulting in weaker generalization and reduced robustness under topology variations.

ResNet-based and GCN-based models partially address spatial feature learning—ResNet through 2D convolutions on processed inputs and GCN through graph convolutions. Both achieve relatively strong performance; however, their ability to capture the underlying topological relationships is limited. ResNet operates on Euclidean grids and cannot represent non-uniform electrical connectivity, while GCN performs homogeneous graph convolutions that cannot adaptively weight critical nodes. These limitations make them less effective than GAT in modeling the directional and heterogeneous dependencies among substation components. In contrast, the proposed GAT-based fusion mechanism performs adaptive, node-wise attention and integrates topology, waveform, SCADA, and alarm information more effectively, yielding the highest overall accuracy.

……

Supplementary Table 1: Comparison of existing fault diagnosis methods for power systems

Method	Type	Input Data	Main Task	Accuracy	Robustness	Generalization	Model Construction Cost	Model Update Cost
Expert Systems[1]	Rule-based	Alarm	Fault location	Moderate	Moderate-Low (sensitive to missing alarms)	Limited (rules tied to specific operation conditions)	High (manual rule-based construction)	High (frequent manual updates required)
Bayesian Networks[2]	Rule-based	Alarm	Fault location	Moderate	Moderate (probabilistic reasoning handles uncertain alarms)	Moderate (structure fixed; parameters adaptable)	Medium (requires expert-defined BN structure)	Medium (parameter tuning needed)
Fuzzy Sets[3]	Rule-based	Topology + Alarm	Fault location	Moderate-High	Moderate-High (tolerant to	Moderate (adapts to topology	Low-Medium (simple fuzzy	Medium (rules easy to adjust)

					vague or missing alarms)	through incidence matrices)	matrices)	
Rough Set[4]	Rule-based	Alarm	Fault location + fault type classification	Moderate-High	Moderate-High (rule reduction enhances stability)	Moderate (rules discovered from data, not topology-dependent)	Medium-Low (automatic rule extraction)	Medium (compact rule-base, low maintenance)
Analytical Models[5]	Rule-based	Alarm	Fault location + protection failure detection	Moderate-High	Moderate-High (robust to redundant alarms; timestamp utilization)	Moderate (supports varied protection schemes & bus configurations)	Medium (analytic formula only)	Medium (only configuration-related adjustments)
Petri Nets[6]	Rule-based	Alarm	Fault location + protection failure detection	Moderate-High	Moderate-High (handles fuzzy evidence)	Moderate (temporal + fuzzy transitions work in many scenarios)	Medium (Petri net modeling required)	Medium (net structure updates moderately costly)
Hierarchical Diagnosis[7]	Rule-based	Alarm + SCADA	Fault location + protection failure detection	High	High (multi-source fusion mitigates conflicting information)	Good (layered use of switching + electrical info)	Medium-High (two-layer diagnostic modeling)	Medium (both layers must be updated)
DS Evidence + BP NN[8]	Data-driven	Fault waveform recordings	Busbar fault type classification	High	High (DS fusion reduces uncertainty before NN training)	Good (learns busbar patterns independent of topology)	Medium (requires DS preprocessing + NN training)	Medium (NN retraining needed for new waveform patterns)
GCNs[9]	Data-driven	Topology + SCADA	Fault location	Very High	Very High (robust to noise, data loss, topology variations)	Very good (graph learning generalizes across networks)	Medium-High (graph modeling + GNN training)	Medium (moderate retraining for topology changes)
CNN-based Transformer[10]	Data-driven	Fault waveform recordings	Fault location + fault type classification	Very High	Very High (attention mechanism resists waveform distortions)	Good (long-term temporal modeling adapts across conditions)	Medium-High (complex hybrid deep model)	Medium (retraining needed for distribution shifts)
R-GNNs[11]	Data-driven	Topology + Fault waveform recordings	Fault location + fault type classification	Very High	Very High (captures spatial-temporal dependencies reliably)	Very good (generalizes across operating modes & topologies)	Medium-High (recurrent GNN architecture)	Medium (requires updates for major topology changes)

MPNN[12]	Data-driven	Topology + Fault waveform recordings	Fault location + fault type classification	Very High	Very High (resilient to topology variations & missing data)	Very good (Graph-Lasso auto-identifies topology)	Medium-High (joint MPNN + topology estimation)	Low-Medium (auto topology update reduces manual work)
Proposed Method	Data-driven	Topology + Alarm + SCADA + Fault waveform recordings	Fault location + fault type classification + protection failure detection	Excellent	Excellent (multi-modal fusion + attention; resistant to noise, missing data)	Excellent (digital twin + graph attention adapt to topology, data quality, and real scenarios)	High (digital twin modeling + multi-modal architecture)	Low-Medium (digital twin reduces update workload)

Q5. The operation time/delay should be highlighted for different scenarios.

A5. We sincerely thank the reviewer for this helpful comment. The reviewer requested that the operation time and diagnostic delay be highlighted for different scenarios. We agree that clarifying the end-to-end latency is important for demonstrating the practical feasibility of the proposed system.

We provide a detailed analysis of the diagnostic latency of the deployed digital twin system in ***Supplementary Information*–*Supplementary Notes*–*Latency analysis of the digital twin system*.** The end-to-end delay consists of three sequential stages: (i) data acquisition, (ii) data processing, and (iii) model inference. Among these, waveform file generation inside protection IEDs/fault recorders is the dominant contributor.

In Stage 1 (data acquisition), switching states, SOE logs, and SCADA measurements are delivered to the station level within milliseconds via IEC 61850 GOOSE and MMS, contributing negligible delay. However, protection IEDs must capture pre-fault and fault-period transients before generating COMTRADE files, and this process takes approximately 50 seconds in the studied 110 kV substation. File upload via MMS adds roughly 10 seconds.

In Stage 2 (data processing), the digital twin reconstructs the topology, parses COMTRADE files, and formats all modalities into model inputs. This takes about 1 s.

In Stage 3 (model inference), the diagnostic network runs on an Intel Xeon E3-1230 v5 CPU and an NVIDIA RTX 3090 GPU, requiring approximately 0.5 s to produce the final result.

Because the SOE and SCADA streams arrive during waveform acquisition, the overall latency is dominated by waveform file generation. With waveform generation and transfer taking about 60 s in total, data preprocessing 1 s, and inference 0.5 s, the full end-to-end latency of the deployed system is approximately 60–65 s. This latency is acceptable in practice, as the proposed method is designed for post-fault diagnosis, where diagnostic accuracy and reliability are more critical than strict real-time response.

The corresponding revisions in the Manuscript:

Abstract (Line 17-18)

……

The deployed digital twin system further demonstrates feasibility, achieving about 1 min data transmission latency over IEC 61850 and 0.5 s inference time on an NVIDIA RTX 3090 GPU.

Results (Line 368-382)

Digital twin application platform

……

During operation, the station level continuously received the real-time switching states of circuit breakers, disconnectors, and grounding switches from bay-level IEDs, ensuring that the pre-fault topology was already available when the fault occurred. Following the fault, the station level immediately obtained the sequence-of-events (SOE) alarms (Supplementary Table 10) and SCADA analog measurements, both of which were transmitted within milliseconds and therefore did not contribute noticeably to the diagnostic latency. In contrast, the acquisition of fault waveforms was substantially slower. The protection IEDs and fault recorders needed to capture the pre-fault window and the complete transient before generating COMTRADE files, and this file-generation step dominated the overall delay. In the 110 kV smart substation used for validation, generating the waveform files from multiple triggered recorders required approximately 50 s, after which the files (about 20 MB each) were uploaded to the station level within roughly 10 s. Once all required waveform files were available, the digital twin system reconstructed the pre-fault topology, parsed and organized the SOE, SCADA, and waveform data, and produced the model inputs, all within about 1 s. The diagnostic model then performed inference on the workstation within approximately 0.5 s. As SOE and SCADA reporting overlap with waveform acquisition, the end-to-end diagnostic latency is dominated by the recorder-side waveform file-generation time (Supplementary Note).

Response to Reviewer #2

The manuscript presents a novel digital twin-driven, multi-modal fusion model for fault diagnosis in substations, integrating GATs, self-attention, and synthetic fault generation. The results are promising, but several clarifications and revisions are needed to strengthen reproducibility and practical impact.

Q1. The authors report near-perfect accuracy for fault location and fault type classification in the paper. They need to clarify how much these improvements exceed transformer-based baselines (Refs. 25-27)?

A1. We sincerely thank the reviewer for this insightful comment. The reviewer asked how much improvement the proposed method achieves compared with Transformer-based baselines. This is an important concern, and we have expanded our experiments to provide a clear and quantitative comparison.

To address this question, we added Transformer-based baseline models for detailed evaluation. Specifically, we constructed a Transformer-based variant of our framework by replacing the Graph Attention Network (GAT) module with a standard Transformer encoder-decoder architecture, while keeping all other components—including input features, optimization settings, hyperparameters, and parameter counts—identical. This ensures a fair and controlled comparison focused solely on the architectural difference between GAT and Transformer. In addition, we compared our model against the Message Passing Neural Network (MPNN) approach described in Ref. [21], which corresponds to the former Ref. [27] in the original submission.

The quantitative results are presented in the revised ***Results*** – ***Comparison of fault diagnosis models*** section and are detailed in ***Supplementary Data.*** These results show that the proposed method consistently outperforms the Transformer-based and MPNN baselines across all diagnostic subtasks. The GAT module more effectively captures system topology, enhances information propagation through graph-structured relationships, and strengthens multi-modal fusion, thereby improving diagnostic robustness and accuracy.

The corresponding revisions in the Manuscript:

Results (Line 184-235)

Comparison of fault diagnosis models

……

Seven baseline models are implemented for performance comparison. …… The MPNN[21] model is a data-driven method that utilizes system topology and fault waveform data to predict fault locations and types. Although originally designed for microgrid fault diagnosis, its modeling framework can be readily extended to substations and represents a state-of-the-art graph-based data-driven approach. The MLP-based, ResNet-based, LSTM-based, GCN-based, and Transformer-based models are constructed by replacing the GAT module in the proposed framework with the corresponding architectures, representing common deep learning

baselines. For fair comparison, all data-driven models are trained under identical optimization settings and data inputs, and their hyperparameters and parameter counts are adjusted to maintain comparable model complexity (Supplementary Table 5,6). While the proposed model does increase the training time compared to some baselines, the overall training and inference costs remain reasonable, without introducing significant overhead relative to other deep learning models (Supplementary Table 7).

All models are evaluated on ten test datasets corresponding to different data degradation conditions, which emulate practical disturbances in communication and measurement commonly encountered in substations. For quantitative evaluation, three diagnostic subtasks are assessed independently. Accuracy is used as the primary performance metric in the main text, while additional metrics, including precision, recall, and F1-score on both training and test datasets, are provided in the supplementary material (Supplementary Data). Each experiment is repeated under five random seeds, and results are reported as mean ± standard deviation to evaluate model stability.

The proposed model consistently outperforms all baseline approaches under varying levels of noise, sampling loss, and sensor failure (Tables 1–3, Fig. 1d–f, Supplementary Fig. 3–5, Supplementary Data).

……

MPNN utilizes system topology and fault waveform recordings, enabling competent performance in fault-location and fault-type identification. However, because it does not incorporate alarm information, it cannot diagnose protection failures. In addition, MPNN is trained in a single end-to-end manner rather than following the staged training strategy adopted in the proposed framework, resulting in less effective feature extraction and, consequently, lower diagnostic accuracy. This training scheme also limits its robustness when the input data are noisy, incomplete, or corrupted.

……and Transformer-based variants, though capable of strong representation learning when sufficient data are available, treat inputs as flattened or tokenized sequences and therefore cannot explicitly leverage the substation's graph structure. This limits their ability to learn inter-node dependencies, resulting in weaker generalization and reduced robustness under topology variations.

……

Q2. In the abstract the authors claim "high diagnostic accuracy" and say the method has been "implemented in actual engineering applications". Please quantify (train/test sample sizes, number of events/substations, hardware, end-to-end latency, and false-alarm/missed-alarm rates).

A2. We sincerely thank the reviewer for this constructive and important suggestion. The reviewer asked us to quantify the claims of "high diagnostic accuracy" and "implementation in actual engineering applications" by providing concrete information on dataset size, number of events/substations, hardware configuration, end-to-end latency, and false-alarm/missed-alarm rates.

To address this comment, we have revised the abstract and main text to include explicit quantitative descriptions of the experimental scale and deployment performance. The revised abstract now specifies that the proposed method is validated on a real 110 kV intelligent substation, with 11,597 training and 3,890 testing fault scenarios generated through the CloudPSS digital twin platform. We also report that

the model achieves 95% accuracy in fault location, 97% in fault type identification, and 90% in protection failure detection under 30% data loss.

Regarding engineering deployment, we clarify that the digital twin system is implemented on a workstation equipped with an NVIDIA RTX 3090 GPU, and that the end-to-end diagnostic latency consists of approximately 1 minute for IEC 61850 communication and file generation, and 0.5 seconds for model inference.

The corresponding revisions in the Manuscript:

Abstract (Line 14–18)

……

In this work, the method is validated on a 110 kV substation with 11,597 training and 3,890 testing scenarios generated in CloudPSS. It achieves over 95% accuracy in fault location, 97% in fault type identification, and 90% in protection failure detection under 30% data loss. The deployed digital twin system further demonstrates feasibility, achieving about 1 min data transmission latency over IEC 61850 and 0.5 s inference time on an NVIDIA RTX 3090 GPU.

Q3. The authors need to add numerical benchmarks from literature (e.g., Bayesian networks, Petri nets, GCNs) to highlight the scale of their improvement?

A3. We sincerely thank the reviewer for this valuable suggestion. The reviewer recommended adding numerical benchmarks from the literature (e.g., Bayesian networks, Petri nets, GCNs) in order to clearly highlight the performance improvement achieved by the proposed method.

To address this comment, we have significantly expanded the benchmark set to include both rule-based and data-driven methods widely reported in existing substation fault diagnosis research. This allows for a comprehensive, quantitative, and fair comparison across different methodological categories.

In particular, we added the TCFPN model, an improved Petri Net-based fault diagnosis framework, as a representative rule-based expert system. This model closely reflects the performance of Bayesian network-style approaches because both rely on structured reasoning over alarm sequences. Thus, TCFPN serves as an appropriate benchmark for traditional analytical and reasoning-based diagnostic systems.

For data-driven baselines, we further incorporated the MPNN model, which adopts a graph-based learning paradigm similar to our framework. In addition, we constructed MLP-based, ResNet-based, LSTM-based, GCN-based, and Transformer-based variants by replacing the GAT module in our approach with the corresponding architectures. All models were trained with identical datasets, training configurations, and comparable parameter scales, ensuring methodological fairness. For each model, we evaluate diagnostic accuracy, precision, recall, F1-score, training time, and inference latency.

The expanded numerical comparisons are presented in the revised ***Results* – *Comparison of fault diagnosis models*** section, with full metrics provided in the ***Supplementary Data*.** The results clearly demonstrate that the proposed method consistently outperforms all baselines across fault location, fault

type classification, and protection failure detection tasks, confirming its superior diagnostic capability and robustness under noise, missing data, and topology variations.

The corresponding revisions in the Manuscript:

Results (Line 184–235)

Comparison of fault diagnosis models

……

Seven baseline models are implemented for performance comparison. Temporal constrained fuzzy Petri net (TCFPN)[12] is a rule-based approach that relies solely on alarm information to identify fault locations and protection failures. It builds upon an improved Petri Net–based framework and represents a typical rule-based solution for substation fault diagnosis. However, the model requires manual updates whenever the system topology changes. The MPNN[21] model is a data-driven method that utilizes system topology and fault waveform data to predict fault locations and types. Although originally designed for microgrid fault diagnosis, its modeling framework can be readily extended to substations and represents a state-of-the-art graph-based data-driven approach. The MLP-based, ResNet-based, LSTM-based, GCN-based, and Transformer-based models are constructed by replacing the GAT module in the proposed framework with the corresponding architectures, representing common deep learning baselines. For fair comparison, all data-driven models are trained under identical optimization settings and data inputs, and their hyperparameters and parameter counts are adjusted to maintain comparable model complexity (Supplementary Table 5,6). While the proposed model does increase the training time compared to some baselines, the overall training and inference costs remain reasonable, without introducing significant overhead relative to other deep learning models (Supplementary Table 7).

All models are evaluated on ten test datasets corresponding to different data degradation conditions, which emulate practical disturbances in communication and measurement commonly encountered in substations. For quantitative evaluation, three diagnostic subtasks are assessed independently. Accuracy is used as the primary performance metric in the main text, while additional metrics, including precision, recall, and F1-score on both training and test datasets, are provided in the supplementary material (Supplementary Data). Each experiment is repeated under five random seeds, and results are reported as mean ± standard deviation to evaluate model stability.

The proposed model consistently outperforms all baseline approaches under varying levels of noise, sampling loss, and sensor failure (Tables 1–3, Fig. 1d–f, Supplementary Fig. 3–5, Supplementary Data).

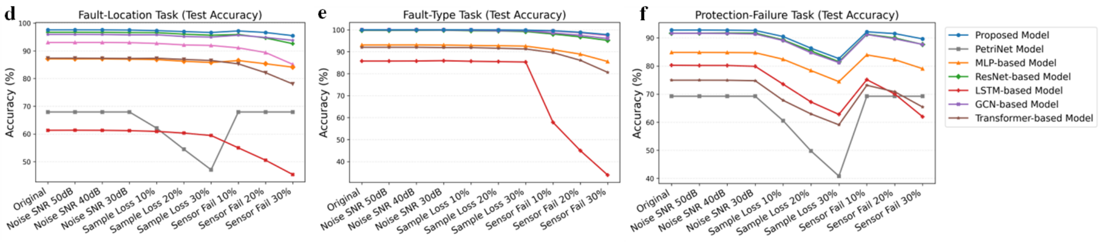

TCFPN relies solely on alarm information; however, multiple fault scenarios can produce highly similar alarm patterns, and the rule-based inference cannot resolve these ambiguities. As a result, its outputs remain largely invariant across some different test conditions, reflecting the limited discriminative capacity of alarm-

only reasoning.

MPNN utilizes system topology and fault waveform recordings, enabling competent performance in fault-location and fault-type identification. However, because it does not incorporate alarm information, it cannot diagnose protection failures. In addition, MPNN is trained in a single end-to-end manner rather than following the staged training strategy adopted in the proposed framework, resulting in less effective feature extraction and, consequently, lower diagnostic accuracy. This training scheme also limits its robustness when the input data are noisy, incomplete, or corrupted.

LSTM-based models emphasize temporal correlations and are not well-suited for capturing spatial or topological interactions, leading to reduced robustness when the input topology changes. The MLP-based and Transformer-based variants, though capable of strong representation learning when sufficient data are available, treat inputs as flattened or tokenized sequences and therefore cannot explicitly leverage the substation's graph structure. This limits their ability to learn inter-node dependencies, resulting in weaker generalization and reduced robustness under topology variations.

ResNet-based and GCN-based models partially address spatial feature learning—ResNet through 2D convolutions on processed inputs and GCN through graph convolutions. Both achieve relatively strong performance; however, their ability to capture the underlying topological relationships is limited. ResNet operates on Euclidean grids and cannot represent non-uniform electrical connectivity, while GCN performs homogeneous graph convolutions that cannot adaptively weight critical nodes. These limitations make them less effective than GAT in modeling the directional and heterogeneous dependencies among substation components. In contrast, the proposed GAT-based fusion mechanism performs adaptive, node-wise attention and integrates topology, waveform, SCADA, and alarm information more effectively, yielding the highest overall accuracy.

……

Q4. On p. 2 - line 51, the authors mention digital twins generating diverse fault samples. They need to specify how many distinct fault types and variations were generated, and how they validated their realism?

A4. We sincerely thank the reviewer for highlighting the need to clarify the diversity and realism of the fault scenarios generated in the digital-twin environment. This is indeed essential for understanding the validity and generalizability of the proposed approach. In the revised manuscript, we have expanded the description of the data-generation process, and a complete summary is now provided in ***Supplementary Table 3***.

In brief, the construction of the dataset follows a systematic simulation and augmentation pipeline, detailed in the revised ***Results - Case setup***:

- Topology variations: The 10 training topologies and 4 testing topologies correspond to different breaker/isolator configurations of the real substation, ensuring structural diversity across operating modes.
- Fault locations and types: 16 possible fault locations were modeled, each simulated under 10 fault types (A/B/C single-phase grounding, AB/BC/CA two-phase short-circuit, AB/BC/CA two-phase-to-ground short-circuit, and three-phase short-circuit).

- Fault resistance: Randomly varied within 0–10 Ω to represent low-resistance faults.
- Protection failures: To model secondary-system uncertainties, we used an automated fault-enumeration procedure based on Breadth-First Search (***Supplementary Methods***), generating 11,597 distinct training scenarios and 3,890 testing scenarios that include varied protection-failure combinations.
- Data modalities: Each scenario contains alarm data, SCADA measurements (2 Hz), and waveform recordings sampled at 4 kHz, consistent with the requirements for smart substations.

To further enhance the realism and robustness of the dataset, multiple data-augmentation strategies were applied, including zero-mean Gaussian noise (30–50 dB SNR), random sampling loss (10–30% of time-series samples), and simulated sensor failures (10–30% complete channel loss). These augmentations produced a total of 115,970 training samples and 38,900 testing samples, ensuring that the data better reflect actual operating conditions and significantly reduce the risk of model overfitting.

This dataset comprehensively covers diverse fault types, resistances, topologies, and secondary-system behaviors, thereby ensuring that the model is trained and validated on scenarios closely reflecting real-world substation conditions.

Notably, the digital-twin model is configured using primary-equipment parameters and protection settings provided by the actual engineering project, ensuring realistic system behavior. In addition, one real fault case from the same substation was used for validation, and the simulated protection actions, alarm sequences, and fault waveforms showed high consistency with the recorded measurements, confirming the realism of the generated data.

The corresponding revisions in the Manuscript:

Results (Line 129–163)

Case setup

……

To capture diverse operational conditions, ten different topologies are simulated by varying circuit breaker open/close statuses (Fig. 1b), generating the training dataset through batch fault simulations. Each topology covers all 16 fault locations and 10 fault types (A/B/C single-phase grounding, AB/BC/CA two-phase short-circuit, AB/BC/CA two-phase-to-ground short-circuit, and three-phase short-circuit). Fault resistance is randomly set within 0–10 Ω to represent low-resistance faults. Based on these combinations, 1,600 base fault scenarios are defined. To further consider secondary system uncertainties, multiple protection failure conditions are generated automatically using a fault enumeration tree traversal method based on Breadth-First Search (Supplementary Methods), resulting in 11,597 distinct fault scenarios for the training dataset.

For each fault case, alarm data are collected from PRs, including time-tagged event signals recorded during the fault process. SCADA measurements record the root-mean-square (RMS) values of three-phase and zero-sequence currents of each line via current transformers (CTs), sampled at 2 Hz, retaining 1 pre-fault and 19 post-fault data points within a 10 s time window. Fault waveform recordings capture the instantaneous values of the same current quantities at a sampling rate of 4 kHz, which is consistent with the requirements for high-speed fault recording in smart substations. Each waveform record contains 5 pre-fault cycles (0.1 s)

and 20 post-fault cycles (0.4 s), yielding 2,000 data points per channel. It is worth noting that the proposed method does not impose strict requirements on the sampling frequency of waveform recordings, as the proposed model extracts RMS features over multi-cycle windows. As long as the sampling rate is sufficient to capture the transient characteristics during fault events, the diagnostic performance of the model remains unaffected.

To enhance robustness and generalization, multiple data augmentation strategies are applied, including adding zero-mean Gaussian noise with signal-to-noise ratios (SNR) of 50, 40, and 30 dB; introducing random sampling loss by removing 10%, 20%, and 30% of time-series data points from alarm, SCADA, and waveform data; and simulating sensor failures by randomly dropping 10%, 20%, and 30% of entire SCADA or waveform channels. These augmentations generate nine additional datasets, which, together with the original data, result in a total of 115,970 training samples.

To evaluate generalization capability, four unseen topologies are selected for testing (Fig. 1c). For each topology, fault scenarios are generated using the same procedure as for the training set—including all fault locations, fault types, fault resistances, and the same protection-failure enumeration method—resulting in 3,890 distinct base fault scenarios. Using identical noise, sampling-loss, and sensor-failure augmentation strategies, 10 corresponding test datasets are constructed, forming the final test set (Supplementary Table 3).

This dataset, incorporating diverse topologies, fault locations, fault types, fault resistances, and protection failures, together with multi-level augmentation strategies, comprehensively represents the complexities and uncertainties of real-world substation operation.

……

Supplementary Table 3: Summary of the dataset

Item	Description
Topologies	10 for training and 4 for testing (different operating configurations)
Fault locations	16 (transmission lines, busbars, transformers, sectionalizers)
Fault types	10 (A/B/C single-phase grounding; AB/BC/CA two-phase short-circuit; AB/BC/CA two-phase-to-ground short-circuit; three-phase short-circuit)
Fault resistance	Randomly sampled within 0–10 Ω to represent low-resistance faults
Protection failure conditions	Automatically enumerated using a Breadth-First Search-based fault enumeration method (Supplementary Method)
Base fault scenarios	11,597 for training; 3,890 for testing
Data modalities	Alarm events, SCADA measurements (2 Hz, RMS values of three-phase and zero-sequence currents from CTs), and fault waveform recordings (4 kHz, instantaneous values of the same current quantities)
Noise levels (SNR)	30, 40, and 50 dB (zero-mean Gaussian noise)
Sampling loss	Random removal of 10%, 20%, or 30% of time-series data points from alarm, SCADA, waveform data
Sensor failures	Random dropping of 10%, 20%, or 30% of the entire SCADA or waveform channels
Total fault samples	115,970 for training; 38,900 for testing

Supplementary Figure 9: Comparison of partial real fault waveforms and simulation waveforms.

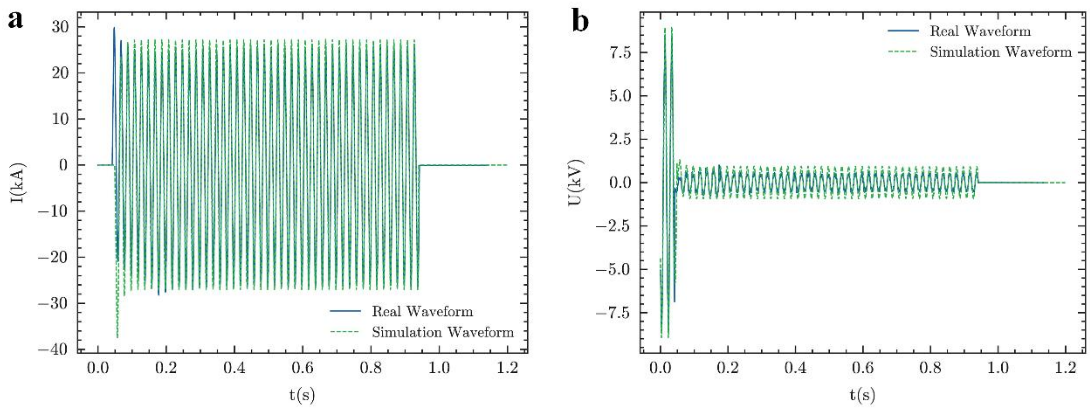

Supplementary Figure 9. Comparison of partial real fault waveforms and simulation waveforms. a Current waveform of phase A on the low-voltage side of the #3 main transformer during a three-phase short circuit occurring at the 10 kV Sectionalizer II. When the fault occurs, the current rises sharply and remains at a high magnitude until 0.9 s, when the low-voltage overcurrent protection operates and trips the low-voltage circuit breaker, causing the current to drop to zero. The green dashed line represents the simulation waveform, and the blue solid line represents the real recorded waveform. **b** Voltage waveform of phase A on the 10 kV Busbar III. Before the fault, the voltage remains within the normal operating range. During the fault, the three-phase short circuit causes the voltage to collapse sharply, leaving only residual voltage. After the protection system isolates the fault, the bus voltage drops to zero. The green dashed line represents the simulation waveform, and the blue solid line represents the real recorded waveform.

Q5. Page 3: In the network description, specify SV/GOOSE sampling rates and typical end-to-end latencies, time-sync method (e.g., PTP/IRIG-B) and accuracy, and how the station/process networks feed the topology matrix used downstream.

A5. We sincerely thank the reviewer for this valuable comment. The question highlights the need to clarify the sampling rates, communication delays, time-synchronization mechanisms, and the way in which process- and station-level networks supply real-time topology information to the downstream diagnosis model. These details are essential for understanding the digital-substation environment and the data pipeline supporting the proposed approach.

To address this, we have substantially expanded the description of the communication architecture in ***Supplementary Note – Overview of the IEC 61850 communication architecture in smart substations*.** The revised text now provides a complete explanation of IEC 61850-based communication architecture. The key information is summarized as follows.

Modern smart substations built upon the IEC 61850 standard adopt a hierarchical three-level, two-bus communication architecture spanning the process, bay, and station levels. Sampled Values (SV) are transmitted on the process bus from merging units to bay-level IEDs at typical rates of 80 samples per cycle (4 kHz) for protection applications and 256 samples per cycle (12.8 kHz) for high-fidelity measurement. The end-to-end transfer time of protection-grade SV is within 3 ms, enabling timely execution of protection algorithms. Generic Object Oriented Substation Event (GOOSE) messages are used for fast peer-to-peer transmission of protection, interlocking, and status information, with typical

end-to-end delay also within 3 ms. MMS is used primarily on the station bus for supervisory monitoring, configuration management, and retrieval of fault records.

A unified time-synchronization system ensures coherent timestamping and time alignment across all three levels of the digital substation. In typical deployments, station-level devices obtain time via the Simple Network Time Protocol (SNTP), which provides millisecond-level accuracy adequate for supervisory and other non-time-critical functions. In contrast, bay-level and process-level equipment are normally synchronized using either the Inter-Range Instrumentation Group time code B (IRIG-B) or the IEEE 1588v2 Precision Time Protocol (PTP), both of which satisfy the microsecond-level timing requirements of protection and high-precision measurement applications. In the 110 kV smart substation investigated in this study, both BeiDou Navigation Satellite System (BDS) and the Global Positioning System (GPS) time references are available, with BDS used as the primary source, and the bay and process levels employ IEEE 1588v2 PTP as the main synchronization method, achieving a timing accuracy better than 1 μs across the IEDs.

Regarding topology acquisition, the digital twin continuously receives real-time switching states of circuit breakers, disconnectors, and grounding switches via GOOSE and MMS. These switching signals are decoded to reconstruct the pre-fault network topology, forming the adjacency matrix fed directly into the GAT-based topology-aware diagnosis model. This ensures that the model always operates on the accurate real-time topology at the moment a fault occurs.

The corresponding revisions in the Supplementary Information:

Supplementary Notes

Overview of the IEC 61850 communication architecture in smart substations

Modern smart substations built upon the IEC 61850[13] standard adopt a unified, hierarchical, and networked automation architecture that integrates primary equipment, intelligent electronic devices (IEDs), and supervisory systems through interoperable communication services and deterministic Ethernet networks.

The system follows the conventional three-level, two-network framework, including process, bay and station levels interconnected by a process bus and a station bus (Supplementary Fig. 1a). At the process level, merging units and intelligent terminals interface directly with primary apparatus such as current and voltage transformers, circuit breakers, and disconnectors, acquiring analog electrical quantities and equipment status and converting them into digital data. The bay level hosts protection, control, and interlocking IEDs, which subscribe to real-time measurement streams from the process level and exchange fast event and command messages to perform protection and automation. The station level provides global supervision, human-machine interfaces, data management, fault recording, and communicates with upper-layer control centers; it also hosts high-level applications such as digital-twin models and advanced analytics.

Information exchange across these three levels relies on the three principal IEC 61850 service models: SV (Sampled Values), GOOSE (Generic Object Oriented Substation Event), and MMS (Manufacturing Message Specification). SV is used at the process level to transmit digitized current and voltage measurements from merging units to bay-level protection and control IEDs. In typical 50 Hz systems, SV streams operate at 80 samples per cycle (4 kHz) for protection-oriented applications and 256 samples per cycle (12.8 kHz) for enhanced measurement and waveform recording accuracy. The end-to-end transfer time for SV used in

protection applications shall not exceed 3 ms, ensuring that protection algorithms receive fresh samples with negligible latency. GOOSE provides fast, reliable, peer-to-peer exchange of binary status and control information among bay-level IEDs and between bay-level devices and process-level intelligent terminals. The end-to-end transmission time of GOOSE messages used for high-speed tripping, interlocking, and fast state exchange shall be within 3 ms. MMS provides client-server services over TCP/IP between bay IEDs and station-level servers, carrying supervisory, operational, and maintenance information such as protection operation reports, alarms, setting parameters, and fault records; it is not used for the most time-critical protection functions but for comprehensive system monitoring and control.

These communication services are mapped onto two deterministic Ethernet networks. The process bus interconnects process-level devices and bay-level IEDs, carrying high-rate SV streams and time-critical GOOSE messages, and therefore requires microsecond- to millisecond-level latency performance. The station bus links the bay and station levels, transporting MMS traffic for supervisory control and data acquisition, and—where required—GOOSE messages for inter-bay coordination, busbar protection, or fast station-level interlocking. In the substation considered in this work, both buses are implemented on a high-speed Ethernet infrastructure with link data rates not lower than 100 Mbps, ensuring adequate bandwidth for concurrent SV, GOOSE, MMS, and other auxiliary traffic.

A unified time-synchronization system ensures coherent timestamping and time alignment across all three levels of the digital substation. In typical deployments, station-level devices obtain time via the Simple Network Time Protocol (SNTP), which provides millisecond-level accuracy adequate for supervisory and other non-time-critical functions. In contrast, bay-level and process-level equipment are normally synchronized using either the Inter-Range Instrumentation Group time code B (IRIG-B) or the IEEE 1588v2 Precision Time Protocol (PTP), both of which satisfy the microsecond-level timing requirements of protection and high-precision measurement applications. In the 110 kV smart substation investigated in this study, both BeiDou Navigation Satellite System (BDS) and the Global Positioning System (GPS) time references are available, with BDS used as the primary source, and the bay and process levels employ IEEE 1588v2 PTP as the main synchronization method, achieving a timing accuracy better than 1 μs across the IEDs.

Latency analysis of the digital twin system

……

Before the fault occurs, the digital twin continuously receives real-time switching states of circuit breakers, disconnectors, and grounding switches via IEC 61850 GOOSE and MMS services, ensuring that the pre-fault topology is already available at the moment the fault begins.

……

Q6. On page 5, clarify how many topologies were required before generalization performance saturated, and how the authors avoided over-fitting to specific topologies?

A6. We sincerely thank the reviewer for this insightful question regarding the number of topologies needed for satisfactory generalization and how overfitting to specific topologies was avoided. This issue is indeed critical for validating the robustness and scalability of the proposed framework. We have therefore

added a detailed explanation of the topology space and the rationale behind our dataset design in the ***Supplementary Discussion -Generalization saturation and mitigation of topology overfitting*.**

The studied system corresponds to a 110 kV smart substation, whose feasible operating topologies are inherently limited by practical engineering constraints, including busbar arrangements and transformer operating modes. The substation employs a double-bus structure with a bus-tie breaker, resulting in exactly three permissible 110 kV busbar configurations. Three transformers (#1, #2, #3) further form nine admissible transformer operating modes, determined by whether one, two, or all three transformers are energized and whether transformer #2 connects to busbar I or II. The 10 kV busbar configuration is fully dictated by the transformer mode. Together, these constraints yield 3 × 9 = 27 physically realizable topologies, which define the complete operational topology space of the substation.

Although 27 topologies are theoretically feasible, in the context of fault diagnosis, t**he factor that governs model generalization is not the number of topologies included in the training set, but rather the diversity of fault-propagation pathways they contain.** A topology influences the diagnostic model primarily through the paths along which fault signatures propagate—i.e., the local connectivity patterns and short propagation segments such as transformer-busbar transitions, busbar branching structures, and different transformer wiring schemes. As long as the training set covers all types of these elementary propagation segments, the model can flexibly compose them to handle any unseen global topology.

The ten historical topologies used for model training, while not the minimal required set, already span all essential propagation motifs within the substation. Specifically, they include:

- all three 110 kV busbar modes,
- representative transformer operating patterns (three-transformer, two-transformer, and single-transformer modes),
- both wiring schemes of transformer #2.

Because all remaining feasible topologies can be regarded as recombinations of these already covered propagation substructures, the GAT-based model does not memorize individual adjacency matrices. Instead, it learns path-level invariants—local structural patterns through which fault information flows, including multiple distinct branching and convergence patterns of power-flow and fault-propagation pathways. Consequently, generalization performance saturates once the training topologies provide full coverage of these fault-propagation building blocks, which our ten training topologies already achieve. This explains the strong performance on the four unseen test topologies without evidence of overfitting.

The corresponding revisions in the Manuscript and Supplementary Information:

Methods (Line 565-570)

Dataset generation

……

To enhance the model’s adaptability to diverse network configurations, multiple substation topologies are incorporated into the dataset. These topologies are derived from commonly observed configurations in

historical operation data, representing various connection structures and equipment layouts. By including multiple topological variations, the model is trained to recognize fault patterns across different network structures, preventing overfitting to a single configuration and ensuring strong adaptability in complex and unseen operational environments (Supplementary Discussion).

…….

Supplementary Information

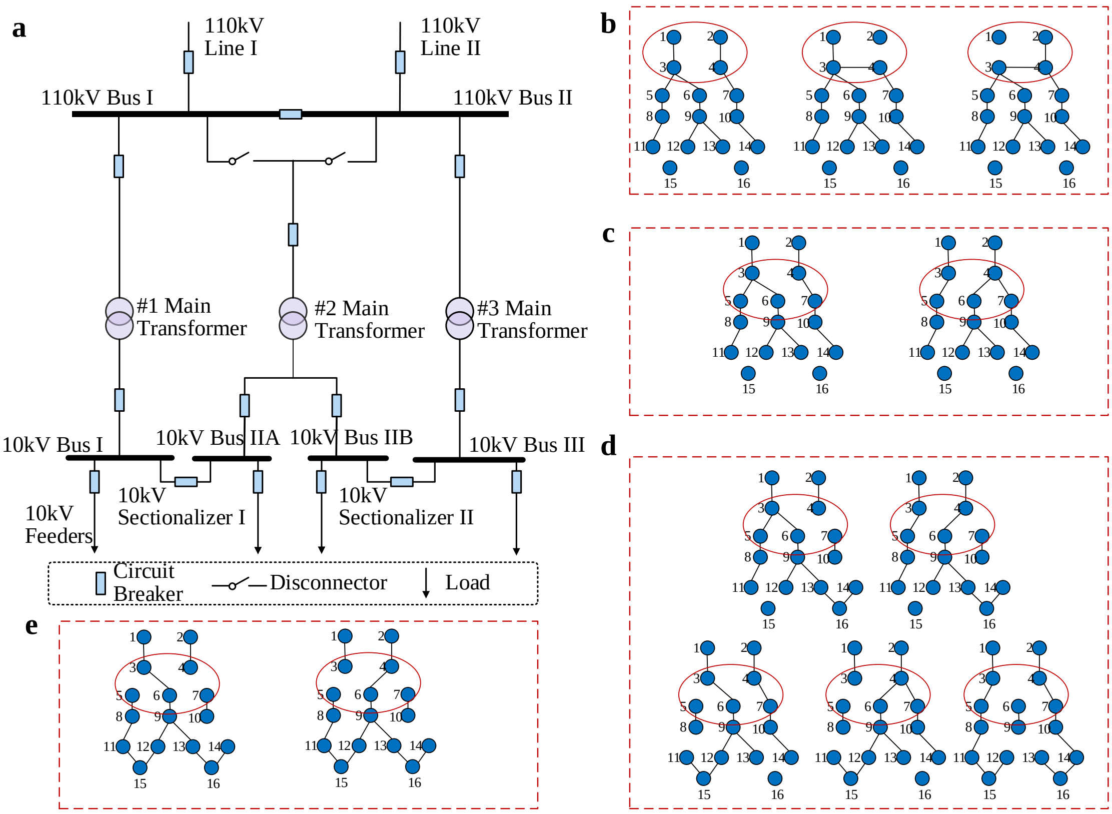

Supplementary Figure 10. System topology and operational configurations of the studied 110 kV substation. a Overall layout of the studied 110 kV substation. The system comprises two 110 kV incoming lines, two 110 kV busbars, three main transformers with both high- and low-voltage terminals, four 10 kV busbars, and two 10 kV sectionalizer switches, defining 16 physically meaningful fault locations within the primary network. **b–e** Illustration of all physically feasible topology categories in the substation, determined by permissible combinations of 110 kV busbar configurations and transformer operating modes. **b** Three admissible 110 kV busbar configurations arising from the double-bus scheme: (i) two bus sections operating independently (bus-tie open), (ii) bus-tie closed with Line I energized and Line II on standby, and (iii) bus-tie closed with Line II energized and Line I on standby. **c** Two feasible three-transformer operating modes, where Transformer #2 can be connected either to Bus I or Bus II. **d** Five admissible two-transformer operating modes. Any two of the three transformers may operate; if Transformer #2 is included, it again has two possible wiring options, yielding five distinct configurations. **e** Two feasible single-transformer operating modes. Only Transformer #2 can operate alone, as the single operation of Transformer #1 or #3 cannot supply all four 10 kV bus sections simultaneously. Together, these constrained combinations form a total of 27 physically realistic topologies for the substation. Among them, ten representative topologies are selected for model training.

Supplementary Discussion

Generalization saturation and mitigation of topology overfitting

The system analyzed in this study corresponds to a single 110 kV smart substation (Supplementary Fig. 10a). Due to its compact scale, fixed equipment configuration, and strict operational constraints, the number of physically feasible operating topologies is inherently limited. These admissible topologies arise from permissible combinations of busbar configurations and transformer operating modes.

1. 110 kV Busbar Configuration

The substation utilizes a double-bus structure with a bus-tie breaker, leading to three operational modes for the 110 kV busbar:

- Two bus sections operating independently (with the bus-tie breaker open).
- Bus-tie breaker closed, Line I energized, Line II standby.
- Bus-tie breaker closed, Line II energized, Line I standby.

Thus, the 110 kV busbar contributes three possible topologies (Supplementary Fig. 10b).

2. Transformer Operating Modes

The substation operates three transformers (#1, #2, #3), and the admissible operating combinations are constrained by system supply requirements:

- Three-transformer operation

Transformer #2 has two valid wiring schemes: connected to 110 kV busbar I or busbar II, allowing for 2 configurations (Supplementary Fig. 10c).

- Two-transformer operation:

Any two of the three transformers may operate, and if transformer #2 is included, it has two wiring options, resulting in 5 configurations (Supplementary Fig. 10d).

- Single-transformer operation:

Only transformer #2 can operate alone, as the single operation of transformer #1 or #3 does not provide sufficient power to all four 10 kV bus sections simultaneously, leading to 2 configurations (Supplementary Fig. 10e).

This gives a total of 9 transformer configurations.

3. 10 kV Busbar Configurations

The 10 kV busbar configuration is determined directly by the operating transformer configuration, ensuring that power is supplied to all feeders. Therefore, the 10 kV busbar topology is entirely defined by the transformer operational modes.

4. Total Feasible Topologies

Combining the above operational constraints, 3×9=27 distinct operational topologies for the substation, which form the complete and physically realistic topology space for the system. No additional topologies are permitted by real-world operational constraints.

5. Generalization Across Topologies

Although 27 distinct topologies are theoretically possible, in practice, the generalization of the model is more influenced by the structural diversity within the training data rather than the absolute number of topologies. To ensure robust model generalization, the training dataset includes a representative subset of these topologies that captures the most important structural variations:

- The 3 distinct 110 kV busbar modes
- Representative transformer operational modes (including three-transformer, two-transformer, and one-transformer configurations)
- Both wiring configurations of transformer #2

The ten topologies used in model training already cover the essential structural variations of the substation, including all busbar modes, representative transformer operating patterns, and the key wiring configurations. Because the remaining feasible topologies can be viewed as minor variations or combinations of these structural motifs, the model learns generalizable topology-dependent patterns rather than memorizing specific configurations. Consequently, even though only ten topologies are included in the training set, they provide sufficient diversity for the model to generalize reliably to unseen topologies, effectively avoiding overfitting and ensuring stable diagnostic performance in new operational conditions.

Q7. Page 4: In Eq. (1): clarify whether outputs are probability-calibrated (e.g., temperature scaling), and state decision thresholds for $\tilde{Y}3$ (multi-label).

A7. We sincerely thank the reviewer for raising this important question regarding probability calibration and the decision thresholds used for the multi-label protection-failure prediction task $\tilde{Y}_3$. This clarification indeed strengthens the methodological transparency of our framework.

In the proposed method, the outputs of the classifier heads are produced using softmax (for fault location and fault type) or sigmoid (for multi-label protection-failure detection), yielding confidence scores within [0,1]. These scores are not post-processed using probability-calibration techniques such as temperature scaling or Platt scaling. The key reason is that actual faults in real power systems are extremely rare, and their true occurrence probabilities are highly skewed toward normal operation. Under such conditions, calibrated probabilities derived from historical statistics would not be meaningful for model training or evaluation.

Moreover, the training dataset in this work is generated through a digital-twin-based enumeration mechanism that systematically produces all feasible combinations of fault locations, fault types, and protection-failure patterns. This dataset is therefore structurally exhaustive rather than probability-representative, and each enumerated scenario can be treated as an approximately equal-likelihood training event for learning purposes. As a result, the model outputs are intended to function as classification confidence scores, rather than calibrated estimators of real-world event frequencies. Given this data construction paradigm, additional probability calibration is unnecessary and would not provide practical benefits.

For the multi-label protection-failure task $\tilde{Y}_3$, a fixed decision threshold of 0.5 is used—i.e., a protection element is classified as “failure to operate” when its predicted probability exceeds 0.5. Sensitivity analyses showed that thresholds in the range of 0.4–0.6 yield nearly identical diagnostic accuracy, indicating that the output scores are already well separated and explicit calibration brings no practical benefits.

Q8. On page 7, the authors selected RMS values for waveform representation. Please justify why this was preferred over other features like wavelets, and whether the architecture could adapt to other inputs without redesign?

A8. We sincerely thank the reviewer for this insightful question. The reviewer raises two important points: the rationale for selecting RMS values as the waveform representation, and whether the proposed architecture can accommodate other input modalities without structural redesign.

First, RMS values were adopted because they provide a direct and physically meaningful measure of current magnitude variation during fault events. RMS features effectively suppress high-frequency measurement noise while preserving the essential disturbance characteristics required for fault-location and fault-type identification. This representation is computationally efficient and robust to sampling-frequency variations across different fault-recording devices in practical substations. Compared with wavelet-based or other high-resolution signal-processing features, RMS features offer a simpler yet sufficiently informative representation for capturing primary-system fault behavior, without introducing substantial computational overhead or increasing model sensitivity to parameter choices in time-frequency transforms.

Second, the proposed multimodal fusion framework is designed to be modular and easily extensible. Each input modality (alarm, SCADA, waveform) is processed through a modality-specific embedding layer followed by an independent GAT-based encoder. This architecture allows new data types—such as environmental sensor streams or image/video data from inspection robots—to be integrated by adapting only the corresponding embedding layer, without modifying the overall model structure or fusion mechanism. The GAT-based topology encoder and the self-attention fusion module remain unchanged, ensuring compatibility with future extensions. In fact, we are currently experimenting with incorporating inspection-robot video data as an additional modality, using the same embedding-encoder-fusion pattern, further demonstrating the flexibility and scalability of the proposed framework.

Q9. On p. 9 - 4.2.1, the authors describe DAE pretraining. It'll be great to provide readers with the computational cost (epochs, runtime per epoch, GPU/CPU specs) if available?

A9. We sincerely thank the reviewer for this helpful suggestion. Providing computational details indeed improves the transparency and reproducibility of the proposed method.

We have added detailed information on the computational cost of the DAE pretraining stage. All DAE modules—including those used in the baseline models—were trained on a workstation equipped with an Intel(R) Xeon(R) CPU E3-1230 v5 and an NVIDIA RTX 3090 GPU (24 GB). Each DAE was trained for 100 epochs using the Adam optimizer with an initial learning rate of 1×10^{-3}. On average, DAE training required approximately 0.2–1.5 minutes per epoch (model-specific values provided in ***Supplementary Table 7***), depending on the architecture of the corresponding encoder and decoder.

The corresponding revisions in the Supplementary Information:

Supplementary Table 7: Training time, inference time and GPU memory usage of each module across all baseline models

Model	DAE Training Time (s/epoch)	DAE GPU Mem (MB)	SCL Training Time (s/epoch)	SCL GPU Mem (MB)	Classifier Training Time (s/epoch)	Classifier GPU Mem (MB)	Total Training Time (hours)	Inference Time (ms/sample)
MPNN (end-to-end training)	N/A	N/A	N/A	N/A	75.3	2183.9	5.23	622

MLP-based	13.62	178.5	10.01	311.4	27.92	198.0	2.87	155
ResNet-based	91.13	6153.4	37.01	1689.6	69.52	1594.9	9.41	337
LSTM-based	18.79	1943.4	11.62	781.7	28.42	684.8	3.14	157
GCN-based	89.68	438.8	39.84	361.9	88.31	262.7	10.84	563
Transformer-based	50.17	5540.8	21.29	627.8	41.30	531.1	5.44	181
Proposed	93.33	1132.0	41.4	383.7	100.90	286.6	11.90	560

Q10. Page 10 - Eq. (10): did the authors tune the τ temperature parameter, and if so what was its effect on performance?

A10. We sincerely thank the reviewer for raising this important question. The temperature parameter τ in the supervised contrastive loss (SCL) plays a critical role in shaping the similarity distribution among samples. Specifically, τ controls the sharpness of the softmax function used when contrasting positive and negative embeddings: smaller τ produces a sharper distribution, emphasizing hard negatives and enforcing stronger separation in the embedding space; larger τ yields a smoother distribution, reducing gradient magnitudes and leading to softer contrastive effects.

Following common practice in supervised contrastive learning literature, we selected a range of values that cover the typical operational regimes of the temperature parameter. We evaluated $\tau \in \{0.03, 0.05, 0.07, 0.10, 0.20\}$, while keeping all other training settings unchanged. The results on the test dataset for the fault location and fault type classification are summarized below:

τ	Fault location accuracy (%)	Fault type accuracy (%)
0.03	96.58	99.51
0.05	97.08	99.56
0.07	97.04	99.62
0.10	96.81	99.60
0.20	96.95	99.57

The results demonstrate that the diagnostic performance is largely insensitive to the choice of τ within this commonly used range. Accuracy variations remain minimal, indicating that the supervised contrastive embedding space is stable under different temperature strengths. Given this robustness and the fact that τ=0.07 is the most widely adopted and empirically validated default value in supervised contrastive learning studies[Ref 51], we retain τ=0.07 in our final model.

[Ref 51] Khosla, P. et al. Supervised contrastive learning, DOI: 10.48550/arXiv.2004.11362 (2021).

Q11. Page 10: For protection failure classification (i.e., Eq. 12), what was the class distribution between failure and non-failure events, and how did the authors address imbalance?

A11. We sincerely thank the reviewer for raising this important question regarding the class distribution in the protection-failure classification task. It is important to clarify that in real power-system operation, actual protection-failure events are extremely rare, and the vast majority of observed cases correspond to normal protection behavior. As a consequence, real-world protection data are highly imbalanced and cannot provide the balanced and diverse samples required to train a reliable multi-label classifier. To address this issue and to obtain a balanced and physically consistent training dataset, we employ a fault enumeration tree traversal method based on breadth-first search (BFS).

In our formulation, protection-failure detection is treated as a multi-label classification problem, where each sample contains a binary label for every protection relay (PR) that may participate in the corresponding fault scenario. Class balance is therefore assessed per relay, rather than at the level of globally aggregated failure versus non-failure samples.

For each combination of fault location and fault type, we first run a baseline simulation in which all PRs operate correctly. This determines which relays are expected to trip under that condition. Based on this baseline behavior, we construct a protection-state tree and systematically enumerate protection-failure cases using the BFS-based digital-twin mechanism described in the ***Supplementary Methods***. The root of the tree corresponds to the healthy scenario, and its descendants represent various combinations of PR refusal events evolving logically from the simulated secondary-system states.

Although this enumeration generates a large number of failure scenarios, different fault locations and types activate different sets of PRs. When these diverse scenarios are aggregated, each PR participates in many fault cases, leading to both normal-operation and failure labels being sufficiently represented for that relay. As a result, for any given protection device, the number of samples in which it is labeled as “normal operation” and the number in which it is labeled as “failure” become comparable in magnitude.

In other words, while the dataset is not globally balanced at the sample level, it is effectively balanced for each individual PR’s binary label, which is precisely the balance required for a multi-label classifier. Owing to this relay-wise balance achieved through systematic enumeration, we did not encounter the typical class-imbalance issue present in real-world protection-failure datasets, and no additional techniques such as resampling or class-weighted loss functions were necessary.

Q12. Page 13, Line 300: Table 2 lists parameters, but why batch size 16 was chosen, whether larger sizes were tested, and the number of samples used for training vs. validation/testing?

A12. We sincerely thank the reviewer for this insightful and constructive comment. We fully agree that the choice of batch size is an important factor in training deep models, and following the reviewer’s suggestion, we conducted additional experiments with larger batch sizes to examine their impact on

performance. These extended tests confirmed that larger batches indeed lead to better diagnostic accuracy and more stable optimization behavior. Based on these results, we updated the final configuration to use batch size = 32, which provides the best balance of performance, contrastive-learning stability, and computational efficiency.

In our implementation, the batch size refers to the number of **raw fault scenarios** sampled in each optimization step. For each raw scenario, nine augmented variants are generated, including Gaussian-noise corruption, sampling-loss corruption, and sensor-failure corruption. Thus, a batch size of 32 corresponds to 32 raw scenarios and 288 augmented variants, i.e., 320 input samples in total per training step. This batch composition is intentional:

- For the denoising autoencoder (DAE) stage, pairing each clean sample with its noisy or incomplete variants enables the DAE to explicitly learn mappings from corrupted inputs to clean latent representations, which significantly improves robustness under missing or degraded data.
- For the supervised contrastive learning (SCL) stage, grouping the raw and augmented versions of the same scenario within the batch provides natural positive pairs and hard negatives, leading to more effective contrastive optimization and stable representation learning.

To identify an appropriate batch size, we conducted a sensitivity study using batch sizes of 8, 16, 32, and 64 across all three diagnostic tasks. The results, summarized in **Supplementary Table 4**, show that a batch size of 32 yields the best overall performance and training stability. Very small batches (e.g., 8) led to noisier gradients and slower convergence, while very large batches (e.g., 64) slightly reduced the effectiveness of supervised contrastive learning due to weaker intra-batch positive/negative contrast and increased GPU memory consumption. Batch size = 32 provides the highest test accuracies across tasks while maintaining efficient training, and is therefore adopted throughout the experiments.

Regarding dataset scale, a full summary is provided in ***Supplementary Table 3***. In total, the digital-twin simulations generated 11,597 distinct raw training fault scenarios and 3,890 raw testing scenarios using unseen topologies. After applying nine augmentation variants per raw sample, the final dataset contains:

- 115,970 training samples,
- 38,900 testing samples,

each including synchronized alarm, SCADA, and waveform modalities. All test topologies are different from those in the training set, ensuring no information leakage.

The corresponding revisions in the Manuscript and Supplementary Information:

Results (Line 177–183)

Comparison of fault diagnosis models

……

Multiple batch sizes (8, 16, 32, 64) are evaluated, and a batch size of 32 is selected based on the highest validation accuracy (Supplementary Table 4). It is worth noting that each batch internally consists of one raw fault sample together with its nine augmented variants (noise-corrupted, sampling-loss, and sensor-failure

versions). This batch composition is intentionally designed to facilitate both the DAE pretraining—by providing paired clean/perturbed samples—and the SCL stage, where raw-augmented pairs naturally form positive samples while other samples in the batch serve as negatives.

…….

Supplementary Table 4: Performance of the proposed model in test dataset with different training batch sizes

Batch size	Fault location accuracy	Fault type classification accuracy	Protection failure detection accuracy
8	0.9621±0.0051	0.9956±0.0003	0.9015±0.0046
16	0.9678±0.0015	0.9960±0.0003	0.9000±0.0039
32	**0.9703±0.0020**	**0.9960±0.0003**	**0.9035±0.0031**
64	0.9662±0.0014	0.9946±0.0001	0.9003±0.0041

Q13. Table 3 reports accuracy values. It'll be great to also provide mean ± standard deviation over multiple seeds to demonstrate stability?

A13. We sincerely thank the reviewer for this helpful suggestion. In the revised manuscript, we have incorporated model-stability analysis by repeating all experiments under five different random seeds (seeds = {42, 123, 3407, 2024, 777}). For each diagnostic task and each baseline model, the performance metrics (accuracy, precision, recall, F1-score) are now reported as mean ± standard deviation, demonstrating the robustness and stability of the results across random initializations.

These updated results are presented in the revised ***Results - Comparison of fault diagnosis models*** section and are fully detailed in the ***Supplementary Data***. This addition ensures that the reported performance values are statistically reliable and not dependent on a single initialization.

The corresponding revisions in the Manuscript:

Results (Line 204–205)

Comparison of fault diagnosis models

……

Each experiment is repeated under five random seeds, and results are reported as mean ± standard deviation to evaluate model stability.

……

Table 1 Fault location identification accuracy (%)

Method	Original test dataset	SNR 50dB	SNR 40dB	SNR 30dB	Sample loss 10%	Sample loss 20%	Sample loss 30%	Sensors failure 10%	Sensors failure 20%	Sensors failure 30%
TCFPN[12]	67.92	67.92	67.92	67.92	62.08	54.45	47.07	67.92	67.92	67.92
MPNN[21]	93.01 ±0.42	93.02 ±0.45	93.03 ±0.44	92.95 ±0.38	92.67 ±0.29	92.14 ±0.36	91.94 ±0.46	91.08 ±0.26	89.39 ±0.43	85.12 ±0.46
MLP-based	0.8711 ±1.19	87.09 ±1.14	87.11 ±1.17	86.99 ±1.05	86.83 ±1.03	86.23 ±0.90	85.79 ±0.90	86.48 ±1.04	85.33 ±1.13	84.10 ±0.89
ResNet-based	96.69	96.70	96.68	96.59	96.56	96.01	95.63	95.88	94.65	92.58

	±0.37	±0.36	±0.34	±0.32	±0.49	±0.42	±0.62	±0.71	±0.73	±0.96
LSTM-based	0.6131 ±4.85	61.33 ±4.87	61.30 ±4.82	61.19 ±4.94	60.92 ±5.07	60.30 ±4.99	59.44 ±4.92	54.98 ±3.29	50.52 ±2.91	45.34 ±2.67
GCN-based	95.93 ±0.68	95.93 ±0.65	95.90 ±0.63	95.77 ±0.63	95.78 ±0.58	95.18 ±0.80	94.95 ±0.87	95.65 ±0.67	94.83 ±0.62	93.80 ±0.65
Transformer-based	87.34 ±2.43	87.34 ±2.42	87.31 ±2.40	87.23 ±2.23	87.32 ±2.35	86.90 ±2.68	86.51 ±2.72	85.30 ±2.01	82.10 ±1.90	78.03 ±1.77
Proposed	97.56 ±0.23	97.58 ±0.25	97.56 ±0.25	97.49 ±0.21	97.29 ±0.27	96.94 ±0.23	96.61 ±0.25	97.18 ±0.22	96.62 ±0.22	95.47 ±0.22

Table 2 Fault type classification accuracy (%)

**Method**	**Original test dataset**	**SNR 50dB**	**SNR 40dB**	**SNR 30dB**	**Sample loss 10%**	**Sample loss 20%**	**Sample loss 30%**	**Sensors failure 10%**	**Sensors failure 20%**	**Sensors failure 30%**
TCFPN[12]	N/A	N/A	N/A	N/A	N/A	N/A	N/A	N/A	N/A	N/A
MPNN[21]	99.99 ±0.02	99.99 ±0.02	99.99 ±0.02	99.99 ±0.02	99.91 ±0.04	99.84 ±0.04	99.57 ±0.06	99.48 ±0.12	98.61 ±0.08	97.35 ±0.20
MLP-based	93.10 ±1.45	93.11 ±1.43	93.12 ±1.43	93.06 ±1.46	92.85 ±1.20	92.74 ±1.15	92.55 ±1.05	90.93 ±1.22	88.90 ±1.42	85.54 ±1.57
ResNet-based	99.72 ±0.23	99.72 ±0.22	99.75 ±0.21	99.79 ±0.18	99.55 ±0.15	99.47 ±0.19	99.19 ±0.25	98.05 ±0.21	96.77 ±0.41	95.17 ±0.47
LSTM-based	85.74 ±2.04	85.74 ±2.03	85.78 ±2.03	85.92 ±2.03	85.65 ±2.10	85.50 ±2.18	85.32 ±2.31	57.84 ±2.35	44.98 ±2.59	33.86 ±2.41
GCN-based	99.87 ±0.13	99.87 ±0.13	99.87 ±0.13	99.86 ±0.11	99.76 ±0.17	99.56 ±0.19	99.54 ±0.15	98.54 ±0.11	97.46 ±0.14	95.99 ±0.29
Transformer-based	92.02 ±1.17	92.02 ±1.20	92.02 ±1.26	91.90 ±1.20	91.87 ±1.19	91.59 ±1.06	91.25 ±0.93	89.60 ±1.17	86.09 ±1.13	80.62 ±1.02
Proposed	100 ±0.00	100 ±0.00	100 ±0.00	100 ±0.00	99.98 ±0.03	99.94 ±0.05	99.85 ±0.09	99.57 ±0.11	98.85 ±0.17	97.78 ±0.14

Table 3 Protection failure detection accuracy (%)

**Method**	**Original test dataset**	**SNR 50dB**	**SNR 40dB**	**SNR 30dB**	**Sample loss 10%**	**Sample loss 20%**	**Sample loss 30%**	**Sensors failure 10%**	**Sensors failure 20%**	**Sensors failure 30%**
TCFPN[12]	69.23	69.23	69.23	69.23	60.54	49.77	40.82	69.23	69.23	69.23
MPNN[21]	N/A	N/A	N/A	N/A	N/A	N/A	NA	NA	NA	NA
MLP-based	84.82 ±0.95	84.81 ±0.95	84.77 ±0.94	84.71 ±1.01	82.38 ±1.16	78.35 ±1.09	74.42 ±1.28	83.94 ±0.65	82.25 ±0.58	79.03 ±0.52
ResNet-based	91.63 ±0.43	91.65 ±0.42	91.71 ±0.41	91.69 ±0.41	89.26 ±0. 41	85.32 ±0.61	81.52 ±0.70	91.28 ±0.50	89.96 ±0.58	87.64 ±0.72
LSTM-based	80.25 ±2.24	80.17 ±2.28	80.16 ±2.24	79.86 ±2.17	73.51 ±1.21	67.19 ±1.05	62.77 ±1.35	75.12 ±1.44	70.03 ±1.11	61.96 ±0.96
GCN-based	91.60 ±0.53	91.60 ±0.53	91.55 ±0.50	91.36 ±0.45	89.04 ±0.64	84.69 ±0.87	81.20 ±0.63	91.17 ±0.59	89.65 ±0.54	87.65 ±0.63
Transformer-based	74.93 ±0.92	74.93 ±0.94	74.92 ±0.90	74.75 ±0.91	67.76 ±1.67	62.93 ±2.82	59.07 ±2.74	73.12 ±0.96	70.83 ±1.08	65.40 ±0.91
Proposed	92.75 ±0.30	92.77 ±0.38	92.72 ±0.38	92.62 ±0.38	90.46 ±0.45	86.30 ±0.56	82.61 ±0.59	92.13 ±0.25	91.51 ±0.31	89.62 ±0.54

Q14. Figure 13: Could authors quantify the additional computational cost of each module (DAE, self-attention, SCL) compared to a CNN baseline?

A14. We sincerely thank the reviewer for raising this important question regarding the computational cost of each model component. To provide a transparent comparison, we added a new table in the supplementary material (Supplementary Table 7) that reports the detailed computational requirements for all baseline models and the proposed method, including:

- DAE training time

- DAE GPU memory usage
- SCL training time
- SCL GPU memory usage
- Classifier training time
- Classifier GPU memory usage
- Total training time
- Inference time

Among all models, MPNN is the only one that is trained in an end-to-end manner and therefore does not include DAE or SCL stages; only the classifier training stage is required. For the MLP-based, ResNet-based (CNN baseline), LSTM-based, GCN-based, Transformer-based, and proposed models, the baseline architectures are constructed by replacing the GAT module (i.e., the encoder-decoder structure in the DAE) with the corresponding model-specific encoders. All other components-including the self-attention module, classifier architecture, optimization settings, input formats, and training pipeline—are kept strictly identical. Furthermore, their hyperparameters and parameter counts were carefully adjusted to maintain comparable model complexity (Supplementary Table 5).

As a result, the primary difference in computational cost across baselines arises in the DAE training stage, where each model uses its own encoder-decoder architecture. In the subsequent SCL and classifier stages, although the training framework is identical, the forward-pass complexity of each baseline's encoder introduces additional cost differences, which are also captured in ***Supplementary Table 7***. While the proposed model does increase the training time compared to some baselines, the overall training and inference costs remain reasonable, without introducing significant overhead relative to other deep learning models

The corresponding revisions in the Supplementary Information:

Supplementary Table 7: Training time, inference time and GPU memory usage of each module across all baseline models

Model	DAE Training Time (s/epoch)	DAE GPU Mem (MB)	SCL Training Time (s/epoch)	SCL GPU Mem (MB)	Classifier Training Time (s/epoch)	Classifier GPU Mem (MB)	Total Training Time (hours)	Inference Time (ms/sample)
MPNN (end-to-end training)	N/A	N/A	N/A	N/A	75.3	2183.9	5.23	622
MLP-based	13.62	178.5	10.01	311.4	27.92	198.0	2.87	155
ResNet-based	91.13	6153.4	37.01	1689.6	69.52	1594.9	9.41	337
LSTM-based	18.79	1943.4	11.62	781.7	28.42	684.8	3.14	157
GCN-based	89.68	438.8	39.84	361.9	88.31	262.7	10.84	563
Transformer-based	50.17	5540.8	21.29	627.8	41.30	531.1	5.44	181
Proposed	93.33	1132.0	41.4	383.7	100.90	286.6	11.90	560

Q15. Figure 14 shows t-SNE visualizations. For better clarification, could authors supplement this with quantitative clustering metrics such as silhouette score or inter/intra-class distance ratios?

A15. We sincerely thank the reviewer for this valuable suggestion. We agree that relying solely on t-SNE visualizations may lead to ambiguous interpretations due to the well-known nonlinear and locally preserving nature of t-SNE. As suggested, we have added quantitative clustering metrics to provide a more rigorous evaluation of the feature-space structure.

In the revised manuscript, we first clarify that t-SNE is used strictly for qualitative visualization. Since t-SNE distortions may impair global distance interpretation, all quantitative analyses are carried out in the original high-dimensional embedding space, not in the 2D projection. To quantitatively assess clustering quality for both the fault-location and fault-type classification embeddings, we compute:

- Silhouette score
- Intra-class distance
- Inter-class distance
- Inter/Intra-class distance ratio

as detailed in ***Supplementary Methods*** and ***Supplementary Equations (1–9)***. A new table has been added

(***Table 4*** in the main text) summarizing these results:

Table 4 Quantitative clustering metrics for multi-class feature embeddings under different model configurations

Task	Feature Representation	Silhouette Score	intra-class distance	inter-class distance	Inter/Intra
Fault location	Encoder	0.0269	4.5099	5.2125	1.1558
Fault location	SA without SCL	0.1548	109.4283	148.1606	1.3540
Fault location	SA with SCL	0.7600	15.7616	117.0393	7.4256
Fault type classification	Encoder	0.0009	4.9346	5.1503	1.0437
Fault type classification	SA without SCL	0.1697	89.6750	124.9386	1.3932
Fault type classification	SA with SCL	0.7309	17.0048	85.7115	5.0404

The results show consistent improvements across both tasks. Self-attention fusion already strengthens cluster compactness compared with the raw encoder output. Adding SCL further enhances class separability, yielding significantly larger silhouette scores and higher inter/intra-class distance ratios. For example, in the fault-location task, the silhouette score increases from 0.15 →0.76, and the inter/intra ratio increases from 1.35 →

7.43 after adding SCL. These quantitative results clearly demonstrate that SCL substantially improves the discriminability of the learned latent space, complementing the qualitative trends observed in the t-SNE plots.

The corresponding revisions in the Manuscript:

Results (Line 288-306)

Ablation Study

……

To investigate how self-attention fusion and SCL influence the structure of the learned feature space, we visualize the embeddings using t-SNE with fixed hyperparameters (perplexity = 30, learning rate = 200, iterations = 1000) (Fig. 2d-i). It is important to note that t-SNE[45] is a nonlinear, locally preserving embedding method; therefore, the distances in its 2D projected space do not reflect the true geometry of the original high-dimensional feature space[46]. Consequently, t-SNE is used only for qualitative visualization, whereas all quantitative metrics are computed in the original feature space.

To rigorously evaluate clustering quality in the two multi-class tasks, several metrics explicitly supporting multi-class settings are adopted, including the silhouette score, intra-class distance, inter-class distance, and their ratio (Supplementary Methods, Supplementary Equation (3)–(11)). The quantitative results reveal consistent trends across both tasks (Table 4). Self-attention fusion improves cluster compactness compared with the encoder output, while introducing SCL further produces substantially more compact and better-separated feature clusters. For the fault location task, the silhouette score increases from 0.15 (SA) to 0.76 (SA+SCL), and the inter/intra-class distance ratio increases from 1.35 to 7.43. Similar improvements are observed in the fault type classification task. These results demonstrate that SCL significantly enhances the discriminability of the latent feature space, enabling the model to more effectively separate fault categories.

Q16. On p. 16, under the CloudPSS deployment, what is the communication latency between real-time data acquisition and the digital twin? please clarify.

A16. We sincerely thank the reviewer for this helpful comment. The reviewer requested that the operation time and diagnostic delay be highlighted for different scenarios. We agree that clarifying the end-to-end latency is important for demonstrating the practical feasibility of the proposed system.

We provide a detailed analysis of the diagnostic latency of the deployed digital twin system in ***Supplementary Information–Supplementary Notes–Latency analysis of the digital twin system.*** The end-to-end delay consists of three sequential stages: (i) data acquisition, (ii) data processing, and (iii) model inference. Among these, waveform file generation inside protection IEDs/fault recorders is the dominant contributor.

In Stage 1 (data acquisition), switching states, SOE logs, and SCADA measurements are delivered to the station level within milliseconds via IEC 61850 GOOSE and MMS, contributing negligible delay. However, protection IEDs must capture pre-fault and fault-period transients before generating COMTRADE files, and this process takes approximately 50 seconds in the studied 110 kV substation. File upload via MMS adds roughly 10 seconds, which is minor relative to the file-generation delay.

In Stage 2 (data processing), the digital twin reconstructs the topology, parses COMTRADE files, and formats all modalities into model inputs. This takes about 1 s.

In Stage 3 (model inference), the diagnostic network runs on an Intel Xeon E3-1230 v5 CPU and an NVIDIA RTX 3090 GPU, requiring approximately 0.5 s to produce the final result.

Because the SOE and SCADA streams arrive during waveform acquisition, the overall latency is dominated by waveform file generation. With waveform generation and transfer taking about 60 s in total, data preprocessing 1 s, and inference 0.5 s, the full end-to-end latency of the deployed system is approximately 60–65 s. This latency is acceptable in practice, as the proposed method is designed for post-fault diagnosis, where diagnostic accuracy and reliability are more critical than strict real-time response.

The corresponding revisions in the Supplementary Information:

Supplementary Notes

Latency analysis of the digital twin system

The end-to-end diagnostic latency of the digital twin system can be understood as the cumulative duration of three sequential stages: (i) acquiring the required data, (ii) processing and organizing the data into model inputs, and (iii) executing the diagnostic model.

- Stage 1 — Data acquisition.

Before the fault occurs, the digital twin continuously receives real-time switching states of circuit breakers, disconnectors, and grounding switches via IEC 61850 GOOSE and MMS services, ensuring that the pre-fault topology is already available at the moment the fault begins. During the fault, bay-level protection IEDs generate microsecond-synchronized Sequence-of-Events (SOE) logs, and SCADA analog measurements continue to be reported through MMS. These messages typically reach the station level within milliseconds and therefore do not contribute significantly to diagnostic latency.

In contrast, the acquisition of fault waveform data is inherently slower. Protection IEDs and standalone fault recorders must capture at least 2 s of pre-fault data and the entire fault transient before a COMTRADE file can be generated, and this file-generation process is time-consuming. In the 110 kV smart substation studied in this work, generating the waveform files from multiple triggered recorders takes approximately 50 seconds, making this step the dominant contributor to the end-to-end latency. Once generated, the waveform files—typically about 20 MB each—are uploaded to the station level via IEC 61850 MMS file services in around 10 seconds, which is negligible compared to the file-generation delay.

- Stage 2 — Data processing.

After all waveform files are available at the station level, the digital twin reconstructs the pre-fault topology, parses the COMTRADE channels, extracts the necessary diagnostic features, and formats the combined SOE, SCADA, and waveform information into the model’s input structure. These operations are computationally lightweight and require roughly 1 s.

- Stage 3 — Model inference.

The processed data are then fed into the diagnostic model, which runs on an Intel Xeon E3-1230 v5 CPU

and an NVIDIA RTX 3090 GPU. The inference stage is fast, producing the final diagnostic decision within approximately 0.5 s.

Because SOE and SCADA reporting overlap with the waveform acquisition phase, the overall latency is determined primarily by the waveform file-generation delay. Let t_1 denote the time for recorder-side file generation and upload, t_2 the data-processing time, and t_3 the inference time. The end-to-end latency is therefore $T_{latency}=t_1+t_2+t_3$. Given that $t_1\approx 60$ s, $t_2\approx 1$ s, and $t_3\approx 0.5$ s, the overall latency is dominated by the waveform file-generation process.

Q17. In the real case study (page 17), please describe in a paragraph any situations where the model misdiagnosed or required operator correction, and how uncertainty is flagged to operators when confidence is low?

A17. We sincerely thank the reviewer for this important question regarding the model's behavior during real-world deployment and the mechanisms that support operator oversight. This allows us to clarify how the system interfaces with operators and how uncertain predictions are handled in practice.

Although the model demonstrates high diagnostic reliability, occasional situations may arise in real operation where input measurements are influenced by transient sensor fluctuations, communication noise or partial data loss. These conditions may reduce the model's confidence in its predictions. To ensure safety and transparency, a confidence-based alarm verification mechanism has been incorporated into the platform. In this mechanism, the model outputs the predicted labels together with the corresponding confidence scores produced by the final softmax or sigmoid layers for the three diagnostic tasks of fault location, fault type classification, and protection failure detection. When the confidence score of any predicted result falls below the predefined threshold of 0.90, a low-confidence alert is automatically triggered. This alert explicitly informs the operator that the diagnostic outcome may be unreliable and therefore requires manual review, validation, or correction. The outputs from the three diagnostic tasks are further ranked according to their confidence scores, with higher-confidence predictions assigned higher inspection priority, thereby guiding maintenance personnel to prioritize inspection actions accordingly. Conversely, predictions with confidence values above the threshold are treated as reliable and are presented without triggering an alarm. This mechanism ensures that any uncertain or ambiguous inference is explicitly flagged and reviewed before supporting operational decision-making.

The corresponding revisions in the Manuscript:

Results (Line 335–346)

Digital twin application platform

……

To enhance diagnostic reliability and support effective human–machine collaboration, a confidence-based alarm verification mechanism is integrated into the overall diagnostic framework. In this mechanism, the model outputs the predicted labels together with the corresponding confidence scores produced by the final softmax or sigmoid layers for the three diagnostic tasks of fault type classification, fault location, and protection failure detection. A unified confidence threshold of 0.90 is applied. Predictions with confidence values higher than this threshold are regarded as reliable and do not trigger an alarm. Predictions with lower

confidence values are treated as uncertain and will trigger an alarm so that the case is escalated for operator review. The outputs from the three diagnostic tasks are further ranked according to their confidence scores, with higher-confidence predictions assigned higher inspection priority, thereby guiding maintenance personnel to prioritize inspection actions accordingly. This mechanism ensures that potentially unreliable inferences receive human attention and thus improves the operational dependability of the diagnostic process.

……

Q18. On page 18 - line 389: the authors emphasize robustness to missing data. Please specify the maximum tolerable missing data percentage before performance becomes unreliable in real deployments?

A18. We sincerely thank the reviewer for this important question. To quantify the practical robustness of the proposed diagnostic framework, we conducted a systematic stress-test by evaluating the model across nineteen test datasets, including the raw test set, nine sampling-loss datasets (10%–90% sampling loss), and nine sensor-failure datasets (10%–90% sensors fail). These degraded datasets were specifically designed to emulate the extreme measurement conditions that may arise in real substation environments, allowing us to observe the degradation pattern of the three diagnostic tasks under progressively worsening data completeness.

The results show that the three tasks exhibit different levels of tolerance to missing data. Fault type classification remains highly robust and retains near-perfect performance even under very severe degradation; for example, its accuracy remains above 95% under up to 90% sampling loss and stays above 90% with sensor failures as high as 60%. This robustness is expected because fault type relies heavily on coarse system-wide patterns that remain identifiable even when many sampling or sensors are missing. Fault location shows moderate sensitivity to missing information: its accuracy remains above 90% when sampling loss is not greater than 60% and remains above 85% when sensor failure is within 40%, but it declines rapidly once these thresholds are exceeded, indicating the model's dependence on adequate spatial observability for precise localization. Protection-failure detection is the most sensitive task, as it relies on fine-grained device-level signals; its performance remains reasonably reliable (above 80–85%) when missing data is within roughly 30%, but drops sharply beyond 40% sampling loss or 30% sensor failure, with accuracy falling below 50% under extremely degraded conditions (e.g., >80% missing data). These findings indicate that while the overall framework demonstrates strong robustness under moderate degradation, extremely high degrees of missingness will inevitably compromise reliability-particularly for the protection-failure task.

Taken together, the stress-test suggests that reliable performance is maintained when sampling loss is within 60% and sensor failure is within 40% for fault location; when sampling loss is within 90% and sensor failure within 60% for fault type classification; and when missing data does not exceed approximately 30% for protection-failure detection. We have included a concise summary of these results in the revised manuscript and provided the complete numerical evaluations in ***Supplementary Table 9***.

The corresponding revisions in the Manuscript:

Results (Line 347–366)

Digital twin application platform

……

In addition to confidence-based verification, the robustness of the diagnostic model under incomplete measurements was evaluated through a stress test using nineteen datasets with 10%–90% sampling loss and 10%–90% sensor failures (Supplementary Table 8). The results show that the three diagnostic tasks have distinct tolerance limits. Fault type classification is the most resilient, maintaining above 95% accuracy even with 90% sampling loss and above 90% accuracy under 40% sensor failures. Fault location is moderately sensitive: its accuracy stays above 90% when sampling loss is within 60% and above 85% when sensor failures are within 40%, but drops sharply beyond these levels (e.g., 63.2% at 90% sampling loss and 59.4% at 80% sensor failure). Protection failure detection is the most vulnerable, remaining stable only when missing data is below roughly 30%, but falling below 50% under extreme degradation such as ≥60% missing data. These differences can be attributed to the nature of the tasks: fault location diagnosis focuses on the spatial distribution and inter-node relationships of electrical features across the network, and therefore requires sufficient measurements from multiple nodes around the faulted area to maintain spatial observability; fault type classification mainly relies on the characteristic patterns of three-phase and zero-sequence currents, which are global fault signatures and remain distinguishable even when a large portion of measurements is missing; in contrast, protection failure detection depends primarily on protection-related alarm and event information, and any loss of such alarm signals is likely to directly result in misdiagnosis, making this task inherently more sensitive to missing data. Overall, the observations indicate practical robustness thresholds of approximately 60% sampling loss or 40% sensor failure for fault location, approximately 90% sampling loss or 40% sensor failure for fault type classification, and roughly 30% missing data for reliable protection-failure detection.

……

Supplementary Information

Supplementary Table 9: Diagnostic accuracy of the proposed model under increasing sampling-loss and sensor-failure conditions

Test dataset	Fault location accuracy (%)	Fault type classification accuracy (%)	Protection failure detection accuracy (%)
Sampling loss 10%	97.35	100	88.15
Sampling loss 20%	96.79	99.97	84.01
Sampling loss 30%	96.32	99.92	80.62
Sampling loss 40%	93.19	98.33	69.43
Sampling loss 50%	92.16	98.41	60.62
Sampling loss 60%	90.72	98.53	50.64
Sampling loss 70%	86.38	98.66	40.13
Sampling loss 80%	80.23	97.63	26.12
Sampling loss 90%	63.21	95.4	13.57
Sensors fail 10%	97.25	99.64	90.26
Sensors fail 20%	96.61	98.87	89.2
Sensors fail 30%	95.48	97.63	87.17
Sensors fail 40%	89.33	91.18	78.61
Sensors fail 50%	84.68	86.38	73.52

Sensors fail 60%	78.17	78.33	65.14
Sensors fail 70%	70.72	68.64	58.51
Sensors fail 80%	59.43	49.23	45.14
Sensors fail 90%	46.97	29.61	33.93

Q19. On page 18 - line 388: the authors mention integrating environmental and visual data. Explain better how these would be fused into the existing GAT/self-attention framework—would additional pretraining be required, and what data sources do you envision?

A19. We sincerely thank the reviewer for raising this important question. The proposed multimodal diagnostic framework was intentionally designed to be modular and easily extensible, making it well-suited for integrating additional modalities such as environmental measurements or visual data.

In the current architecture, each modality—alarm signals, SCADA measurements, and waveform features—is processed through an independent feature-extraction branch consisting of a modality-specific embedding layer followed by a GAT-based encoder. The GAT encoders all share the same topology graph derived from the substation's primary equipment. This means that introducing a new modality does not require modifying the GAT structure, only the embedding layer needs to be designed to convert the raw input into node-aligned features. This design naturally accommodates visual or environmental data, because device-level video streams or environmental sensors (e.g., temperature, vibration, humidity) can be associated with specific equipment nodes in the same topology graph.

Incorporating visual data—for example, RGB images or video streams collected by substation inspection robots—would involve adding a new feature-extraction branch with an appropriate embedding network, such as CNNs, 3D CNNs, or Vision Transformers (ViT). The resulting embeddings would then pass through the same topology-aware GAT encoder and be fused with the existing modalities via the self-attention module. Because visual features differ substantially from electrical measurements, additional pretraining is required to initialize the visual encoder and align its embedding distribution with the existing latent space.

Regarding data sources, practical deployment would rely on inspection robots that routinely capture panoramic or device-level video for equipment monitoring. To support supervised multimodal training, a visual dataset can also be generated via the digital twin: a 3D substation model can be rendered synchronously with electromagnetic simulations in CloudPSS, producing temporally aligned visual scenes and physical states. This ensures semantic consistency between video data and the simulated fault processes, enabling seamless integration of visual or environmental modalities into the existing GAT/self-attention framework without redesigning the overall architecture.

The corresponding revisions in the Manuscript:

Discussion (Line 416–420)

……we plan to extend the multi-modal fusion capability by integrating visual data collected from

substation inspection robots. This will provide complementary spatial and contextual information for enhanced situational awareness. The visual data can be incorporated into the existing framework by adding a visual feature extraction branch parallel to the existing input encoders. This extension will require additional DAE pretraining to adapt visual features to the shared latent space. To support training, visual datasets will be generated from a 3D digital model of the substation, dynamically linked with the electromagnetic simulation in CloudPSS to ensure physical and temporal consistency between visual scenes and system states.

Q20. Under Appendix, the BFS-based fault enumeration algorithm is outlined, but providing a sample implementation would be ideal so readers could replicate author's training data generation process.

A20. We sincerely thank the reviewer for this helpful suggestion. We fully agree that providing a concrete example of the BFS-based fault enumeration procedure greatly improves the reproducibility and clarity of the dataset-generation pipeline. In the revised manuscript, we have added a complete illustrative example in the Appendix (***Supplementary Methods***) to demonstrate the entire traversal process.

The new example walks through a phase-to-phase short-circuit fault occurring at the low-voltage side of Transformer #1 in the substation shown in ***Supplementary Fig. 2a***. The example constructs a fault-enumeration tree starting from the base case (all relays available), identifies which protections operate (PR1 and PR2), and then systematically generates child nodes by blocking these protections in all combinations. For each generated node, a new time-domain simulation is executed under the same fault and topology, and the protection actions observed (e.g., PR3 or PR4 operations) determine the next level of child nodes in the BFS queue. The example traces the enumeration process across multiple levels, showing how blocking PR1 and PR3 causes fault propagation to the 110 kV side, thereby triggering PR4, and how the traversal terminates once deeper blocking combinations produce no new relay actions.

This illustrative example demonstrates how the BFS-based method systematically explores all feasible combinations of protection relay failure while avoiding redundant simulations. Starting from the fully functional scenario as the root node, each successive level represents incremental protection-failure combinations, enabling the algorithm to exhaustively enumerate all meaningful secondary-system failure modes for a given fault and topology. The added example provides sufficient detail—relay states, blocking logic, BFS queue evolution, and termination criteria—to allow readers to replicate our procedure in their own digital-twin environments.

Furthermore, we have released a CloudPSS simulation project file containing the joint primary-secondary digital twin model used for data generation (***Supplementary Software***). This allows readers to directly reproduce fault simulations, verify the enumeration process, and extend the dataset if needed.

The corresponding revisions in the Supplementary Information:

Supplementary Methods

Fault enumeration tree traversal method based on breadth-first search

……

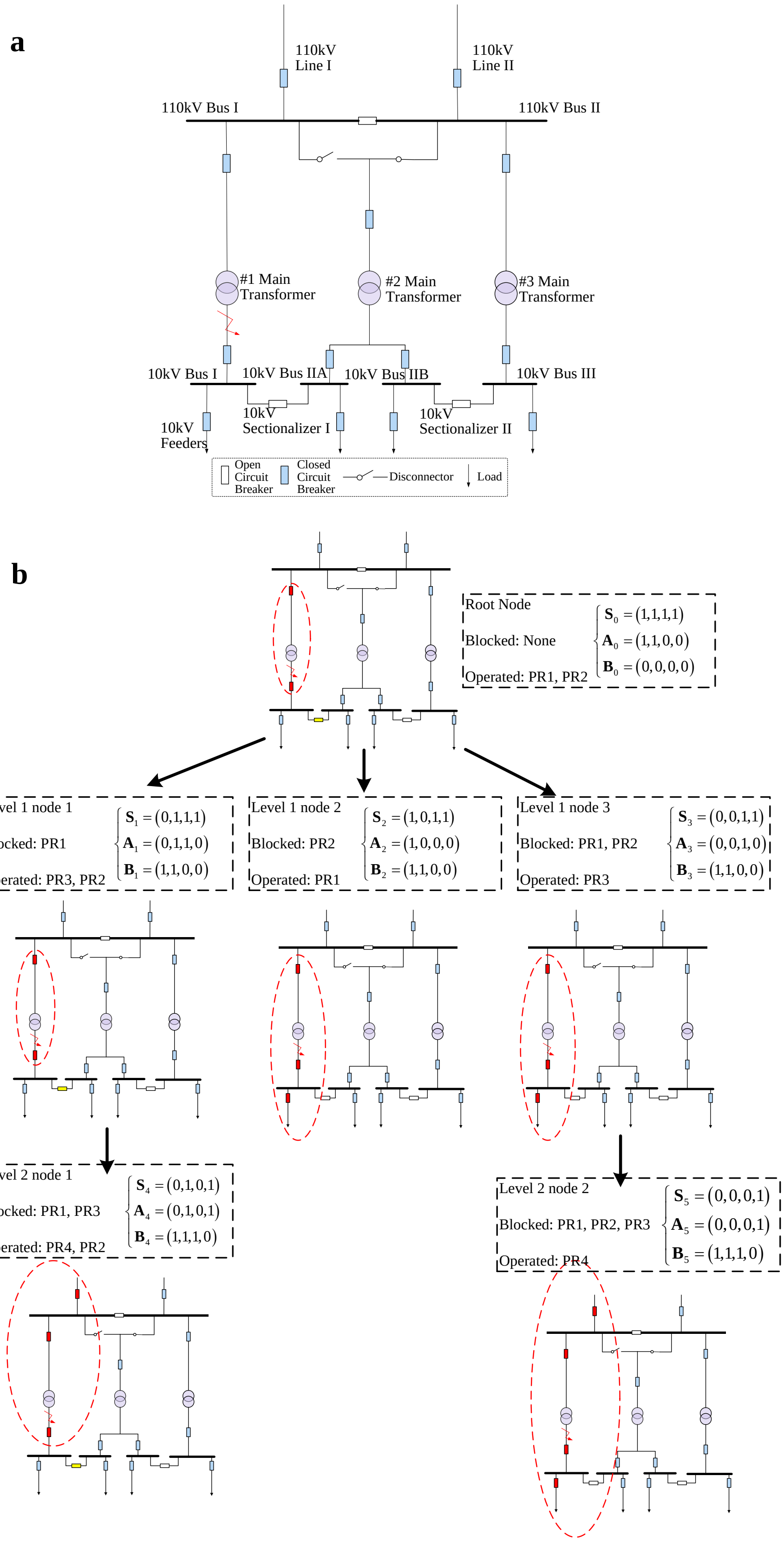

An illustrative example of the proposed fault enumeration tree traversal method is built on the substation shown in Supplementary Fig. 2a, whose main protection configuration is summarized in Supplementary Table 2. A phase-to-phase short-circuit fault occurring on the low-voltage side of #1 main transformer is selected to demonstrate the fault enumeration tree traversal method based on BFS. Under the typical operating topology

in Supplementary Fig. 2a, a phase-to-phase short circuit is applied to the low-voltage side of #1 main transformer in the simulation model, with all protection devices in service. In the base case, the simulation results show that the longitudinal differential protection of #1 main transformer (denoted as PR1) operates first and trips both circuit breakers on its high-voltage and low-voltage sides. After a short delay, the 10 kV automatic transfer scheme (denoted as PR2) operates, closes the 10 kV sectionalizer II, and restores the supply to the 10 kV Bus III. This fault scenario, with all relays available, is taken as the root node of the fault enumeration tree. Since two protection devices (PR1 and PR2) operate in this scenario, the root node generates three first-level child nodes, corresponding to: (i) PR1 blocked, PR2 available; (ii) PR2 blocked, PR1 available; and (iii) both PR1 and PR2 blocked.

Next, all first-level nodes are traversed in a breadth-first manner, and for each node, a time-domain simulation is repeated under the same fault and topology, but with the specified relays blocked. For the first child node (PR1 blocked, PR2 available), the simulation shows that, after the fault occurs, the high-voltage side overcurrent protection of #3 main transformer (denoted as PR3) operates, tripping its associated circuit breakers. After a delay, PR2 still operates and closes 10 kV sectionalizer II, thereby restoring the supply to 10 kV Bus III. Because a new protection element (PR3) is involved in this scenario, a new child node is generated from this node by additionally blocking PR3 (i.e., PR1 and PR3 blocked, PR2 available).

For the second child node (PR2 blocked, PR1 available), the simulation results indicate that PR1 operates as in the base case, but PR2 does not. Consequently, 10 kV Bus III is not resupplied after the fault is cleared. No additional protections operate beyond PR1, so this node does not generate any further child nodes.

For the third child node (PR1 and PR2 both blocked), the simulation shows that PR3 operates after the fault, while PR2 remains blocked and 10 kV Bus III is not restored. Since a new relay (PR3) operates in this scenario, a new child node is again generated by additionally blocking PR3 (i.e., PR1, PR2, and PR3 all blocked).

After traversing all first-level nodes and analyzing their corresponding fault scenarios, the newly generated second-level nodes are appended to the BFS queue and evaluated in the same manner. For the first second-level node (PR1 and PR3 blocked, PR2 available), the simulation shows that the fault propagates to the 110 kV level. The distance protection stage III of 110 kV transmission line I (denoted as PR4) operates, tripping the line, and PR2 subsequently operates to close 10 kV sectionalizer II and restore the supply to 10 kV Bus III. For the second second-level node (PR1, PR2, and PR3 all blocked), the fault likewise propagates to the 110 kV system and PR4 operates, indicating a similar escalation of the fault to the transmission line.

From these second-level nodes, additional child nodes can in principle be generated by further blocking PR4 (e.g., PR1-PR4 all blocked). However, the subsequent simulations for these deeper nodes show no new protection operations, and the fault continues without any further relay response. As no additional relays are triggered and no new child nodes are created, the BFS traversal of the fault enumeration tree terminates at this point.

This example illustrates how the proposed BFS-based fault enumeration tree systematically explores all feasible combinations of relay failures (blocking scenarios) for a given fault and topology. Starting from the fully functional case as the root node, each layer represents an incremental blocking of protections observed in the parent scenarios, while the breadth-first traversal ensures that lower-order failure combinations (with fewer blocked relays) are examined first. The resulting set of scenarios provides a structured and comprehensive basis for analyzing fault propagation paths and protection malfunction behaviors in complex

substation systems.

Response to Reviewer #3

The manuscript with title "Digital twin-driven fault diagnosis of power components within substations by multi-modal fusion learning" presents a Graph Attention Network with self-attention to model power substations. The model uses alarm information, fault waveform recordings, and SCADA measurement data to mainly classify fault location, fault type and protection failure (ref Fig 8). A case study based on 110kV substation located in Shenzhen, China is carried out. In section 6.2, an ablation study was carried out for data loss. In general, the work is interesting and brings practical contributions. Some comments and questions that arise:

Q1. The introduction/literature review on digital twins relating to substations requires more explanation and linkage towards this manuscript. There are good number of literature in the field which could be easily found.

A1. We sincerely thank the reviewer for this valuable comment. We fully agree that the literature review related to digital-twin (DT) technologies in substations needed further elaboration and clearer linkage to the proposed framework. In response, we have expanded the ***Introduction*** to more comprehensively position our work within existing DT research and highlight its relevance to intelligent fault diagnosis.

In the revised ***Introduction***, we now discuss that DT technology—defined as a high-fidelity virtual representation of physical systems coupled with real-time data and computational models—has enabled significant advances in condition monitoring, simulation, predictive maintenance, and autonomous operation in substations. Recent studies have demonstrated the applicability of DTs in substations for real-time fault monitoring and maintenance planning, as well as their capability to reproduce diverse fault scenarios and generate balanced datasets for data-driven diagnostic models. These works collectively show that DTs play a dual role: (i) providing a scalable simulation environment for generating labeled training data under varied operating conditions, and (ii) enhancing operational situational awareness through continuous synchronization with the physical system.

Building upon these developments, our work employs a DT of a real 110 kV smart substation to generate abundant and balanced fault samples that effectively address the data-scarcity challenge inherent in data-driven diagnosis. At the same time, the DT environment provides a practical platform for flexible model deployment and online updating, which enhances the scalability and engineering applicability of the proposed multimodal diagnostic framework.

The corresponding revisions in the Manuscript:

Introduction (Line 62-74)

……

Digital Twin (DT) technology provides a promising pathway to address several inherent limitations of existing fault diagnosis approaches. As a high-fidelity virtual representation of the physical substation that integrates real-time operational data with computational models, a DT enables intelligent monitoring, simulation, and optimization of system behaviors[29,30]. In current substation practice, DTs have already been applied to enhance real-time fault monitoring, fault localization, and predictive maintenance across both

primary and secondary systems[31-33]. By simulating diverse and realistic fault scenarios, DTs can generate abundant, balanced, and topology-aware training samples to mitigate the data scarcity and class imbalance commonly encountered in data-driven methods[34]. Moreover, the ability of DTs to provide consistent modeling of topology, device interactions, and protection logic greatly facilitates the alignment and fusion of heterogeneous data. Additionally, DTs support flexible model deployment and online updating, and their efficient data interaction within a coherent cyber–physical environment enables timely access to multi-source information, thereby enhancing the practicality, adaptability, and scalability of intelligent fault diagnosis solutions.

……

Q2. Referring to Fig 4., the multi-modal fusion applies a direct mid-level fusion on the feature space (Z) of three GATs. The fusion approach seems very straightforward, has other data fusion strategy been explored? What is the rationale on selecting this option as oppose to other data fusion strategy?

A2. In the proposed framework, alarm, SCADA, and waveform data are first encoded independently using three topology-aware GAT encoders. Although their outputs are organized in a concatenated form, the true fusion process is subsequently realized through a self-attention fusion module. This module learns adaptive, task-specific importance weights across modalities by explicitly modeling inter-modal correlations, enabling the network to automatically emphasize informative modalities while suppressing noisy or inconsistent ones.

The rationale behind choosing this mid-level self-attention fusion strategy is twofold. First, different diagnostic tasks rely on different modalities in varying degrees—for example, waveform and SCADA data dominate primary-system fault location and fault-type classification, whereas alarm information plays a more critical role in identifying secondary protection failures. Self-attention allows the fusion module to dynamically adjust modality contributions rather than relying on static rules or equal weighting. Second, compared with early fusion (which mixes raw inputs) or late fusion (which combines independent predictions), mid-level fusion with attention preserves modality-specific feature expressiveness while enabling effective cross-modal interaction, yielding both flexibility and robustness.

The effectiveness of this design is also empirically validated. As shown in our ablation study, removing the self-attention fusion module results in a notable degradation in performance, confirming that the proposed adaptive fusion mechanism is essential for capturing cross-modal dependencies, enhancing discriminability, and improving robustness under noisy or missing data.

Q3. In section 6.2, the comparison between the proposed method and the selected alternative method is limited. It is still lacking on more comparisons, for example, authors can consider deep learning techniques based on LSTM architecture, residual network etc.

A3. We sincerely thank the reviewer for this valuable suggestion. We fully agree that the original comparison was insufficient and have therefore substantially expanded the benchmark experiments to include a broader set of representative deep-learning methods, including LSTM-based and ResNet-based architectures as suggested by the reviewer.

In the revised manuscript, we now evaluate **seven baseline models**:

- TCFPN—a rule-based Petri-Net-based diagnosis model relying solely on alarm information, representing classical expert-system solutions used in substations.
- MPNN—a topology-aware, graph-based data-driven method that represents state-of-the-art approaches.
- Five deep-learning baselines: MLP-based, ResNet-based, LSTM-based, GCN-based, and Transformer-based architectures.

To ensure strict fairness, each deep-learning baseline was implemented by replacing only the GAT module in our framework while keeping all other components (data inputs, loss functions, optimization settings, and training procedures) unchanged. Hyperparameters and parameter counts were adjusted to maintain comparable model complexity across all baselines (details provided in Supplementary Table 5).

All models were tested on ten test datasets that simulate realistic disturbances in substation environments, such as noise, sampling loss, and sensor failures. Performance was evaluated for all three diagnostic subtasks. Accuracy results are presented in the main text, while precision, recall, and F1-scores are provided in the ***Supplementary Data***. To assess model stability, each experiment was repeated under five random seeds, and results are reported as mean ± standard deviation.

Across all baseline methods and under all disturbance conditions, the proposed model consistently achieves the highest diagnostic accuracy and demonstrates superior robustness (Tables 1–3, Fig. 1d–f, Supplementary Fig. 3–5). These results confirm the effectiveness and generalization capability of the proposed multimodal, topology-aware diagnostic framework.

The corresponding revisions in the Manuscript:

Results (Line 184–235)

Comparison of fault diagnosis models

……

Seven baseline models are implemented for performance comparison. Temporal constrained fuzzy Petri net (TCFPN)[12] is a rule-based approach that relies solely on alarm information to identify fault locations and protection failures. It builds upon an improved Petri Net–based framework and represents a typical rule-based

solution for substation fault diagnosis. However, the model requires manual updates whenever the system topology changes. The MPNN[21] model is a data-driven method that utilizes system topology and fault waveform data to predict fault locations and types. Although originally designed for microgrid fault diagnosis, its modeling framework can be readily extended to substations and represents a state-of-the-art graph-based data-driven approach. The MLP-based, ResNet-based, LSTM-based, GCN-based, and Transformer-based models are constructed by replacing the GAT module in the proposed framework with the corresponding architectures, representing common deep learning baselines. For fair comparison, all data-driven models are trained under identical optimization settings and data inputs, and their hyperparameters and parameter counts are adjusted to maintain comparable model complexity (Supplementary Table 5,6). While the proposed model does increase the training time compared to some baselines, the overall training and inference costs remain reasonable, without introducing significant overhead relative to other deep learning models (Supplementary Table 7).

All models are evaluated on ten test datasets corresponding to different data degradation conditions, which emulate practical disturbances in communication and measurement commonly encountered in substations. For quantitative evaluation, three diagnostic subtasks are assessed independently. Accuracy is used as the primary performance metric in the main text, while additional metrics, including precision, recall, and F1-score on both training and test datasets, are provided in the supplementary material (Supplementary Data). Each experiment is repeated under five random seeds, and results are reported as mean ± standard deviation to evaluate model stability.

The proposed model consistently outperforms all baseline approaches under varying levels of noise, sampling loss, and sensor failure (Tables 1–3, Fig. 1d–f, Supplementary Fig. 3–5, Supplementary Data).

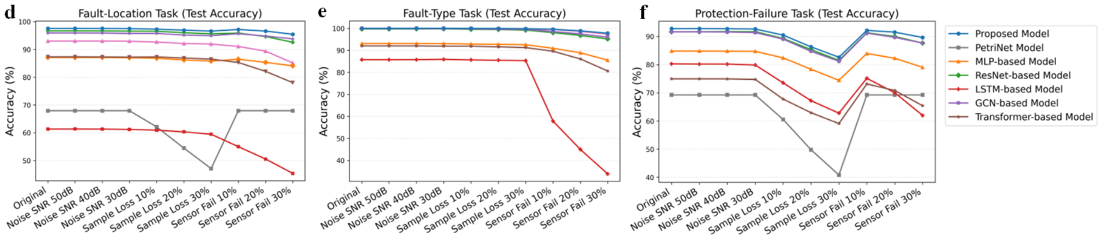

TCFPN relies solely on alarm information; however, multiple fault scenarios can produce highly similar alarm patterns, and the rule-based inference cannot resolve these ambiguities. As a result, its outputs remain largely invariant across some different test conditions, reflecting the limited discriminative capacity of alarm-only reasoning.

MPNN utilizes system topology and fault waveform recordings, enabling competent performance in fault-location and fault-type identification. However, because it does not incorporate alarm information, it cannot diagnose protection failures. In addition, MPNN is trained in a single end-to-end manner rather than following the staged training strategy adopted in the proposed framework, resulting in less effective feature extraction and, consequently, lower diagnostic accuracy. This training scheme also limits its robustness when the input data are noisy, incomplete, or corrupted.

LSTM-based models emphasize temporal correlations and are not well-suited for capturing spatial or topological interactions, leading to reduced robustness when the input topology changes. The MLP-based and Transformer-based variants, though capable of strong representation learning when sufficient data are available, treat inputs as flattened or tokenized sequences and therefore cannot explicitly leverage the

substation's graph structure. This limits their ability to learn inter-node dependencies, resulting in weaker generalization and reduced robustness under topology variations.

ResNet-based and GCN-based models partially address spatial feature learning—ResNet through 2D convolutions on processed inputs and GCN through graph convolutions. Both achieve relatively strong performance; however, their ability to capture the underlying topological relationships is limited. ResNet operates on Euclidean grids and cannot represent non-uniform electrical connectivity, while GCN performs homogeneous graph convolutions that cannot adaptively weight critical nodes. These limitations make them less effective than GAT in modeling the directional and heterogeneous dependencies among substation components. In contrast, the proposed GAT-based fusion mechanism performs adaptive, node-wise attention and integrates topology, waveform, SCADA, and alarm information more effectively, yielding the highest overall accuracy.

……

Q4. Fair selection of alternative model hyperparameters: The selection of hyperparameters in the CNN layer is also not mentioned for study, which could significantly affect the performance. Such hyperparameter exploration will also be significant for other alternative models.

A4. We sincerely thank the reviewer for this important comment. We fully agree that fair hyperparameter selection is essential to ensure unbiased comparison across all baseline models.

In this study, for all alternative architectures—including the MPNN-based, MLP-based, ResNet-based, LSTM-based, GCN-based, and Transformer-based models—we adjusted key structural hyperparameters (e.g., number of layers, convolution kernel sizes, hidden feature dimensions, attention heads, and dropout rates) within reasonable configuration ranges to ensure that the total number of learnable parameters of each baseline model is comparable to that of the proposed method. This design choice ensures that performance differences truly reflect representational capability rather than discrepancies in model size.

All detailed hyperparameters and the exact number of learnable parameters for every baseline model are provided in **Supplementary Table 6**, and the complete configurations can also be directly inspected in the submitted source code, ensuring full transparency and reproducibility.

The corresponding revisions in the Supplementary Information:

Supplementary Table 6: Architecture and parameter counts of all baseline models

Model	Encoder architecture	Encoder learnable parameters (Millions)	Decoder architecture	Decoder learnable parameters (Millions)	Self-attention layer learnable parameters (Millions)	MLP Classifiers learnable parameters (Millions)	Total learnable parameters (Millions)
MPNN	2-layer Conv1d + MLP + 4-layer	59.89	N/A	N/A	N/A	0.85	60.74

	MPNN						
MLP-based	3-branch × 3-layer MLP	2.3	3-layer MLP	3.54	12.59× 3	3.22× 3	53.15
ResNet-based	3-branch × 3-layer Conv2d ResNet	2.22	3-layer ConvTranspose2d ResNet	3.68	12.59× 3	3.22× 3	53.21
LSTM-based	3-branch × 3-layer LSTM	2.05	3-layer LSTM	3.41	12.59× 3	3.22× 3	52.77
GCN-based	3-branch × 3-layer GCN	1.94	3-layer GCN	3.55	12.59× 3	3.22× 3	52.60
Transformer-based	3-branch × 3-layer Transformer Encoder	2.5	3-layer Transformer Decoder	3.59	12.59× 3	3.22× 3	53.4
Proposed	3-branch × 3-layer GAT	1.92	3-layer GAT	3.3	12.59× 3	3.22× 3	52.53

Q5. In figure 14, author performed t-SNE on data relating to the model. The classes are labelled from 0-15 on the top figures, and 0-9 on the bottom figures, however I do not find explanation of such classes. This needs to be properly explained.

A5. We sincerely thank the reviewer for pointing out this missing clarification. The class indices in the t-SNE visualizations indeed correspond to two different diagnostic tasks, and we agree that this should be made explicit.

In the revised manuscript, we clarify that Fig. 2d–f (formerly Fig. 14 upper row) shows the learned embeddings for the fault location task, which includes 16 classes representing the 16 physically defined fault locations in the substation. Therefore, labels 0–15 correspond to these 16 fault-location categories.

Fig. 2g–i (formerly Fig. 14 lower row) shows the embeddings for the fault type classification task, which includes 10 classes: A/B/C single-phase grounding, AB/BC/CA two-phase short circuits, ABG/BCG/CAG two-phase-to-ground faults, and three-phase short circuits. Thus, labels 0–9 correspond to these 10 fault-type categories.

To avoid any ambiguity, we have updated the caption of Fig. 2 in the revised manuscript to explicitly describe the meaning of each class index in the t-SNE plots.

The corresponding revisions in the Manuscript:

Results (Line 244–256)

Ablation Study

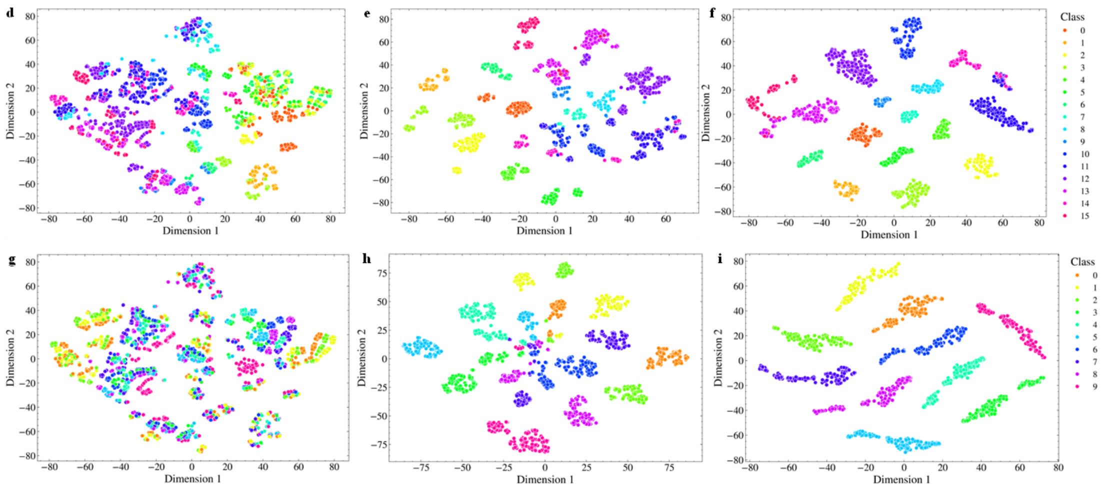

Figure 2. Ablation study results.…… **d** t-SNE visualization of the encoder-output feature embeddings for the fault-location classification task. **e** t-SNE visualization of the feature embeddings after self-attention (SA) fusion for the fault-location classification task. **f** t-SNE visualization of the feature embeddings after applying self-attention with supervised contrastive learning (SA+SCL) for the fault-location classification task. In panels **d**–**f**, labels 0–15 correspond to the 16 fault-location categories. **g** t-SNE visualization of the encoder-output feature embeddings for the fault-type classification task. **h** t-SNE visualization of the feature embeddings after SA for the fault-type classification task. **i** t-SNE visualization of the feature embeddings after SA+SCL for the fault-type classification task. In panels **g**–**i**, labels 0–9 correspond to the 10 fault-type categories. All t-SNE plots (**d**–**i**) are generated using identical hyperparameters (perplexity = 30, learning rate = 200, iterations = 1000). The use of self-attention improves cluster compactness, while the addition of supervised contrastive learning further enhances intra-class compactness and inter-class separability, leading to more structured and distinguishable feature clusters.

Q6. Relating to the previous point, t-SNE is a dimensionality reduction technique that is stochastic and relatively difficult to interpret (see Wattenberg et al. below). In manuscript line 345, the author interpreted the separation of clusters within T-SNE. This is not quite appropriate for T-SNE, as distances between clusters might not mean anything. *Wattenberg, M., Viégas, F. and Johnson, I., 2016. How to use t-SNE effectively. Distill, 1(10), p.e2.*

A6. We sincerely thank the reviewer for raising this important methodological point. The reviewer correctly notes that t-SNE is a stochastic dimensionality-reduction technique and that interpreting cluster distances directly can be misleading, as highlighted by Wattenberg et al. (2016). We appreciate this reminder and agree that additional clarification and quantitative evaluation are necessary.

In response, we have revised the manuscript to clarify the role of t-SNE and to avoid over-interpretation of visual cluster separation. In particular, the text now explicitly states that t-SNE is used solely for qualitative visualization and that no quantitative conclusions are drawn from distances in the 2D embedding. The revision also notes that t-SNE does not preserve global structure, following the recommendations in Wattenberg et al. (2016).

To provide rigorously interpretable evidence of cluster structure, we have supplemented the visualization with quantitative clustering metrics computed directly in the original latent space, including silhouette score, intra-class distance, inter-class distance, and inter/intra-class distance ratios. These metrics are now presented in **Table 4** and further detailed in the ***Supplementary Methods.***

The quantitative results consistently show that self-attention fusion improves feature compactness compared to the encoder output, and that incorporating SCL leads to further improvements—for example, in the fault-location task, the silhouette score increases from 0.15 (SA) to 0.76 (SA+SCL), and the inter/intra-class distance ratio rises from 1.35 to 7.43. Similar improvements are observed for the fault-type classification task. These numerical indicators provide reliable and interpretable evidence—independent of t-SNE—that SCL enhances the discriminability of the learned feature space.

The corresponding revisions in the Manuscript:

Results (Line 288–306)

Ablation study

……

To investigate how self-attention fusion and SCL influence the structure of the learned feature space, we visualize the embeddings using t-SNE with fixed hyperparameters (perplexity = 30, learning rate = 200, iterations = 1000) (Fig. 2d–i). It is important to note that t-SNE[45] is a nonlinear, locally preserving embedding method; therefore, the distances in its 2D projected space do not reflect the true geometry of the original high-dimensional feature space[46]. Consequently, t-SNE is used only for qualitative visualization, whereas all quantitative metrics are computed in the original feature space.

Table 4 Quantitative clustering metrics for multi-class feature embeddings under different model configurations

Task	Feature Representation	Silhouette Score	intra-class distance	inter-class distance	Inter/Intra
Fault location	Encoder	0.0269	4.5099	5.2125	1.1558
Fault location	SA without SCL	0.1548	109.4283	148.1606	1.3540
Fault location	SA with SCL	0.7600	15.7616	117.0393	7.4256
Fault type classification	Encoder	0.0009	4.9346	5.1503	1.0437
Fault type classification	SA without SCL	0.1697	89.6750	124.9386	1.3932
Fault type classification	SA with SCL	0.7309	17.0048	85.7115	5.0404

To rigorously evaluate clustering quality in the two multi-class tasks, several metrics explicitly supporting multi-class settings are adopted, including the silhouette score, intra-class distance, inter-class distance, and their ratio (Supplementary Methods, Supplementary Equation (3)–(11)). The quantitative results reveal consistent trends across both tasks (Table 4). Self-attention fusion improves cluster compactness compared with the encoder output, while introducing SCL further produces substantially more compact and better-separated feature clusters. For the fault location task, the silhouette score increases from 0.15 (SA) to 0.76 (SA+SCL), and the inter/intra-class distance ratio increases from 1.35 to 7.43. Similar improvements are

observed in the fault type classification task. These results demonstrate that SCL significantly enhances the discriminability of the latent feature space, enabling the model to more effectively separate fault categories.

Q7. In the Table 4, the alarm information seems supervised. What would happen if there were a fault that was not within the database or existing distributions?

A7. We sincerely thank the reviewer for raising this important question. The concern about the supervised nature of the alarm information and the model's behavior under previously unseen fault conditions is highly relevant to evaluating the robustness and practical reliability of the proposed system.

First, in practical substation engineering, the alarm set is finite, standardized, and explicitly defined in the SCD (Substation Configuration Description) file of the IEC 61850 system. Therefore, in real deployments, the system will not encounter entirely unknown alarm types outside this predefined set. Even when abnormal alarm patterns arise—such as due to communication noise or transient transmission errors—our alarm encoder incorporates a fuzzy Petri-net-based design that provides inherent noise tolerance and can filter out logically inconsistent or physically impossible alarm combinations. This mechanism ensures that random or spurious alarms will not mislead the diagnosis process.

Second, regarding unseen fault scenarios, it is true that any supervised model may face challenges when confronted with fault conditions that fall outside the distributions represented in the training data. Although the digital-twin-generated dataset systematically covers a broad range of realistic variations—including diverse topologies, fault locations, fault types, fault resistances, and protection-failure patterns—the model's generalization capacity is not unlimited. Rare or atypical fault behaviors, unconventional protection misoperations, or extreme sensor anomalies could still lead to degraded diagnostic accuracy. In this sense, the digital-twin data significantly reduce—but cannot fully eliminate—the risk associated with out-of-distribution operation.

To further mitigate this risk, we incorporate a confidence-based verification mechanism. For all three categories of fault-diagnosis tasks, the system outputs both the diagnostic decision and its corresponding confidence score. When the confidence score falls below 0.9, the system automatically marks the decision as low-confidence and prompts operator intervention. This mechanism ensures that ambiguous or potentially out-of-distribution cases are routed to human experts, thereby enhancing robustness, preventing misjudgment, and improving the trustworthiness of the system in real operational environments.

Q8. Relating to real-world application, most of the time, when data are missing in substations, they are rarely 10% or 20% missing, but fully missing due to sensor damages. Does the algorithm proposed deals with some variables being 100% missing? Perhaps authors can also justify the real-world potential relating to the ablation study of 10-50% missing data ratio. What happens when there are higher missing data ratio?

A8. We sincerely thank the reviewer for this insightful comment. Following the reviewer's suggestion, we have expanded our analysis to more comprehensively examine sensor-failure scenarios. In the revised manuscript, we explicitly include sensor-failure degradations in both the data-augmentation process and the newly added stress-test evaluation, which together provide a clearer understanding of the model's robustness under realistic substation conditions.

In real-world substations, measurement loss typically appears either as transient sampling interruptions or as complete channel failures caused by sensor malfunction. To ensure that the training data reflect these practical conditions, the digital-twin-generated dataset incorporates explicit data-degradation mechanisms. Specifically, Gaussian noise with SNR levels of 50, 40, and 30 dB is added to waveform and SCADA measurements; random sampling loss of 10%, 20%, and 30% of time-series samples is applied to alarm, SCADA, and waveform data; and complete sensor failures are simulated by dropping 10%, 20%, and 30% of entire SCADA or waveform channels (i.e., 100% missing for affected sensors). These augmentation strategies allow the model to learn noise-tolerant and failure-resilient representations and avoid dependence on any single measurement source.

To further assess the model's behavior under more extreme degradation, we conducted a comprehensive stress-test using nineteen degraded datasets with 10%-90% sampling loss and 10%-90% sensor failures. The results demonstrate that the three diagnostic tasks have distinct tolerance limits. Fault type classification is the most resilient, maintaining above 95% accuracy even with 90% sampling loss and above 90% accuracy under 40% sensor failures. Fault location shows moderate sensitivity: its accuracy remains above 90% when sampling loss is within 60% and above 85% when sensor failures are within 40%, but drops sharply beyond these levels (e.g., 63.2% at 90% sampling loss and 59.4% at 80% sensor failure). Protection-failure detection is the most vulnerable, remaining stable only when data loss is within roughly 30%, but falling below 50% under extreme degradation such as ≥60% missing data. These patterns arise from the nature of the tasks: fault type relies on global disturbance patterns that remain discernible under heavy data loss; fault location requires sufficient spatial observability around the faulted area; and protection-failure detection depends on fine-grained, device-level multi-label signals, making it inherently more sensitive to missing information. In summary, while the model can tolerate full loss of a subset of sensors—owing to the sensor-failure augmentation used during training—its performance inevitably degrades when a large proportion of sensors fail simultaneously. The stress-test therefore provides a realistic upper bound on missing-data robustness, indicating practical thresholds of approximately 60% sampling loss or 40% sensor failure for fault location, approximately 90% sampling loss or 40% sensor failure for fault type classification, and roughly 30% missing data for reliable

protection-failure detection.

The corresponding revisions in the Manuscript:

Results

Case setup (Line 149-154)

……

To enhance robustness and generalization, multiple data augmentation strategies are applied, including adding zero-mean Gaussian noise with signal-to-noise ratios (SNR) of 50, 40, and 30 dB; introducing random sampling loss by removing 10%, 20%, and 30% of time-series data points from alarm, SCADA, and waveform data; and simulating sensor failures by randomly dropping 10%, 20%, and 30% of entire SCADA or waveform channels. These augmentations generate nine additional datasets, which, together with the original data, result in a total of 115,970 training samples.

……

Digital twin application platform (Line 347-366)

……

……the robustness of the diagnostic model under incomplete measurements was evaluated through a stress test using nineteen datasets with 10%–90% sampling loss and 10%–90% sensor failures (Supplementary Table 8). The results show that the three diagnostic tasks have distinct tolerance limits. Fault type classification is the most resilient, maintaining above 95% accuracy even with 90% sampling loss and above 90% accuracy under 40% sensor failures. Fault location is moderately sensitive: its accuracy stays above 90% when sampling loss is within 60% and above 85% when sensor failures are within 40%, but drops sharply beyond these levels (e.g., 63.2% at 90% sampling loss and 59.4% at 80% sensor failure). Protection failure detection is the most vulnerable, remaining stable only when missing data is below roughly 30%, but falling below 50% under extreme degradation such as ≥60% missing data. These differences can be attributed to the nature of the tasks: fault location diagnosis focuses on the spatial distribution and inter-node relationships of electrical features across the network, and therefore requires sufficient measurements from multiple nodes around the faulted area to maintain spatial observability; fault type classification mainly relies on the characteristic patterns of three-phase and zero-sequence currents, which are global fault signatures and remain distinguishable even when a large portion of measurements is missing; in contrast, protection failure detection depends primarily on protection-related alarm and event information, and any loss of such alarm signals is likely to directly result in misdiagnosis, making this task inherently more sensitive to missing data. Overall, the observations indicate practical robustness thresholds of approximately 60% sampling loss or 40% sensor failure for fault location, approximately 90% sampling loss or 40% sensor failure for fault type classification, and roughly 30% missing data for reliable protection-failure detection.

……

Methods (Line 584-599)

Data generation

……

To further enhance the realism and robustness of the dataset, multiple data augmentation strategies are applied to the simulated samples, ensuring that the training data better reflect real-world operating conditions and mitigating the risk of model overfitting. Three augmentation mechanisms are designed. First, random Gaussian noise is added to both fault waveform and SCADA measurements to emulate environmental disturbances, sensor precision limitations, and communication noise commonly observed in field deployments. This enables the model to learn noise-tolerant representations and maintain diagnostic reliability under uncertain signal conditions. Second, sampling-loss simulation is performed by randomly removing a subset of time-series data points at each measurement node. This mimics transient communication delays or temporary signal interruptions, compelling the model to infer missing information and improving its resilience to incomplete measurements. Third, sensor-failure simulation is introduced by completely eliminating the measurements from selected sensors to represent sensor malfunctions or disconnections. This ensures that the model remains functional and reliable even when partial sensing devices fail in practice. Finally, the augmentation pipeline is designed to be extensible: real fault cases occurring during actual operation are archived and later incorporated into the framework for fine-tuning and iterative model enhancement. This enables the model to progressively adapt to real operating environments as more field data become available.

……

Q9. In Figure 12, it might be useful to label the nodes in the topology to demonstrate what these nodes represent.

A9. We sincerely thank the reviewer for this constructive suggestion. We agree that explicitly labeling the nodes in the topology diagrams is important for improving interpretability and helping readers understand the physical meaning of each node in the diagnostic graph.

In response, we have revised the figure caption of the corresponding topology figure (now Fig. 1 in the revised manuscript) to clearly describe what each node represents. Specifically, all 16 nodes are now explicitly mapped to their associated primary-equipment locations within the 110 kV substation.

The corresponding revisions in the Manuscript:

Results (Line 99-116)

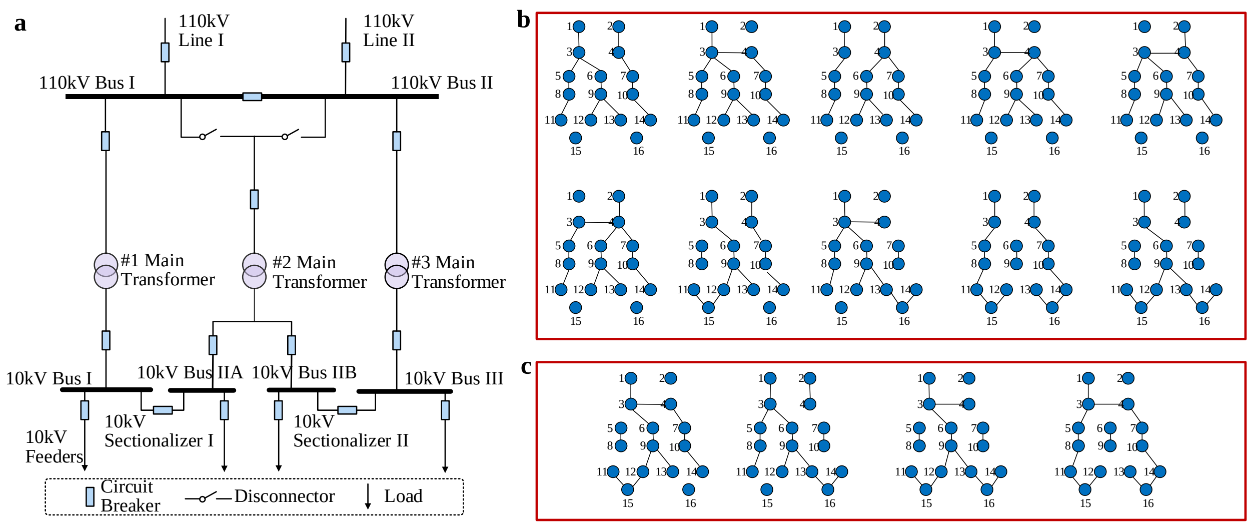

Figure 1. Substation layout, topology variations, and model performance across diagnostic tasks. a Overall layout of the studied substation. The system comprises two 110kV incoming lines, two 110kV busbars, three main transformers with both high- and low-voltage terminals, four 10kV busbars, and two 10kV sectionalizer switches, defining 16 physically meaningful fault locations within the primary network. **b** The ten topology configurations used for model training. Each graph contains 16 annotated nodes representing the same set of primary-system fault locations—Nodes 1-2: 110 kV Lines I and II; Nodes 3-4: 110 kV Buses I and II; Nodes 5-7: high-voltage terminals of Main Transformers #1-#3; Nodes 8-10: low-voltage terminals of Main Transformers #1-#3; Nodes 11-14: 10 kV Buses I, IIA, IIB, and III; and Nodes 15-16: 10 kV Sectionalizers I and II. **c** The four topology configurations reserved for testing, designed to introduce previously unseen structural variations and thereby assess the generalization ability of the diagnostic models.

Q10. Although the model is of a general deep learning framework, its deployability to other substations/case studies will still require effort. I do think authors should discuss such aspects together with further potential for improvement or wider application.

A10. We sincerely thank the reviewer for raising this important point regarding the deployability and transferability of the proposed framework to other substations. We agree that applying the method to new substations requires additional engineering steps, and it is valuable to clearly articulate these practical considerations and the potential for broader application.

In the revised manuscript, we have expanded the ***Discussion*** to explain the deployment workflow and the associated workload. Deploying the framework to another substation mainly involves: (1) constructing or adapting that substation’s digital twin model, including its primary equipment and secondary protection configuration; (2) generating large-scale fault scenarios using the adapted digital twin; and (3) performing model training, validation, and calibration based on the newly generated dataset. Owing to the high level of standardization in smart substations following IEC 61850 and common design specifications, most components of the simulation model can be directly reused. Only a small set of electrical parameters, topology details, and protection settings need to be modified for a new site. With the CloudPSS simulation platform, these adjustments can be efficiently executed, enabling rapid generation of representative training data and reducing engineering overhead. Therefore, while some adaptation is necessary, the overall framework

exhibits strong transferability and practical scalability across substations with different configurations.

We have added a dedicated discussion in the ***Discussion*** section to elaborate on these aspects and to highlight the framework's potential for wider application and future improvements.

The corresponding revisions in the Manuscript:

Discussion (Line 404-411)

……

The proposed framework also exhibits strong transferability across substations. When applied to a new substation, the main tasks include constructing or adapting its simulation model, generating batch training data, and performing model retraining and validation. Owing to the high standardization of smart substation designs, most existing digital twin models can be reused with only minor adjustments to electrical parameters and protection configurations. With the CloudPSS platform, large-scale training datasets can be efficiently generated, enabling rapid adaptation of the fault diagnosis model to new substations. Thus, the proposed framework offers a scalable and generalizable solution for smart substation fault diagnosis and maintenance.

……

Q11. (Remarks on code availability): The code was reproducible on codeocean. There is a README document that explains the code.

A11. We sincerely thank the reviewer for carefully checking the CodeOcean capsule and confirming the reproducibility of the code. We appreciate this positive feedback and have further improved the organization and clarity of the released implementation.

In particular, we have reorganized the project structure, refined the module naming, and expanded the README documentation to provide clearer instructions on dataset preparation, parameter configuration, model training, and fault scenario generation. Additional comments have been added to key scripts to enhance readability and ease of modification for users who wish to adapt the workflow to other substations.

These updates ensure that the codebase is more accessible and user-friendly, allowing researchers and engineers to more easily reproduce our results or extend the framework for their own applications.

The computer code is available via Code Ocean at: https://doi.org/10.24433/CO.6548109.v3

Response to Reviewer #4

The paper proposes a novel fault diagnosis framework that integrates digital twin technology with graph attention networks and self-attention mechanisms to enhance diagnostic accuracy and robustness under diverse conditions. The detailed reviewer comments are provided below.

Q1. The literature review is informative but somewhat broad, as it mainly covers general fault diagnosis methods in power systems. It would be stronger if the review focused more specifically on fault detection and diagnosis in substations. For example, Jiang et al. (2024) proposed a busbar fault diagnosis method based on multi-source fusion, which is directly relevant to the current study but not cited. Including such works would better position the contribution of this paper within the state-of-the-art. *[A] Jiang X, Cao H, Zhou C, Ren X, Shen J and Yu J (2024) Busbar fault diagnosis method based on multi-source information fusion. Front. Energy Res. 12:1443570. doi: 10.3389/fenrg.2024.1443570.*

A1. We sincerely thank the reviewer for this insightful suggestion. We fully agree that the original literature review was too broad and mainly covered general fault diagnosis methods in power systems. In response, we have substantially strengthened the Introduction by adding a focused review of substation-oriented fault diagnosis methods, especially those related to multi-source fusion, secondary-system analysis, and smart-substation scenarios.

In the revised manuscript, we expanded the literature review to highlight representative approaches specifically developed for substation fault detection and diagnosis, including Bayesian network-based hybrid reasoning models, Petri-net methods for locating faults and identifying protection misoperations, rough-set-based models for handling uncertain alarm information, and several emerging data-driven approaches for smart substations.

Most importantly, following the reviewer's recommendation, we have explicitly included and discussed the directly relevant work by Jiang et al. (2024) on multi-source busbar fault diagnosis. This work combines time- and frequency-domain features using Dempster-Shafer evidence theory and BP neural networks. While highly relevant to our study, it focuses exclusively on busbar faults and does not incorporate alarm signals or extend to transformer, line, or protection-failure scenarios. We emphasize this distinction to better position the novelty of our proposed fully multimodal framework covering both primary- and secondary-system faults.

The corresponding revisions in the Manuscript:

Introduction (Line 48-61)

……

For substation fault diagnosis, traditional rule-based approaches are widely applied, and many studies have attempted to enhance them with more flexible reasoning mechanisms. For example, researchers have

introduced a Bayesian network-expert system hybrid to enhance coordinated analysis of primary and secondary systems[22], a Petri net-based method to support integrated fault identification and protection verification[23], and a rough set-based bio-inspired model that combines rough set reduction with spiking neural P systems to better handle uncertainty in alarm information[24]. With increasing data availability, research has gradually shifted toward data-driven methods. Recent work includes deep reinforcement learning models that learn fault patterns from historical data[25], as well as ANN- and RNN-based approaches that leverage communication, protection, and secondary system data for fast diagnosis and improved fault localization[26,27]. These data-driven studies, however, primarily focus on the secondary systems of smart substations. Beyond these developments, multi-source information fusion has also been investigated. One representative study combines time-frequency features with Dempster-Shafer evidence theory and BP neural networks for busbar fault diagnosis[28]. However, this method focuses solely on busbar faults and does not incorporate alarm information, thereby limiting its diagnostic scope.

……

Q2. The evaluation is restricted to a single 110 kV substation. Broader validation across substations with different voltage levels, configurations, and operational conditions would strengthen claims of generalization.

A2. We sincerely thank the reviewer for this valuable comment. We fully agree that broader validation across substations with different voltage levels, configurations, and operational environments would further strengthen the generalization claims of the proposed method.

At present, our evaluation is limited by practical data availability. The 110 kV smart substation used in this study is the only site for which we have full project-based access to the complete digital-twin configuration, including the primary system topology, equipment parameters, secondary protection settings, and the IEC 61850 SCD file. Constructing a high-fidelity digital twin for additional substations requires these detailed engineering data, which are generally confidential and not publicly accessible; therefore, we currently lack authorization to obtain comparable datasets from other substations.

Nevertheless, the proposed digital-twin-driven multimodal framework is not inherently limited to 110 kV systems. Because it relies on standardized IEC 61850 data sources, it can be directly transferred to substations of other voltage levels (e.g., 220 kV, 500 kV) provided that these substations adopt the IEC 61850 communication standards and their corresponding IEC 61850 data and configuration files are available.

Furthermore, we are currently collaborating with industry partners to extend this framework to higher-voltage AC substations and HVDC converter stations, where digital-twin models and multimodal data acquisition are being constructed. These ongoing projects will enable broader cross-station validation in future work.

The corresponding revisions in the Manuscript:

Results (Line 118-121)

Case setup

The case study and engineering validation are based on a real 110 kV smart substation located in Shenzhen, China (Fig. 1a), which operates in full compliance with the IEC 61850 standard (Supplementary Notes). The proposed method, however, is equally applicable to substations at other voltage levels (e.g., 220 kV, 500 kV) that adopt the IEC 61850 communication standards.

……

Q3. The paper addresses robustness against missing data but does not explicitly analyze the effect of measurement noise on diagnostic accuracy. Considering that measurement noise is inevitable in SCADA systems and waveform recorders, an explicit evaluation of noise robustness would strengthen the practical relevance of the results.

A3. We sincerely thank the reviewer for this insightful comment. The reviewer is absolutely correct that measurement noise is unavoidable in practical substations, especially in SCADA measurements and fault waveform recordings. Evaluating noise robustness is therefore essential to ensure the practical value and real-world reliability of the proposed method. We appreciate the opportunity to clarify and strengthen this aspect of the study.

In the revised manuscript, we explicitly analyze the effects of measurement noise through systematic noise-augmentation experiments. During dataset generation, zero-mean Gaussian noise with multiple signal-to-noise ratios (SNRs) was introduced into SCADA signals and fault waveform data to emulate realistic disturbance levels observed in substation environments. Specifically, we considered SNR levels of 50 dB, 40 dB, and 30 dB, which correspond to common ranges reported for high-quality SCADA sensors and digital fault recorders. These noise perturbations were applied independently and in combination with missing-data scenarios to evaluate the robustness of the proposed multimodal fusion model under multiple real-world uncertainties.

The experimental results, detailed in the revised ***Results*** section and in ***Supplementary Data***, demonstrate that the proposed method exhibits strong resilience to measurement noise. Across all evaluated SNR levels, the diagnostic performance in fault location, fault type classification, and protection failure detection shows only marginal degradation compared to the noise-free baseline. These findings confirm that the denoising autoencoder (DAE) initialization and the multimodal self-attention fusion jointly contribute to stable performance under noisy measurement conditions, thereby enhancing the engineering applicability of the proposed framework.

The corresponding revisions in the Manuscript:

Results

Case setup(Line 149-154)

……

To enhance robustness and generalization, multiple data augmentation strategies are applied, including adding zero-mean Gaussian noise with signal-to-noise ratios (SNR) of 50, 40, and 30 dB; introducing random

sampling loss by removing 10%, 20%, and 30% of time-series data points from alarm, SCADA, and waveform data; and simulating sensor failures by randomly dropping 10%, 20%, and 30% of entire SCADA or waveform channels. These augmentations generate nine additional datasets, which, together with the original data, result in a total of 115,970 training samples.

……

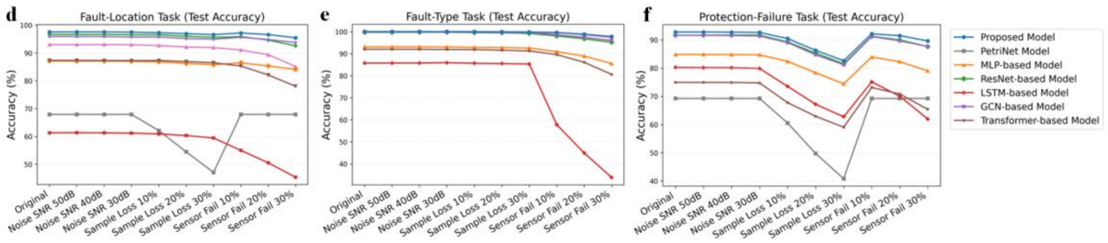

Figure 1. ……diagnostic model performance under diverse test conditions. ……d Model performance on the fault-location task under a range of test conditions. Including the original dataset, noise-corrupted datasets (SNR = 50, 40, and 30 dB), sampling-loss conditions (10%, 20%, and 30%), and sensor-failure conditions (10%, 20%, and 30%). Line plots report the mean accuracy across 5 different random seeds. **e** Performance of the same set of models on the fault-type classification task across the corresponding test conditions. **f** Performance on the protection failure detection task, evaluated across all test datasets. In all diagnostic tasks, the curves highlight differences in model robustness as data quality degrades.

Q4. While the paper provides a systematic strategy for training data generation through topology selection, fault enumeration, and data augmentation, the description of the simulated fault scenarios remains somewhat vague. It is not explicitly stated which fault types (e.g., single-line-to-ground, line-to-line, three-phase faults) or fault resistances were considered. Since the realism and diversity of training data critically affect generalization in practice, a clearer specification of fault categories, resistance ranges, and operating conditions would significantly strengthen the methodology.

A4. We sincerely thank the reviewer for this valuable comment. We fully agree that a clearer and more explicit specification of the simulated fault scenarios—particularly fault categories, resistance ranges, and operating conditions—is essential for demonstrating the realism and diversity of the digital-twin-generated dataset. In response, we have substantially expanded the description of the fault-simulation settings in the revised manuscript.

As detailed in the revised ***Results - Case setup*** section and summarized in ***Supplementary Table 3***, the digital twin systematically covers all physically meaningful primary-system fault categories and operating conditions of the studied substation:

- Operating topologies.

  Faults were simulated under 10 distinct operating topologies for training and 4 previously unseen topologies for testing, each constructed from real breaker/isolator configurations.

- Fault locations and types.

A total of 16 fault locations (covering lines, busbars, transformer HV/LV terminals, and sectionalizers) were modeled.

For each location, 10 standard fault types were simulated, including:

- A/B/C single-line-to-ground faults

- AB/BC/CA line-to-line faults

- ABG/BCG/CAG line-to-line-to-ground faults

- three-phase short-circuit faults

- Fault resistance.

  To reflect realistic low-resistance faults, the resistance was randomized within 0-10 Ω for every simulation, enabling diverse fault currents and protection-operation patterns.

- Protection-failure variations.

  Secondary-system uncertainties were included through an automated Breadth-First-Search-based fault-enumeration tree, generating 11,597 training scenarios and 3,890 test scenarios with diverse protection-failure combinations.

- Data modalities.

  Each simulated scenario provides synchronized alarm sequences, SCADA measurements (2 Hz), and waveform recordings (4 kHz), consistent with IEC 61850 practice in real substations.

Furthermore, to strengthen realism and promote generalization, we applied nine augmentation strategies, including Gaussian noise (30-50 dB SNR), random sampling loss (10–30%), and complete channel loss (10–30%) for SCADA and waveform data—reflecting real-world sensor degradation, communication issues, and device failures.

These explicit specifications ensure that the training dataset spans the full range of fault modes, resistances, operating topologies, and secondary-system behaviors that occur in practice. We have incorporated these clarifications into the revised manuscript for greater transparency and methodological rigor.

The corresponding revisions in the Manuscript:

Results

Case setup (Line 129–163)

To capture diverse operational conditions, ten different topologies are simulated by varying circuit breaker open/close statuses (Fig. 1b), generating the training dataset through batch fault simulations. Each

topology covers all 16 fault locations and 10 fault types (A/B/C single-phase grounding, AB/BC/CA two-phase short-circuit, AB/BC/CA two-phase-to-ground short-circuit, and three-phase short-circuit). Fault resistance is randomly set within 0–10 Ω to represent low-resistance faults. Based on these combinations, 1,600 base fault scenarios are defined. To further consider secondary system uncertainties, multiple protection failure conditions are generated automatically using a fault enumeration tree traversal method based on Breadth-First Search (Supplementary Methods), resulting in 11,597 distinct fault scenarios for the training dataset.

For each fault case, alarm data are collected from PRs, including time-tagged event signals recorded during the fault process. SCADA measurements record the root-mean-square (RMS) values of three-phase and zero-sequence currents of each line via current transformers (CTs), sampled at 2 Hz, retaining 1 pre-fault and 19 post-fault data points within a 10 s time window. Fault waveform recordings capture the instantaneous values of the same current quantities at a sampling rate of 4 kHz, which is consistent with the requirements for high-speed fault recording in smart substations. Each waveform record contains 5 pre-fault cycles (0.1 s) and 20 post-fault cycles (0.4 s), yielding 2,000 data points per channel. It is worth noting that the proposed method does not impose strict requirements on the sampling frequency of waveform recordings, as the proposed model extracts RMS features over multi-cycle windows. As long as the sampling rate is sufficient to capture the transient characteristics during fault events, the diagnostic performance of the model remains unaffected.

To enhance robustness and generalization, multiple data augmentation strategies are applied, including adding zero-mean Gaussian noise with signal-to-noise ratios (SNR) of 50, 40, and 30 dB; introducing random sampling loss by removing 10%, 20%, and 30% of time-series data points from alarm, SCADA, and waveform data; and simulating sensor failures by randomly dropping 10%, 20%, and 30% of entire SCADA or waveform channels. These augmentations generate nine additional datasets, which, together with the original data, result in a total of 115,970 training samples.

To evaluate generalization capability, four unseen topologies are selected for testing (Fig. 1c). For each topology, fault scenarios are generated using the same procedure as for the training set—including all fault locations, fault types, fault resistances, and the same protection-failure enumeration method—resulting in 3,890 distinct base fault scenarios. Using identical noise, sampling-loss, and sensor-failure augmentation strategies, 10 corresponding test datasets are constructed, forming the final test set (Supplementary Table 3).

This dataset, incorporating diverse topologies, fault locations, fault types, fault resistances, and protection failures, together with multi-level augmentation strategies, comprehensively represents the complexities and uncertainties of real-world substation operation.

……

Supplementary Table 3: Summary of the dataset

Item	Description
Topologies	10 for training and 4 for testing (different operating configurations)
Fault locations	16 (transmission lines, busbars, transformers, sectionalizers)
Fault types	10 (A/B/C single-phase grounding; AB/BC/CA two-phase short-circuit; AB/BC/CA two-phase-to-ground short-circuit; three-phase short-circuit)
Fault resistance	Randomly sampled within 0-10 Ω to represent low-resistance faults

Protection failure conditions	Automatically enumerated using a Breadth-First Search-based fault enumeration method (Supplementary Method)
Base fault scenarios	11,597 for training; 3,890 for testing
Data modalities	Alarm events, SCADA measurements (2 Hz, RMS values of three-phase and zero-sequence currents from CTs), and fault waveform recordings (4 kHz, instantaneous values of the same current quantities)
Noise levels (SNR)	30, 40, and 50 dB (zero-mean Gaussian noise)
Sampling loss	Random removal of 10%, 20%, or 30% of time-series data points from alarm, SCADA, waveform data
Sensor failures	Random dropping of 10%, 20%, or 30% of the entire SCADA or waveform channels
Total fault samples	115,970 for training; 38,900 for testing

Q5. The combined use of GAT, DAE, Self-Attention, and SCL likely imposes a significant computational burden. The paper would benefit from a more detailed analysis of complexity, training time, hardware requirements, and feasibility for real-time deployment.

A5. We sincerely thank the reviewer for this important comment. We agree that a more detailed analysis of computational complexity, training cost, hardware requirements, and real-time deployment feasibility would significantly strengthen the manuscript. In the revised version, we have added comprehensive quantitative evaluations and clarified the latency behavior of the deployed digital-twin system.

To enable transparent assessment of computational overhead, we provide detailed architectural parameters for all modules—including the GAT encoders, DAE decoder, self-attention layers, and task-specific classifiers—in ***Supplementary Table 5***. These include the number of layers, hidden dimensions, attention heads, and learnable parameters for each component. The full model contains approximately 50 million parameters, which is comparable to other advanced deep-learning approaches for multimodal time-series and graph-structured data.

To further quantify training and inference cost, ***Supplementary Table 7*** reports the training time per epoch, GPU memory usage, total training duration, and inference latency across all baseline models and all stages of the proposed framework (DAE pretraining, SCL training, classifier training). All experiments were conducted on an Intel Xeon E3-1230 v5 CPU and an NVIDIA RTX 3090 GPU, which represent a realistic hardware configuration for substation-level deployments. The results show that while the proposed model requires a longer training time than some lightweight baselines, the overall cost remains reasonable. GPU memory consumption is also moderate across all modules, fitting comfortably within typical workstation GPU constraints. Importantly, inference remains efficient, requiring only ≈0.5 s per sample, which is consistent with other deep-learning methods used for high-resolution time-series and graph data. These findings confirm that although the proposed method integrates multiple advanced components (GAT, DAE, self-attention, SCL), the overall computational burden remains well within practical limits for substation applications.

Finally, in the ***Supplementary Notes - Latency analysis of the digital twin system***, we provide a detailed breakdown of the end-to-end diagnostic delay in actual engineering deployment. The total latency consists of: (i) data acquisition, (ii) data processing, and (iii) model inference. For data acquisition, SCADA and alarm/event data arrive with millisecond-level delay and therefore do not significantly impact overall timing. The dominant contributor is the generation of waveform files inside protection IEDs, which takes approximately 60 s. Data processing requires about 1 s, and model inference only ≈0.5 s. This latency is acceptable in practice, as the proposed method is designed for post-fault diagnosis, where diagnostic accuracy and reliability are more critical than strict real-time response. These results confirm that the proposed diagnostic framework is fully feasible for engineering deployment in digital-twin-enabled substations.

The corresponding revisions in the Supplementary Information:

Supplementary Table 5: Hyperparameters of the proposed model

**Feature Extraction GAT 1/2/3**	
**Item**	**Parameter**
Layers	3
Node num	16
Input node dim	143/100/80
Hidden layer 1 node dim	256
Hidden layer 2 node dim	256
Output node dim	256
Attention Heads	4 heads per layer
Learnable parameters (Millions)	0.68/0.63/0.61
**DAE Decoder GAT 4**	
**Item**	**Parameter**
Layers	3
Node num	16
Input node dim	3×256=768
Hidden layer 1 node dim	512
Hidden layer 2 node dim	512
Output node dim	143+100+80=323
Attention Heads	4 heads per layer
Learnable parameters (Millions)	3.3
**Self-attention Layer 1/2/3**	
**Item**	**Parameter**
Sequence Length (Input Modality)	3
Input Dim	16×256 = 4096
Normalization Method	Layer Norm
Attention Heads	16
Output Dim	1024
Learnable parameters (Millions)	12.59
**Classifier MLP 1/2/3**	

Item	Parameter
Layers	2
Input Dim	3×1024
Hidden Dim	1024
Output Dim	16/10/143
Learnable parameters (Millions)	3.16/3.16/3.22

Supplementary Table 7: Training time, inference time and GPU memory usage of each module across all baseline models

Model	DAE Training Time (s/epoch)	DAE GPU Mem (MB)	SCL Training Time (s/epoch)	SCL GPU Mem (MB)	Classifier Training Time (s/epoch)	Classifier GPU Mem (MB)	Total Training Time (hours)	Inference Time (ms/sample)
MPNN (end-to-end training)	N/A	N/A	N/A	N/A	75.3	2183.9	5.23	622
MLP-based	13.62	178.5	10.01	311.4	27.92	198.0	2.87	155
ResNet-based	91.13	6153.4	37.01	1689.6	69.52	1594.9	9.41	337
LSTM-based	18.79	1943.4	11.62	781.7	28.42	684.8	3.14	157
GCN-based	89.68	438.8	39.84	361.9	88.31	262.7	10.84	563
Transformer-based	50.17	5540.8	21.29	627.8	41.30	531.1	5.44	181
Proposed	93.33	1132.0	41.4	383.7	100.90	286.6	11.90	560

Supplementary Notes

Latency analysis of the digital twin system

The end-to-end diagnostic latency of the digital twin system can be understood as the cumulative duration of three sequential stages: (i) acquiring the required data, (ii) processing and organizing the data into model inputs, and (iii) executing the diagnostic model.

- Stage 1 — Data acquisition.

Before the fault occurs, the digital twin continuously receives real-time switching states of circuit breakers and disconnectors via IEC 61850 GOOSE and MMS services, ensuring that the pre-fault topology is already available at the moment the fault begins. During the fault, bay-level protection IEDs generate microsecond-synchronized Sequence-of-Events (SOE) logs, and SCADA analog measurements continue to be reported through MMS. These messages typically reach the station level within milliseconds and therefore do not contribute significantly to diagnostic latency.

In contrast, the acquisition of fault waveform data is inherently slower. Protection IEDs and standalone fault recorders must capture at least 2 s of pre-fault data and the entire fault transient before a COMTRADE file can be generated, and this file-generation process is time-consuming. In the 110 kV smart substation studied in this work, generating the waveform files from multiple triggered recorders takes approximately 50 seconds, making this step the dominant contributor to the end-to-end latency. Once generated, the waveform

files—typically about 20 MB each—are uploaded to the station level via IEC 61850 MMS file services in around 10 seconds.

- Stage 2 — Data processing.

After all waveform files are available at the station level, the digital twin reconstructs the pre-fault topology, parses the COMTRADE channels, extracts the necessary diagnostic features, and formats the combined SOE, SCADA, and waveform information into the model's input structure. These operations are computationally lightweight and require roughly 1 s.

- Stage 3 — Model inference.

The processed data are then fed into the diagnostic model, which runs on an Intel Xeon E3-1230 v5 CPU and an NVIDIA RTX 3090 GPU. The inference stage is fast, producing the final diagnostic decision within approximately 0.5 s.

Because SOE and SCADA reporting overlap with the waveform acquisition phase, the overall latency is determined primarily by the waveform file-generation delay. Let t1 denote the time for recorder-side file generation and upload, t2 the data-processing time, and t3 the inference time. The end-to-end latency is therefore

$$T_{\text{latency}}=t_1+t_2+t_3 \tag{13}$$

Given that $t_1≈60$ s, $t_2≈1$ s, and $t_3≈0.5$ s, the overall latency is dominated by the waveform file-generation process.

Q6. The evaluation relies mainly on accuracy metrics. Adding precision, recall, F1-score, … would provide a more comprehensive understanding of model performance.

A6. We sincerely thank the reviewer for this insightful suggestion. We fully agree that accuracy alone may not fully reflect the diagnostic performance, especially for multi-class and multi-label tasks such as fault location, fault type classification, and protection failure detection. Including additional metrics such as precision, recall, and F1-score can provide a more comprehensive and balanced evaluation of the proposed model and the baseline methods.

In response, we expanded the evaluation framework to report precision, recall, and F1-score for all diagnostic subtasks across both training and testing sets. These metrics allow us to quantify not only the correctness of predictions but also the model's ability to avoid false alarms and missed detections—two aspects of particular importance in practical substation applications. To ensure fairness, all metrics were computed under five random seeds, and the results are presented as mean ± standard deviation to reflect model stability.

The detailed quantitative results for all baseline models (including TCFPN, MPNN, MLP-based, ResNet-based, LSTM-based, GCN-based, and Transformer-based models) and the proposed framework are now included in ***Supplementary Data***. The expanded comparisons further demonstrate the superiority and robustness of the proposed multimodal fusion approach under noisy and degraded data conditions.

Q7. The scope of the study is focused on diagnosing faults within substations, addressing fault location, type, and protection failures. Can external faults also generate alarms, waveform disturbances, and SCADA events in substations, resulting in mal-operation of the proposed scheme?

A7. We sincerely thank the reviewer for raising this important question. Clarifying the influence of external faults on the proposed diagnostic scheme is indeed essential.

In practical substation operation, due to the selectivity of protection coordination, only the protection devices directly associated with the faulted equipment will operate. Therefore, when a fault occurs outside the substation, the primary protection on the external line or feeder typically clears it, and no internal protection device inside the substation should operate. In this normal case, the proposed diagnostic system is not triggered because no internal relay trips, no station-level waveform recorders are activated, and the alarm pattern does not correspond to an internal fault.

However, if an external fault is not cleared by its primary protection—such as when the main protection refuses to operate—backup protections inside the substation may be triggered. These backup actions will generate alarm sequences, activate waveform recording, and produce SCADA disturbances. Because the diagnostic capability is inherently limited by the modeling boundary of the digital twin, external faults beyond the modeled scope cannot yet be reliably distinguished. In such situations, the diagnostic process will naturally converge to the boundary nodes where the substation connects to the external network. Specifically, for source-side external faults, the diagnostic result will point to the corresponding 110 kV incoming line terminals, which are modeled as boundary nodes in the topology. For load-side external faults, the diagnosis will converge to the corresponding 10 kV busbars, which represent the connection points to distribution feeders outside the station.

If broader coverage of external systems is desired—for example, diagnosing faults anywhere on the connected transmission or distribution network—the digital twin and diagnostic model should be extended to include the upstream or downstream network sections, thereby enabling end-to-end fault diagnosis beyond substation boundaries. Future work will therefore focus on expanding the modeling scope and integrating detailed representations of adjacent network segments, so that the proposed scheme can accurately identify, classify, and localize faults occurring outside the substation, and enhance its robustness under complex system-wide disturbance scenarios.

Response to Reviewer #5

The paper proposes a deep learning framework based on graph neural networks for the design of digital twin for fault diagnosis. Specifically graph attention networks (GAT) are utilized with incorporation of a multi-modal fusion strategy. The multi-modal fusion strategy allows to incorporate various data sources such as alarms, fault waveform recordings and supervisory control and data acquisition measurements.

Overall assessment: The proposed approach is interesting in the context of a new application, but the methodological novelties and their added value compared to existing methods are not completely clear. Experimental setup and the description of the data are too limited to fully see potential benefit of the work.

Q1. It is not completely clear how novel the proposed framework is in the context of graph attention networks and multi-modal fusion. Different pipelines already exist in different fields and a more thorough review/clear novelty in terms of methodological contributions should be described.

A1. We sincerely thank the reviewer for raising this important point. We fully agree that, given the rapid progress of multimodal fusion and graph neural networks, it is essential to clearly position the novelty of our proposed framework relative to existing pipelines. To address this comment, we have substantially expanded the literature review and clarified the methodological innovations of our work in both the ***Introduction*** and ***Method*** sections.

In the revised manuscript, we provide a more comprehensive review of existing multimodal fusion paradigms—including encoder-decoder-based, attention-based, GNN-based, generative, and constraint-based frameworks—and summarize their strengths in industrial diagnosis settings. Building on these paradigms, our study learns from existing designs but further develops a framework specifically tailored to the electrical, topological, and multimodal characteristics of substation fault diagnosis, which differ fundamentally from conventional applications.

Specifically, the methodological novelty of our framework lies in the following aspects:

- Digital-twin-based data generation for training and deployment consistency

  We leverage a high-fidelity digital twin of the substation to generate diverse, labeled multimodal training samples, addressing the scarcity and imbalance of real fault data. Moreover, because the digital twin can interact with the physical system, enabling seamless transfer of the learned model to practical engineering environments.

- Topology-adaptive GAT designed for substation topology

  Instead of applying a generic GAT, we design a topology-adaptive GAT encoder that incorporates electrical topology. This allows message passing to follow protection zones, logical connectivity,

and actual power-flow relationships, enabling the model to learn fault semantics that are physically meaningful and generalize across substations with different configurations.

- Task-specific multimodal fusion via self-attention

  Different modalities contribute differently to different diagnostic tasks (e.g., waveform for fault type, alarm for protection failure detection). We therefore adopt a task-specific self-attention fusion mechanism, in which cross-modal attention weights are learned independently for each task, enabling the model to adaptively allocate modality importance rather than using a one-size-fits-all fusion strategy. This leads to more accurate, interpretable, and physically grounded multimodal integration.

- Denoising autoencoder (DAE) for robust shared representation learning

  Substation measurements inevitably contain noise, missing values, and asynchronous sampling. To handle these challenges, we introduce a denoising autoencoder that learns noise-invariant latent features before multimodal fusion, improving robustness against SCADA noise, waveform distortion, and partial data loss.

The corresponding revisions in the Manuscript:

Introduction (Line 75-84)

……

Multi-modal fusion aims to integrate heterogeneous data, exploit their complementarity and correlations, and thereby enhance the performance and generalization of downstream tasks. A recent work[35] categorized fusion models into encoder-decoder-based[36], attention-based[37], GNN-based[38], generative neural network-based[39], and constraint-based[40] frameworks. These models have shown strong potential in industrial fault diagnosis—for example, deep coupling autoencoders for multi-sensor data fusion in rotating machinery[41], time-frequency transformers for bearing fault diagnosis[42], and graph-based fusion models for root cause localization in large-scale microservice systems[43]. Motivated by these advances, this study aims to design a substation fault diagnosis framework that leverages diverse data modalities and combines the strengths of attention and graph-based fusion for accurate, robust, and topology-generalizable performance.

……

Methods

……

Digital twin-driven fault diagnosis framework (Line 451-453)

The proposed digital twin-driven fault diagnosis framework aims to achieve accurate and adaptive fault diagnosis in power substations through the integration of virtual simulation and multi-modal fusion learning, thereby facilitating practical engineering applications.

……

Feature extraction networks based on GATs (Line 495-500)

GAT is a type of graph neural network that integrates the attention mechanism into the message-passing process[47]. GATs are inherently suitable for processing graph-structured data, enabling efficient modeling of complex topological relationships and inter-node dependencies. Their adaptability to dynamic topologies allows the model to effectively utilize both the substation's primary topology information and diverse fault data, thereby extracting high-quality feature representations. This enhances the topological generalization capability of the model and improves fault diagnosis accuracy.

……

Feature fusion network based on self-attention mechanism (Line 536-539)

In the feature fusion network, self-attention mechanism is employed to model global correlations among multi-modal fault features and adaptively adjust their significance according to the diagnostic task. By leveraging this mechanism, the proposed approach ensures flexible and effective feature fusion, enhancing the accuracy of each fault classification task.

……

Denoising autoencoder pretraining (Line 621-624)

To enhance feature extraction from heterogeneous fault data and mitigate the adverse effects of noise and data loss on diagnostic performance, a DAE is introduced to pretrain the feature extraction network. As an unsupervised model capable of learning latent representations through reconstruction[50], the DAE effectively derives robust features even when inputs are noisy or partially missing.

……

Q2. It is not clear what are the added benefits of attention features extractors. (Figure 6). Why can these not be simply replaced by MLPs? This would make the model simpler. Experiments to justify this block of the model are needed.

A2. We sincerely thank the reviewer for this insightful question. We have substantially expanded the explanation and added new comparative experiments to justify the design choice.

In our framework, the GAT module is designed to extract topology-aware structural features from the real-time substation topology and to learn how electrical components interact under different operating conditions. Protection behavior and fault propagation patterns depend strongly on the connectivity and interaction among lines, buses, transformers, and sectionalizers. GAT explicitly operates on the substation topology, using attention to weight messages passed along edges, so that nodes corresponding to critical equipment can be highlighted adaptively. In contrast, a standard MLP assumes independent, unstructured inputs and cannot encode graph connectivity or directional relationships. As a result, an MLP-based feature extractor is fundamentally limited in representing topology-dependent fault signatures, especially when the operating topology changes.

To directly evaluate the reviewer's concern, we conducted a comprehensive set of experiments in which the GAT module was replaced by five commonly used neural architectures:

- MLP-based model, which ignores structural information and treats all node features as a flat vector;

- ResNet-based model, which has strong local feature extraction capability in Euclidean grids but no explicit graph modeling;
- LSTM-based model, which emphasizes temporal dependencies but is weak in representing spatial/topological structure;
- GCN-based model, which performs graph convolution with uniform neighborhood aggregation but without adaptive attention;
- Transformer-based model, which applies global self-attention on token sequences but does not explicitly encode the substation topology.

For fairness, all these data-driven baselines were implemented by replacing only the GAT module in the proposed framework, while keeping all other components, data inputs, and optimization settings unchanged. Their hyperparameters and parameter counts were further adjusted so that the total number of learnable parameters is comparable to that of the proposed model (see ***Supplementary Table 5,6***). This ensures that performance differences are attributable to the modeling of topology and multimodal interactions rather than to model size.

The revised ***Results - Comparison of fault diagnosis models*** section now reports a detailed comparison against these baselines. All models are evaluated on ten test datasets emulating realistic data degradations (noise, sampling loss, and sensor failures) across the three diagnostic tasks. Accuracy is reported in the main text, and precision, recall, and F1-scores (mean ± standard deviation over five random seeds) are provided in the ***Supplementary Data***. The results show that the proposed GAT-based model consistently outperforms all baselines under varying disturbance conditions. In particular, models without explicit topology awareness (MLP, LSTM, Transformer) and those with limited structural adaptivity (ResNet, GCN) exhibit reduced robustness when topologies change or when data are partially missing, confirming that topology-aware attention is indeed beneficial rather than an unnecessarily complex choice.

The corresponding revisions in the Manuscript:

Results (Line 184–235)

Comparison of fault diagnosis models

……

Seven baseline models are implemented for performance comparison. Temporal constrained fuzzy Petri net (TCFPN)[12] is a rule-based approach that relies solely on alarm information to identify fault locations and protection failures. It builds upon an improved Petri Net–based framework and represents a typical rule-based solution for substation fault diagnosis. However, the model requires manual updates whenever the system topology changes. The MPNN[21] model is a data-driven method that utilizes system topology and fault waveform data to predict fault locations and types. Although originally designed for microgrid fault diagnosis, its modeling framework can be readily extended to substations and represents a state-of-the-art graph-based data-driven approach. The MLP-based, ResNet-based, LSTM-based, GCN-based, and Transformer-based models are constructed by replacing the GAT module in the proposed framework with the corresponding architectures, representing common deep learning baselines. For fair comparison, all data-driven models are trained under identical optimization settings and data inputs, and their hyperparameters and parameter counts are adjusted to maintain comparable model complexity (Supplementary Table 5,6). While the proposed model does increase the training time compared to some baselines, the overall training and inference costs remain

reasonable, without introducing significant overhead relative to other deep learning models (Supplementary Table 7).

All models are evaluated on ten test datasets corresponding to different data degradation conditions, which emulate practical disturbances in communication and measurement commonly encountered in substations. For quantitative evaluation, three diagnostic subtasks are assessed independently. Accuracy is used as the primary performance metric in the main text, while additional metrics, including precision, recall, and F1-score on both training and test datasets, are provided in the supplementary material (Supplementary Data). Each experiment is repeated under five random seeds, and results are reported as mean ± standard deviation to evaluate model stability.

The proposed model consistently outperforms all baseline approaches under varying levels of noise, sampling loss, and sensor failure (Tables 1–3, Fig. 1d–f, Supplementary Fig. 3–5, Supplementary Data).

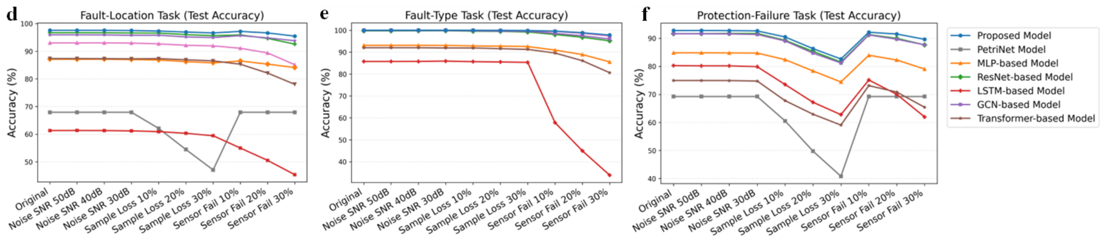

TCFPN relies solely on alarm information; however, multiple fault scenarios can produce highly similar alarm patterns, and the rule-based inference cannot resolve these ambiguities. As a result, its outputs remain largely invariant across some different test conditions, reflecting the limited discriminative capacity of alarm-only reasoning.

MPNN utilizes system topology and fault waveform recordings, enabling competent performance in fault-location and fault-type identification. However, because it does not incorporate alarm information, it cannot diagnose protection failures. In addition, MPNN is trained in a single end-to-end manner rather than following the staged training strategy adopted in the proposed framework, resulting in less effective feature extraction and, consequently, lower diagnostic accuracy. This training scheme also limits its robustness when the input data are noisy, incomplete, or corrupted.

LSTM-based models emphasize temporal correlations and are not well-suited for capturing spatial or topological interactions, leading to reduced robustness when the input topology changes. The MLP-based and Transformer-based variants, though capable of strong representation learning when sufficient data are available, treat inputs as flattened or tokenized sequences and therefore cannot explicitly leverage the substation's graph structure. This limits their ability to learn inter-node dependencies, resulting in weaker generalization and reduced robustness under topology variations.

ResNet-based and GCN-based models partially address spatial feature learning—ResNet through 2D convolutions on processed inputs and GCN through graph convolutions. Both achieve relatively strong performance; however, their ability to capture the underlying topological relationships is limited. ResNet operates on Euclidean grids and cannot represent non-uniform electrical connectivity, while GCN performs homogeneous graph convolutions that cannot adaptively weight critical nodes. These limitations make them less effective than GAT in modeling the directional and heterogeneous dependencies among substation components. In contrast, the proposed GAT-based fusion mechanism performs adaptive, node-wise attention and integrates topology, waveform, SCADA, and alarm information more effectively, yielding the highest

overall accuracy.

……

Q3. It is not clear whether there is no edge information in the graphs and if not why not. At least this seems to be the case in the way the models are described.

A3. We sincerely thank the reviewer for raising this important question and apologize for the lack of clarity in our previous description. We clarify that edge information is indeed incorporated in our graph modeling, and it plays a central role in the proposed method.

First, the substation topology is explicitly represented as a graph whose edges are defined by the electrical connectivity between components. The adjacency matrix is constructed from the switching states of circuit breakers, disconnectors, and grounding switches. A non-zero entry indicates a physical electrical connection between two components. This adjacency matrix directly determines the graph structure used by the GAT and is dynamically updated when the operating configuration changes (e.g., feeder switching or busbar reconfiguration). These connectivity-based edges encode fault propagation paths and protection-zone relationships, which are essential for topology-aware fault diagnosis.

Second, regarding the form of edge information, we adopt a standard GAT architecture in which edges mainly represent connectivity relationships rather than having explicitly defined edge attributes. Instead of using handcrafted edge features, the GAT learns attention coefficients between connected node pairs during message passing. These coefficients adaptively weight the information aggregated from neighboring nodes and can be interpreted as implicit, data-driven edge features, reflecting the varying importance of interactions between connected components under different operating conditions.

Finally, the importance of explicitly modeling edges is confirmed by our empirical results. The test datasets contain operating topologies different from those seen during training, which is essential for assessing the model's capacity to generalize across different substation topologies. Baseline models that do not incorporate explicit topology (e.g., MLP, LSTM, Transformer) show a clear decrease in diagnostic performance under these unseen topological conditions. This performance degradation indicates that edge information plays a critical role in capturing the spatial interactions and protection-related dependencies inherent to substation fault mechanisms, and that omitting it would significantly weaken the model's generalization and robustness. Furthermore, we also evaluated graph-based baselines that explicitly incorporate the same topological edges, including GCN and MPNN. Although these models benefit from topology awareness and outperform non-graph baselines, their performance remains consistently inferior to that of the proposed GAT. This result suggests that, beyond merely introducing edge connectivity, the adaptive attention mechanism of GAT enables more effective utilization of edge information, which is particularly important under varying and unseen operating topologies.

Q4. The correlation feature analysis should be provided to understand the structure of the data better. Do we really need to have separate blocks for attention based features extraction, even if the features are highly correlated?

A4. We sincerely thank the reviewer for this insightful question. The reviewer correctly points out the importance of understanding the correlation structure among different data sources and raises a valuable question regarding the necessity of using separate attention-based encoders when the features may be correlated. This comment allows us to clarify both the relationships among the three modalities and the rationale behind our multi-branch encoder design.

Although alarm data, SCADA measurements, and fault waveforms originate from the same underlying physical fault and thus exhibit certain correlations, the three modalities used in this study differ fundamentally in their temporal resolution and semantic structure:

- Alarm data in our dataset are discrete, event-driven signals collected from protection relays (PRs), recorded as time-tagged events during the fault process.
- SCADA measurements used in this study are low-frequency quasi-steady signals, sampled at 2 Hz, providing 1 pre-fault and 19 post-fault data points over a 10-second window for each fault case.
- Fault waveform recordings in this study are high-frequency transient signals, sampled at 4 kHz, capturing 5 pre-fault and 20 post-fault cycles (0.1 s + 0.4 s), resulting in 2000 data points per channel.

These modality characteristics underline the sharp heterogeneity in sampling rates, sequence lengths, information densities, and semantic granularity. Therefore, even if physical correlations exist, the data characteristics used in this work are intrinsically incompatible at the raw level. The proposed framework employs independent encoders to map these heterogeneous modalities into a unified latent space. Each encoder is tailored to the statistical nature of its corresponding input, ensuring that modality-specific patterns are effectively captured and that the representations are properly aligned before fusion.

Furthermore, independent encoders are essential for effective self-attention-based fusion, not only because attention relies on structured modality-consistent embeddings, but also because different diagnostic subtasks depend on different modalities to varying degrees. For example, waveform data contribute more to fault type classification and alarm signals are often critical for diagnosing protection failures. By extracting modality-specific representations first, the attention mechanism is able to adaptively assign appropriate weights to each modality according to the requirements of each diagnostic task. This flexibility would not be achievable if the modalities were mixed prematurely in the raw or weakly processed feature space. Only after reliable and aligned modality-specific embeddings are obtained can cross-modal dependencies be learned in a meaningful and task-aware manner.

Finally, the multi-branch design improves the extensibility of the framework. If additional information sources (e.g., video data) become available in future applications, they can be incorporated naturally by adding a new encoder branch without redesigning the entire architecture.

In summary, although the three data sources are physically correlated, their highly heterogeneous sampling configurations and distinct roles across diagnostic subtasks make independent attention-based

encoders necessary. This design ensures robust representation learning, balanced modality contributions, task-adaptive fusion behavior, and effective cross-modal attention. We sincerely appreciate the reviewer’s question, which allowed us to clarify this key methodological rationale.

Q5. The baseline methods in the experiments are not well chosen, the proposed method already contains TCFPN in it, so it is not very surprising that the proposed method outperforms it. More conventional baselines are needed for proper assessment. This comparison should also include computational training and inference time.

A5. We sincerely thank the reviewer for this insightful and constructive comment. The reviewer correctly pointed out the need for a more comprehensive and representative set of baseline models. In response, we significantly expanded and refined our baseline design to ensure methodological diversity and fair performance comparison.

In the revised manuscript, we carefully selected baseline models that collectively represent the major paradigms used in substation fault diagnosis and related power system applications:

(1) Rule-based paradigm — TCFPN.

Temporal constrained fuzzy Petri net (TCFPN) is a classical rule-based approach that relies solely on alarm information. It reflects the long-standing industry practice of using logic- and rule-driven inference for substation fault diagnosis. Including TCFPN allows comparison with traditional operational methodologies, especially those still widely deployed in real substations. Its limitations—such as needing manual updates when system topology changes—also highlight the benefit of data-driven solutions.

(2) Graph-based data-driven paradigm — MPNN.

MPNN is a state-of-the-art graph-learning model that uses system topology and fault waveform data. Although originally proposed for microgrids, its framework can be directly extended to substation structures. Including MPNN enables comparison against the leading graph-based learning approaches and provides a strong, topology-aware data-driven baseline that does not rely on alarm information.

(3) Widely adopted deep learning paradigms — MLP / ResNet / LSTM / GCN / Transformer.

To ensure comprehensive and fair evaluation, we further introduced five representative deep learning architectures by replacing the GAT module with the corresponding backbone while keeping the rest of the framework unchanged:

- MLP-based: a classical non-structured deep network assessing performance without inductive biases.
- ResNet-based: a strong convolutional baseline capturing local spatial patterns.
- LSTM-based: a temporal modeling architecture emphasizing sequential dependencies.
- GCN-based: a graph convolution approach representing homogeneous message passing.
- Transformer-based: a powerful attention-based architecture capable of long-range modeling.

These models collectively cover the most common architectural families used in modern data-driven diagnosis systems. Their inclusion ensures that performance gains of the proposed model cannot be attributed to model size, optimization settings, or training procedures but rather to the intrinsic advantages of the GAT-based fusion mechanism.

All data-driven baselines were trained under identical data inputs and optimization settings, with parameter counts adjusted to achieve comparable model complexity (Supplementary Tables 5-6). This controlled design isolates the contribution of each model's feature-extraction capability and ensures that comparisons remain fair and scientifically rigorous.

As requested by the reviewer, we additionally evaluated training time, GPU memory usage, and inference latency for these models (Supplementary Table 7). Experiments were conducted on the same hardware platform. The proposed model introduces a moderate increase in training time due to its adaptive attention computations but maintains practical inference latency (≈0.5 s), satisfying fast operational requirements.

In summary, the expanded baseline suite now spans traditional rule-based, topology-aware graph learning, and representative deep neural architectures, providing a balanced and rigorous comparative foundation. We sincerely appreciate the reviewer's suggestion, which significantly improved the completeness, transparency, and credibility of our experimental evaluation.

The corresponding revisions in the Manuscript:

Results (Line 184–235)

Comparison of fault diagnosis models

……

Seven baseline models are implemented for performance comparison. Temporal constrained fuzzy Petri net (TCFPN)[12] is a rule-based approach that relies solely on alarm information to identify fault locations and protection failures. It builds upon an improved Petri Net–based framework and represents a typical rule-based solution for substation fault diagnosis. However, the model requires manual updates whenever the system topology changes. The MPNN[21] model is a data-driven method that utilizes system topology and fault waveform data to predict fault locations and types. Although originally designed for microgrid fault diagnosis, its modeling framework can be readily extended to substations and represents a state-of-the-art graph-based data-driven approach. The MLP-based, ResNet-based, LSTM-based, GCN-based, and Transformer-based models are constructed by replacing the GAT module in the proposed framework with the corresponding architectures, representing common deep learning baselines. For fair comparison, all data-driven models are trained under identical optimization settings and data inputs, and their hyperparameters and parameter counts are adjusted to maintain comparable model complexity (Supplementary Table 5,6). While the proposed model does increase the training time compared to some baselines, the overall training and inference costs remain reasonable, without introducing significant overhead relative to other deep learning models (Supplementary Table 7).

All models are evaluated on ten test datasets corresponding to different data degradation conditions, which emulate practical disturbances in communication and measurement commonly encountered in

substations. For quantitative evaluation, three diagnostic subtasks are assessed independently. Accuracy is used as the primary performance metric in the main text, while additional metrics, including precision, recall, and F1-score on both training and test datasets, are provided in the supplementary material (Supplementary Data). Each experiment is repeated under five random seeds, and results are reported as mean ± standard deviation to evaluate model stability.

The proposed model consistently outperforms all baseline approaches under varying levels of noise, sampling loss, and sensor failure (Tables 1–3, Fig. 1d–f, Supplementary Fig. 3–5, Supplementary Data).

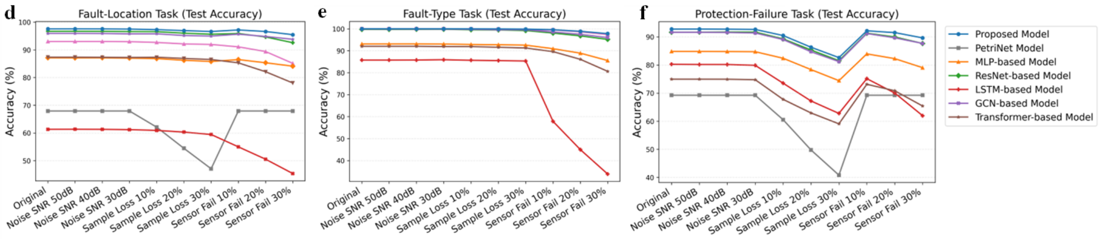

TCFPN relies solely on alarm information; however, multiple fault scenarios can produce highly similar alarm patterns, and the rule-based inference cannot resolve these ambiguities. As a result, its outputs remain largely invariant across some different test conditions, reflecting the limited discriminative capacity of alarm-only reasoning.

MPNN utilizes system topology and fault waveform recordings, enabling competent performance in fault-location and fault-type identification. However, because it does not incorporate alarm information, it cannot diagnose protection failures. In addition, MPNN is trained in a single end-to-end manner rather than following the staged training strategy adopted in the proposed framework, resulting in less effective feature extraction and, consequently, lower diagnostic accuracy. This training scheme also limits its robustness when the input data are noisy, incomplete, or corrupted.

LSTM-based models emphasize temporal correlations and are not well-suited for capturing spatial or topological interactions, leading to reduced robustness when the input topology changes. The MLP-based and Transformer-based variants, though capable of strong representation learning when sufficient data are available, treat inputs as flattened or tokenized sequences and therefore cannot explicitly leverage the substation’s graph structure. This limits their ability to learn inter-node dependencies, resulting in weaker generalization and reduced robustness under topology variations.

ResNet-based and GCN-based models partially address spatial feature learning—ResNet through 2D convolutions on processed inputs and GCN through graph convolutions. Both achieve relatively strong performance; however, their ability to capture the underlying topological relationships is limited. ResNet operates on Euclidean grids and cannot represent non-uniform electrical connectivity, while GCN performs homogeneous graph convolutions that cannot adaptively weight critical nodes. These limitations make them less effective than GAT in modeling the directional and heterogeneous dependencies among substation components. In contrast, the proposed GAT-based fusion mechanism performs adaptive, node-wise attention and integrates topology, waveform, SCADA, and alarm information more effectively, yielding the highest overall accuracy.

……

Supplementary Table 7: Training time, inference time and GPU memory usage of each module across all baseline models

**Model**	**DAE Training Time (s/epoch)**	**DAE GPU Mem (MB)**	**SCL Training Time (s/epoch)**	**SCL GPU Mem (MB)**	**Classifier Training Time (s/epoch)**	**Classifier GPU Mem (MB)**	**Total Training Time (hours)**	**Inference Time (ms/sample)**
MPNN (end-to-end training)	N/A	N/A	N/A	N/A	75.3	2183.9	5.23	622
MLP-based	13.62	178.5	10.01	311.4	27.92	198.0	2.87	155
ResNet-based	91.13	6153.4	37.01	1689.6	69.52	1594.9	9.41	337
LSTM-based	18.79	1943.4	11.62	781.7	28.42	684.8	3.14	157
GCN-based	89.68	438.8	39.84	361.9	88.31	262.7	10.84	563
Transformer-based	50.17	5540.8	21.29	627.8	41.30	531.1	5.44	181
Proposed	93.33	1132.0	41.4	383.7	100.90	286.6	11.90	560

Q6. The data should be described in more detail with a table with summary statistics. In particular, how balanced is the data set, how diverse are the topologies in terms of structure and size. What are summary statistics of the features.

A6. We sincerely thank the reviewer for this valuable comment. We fully agree that providing a clearer and more detailed description of the dataset is essential for demonstrating the realism, completeness, and representativeness of the digital-twin-generated data. In response, we have substantially expanded the dataset description and simulation settings in the revised manuscript.

As detailed in the revised ***Results - Case setup*** section and summarized in ***Supplementary Table 3***, the digital twin systematically covers all physically meaningful primary-system fault categories and operating conditions of the studied substation:

- Operating topologies.

  Faults were simulated under 10 distinct operating topologies for training and 4 previously unseen topologies for testing, each constructed from real breaker/isolator configurations.

- Fault locations and types.

  A total of 16 fault locations (covering lines, busbars, transformer HV/LV terminals, and sectionalizers) were modeled.

  For each location, 10 standard fault types were simulated, including:

- A/B/C single-line-to-ground faults

- AB/BC/CA line-to-line faults

- ABG/BCG/CAG line-to-line-to-ground faults

- three-phase short-circuit faults

- Fault resistance.

  To reflect realistic low-resistance faults, the resistance was randomized within 0-10 Ω for every simulation, enabling diverse fault currents and protection-operation patterns.

- Protection-failure variations.

  Secondary-system uncertainties were included through an automated Breadth-First-Search-based fault-enumeration tree, generating 11,597 training scenarios and 3,890 test scenarios with diverse protection-failure combinations.

- Data modalities.

  Each simulated scenario provides synchronized alarm sequences, SCADA measurements (2 Hz), and waveform recordings (4 kHz), consistent with IEC 61850 practice in real substations.

Furthermore, to strengthen realism and promote generalization, we applied nine augmentation strategies, including Gaussian noise (30-50 dB SNR), random sampling loss (10-30%), and complete channel loss (10-30%) for SCADA and waveform data—reflecting real-world sensor degradation, communication issues, and device failures.

These explicit specifications ensure that the training dataset spans the full range of fault modes, resistances, operating topologies, and secondary-system behaviors that occur in practice. We have incorporated these clarifications into the revised manuscript for greater transparency and methodological rigor.

The corresponding revisions in the Manuscript:

Results

Case setup (Line 129-163)

To capture diverse operational conditions, ten different topologies are simulated by varying circuit breaker open/close statuses (Fig. 1b), generating the training dataset through batch fault simulations. Each topology covers all 16 fault locations and 10 fault types (A/B/C single-phase grounding, AB/BC/CA two-phase short-circuit, AB/BC/CA two-phase-to-ground short-circuit, and three-phase short-circuit). Fault resistance is randomly set within 0–10 Ω to represent low-resistance faults. Based on these combinations, 1,600 base fault scenarios are defined. To further consider secondary system uncertainties, multiple protection failure conditions are generated automatically using a fault enumeration tree traversal method based on

Breadth-First Search (Supplementary Methods), resulting in 11,597 distinct fault scenarios for the training dataset.

For each fault case, alarm data are collected from PRs, including time-tagged event signals recorded during the fault process. SCADA measurements record the root-mean-square (RMS) values of three-phase and zero-sequence currents of each line via current transformers (CTs), sampled at 2 Hz, retaining 1 pre-fault and 19 post-fault data points within a 10 s time window. Fault waveform recordings capture the instantaneous values of the same current quantities at a sampling rate of 4 kHz, which is consistent with the requirements for high-speed fault recording in smart substations. Each waveform record contains 5 pre-fault cycles (0.1 s) and 20 post-fault cycles (0.4 s), yielding 2,000 data points per channel. It is worth noting that the proposed method does not impose strict requirements on the sampling frequency of waveform recordings, as the proposed model extracts RMS features over multi-cycle windows. As long as the sampling rate is sufficient to capture the transient characteristics during fault events, the diagnostic performance of the model remains unaffected.

To enhance robustness and generalization, multiple data augmentation strategies are applied, including adding zero-mean Gaussian noise with signal-to-noise ratios (SNR) of 50, 40, and 30 dB; introducing random sampling loss by removing 10%, 20%, and 30% of time-series data points from alarm, SCADA, and waveform data; and simulating sensor failures by randomly dropping 10%, 20%, and 30% of entire SCADA or waveform channels. These augmentations generate nine additional datasets, which, together with the original data, result in a total of 115,970 training samples.

To evaluate generalization capability, four unseen topologies are selected for testing (Fig. 1c). For each topology, fault scenarios are generated using the same procedure as for the training set—including all fault locations, fault types, fault resistances, and the same protection-failure enumeration method—resulting in 3,890 distinct base fault scenarios. Using identical noise, sampling-loss, and sensor-failure augmentation strategies, 10 corresponding test datasets are constructed, forming the final test set (Supplementary Table 3).

This dataset, incorporating diverse topologies, fault locations, fault types, fault resistances, and protection failures, together with multi-level augmentation strategies, comprehensively represents the complexities and uncertainties of real-world substation operation.

Supplementary Information

Supplementary Table 3: Summary of the dataset

Item	Description
Topologies	10 for training and 4 for testing (different operating configurations)
Fault locations	16 (transmission lines, busbars, transformers, sectionalizers)
Fault types	10 (A/B/C single-phase grounding; AB/BC/CA two-phase short-circuit; AB/BC/CA two-phase-to-ground short-circuit; three-phase short-circuit)
Fault resistance	Randomly sampled within 0-10 Ω to represent low-resistance faults
Protection failure conditions	Automatically enumerated using a Breadth-First Search-based fault enumeration method (Supplementary Method)
Base fault scenarios	11,597 for training; 3,890 for testing
Data modalities	Alarm events, SCADA measurements (2 Hz, RMS values of three-phase and zero-sequence

	currents from CTs), and fault waveform recordings (4 kHz, instantaneous values of the same current quantities)
Noise levels (SNR)	30, 40, and 50 dB (zero-mean Gaussian noise)
Sampling loss	Random removal of 10%, 20%, or 30% of time-series data points from alarm, SCADA, waveform data
Sensor failures	Random dropping of 10%, 20%, or 30% of the entire SCADA or waveform channels
Total fault samples	115,970 for training; 38,900 for testing

Q7. Potential overfitting should be explored and mitigated. At what point would the model start overfitting?

A7. We sincerely thank the reviewer for raising this important question regarding potential overfitting. This allows us to clarify how the training strategy, regularization settings, and dataset design jointly mitigate overfitting, as well as to explain where mild overfitting tendencies may appear during the different stages of model training.

The proposed model is trained following a staged procedure. First, a denoising autoencoder (DAE) is pretrained for 100 epochs using Adam with a learning rate of 1×10^{-3} to obtain noise-robust representations. In the second stage for the fault-location task, the self-attention module is trained with supervised contrastive learning (SCL) for 100 epochs, after which the classifier is trained for 50 epochs with a reduced learning rate of 1×10^{-4}. Throughout all stages, early stopping based on validation loss and L_2 weight decay are consistently applied, and the staged training structure itself avoids overly aggressive end-to-end fitting. These mechanisms collectively restrict model complexity and effectively suppress overfitting.

The dataset construction also plays a decisive role in preventing overfitting. To reflect diverse operating conditions, the training data cover ten topologies generated by breaker switching, and each topology contains all combinations of fault locations, fault types, and randomized fault resistances. Protection-failure enumeration further expands the sample space to 11,597 distinct scenarios. In addition, extensive data augmentation—Gaussian noise at multiple SNR levels, random sampling loss, and simulated channel failures—expands the full training set to 115,970 samples. This broad coverage of topologies, operating conditions, fault scenarios, and data disturbances substantially reduces the likelihood of overfitting.

The loss curves of the three training stages for the fault-location task further illustrate how overfitting behaves in practice. During DAE pretraining, the training and validation losses decrease almost synchronously and remain closely aligned throughout all 100 epochs, indicating that no noticeable overfitting occurs in this stage. During SCL training, although the validation loss exhibits mild fluctuations and a slight upward trend after a certain point, this behavior does not reflect severe overfitting. Instead, it corresponds to the inherent characteristics of contrastive learning. Once the SCL objective succeeds in separating feature clusters in the embedding space—which the curves indicate happens relatively early—the validation loss becomes less sensitive and may fluctuate even when the representations are already well structured. In other words, SCL only needs to reach a point where

samples from different fault classes become well separated, and training beyond that point does not degrade the learned embeddings in a harmful way. This also explains why the classifier stage requires only a small number of epochs to converge: once SCL has produced sufficiently discriminative embeddings, the classifier's role is straightforward, and its validation loss stabilizes quickly. During classifier training, a minor gap between training and validation loss appears after the first few epochs, but the effect remains mild and controlled due to the short training duration and small learning rate.

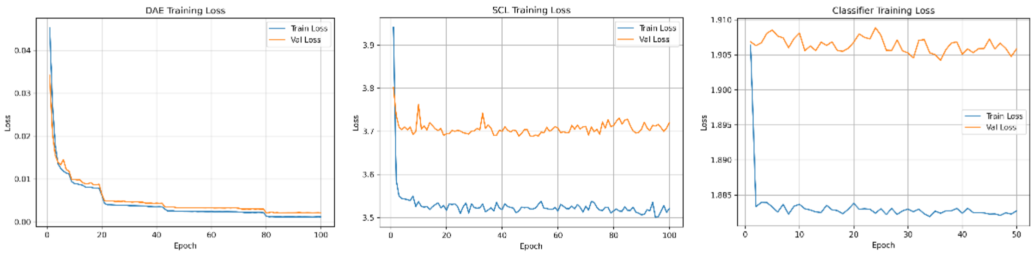

Overall, the DAE stage shows no overfitting, the SCL stage exhibits only mild and expected fluctuations rather than meaningful overfitting, and the classifier stage presents a slight but controlled overfitting tendency that does not affect generalization. Together with early stopping, L_2 weight decay, staged optimization, and a large, diverse dataset, these observations demonstrate that overfitting is effectively managed and does not impact the final performance of the proposed method.

Q8. All figures should be self contained. Currently the captions are very minimal.

A8. We sincerely thank the reviewer for this helpful comment. The reviewer points out that several figures in the manuscript do not provide sufficient standalone explanations, and their captions are too minimal to allow the figures to be properly understood without referring back to the main text. We fully agree that clear and self-contained figure captions are essential for readability and for conveying the contributions of each figure.

In the revised manuscript, we have substantially expanded all figure captions to ensure that each figure can be understood independently.

The corresponding revisions in the Manuscript:

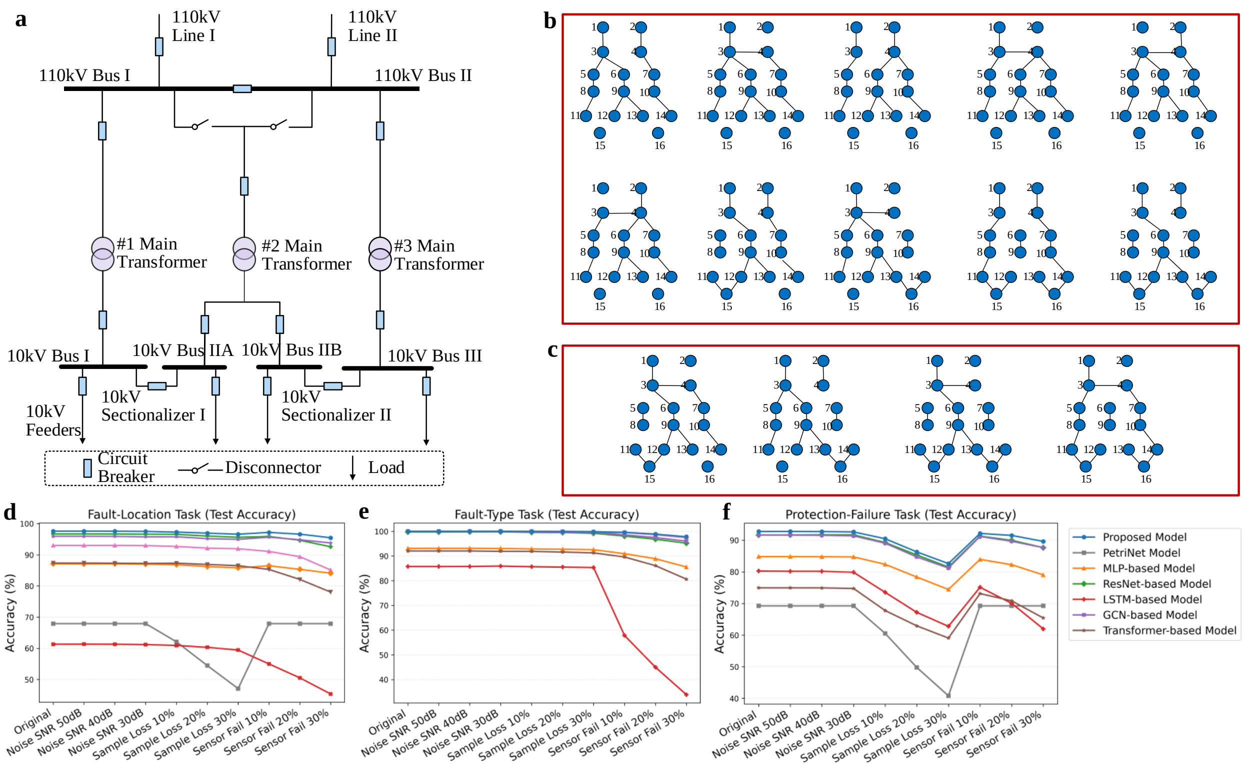

Figure 1. Overview of the substation system, topology configurations, and diagnostic model performance under diverse test conditions. a Overall layout of the studied substation. The system comprises two 110kV incoming lines, two 110kV busbars, three main transformers with both high- and low-voltage terminals, four 10kV busbars, and two 10kV sectionalizer switches, defining 16 physically meaningful fault locations within the primary network. **b** The ten topology configurations used for model training. Each graph contains 16 annotated nodes representing the same set of primary-system fault locations—Nodes 1-2: 110 kV Lines I and II; Nodes 3-4: 110 kV Buses I and II; Nodes 5-7: high-voltage terminals of Main Transformers #1-#3; Nodes 8-10: low-voltage terminals of Main Transformers #1-#3; Nodes 11-14: 10 kV Buses I, IIA, IIB, and III; and Nodes 15-16: 10 kV Sectionalizers I and II. **c** The four topology configurations reserved for testing, designed to introduce previously unseen structural variations and thereby assess the generalization ability of the diagnostic models. **d** Model performance on the fault-location task under a range of test conditions, including the original dataset, noise-corrupted datasets (SNR = 50, 40, and 30 dB), sampling-loss conditions (10%, 20%, and 30%), and sensor-failure conditions (10%, 20%, and 30%). Line plots report the mean accuracy across 5 different random seeds. **e** Performance of the same set of models on the fault-type classification task across the corresponding test conditions. **f** Performance on the protection failure detection task, evaluated across all test datasets. In all diagnostic tasks, the curves highlight differences in model robustness as data quality degrades.

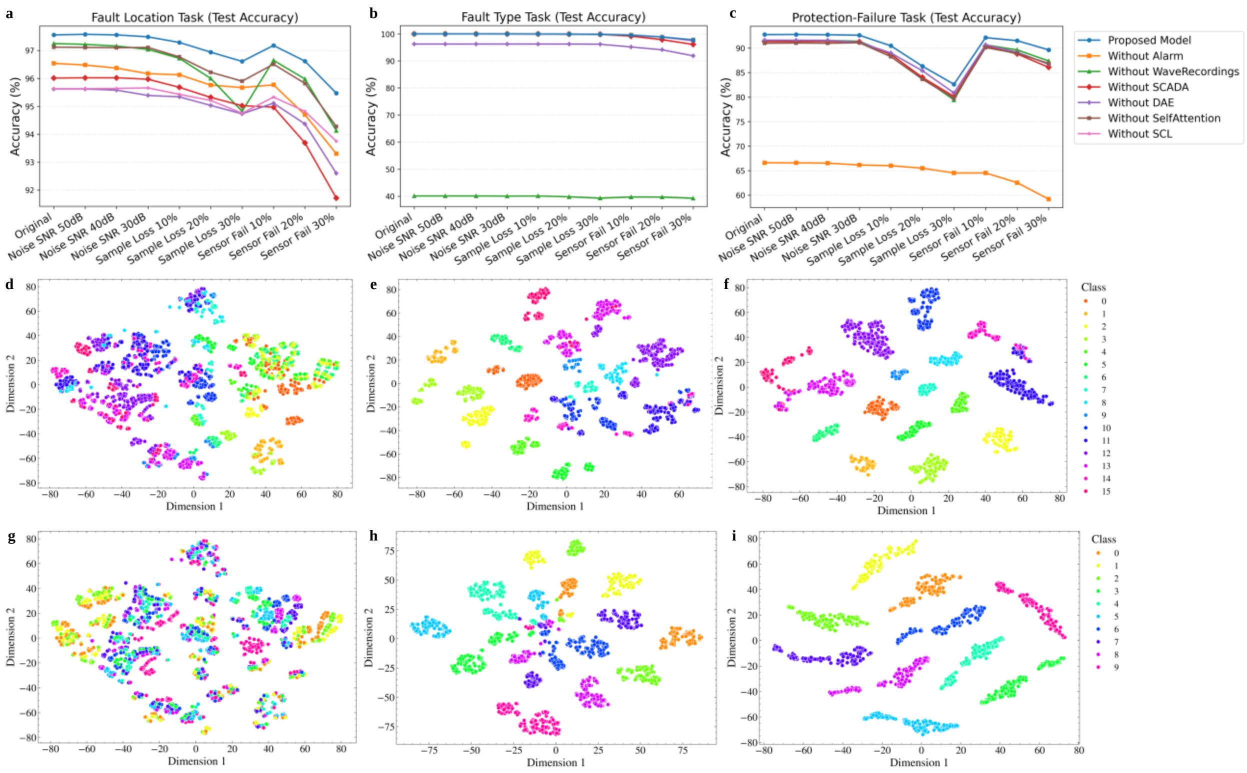

Figure 2. Ablation study results. a Performance of the ablation variants on the fault-location task. The variants include without Alarm, without Waveform, without SCADA, without DAE, without self-attention, without SCL, and the Proposed Model. **b** Performance of the ablation variants on the fault-type classification task under the same evaluation conditions. **c** Performance of the ablation variants on the protection-failure detection task. All models in **a-c** are evaluated on the original test dataset, noise-corrupted datasets (SNR = 50, 40, 30 dB), sampling-loss conditions (10%, 20%, 30%), and sensor-failure conditions (10%, 20%, 30%). Line plots report the mean accuracy across 5 different random seeds. **d** t-SNE visualization of the encoder-output feature embeddings for the fault-location classification task. **e** t-SNE visualization of the feature embeddings after self-attention (SA) fusion for the fault-location classification task. **f** t-SNE visualization of the feature embeddings after applying self-attention with supervised contrastive learning (SA+SCL) for the fault-location classification task. In panels **d-f**, labels 0-15 correspond to the 16 fault-location categories. **g** t-SNE visualization of the encoder-output feature embeddings for the fault-type classification task. **h** t-SNE visualization of the feature embeddings after SA for the fault-type classification task. **i** t-SNE visualization of the feature embeddings after SA+SCL for the fault-type classification task. In panels **g-i**, labels 0-9 correspond to the 10 fault-type categories. All t-SNE plots (**d-i**) are generated using identical hyperparameters (perplexity = 30, learning rate = 200, iterations = 1000). The use of self-attention improves cluster compactness, while the addition of supervised contrastive learning further enhances intra-class compactness and inter-class separability, leading to more structured and distinguishable feature clusters.

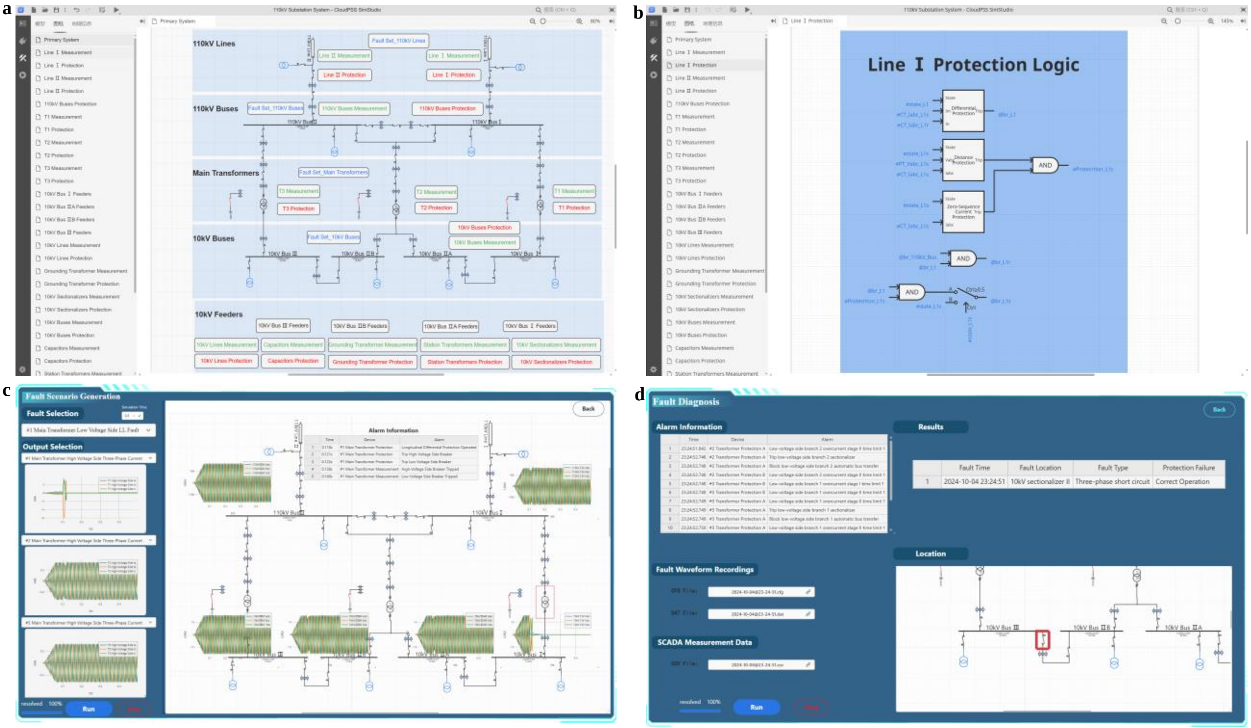

Figure 3. Architecture and functional modules of the digital twin platform. a Primary system model constructed in CloudPSS, reproducing the electrical network configuration of the substation, including power sources, busbars, main transformers, loads, capacitors, grounding transformers, circuit breakers, and instrument transformers (CTs/PTs), to emulate the operational behavior of primary equipment. **b** Secondary protection system model reflecting the actual relay settings, device configurations, and protection logic deployed in the substation, enabling realistic simulation of interactions among secondary devices. **c** Automated fault scenario generation module used to create diverse fault cases for training and validation, ensuring broad coverage of operating conditions and fault patterns. **d** Fault diagnosis module integrating the proposed learning framework to perform fast fault localization, fault-type classification, and protection-failure analysis within the digital twin environment.

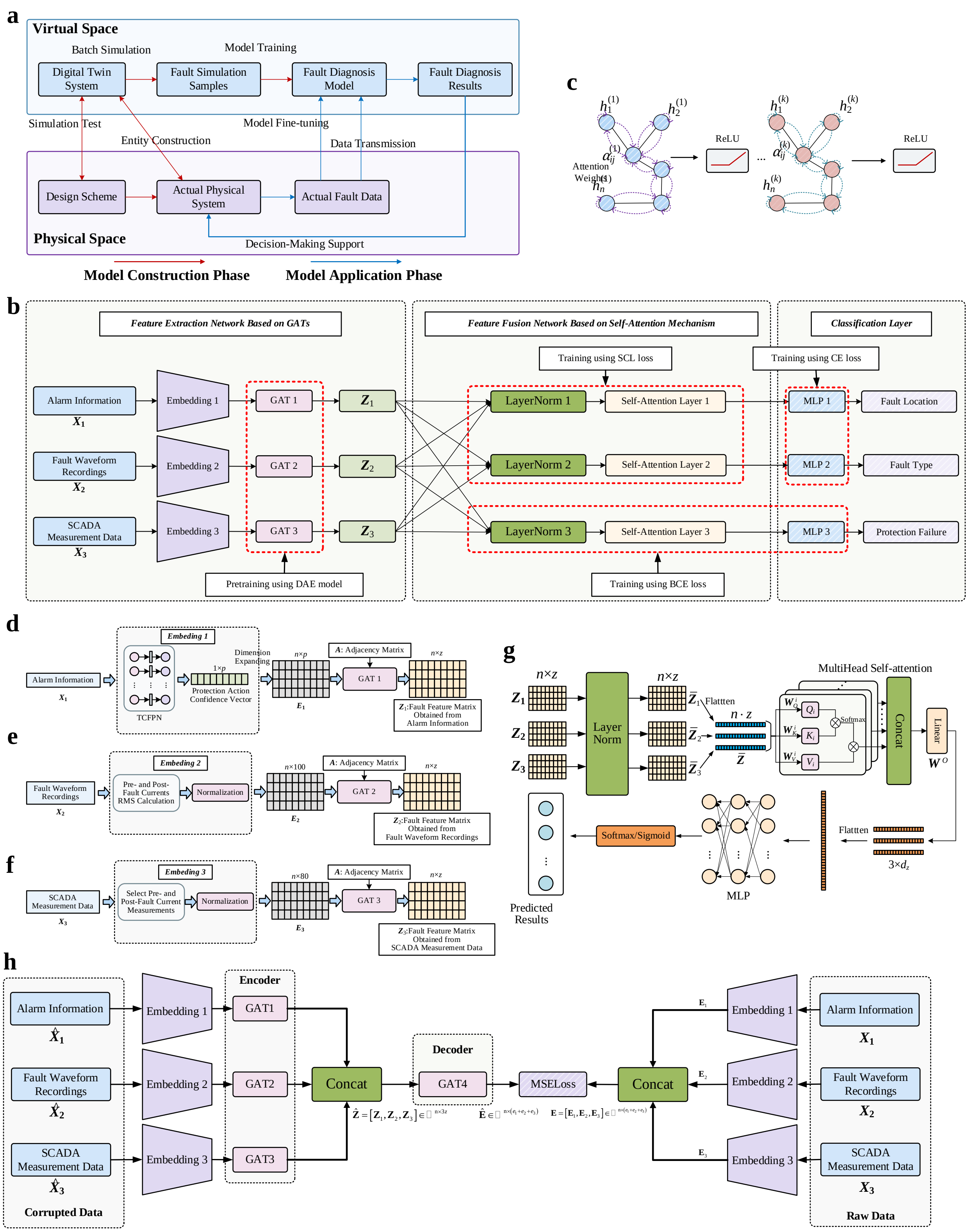

Figure 4. Overall framework and model architecture of the proposed method. a Digital twin-driven fault diagnosis framework. In the model construction phase, a digital twin is built to simulate operating and fault scenarios and to support training of the multi-modal fusion model; in the model application phase, real-time substation data are synchronized to the virtual space for fault inference, decision support, and continuous model refinement, forming a closed-loop cyber-physical system. **b** Architecture and training strategy of the proposed model. The model comprises a GAT-based multi-modal feature extractor, a task-specific self-

attention fusion module, and MLP classifiers. After DAE pretraining for noise-robust initialization, downstream training optimizes the self-attention layers with SCL and the classifier with CE loss for fault location and fault-type tasks, while the multi-label protection-failure task is trained directly using BCE loss. **c** GAT message-passing mechanism. Each node aggregates attention-weighted messages from its neighbors to learn topology-aware and context-dependent representations. **d** Feature extraction network for alarm information. Alarm data are processed using a TCFPN to obtain relay-action confidence vectors, which are expanded and encoded by GATs to produce alarm feature matrices. **e** Feature extraction network for fault waveform recordings. RMS values of three-phase and zero-sequence currents over pre- and post-fault windows are normalized and passed through GATs to extract waveform-based features. **f** Feature extraction network for SCADA measurements. Normalized current measurements at each primary node, including pre- and post-fault samples, are encoded by GATs to obtain SCADA feature matrices. **g** Self-attention-based feature fusion network. Alarm, waveform, and SCADA features are normalized, concatenated, and fused via multi-head self-attention to model global cross-modal dependencies, followed by an MLP for task-specific prediction (Softmax for location/type; Sigmoid for protection failure). **h** DAE pretraining structure. A GAT-based encoder-decoder DAE is used to learn noise-robust latent representations from augmented multi-modal inputs.

Q9. (Remarks on code availability): At this point I have not reviewed the code since the paper still requires quite some work in my opinion.

A9. We sincerely thank the reviewer for this comment and fully understand the reviewer’s decision not to review the code at this stage. As noted, the manuscript previously required substantial clarification and improvement, and we agree that it is appropriate to reassess the code only after the paper itself reaches a more mature version.

In preparation for future review and to ensure full reproducibility, we have significantly enhanced the codebase accompanying the revised manuscript. In particular:

- All baseline model implementations (MLP-based, ResNet-based, LSTM-based, GCN-based, Transformer-based, and MPNN variants) have been fully added, organized, and verified to ensure that every comparison reported in the paper can be reproduced end-to-end.
- The entire project structure has been reorganized, with clear module separation (data generation, preprocessing, model architectures, training pipeline, evaluation scripts, utilities).
- The README file has been expanded substantially, now including:
  - environment setup
  - detailed instructions for dataset reproduction
  - scripts for training and evaluating each baseline
  - how to reproduce tables and figures in the manuscript
- Additional comments have been added to all key modules, including the GAT encoder, multimodal fusion block, DAE pretraining, and SCL implementation.
- The CloudPSS simulation project and dataset generation scripts have been updated, ensuring full consistency with the results in the manuscript.

The computer code is available via Code Ocean at: https://doi.org/10.24433/CO.6548109.v3

Catalogs

RESPONSES TO REVIEWERS AND REVISIONS BASED ON THE COMMENTS 2
Response to Reviewer #1 2
Response to Reviewer #2 3
Response to Reviewer #3 7
Q1 7
Q2 7
Q3 8
Q4 9
Q5 10
Q6 10
Q7 11
Q8 12
Response to Reviewer #4 13
Response to Reviewer #5 14
Q1 14
Q2 16

RESPONSES TO REVIEWERS AND REVISIONS BASED ON THE COMMENTS

Response to Reviewer #1

Review Comments:

No further comment.

Our Response:

We sincerely thank the reviewer for the careful evaluation of our revised manuscript and for the positive feedback. We greatly appreciate the reviewer's time and effort in assessing our work.

Response to Reviewer #2

Review Comments:

The manuscript presents several noteworthy results that advance understanding in the field, particularly through its key finding/method/result, which appears to extend existing work in a meaningful way.

Overall, the study has the potential to be significant both to the core field and to related disciplines, and it compares favorably with established literature, although clearer positioning relative to prior studies would strengthen the contribution.

The data largely support the authors' main conclusions; however, in some cases the claims appear broader than what is directly evidenced, and additional justification or clarification would be beneficial. The methodology is generally sound and consistent with current standards in the field, though certain aspects of the data analysis and interpretation would benefit from further explanation to rule out alternative interpretations. Importantly, while the methods are mostly well described, additional detail in specific steps would improve reproducibility. Overall, the revised revision is solid.

Our Response:

We sincerely thank the reviewer for the positive and constructive evaluation of our work. We greatly appreciate the recognition of the manuscript's contribution and its potential significance to both the core field and related disciplines. Following the reviewer's suggestions, we have carefully revised the manuscript to further clarify the positioning, strengthen the justification of certain claims, and enhance methodological transparency. The major revisions are summarized as follows:

- **Clearer positioning relative to prior studies**

To better position our work within the existing literature, we have **added a summary table (Supplementary Table 1) that systematically reviews representative rule-based and data-driven fault diagnosis methods**, including expert systems, Bayesian networks, fuzzy and rough-set models, Petri nets, D–S evidence–enhanced neural networks, GCN/GNN-based approaches, CNN–Transformer hybrids, and MPNN-based models.

The table provides a structured comparison in terms of method category, input modalities, supported diagnostic tasks, reported accuracy, robustness characteristics, generalization capability, and model construction or update cost. This comparative overview clarifies the methodological landscape and highlights the distinctive aspects of the proposed framework—namely, the unified integration of topology, alarm, SCADA, and waveform information within a single model, and its ability to jointly address fault location, fault type classification, and protection failure detection with demonstrated robustness to noise and incomplete data.

The corresponding revisions in the Manuscript:

Introduction (Line 62-74)

……

To highlight the conceptual advantages of the proposed approach, we provide a comparison with

representative existing methods (Supplementary Table 1).

Supplementary Table 1: Comparison of existing fault diagnosis methods for power systems

Method	Type	Input Data	Main Task	Accuracy	Robustness	Generalization	Model Construction Cost	Model Update Cost
Expert Systems[1]	Rule-based	Alarm	Fault location	Moderate	Moderate–Low (sensitive to missing alarms)	Limited (rules tied to specific operation conditions)	High (manual rule-based construction)	High (frequent manual updates required)
Bayesian Networks[2]	Rule-based	Alarm	Fault location	Moderate	Moderate (probabilistic reasoning handles uncertain alarms)	Moderate (structure fixed; parameters adaptable)	Medium (requires expert-defined BN structure)	Medium (parameter tuning needed)
Fuzzy Sets[3]	Rule-based	Topology + Alarm	Fault location	Moderate–High	Moderate–High (tolerant to vague or missing alarms)	Moderate (adapts to topology through incidence matrices)	Low–Medium (simple fuzzy matrices)	Medium (rules easy to adjust)
Rough Set[4]	Rule-based	Alarm	Fault location + fault type classification	Moderate–High	Moderate–High (rule reduction enhances stability)	Moderate (rules discovered from data, not topology-dependent)	Medium–Low (automatic rule extraction)	Medium (compact rule-base, low maintenance)
Analytical Models[5]	Rule-based	Alarm	Fault location + protection failure detection	Moderate–High	Moderate–High (robust to redundant alarms; timestamp utilization)	Moderate (supports varied protection schemes & bus configurations)	Medium (analytic formula only)	Medium (only configuration-related adjustments)
Petri Nets[6]	Rule-based	Alarm	Fault location + protection failure detection	Moderate–High	Moderate–High (handles fuzzy evidence)	Moderate (temporal + fuzzy transitions work in many scenarios)	Medium (Petri net modeling required)	Medium (net structure updates moderately costly)
Hierarchical Diagnosis[7]	Rule-based	Alarm + SCADA	Fault location + protection failure detection	High	High (multi-source fusion mitigates conflicting information)	Good (layered use of switching + electrical info)	Medium–High (two-layer diagnostic modeling)	Medium (both layers must be updated)
DS Evidence + BP NN[8]	Data-driven	Fault waveform recordings	Busbar fault type classification	High	High (DS fusion reduces uncertainty before NN training)	Good (learns busbar patterns independent of topology)	Medium (requires DS preprocessing + NN training)	Medium (NN retraining needed for new waveform patterns)
GCNs[9]	Data-driven	Topology + SCADA	Fault location	Very High	Very High (robust to noise, data loss, topology variations)	Very good (graph learning generalizes across networks)	Medium–High (graph modeling + GNN training)	Medium (moderate retraining for topology changes)
CNN-based Transformer[10]	Data-driven	Fault waveform recordings	Fault location + fault type classification	Very High	Very High (attention mechanism resists waveform distortions)	Good (long-term temporal modeling adapts across conditions)	Medium–High (complex hybrid deep model)	Medium (retraining needed for distribution shifts)
R-GNNs[11]	Data-driven	Topology + Fault waveform recordings	Fault location + fault type classification	Very High	Very High (captures spatial–temporal dependencies reliably)	Very good (generalizes across operating modes & topologies)	Medium–High (recurrent GNN architecture)	Medium (requires updates for major topology changes)
MPNN[12]	Data-driven	Topology + Fault waveform recordings	Fault location + fault type classification	Very High	Very High (resilient to topology variations & missing data)	Very good (Graph-Lasso auto-identifies topology)	Medium–High (joint MPNN + topology estimation)	Low–Medium (auto topology update reduces manual work)
Proposed Method	Data-driven	Topology + Alarm + SCADA + Fault waveform recordings	Fault location + fault type classification + protection failure detection	Excellent	Excellent (multi-modal fusion + attention; resistant to noise, missing data)	Excellent (digital twin + graph attention adapt to topology, data quality, and real scenarios)	High (digital twin modeling + multi-modal architecture)	Low–Medium (digital twin reduces update workload)

- **Refinement of claims, scope, and interpretation**

We thank the reviewer for these important comments. In response, we have carefully revised the Results section (Lines 206–258) to ensure that all conclusions remain proportionate to the empirical evidence and clearly bounded within the evaluated scenarios.

First, we have moderated statements that could previously be interpreted as overly broad and explicitly clarified the practical scope of the proposed method. In particular, **we contextualize the observed accuracy–efficiency trade-off within substation operational workflows and avoid generalizing beyond the studied conditions.** The revised text now **specifies the experimental settings under which performance advantages are demonstrated**. Second, to address potential alternative interpretations, **we have expanded the analysis to disentangle the contributions of structural modeling capability, multi-modal information integration, and robustness under different data degradation mechanisms.**

These revisions strengthen the logical interpretation of the findings and ensure that the claims are fully supported by the presented evidence.

The corresponding revisions in the Manuscript:

Results (Line 206–258)

Comparison of fault diagnosis models

……

The proposed model consistently outperforms all baseline approaches under varying levels of noise, sampling loss, and sensor failure (Tables 1–3, Fig. 1d–f, Supplementary Fig. 3–5, Supplementary Data). While it incurs moderate additional computational overhead (Supplementary Table 7), the overall training and inference costs remain within a practically deployable range. From an operational perspective, the observed accuracy–efficiency trade-off should be interpreted in the context of practical substation workflows. Protection relays typically isolate faults within milliseconds, and subsequent diagnostic analysis is conducted after system stabilization using monitoring data collected from multiple sources. Under such post-event conditions, both the proposed model and the baseline methods operate within a sub-second inference range, which is sufficient for practical deployment. Given the safety-critical nature of substations, even moderate improvements in diagnostic accuracy can enhance operational reliability and reduce the risk of misdiagnosis or missed faults. Therefore, while computational efficiency remains an important consideration, the additional resource requirements of the proposed model are considered acceptable in applications where diagnostic robustness and precision are prioritized.

The comparative results can be interpreted from both structural modeling capability and architectural design perspectives.

TCFPN relies solely on alarm information and performs rule-based reasoning without learning from multi-modal data. Although it reflects predefined protection logic, multiple fault scenarios may produce highly similar alarm patterns, and rule-based inference cannot resolve such ambiguities. Consequently, its outputs remain largely invariant across certain test conditions, indicating limited discriminative capability and weak adaptability to noisy or incomplete inputs.

MLP-, LSTM-, and Transformer-based models do not explicitly encode electrical topology. MLP treats inputs as flattened feature vectors, LSTM emphasizes temporal dependencies, and Transformer models token-level relationships through self-attention. While these architectures possess strong representation capacity, they lack structural inductive bias reflecting electrical connectivity. Therefore, when system topology changes, their learned correlations may not generalize effectively, resulting in reduced robustness under unseen topology configurations. ResNet-based model enhances feature extraction through convolutional operations and achieves relatively competitive performance. However, convolution is defined on regular Euclidean grids and cannot accurately represent the irregular and non-uniform connectivity of substation components. As a result, although ResNet captures local spatial patterns, it cannot explicitly model topology-dependent fault propagation paths.

Among the graph-based models, MPNN explicitly incorporates topology through message passing between connected nodes and utilizes waveform information for diagnosis. This structural modeling enables better performance than non-graph models in topology-sensitive tasks. However, MPNN does not incorporate alarm information and is trained in a single end-to-end manner without staged pretraining, which limits its ability to diagnose protection failures and reduces robustness under noisy or incomplete inputs. GCN-based model also encodes topology via graph convolutions and achieves stronger overall performance than MPNN. By aggregating neighborhood information layer by layer, GCN more effectively captures structural dependencies among components. Nevertheless, its aggregation mechanism is homogeneous, applying fixed normalization and uniform weighting to neighboring nodes. This limitation makes it less effective than GAT in modeling the directional and heterogeneous dependencies among substation components. In contrast, the proposed GAT-based fusion mechanism performs adaptive, node-wise attention and integrates topology, waveform, SCADA, and alarm information more effectively, yielding the highest overall accuracy.

In addition to architectural differences, it is important to analyze how different data degradation mechanisms affect model performance. To further understand this effect, we compare model performance under sample loss and sensor failure conditions. The impacts of sample loss and sensor failure differ due to their distinct degradation mechanisms. Sample loss removes partial time-series data across alarm, SCADA, and waveform modalities, whereas sensor failure drops entire electrical measurement channels while preserving alarm information. Therefore, the performance variation depends on the modality importance of each task. For fault-location, most models rely more on electrical measurements, making sensor failure more detrimental, except for the TCFPN model, which depends solely on alarm data. For fault-type classification, electrical measurements dominate, and sensor failure leads to greater degradation. In contrast, protection failure detection relies primarily on alarm signals, making it more sensitive to sample loss.

……

- **Improved methodological transparency and reproducibility**

To further enhance methodological transparency and reproducibility, we have ensured that each major step of the proposed framework is explicitly documented in the released codebase. **All implementation steps are clearly structured and accompanied by detailed inline comments to facilitate traceability and understanding.** This comprehensive documentation enables other researchers to reproduce the reported results and provides a clear reference for extending or adapting the framework to related applications.

Response to Reviewer #3

Review Comments:

Authors have amended the manuscript with good effort. Nevertheless, there are still multiple aspects which needs more attention. My comments can be found below:

Q1. Although I appreciate that authors are providing some key results in the abstract, I find it too specific at the moment even to include inference time and GPU model. The abstract needs to be critically re-written to better reflect the work.

A1. We sincerely thank the reviewer for this constructive comment. We understand the concern that the current abstract contains implementation-specific details (e.g., inference time and GPU model), which may be overly technical and detract from the broader scientific contributions of the work. We agree that the abstract should focus more on the methodological innovation and overall performance rather than deployment-level specifications.

In response, **we have critically revised and streamlined the abstract to improve its clarity and balance.** Specifically, we removed hardware-specific details and refined the description of system performance to emphasize methodological contributions, overall effectiveness, and practical significance. The revised abstract now better reflects the scope, novelty, and impact of the study.

The corresponding revisions in the manuscript:

Abstract (Line 9–19)

Substations are critical infrastructures for ensuring reliable power system operation. With increasing digitalization and system complexity, the rapid growth of multi-source data poses significant challenges for accurate and timely fault diagnosis. Existing approaches often struggle to effectively integrate heterogeneous data or adapt to varying operating conditions. To address these limitations, this study proposes a digital twin-driven fault diagnosis framework incorporating a multi-modal fusion model that integrates system topology, alarms, fault waveforms, and SCADA data through Graph Attention Networks and self-attention mechanisms. In this work, the method is validated on a 110 kV substation using 11,597 training and 3,890 testing scenarios generated in CloudPSS. Experimental results demonstrate over 95% accuracy in fault location, 97% in fault type identification, and 90% accuracy in protection failure detection under 30% data loss conditions. The deployed digital twin system further verifies the practical feasibility of the proposed approach, highlighting its robustness in complex operating environments.

Q2. From the newly amended manuscript, I do not understand the motivation behind adding zero-mean Gaussian noise with specific signal-to-noise ratio. Also, looking at the performance results, it seems that zero-mean Gaussian noise does not do too much. It would be preferred if more irregular noises would be used as a study. Perhaps authors can justify.

A2. We sincerely thank the reviewer for this valuable comment. **Measurement noise is unavoidable in practical substations**, particularly in SCADA measurements and fault waveform recordings, due to sensor imperfections, communication interference, and electromagnetic disturbances. Such

noise directly affects the quality of diagnostic data. Therefore, **evaluating the robustness of the proposed method under noisy conditions is essential to ensure its practical applicability and real-world reliability.**

In this study, additive zero-mean Gaussian noise with specified SNR levels is introduced to emulate measurement noise. **In power system research, measurement errors are conventionally modeled as Gaussian in state estimation and signal processing frameworks.** For example, in hybrid SCADA-based state estimation, the measurement error vector is typically assumed to follow a zero-mean Gaussian distribution with known covariance, which underpins weighted least squares estimation. Similarly, classical estimators such as Kalman filter variants are derived under Gaussian noise assumptions to achieve minimum mean-square error performance. Therefore, **Gaussian noise serves as a standard and widely accepted baseline model for evaluating algorithm robustness.**

We acknowledge that real substation measurement noise may deviate from ideal Gaussian assumptions and can include more irregular forms such as impulsive or heavy-tailed noise. However, **modeling and handling such irregular noise types generally fall within the research domain of bad data detection and robust estimation, which involves dedicated noise identification and cleansing frameworks.** These topics are beyond the scope of the present study. In this work, Gaussian noise is adopted as a representative baseline to enable controlled robustness evaluation, while more complex noise modeling is left for future investigation.

Q3. Regarding Figure 1, it is appreciated that there are now numbers on the nodes in fig 1b. However, are these numbers corresponding to fig 1a? In this case, it would be best to also number figure 1a.

A3. We sincerely thank the reviewer for this careful observation. We understand the concern regarding the consistency between Fig. 1a and Fig. 1b and agree that clearer correspondence between the two subfigures would improve readability and interpretation.

The numbers shown on the nodes in Fig. 1b indeed correspond directly to the components illustrated in Fig. 1a. To improve clarity and avoid potential confusion, **we have added the corresponding numbering to Fig. 1a as well, ensuring consistency between the two subfigures.**

The corresponding revisions in the Manuscript:

Figure 1

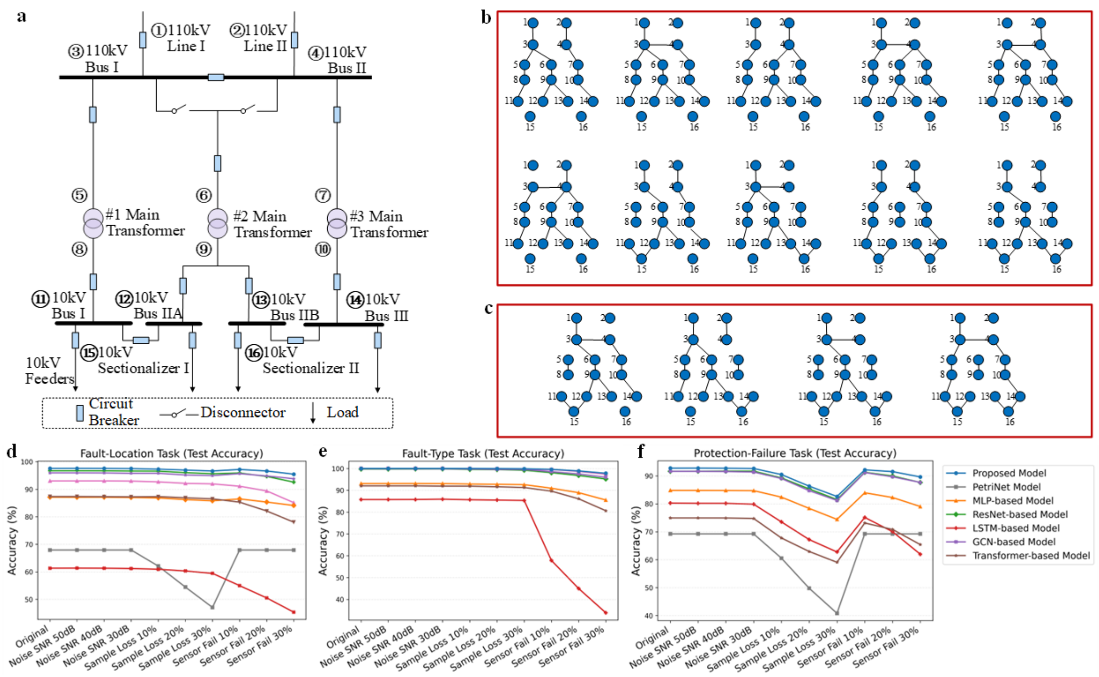

Q4. Regarding the accuracy in Fig 1d,e,f, I find it curious that accuracy due to sensor fail are in some case better than the accuracy of sample loss. This is not natural, could authors explain this?

A4. We sincerely thank the reviewer for this insightful comment. We understand that, at first glance, it may appear counterintuitive that, in some cases, the accuracy under simulated sensor failure is higher than that under sample loss. We appreciate the opportunity to clarify this phenomenon.

As described in the manuscript, the two data degradation strategies differ fundamentally. **Sample loss** was introduced by randomly removing 10%, 20%, and 30% of time-series data points from alarm, SCADA, and waveform data, thereby **affecting all modalities simultaneously**. In contrast, **sensor failures** were simulated by randomly dropping 10%, 20%, and 30% of entire SCADA or waveform channels, **while alarm data remained intact**. Due to this distinction, the performance impact depends strongly on the relative importance of different modalities for each task.

Specifically, the observed behavior differs across the three tasks shown in Fig. 1d–f:

Fig. 1d (Fault location task):

For most models, the **fault-location task relies more heavily on electrical measurement modalities (SCADA and waveform data)**. Therefore, **under sensor failure (entire channel loss), the performance degradation is generally more pronounced than under sample loss (partial sampling loss)**. The **only exception is the Petri Net model, which relies solely on alarm information.** Since alarm data are partially removed under the sample loss setting but remain intact under simulated sensor failure, the Petri Net model exhibits performance degradation only in the sample loss case. Other multi-modal models do not show this behavior.

Fig. 1e (Fault type classification task):

This task depends predominantly on electrical measurement data rather than alarm information. As shown in the ablation study, removing the alarm modality has minimal impact on fault-type classification accuracy. Consequently, sensor failure (loss of entire electrical channels) generally causes larger accuracy degradation than sample loss. Therefore, **the phenomenon noted by the reviewer does not occur in this task.**

Fig. 1f (Protection failure detection task):

In contrast, **alarm information plays a dominant role in protection failure detection.** Since the sample loss setting includes partial removal of alarm data, it leads to more significant performance degradation. Under simulated sensor failure, alarm data remain fully available, allowing the model to retain strong diagnostic cues. As a result, **the phenomenon is particularly evident in this task.**

Overall, the differences arise from the distinct degradation mechanisms and the task-dependent importance of different data modalities. **To improve clarity, we have added further explanation in the revised manuscript to explicitly describe these modality- and task-dependent effects.**

The corresponding revisions in the Manuscript:

Results (Line 249–258)

Comparison of fault diagnosis models

……

In addition to architectural differences, it is important to analyze how different data degradation mechanisms affect model performance. To further understand this effect, we compare model performance under sample loss and sensor failure conditions. The impacts of sample loss and sensor failure differ due to their distinct degradation mechanisms. Sample loss removes partial time-series data across alarm, SCADA, and waveform modalities, whereas sensor failure drops entire electrical measurement channels while preserving alarm information. Therefore, the performance variation depends on the modality importance of each task. For fault-location, most models rely more on electrical measurements, making sensor failure more detrimental, except for the TCFPN model, which depends solely on alarm data. For fault-type classification, electrical measurements dominate, and sensor failure leads to greater degradation. In contrast, protection failure detection relies primarily on alarm signals, making it more sensitive to sample loss.

Q5. There are a lot of problematic symbols in the pdf in Eq 3, 4 and their explanation. Please fix it.

Q6. Fig 4h also has some problem in the symbols. Please look into this.

A5. & A6. We sincerely apologize for these formatting issues and thank the reviewer for carefully identifying them. The problematic symbols in Eq. (3), Eq. (4), their explanations, and Fig. 4h were caused by formatting errors during the automatic conversion of the submitted Word document into PDF by the journal’s submission system, which resulted in incorrect rendering of certain mathematical symbols.

We have carefully corrected all affected equations, figure labels, and related explanations in the revised manuscript. In addition, we have thoroughly reviewed the entire manuscript to ensure that all mathematical expressions and symbols are accurately displayed and consistently formatted.

The corresponding revisions in the Manuscript:

Fig. 4h

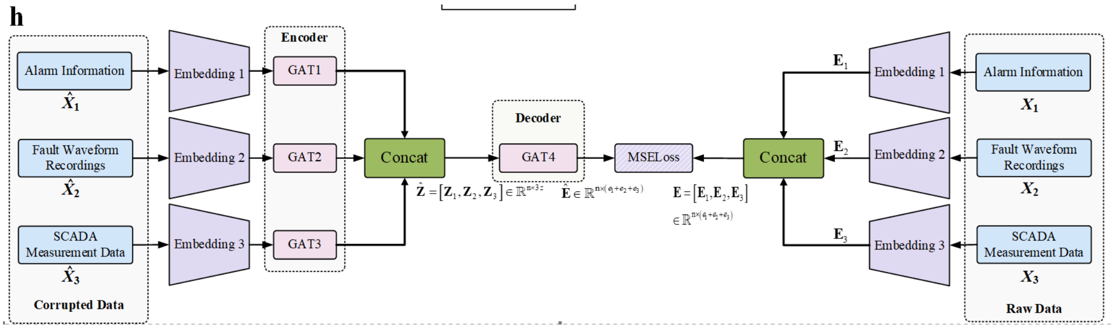

$$e_{ij} = \mathrm{LeakyReLU}\left(\mathbf{a}^{\mathrm{T}}\left[\mathbf{W}h_i \,\|\, \mathbf{W}h_j\right]\right) \quad (1)$$

$$\overline{\mathbf{Z}}_i = \frac{\mathbf{Z}_i - \mu_i}{\sigma_i + \varepsilon} \odot \gamma + \beta, \quad i = 1,2,3 \quad (2)$$

Q7. Authors gave some very reasonable explanation on the rationale on the model architecture and data fusion approach, however this is not highlighted in the model training strategy section (and sections below). I would appreciate if authors highlight the rationale on the model training.

A7. We sincerely thank the reviewer for this valuable suggestion. In the original manuscript, the rationale behind the training strategy was not sufficiently emphasized. **In the revised version, we have explicitly clarified the motivations for the two-stage training framework,** including the use of unsupervised DAE pretraining for noise-robust and topology-aware feature learning, freezing the pretrained encoder to preserve stable representations, and adopting task-specific optimization strategies (SCL + CE for single-label tasks and BCE for multi-label protection failure classification). These additions better highlight the principled design of the model training strategy.

The corresponding revisions in the Manuscript:

Methods

Model training strategy (Line 625–631)

……This training design is motivated by two key considerations. First, substation fault data are inherently noisy, incomplete, and heterogeneous across modalities. Direct end-to-end supervised training under such conditions may lead to unstable optimization and modality bias. Therefore, an unsupervised pretraining stage is introduced to learn noise-robust and topology-aware feature representations before task-specific optimization. Second, the three downstream tasks exhibit different label structures and modality dependencies, requiring tailored optimization strategies to ensure discriminative and stable learning for each task.

Denoising autoencoder pretraining (Line 647–651)

……Compared with purely supervised pretraining, the DAE enables the encoder to capture intrinsic structural dependencies and cross-modal correlations without relying on labeled fault categories. Freezing the

pretrained encoder during downstream optimization prevents catastrophic forgetting and preserves the learned topology-aware and noise-robust representations, ensuring stable transfer to task-specific learning.

Downstream tasks training (Line 671–677)

This study focuses on single-point faults, formulating both the primary fault location and fault type diagnosis tasks as multi-class classification problems. To enhance feature discrimination and improve the stability of model optimization, the training of the self-attention layers and classification layers is organized into two consecutive optimization procedures. In the first procedure, SCL[51] is applied to train the self-attention layers, enhancing the discriminative capability of the learned feature representations. SCL promotes feature separability by pulling samples of the same class closer together while pushing samples of different classes farther apart in the feature space.

……

(Line 691–696)

For protection failure classification, the task is modeled as a multi-label classification problem, since a single fault event may involve simultaneous failures of multiple protection relays. In this task, the multi-label nature introduces label overlap and dependency among protection relays, making it difficult to define unambiguous positive and negative pairs required for contrastive learning. Therefore, directly optimizing the model using Binary Cross-Entropy (BCE) loss provides a more appropriate objective for capturing independent yet potentially correlated relay failure events.

……

Q8. (Remarks on code availability): The code is available on oceancode. It has been improved for clarity according to the revision.

A8. We sincerely appreciate the reviewer's acknowledgment of the improved code clarity and availability. We are glad that the revised version meets the reviewer's expectations.

Response to Reviewer #4

Review Comments:

My comments were properly addressed.

Our Response:

We sincerely thank the reviewer for the careful re-evaluation of our manuscript. We greatly appreciate the reviewer’s constructive comments in the previous round, which have helped us improve the quality and clarity of the manuscript. We are pleased that our revisions have satisfactorily addressed the reviewer’s concerns.

Response to Reviewer #5

Review Comments:

The manuscript addressed some of the raised questions and concerns. However, some questions remain.

Q1. Related to Q3.

The authors' response mentions that edge connectivity is indeed used to define the graph structure via an adjacency matrix derived from switching states. However, this aspect is standard and essentially implicit in any GNN-based model: a graph neural network necessarily operates on an adjacency structure, and representing electrical connectivity through edges does not in itself constitute additional or novel edge information.

More importantly, the response does not fully address the fairness of the comparative evaluation. If topology and edge connectivity are central to fault propagation and protection-zone modelling, as the authors correctly argue, then comparing a topology-aware GAT against baselines (MLP, LSTM, Transformer) that do not have access to this structural information in the form of edge features is inherently unbalanced. The observed performance degradation of these non-graph models under unseen topologies is therefore expected and cannot be attributed solely to modelling limitations, but rather to the absence of critical topological input.

While the inclusion of graph-based baselines such as GCN and MPNN is appropriate and more informative, the manuscript should more clearly distinguish between:

- Performance gains due to explicit access to graph topology, and
- Performance gains due to the adaptive attention mechanism of GAT.

A1. We sincerely thank the reviewer for this insightful and technically important comment.

First, we agree that representing electrical connectivity through an adjacency matrix does not, by itself, constitute additional or novel edge information in a GNN framework. In this work, **we indeed do not introduce explicit edge features beyond connectivity.** The main reason is rooted in physical and engineering considerations. In substation fault diagnosis, the three data sources—alarm signals, SCADA measurements, and waveform recordings—are naturally associated with physical equipment (lines, transformers, breakers, buses), and therefore can be clearly and meaningfully embedded as node features with well-defined physical interpretations. In contrast, **it is difficult to define edge features that are simultaneously applicable and physically meaningful across all three modalities.** For example, waveform and SCADA measurements correspond to device-level electrical quantities, while alarms reflect protection actions tied to specific components rather than to inter-component relationships. Consequently, instead of constructing artificial or loosely defined edge attributes, we use the switching-state-derived adjacency matrix to encode the strong electrical coupling and protection-zone relationships between components, which are central to fault propagation paths and protection coordination. This design **ensures physical interpretability while avoiding the introduction of heuristic edge features without clear engineering meaning.**

Second, regarding the fairness of the comparative evaluation, we acknowledge the reviewer's point that non-graph models (MLP, LSTM, Transformer) do not explicitly incorporate topology and therefore are at a disadvantage under unseen topologies. However, this comparison is intentional and reflects a practical modeling distinction. Many existing data-driven diagnostic methods treat substations as flat feature vectors or temporal sequences without explicitly encoding structural topology. **Our goal is to demonstrate that when topology is critical to fault propagation and protection-zone modeling, explicitly embedding graph structure leads to improved generalization under topology changes.** Therefore, the observed degradation of non-graph models under unseen topologies is not attributed solely to inferior modeling capacity, but rather to their lack of explicit structural inductive bias. To make this distinction clearer, **we have revised the Results section to separate the analysis into two levels: (i) graph-based models vs. non-graph models, highlighting the performance gains due to explicit access to topology; and (ii) GAT vs. other GNN variants (e.g., GCN, MPNN), isolating the additional improvements brought by the adaptive attention mechanism.** This revised analysis more clearly distinguishes performance gains from structural awareness and those from the attention-based aggregation strategy.

The corresponding revisions in the Manuscript:

Results (Line 221–248)

Comparison of fault diagnosis models

……

TCFPN relies solely on alarm information and performs rule-based reasoning without learning from multi-modal data. Although it reflects predefined protection logic, multiple fault scenarios may produce highly similar alarm patterns, and rule-based inference cannot resolve such ambiguities. Consequently, its outputs remain largely invariant across certain test conditions, indicating limited discriminative capability and weak adaptability to noisy or incomplete inputs.

MLP-, LSTM-, and Transformer-based models do not explicitly encode electrical topology. MLP treats inputs as flattened feature vectors, LSTM emphasizes temporal dependencies, and Transformer models token-level relationships through self-attention. While these architectures possess strong representation capacity, they lack structural inductive bias reflecting electrical connectivity. Therefore, when system topology changes, their learned correlations may not generalize effectively, resulting in reduced robustness under unseen topology configurations. ResNet-based model enhances feature extraction through convolutional operations and achieves relatively competitive performance. However, convolution is defined on regular Euclidean grids and cannot accurately represent the irregular and non-uniform connectivity of substation components. As a result, although ResNet captures local spatial patterns, it cannot explicitly model topology-dependent fault propagation paths.

Among the graph-based models, MPNN explicitly incorporates topology through message passing between connected nodes and utilizes waveform information for diagnosis. This structural modeling enables better performance than non-graph models in topology-sensitive tasks. However, MPNN does not incorporate alarm information and is trained in a single end-to-end manner without staged pretraining, which limits its ability to diagnose protection failures and reduces robustness under noisy or incomplete inputs. GCN-based model also encodes topology via graph convolutions and achieves stronger overall performance than MPNN.

By aggregating neighborhood information layer by layer, GCN more effectively captures structural dependencies among components. Nevertheless, its aggregation mechanism is homogeneous, applying fixed normalization and uniform weighting to neighboring nodes. This limitation makes it less effective than GAT in modeling the directional and heterogeneous dependencies among substation components. In contrast, the proposed GAT-based fusion mechanism performs adaptive, node-wise attention and integrates topology, waveform, SCADA, and alarm information more effectively, yielding the highest overall accuracy.

Q2. Related to Q5.

The expanded set of baselines improves the breadth of the experimental evaluation. However, the revised results raise an important issue that is currently not sufficiently analysed or discussed in the manuscript.

Notably, the ResNet-based baseline achieves performance very close to that of the proposed method across multiple diagnostic tasks, while exhibiting substantially lower computational requirements. Based on the reported results, the ResNet-based model demonstrates:

- Comparable fault localisation and type classification accuracy,
- Approximately half the inference time,
- Lower training time, and
- Reduced GPU memory usage.

This observation is particularly important in the context of real-time substation fault diagnosis and protection applications, where inference latency, computational efficiency, and system reliability are often as critical as marginal accuracy improvements. In such operational settings, faster and more resource-efficient models may be preferable if their performance is comparable.

The manuscript primarily emphasises accuracy gains, without addressing this accuracy–efficiency trade-off. A more critical discussion is needed to clarify:

- Under which operating conditions or fault scenarios does the proposed method provide clear and practically meaningful advantages over simpler architectures such as ResNet, and
- Whether the additional architectural complexity and computational cost of the proposed method are justified for real-time or near–real-time deployment in substations.

A2. We sincerely thank the reviewer for raising the important issue of the trade-off between diagnostic accuracy and computational efficiency. We agree that the ResNet-based model demonstrates appealing efficiency advantages, including faster inference, reduced training time, and lower GPU memory usage. These results confirm that simpler architectures can provide competitive performance with improved computational efficiency.

However, in the context of substation fault diagnosis, several practical considerations justify prioritizing diagnostic precision while maintaining acceptable computational cost:

- **Model Training and Deployment in Substation Systems:**

Substations are critical infrastructure and represent relatively stable physical systems. Fault diagnosis models therefore **do not require frequent retraining or online learning**. In practical deployment, **the model training is carried out in the cloud**, where computational resources are abundant and **not constrained by on-site hardware limitations.** This allows for more complex models, such as the proposed GAT-based architecture, to be trained without concerns about local resource constraints. For inference, although the proposed model introduces additional computational overhead, **its latency remains within the sub-second range and does not exceed the capability of typical substation monitoring servers.** Therefore, the increased architectural complexity does not prevent practical deployment under normal hardware configurations.

- **Accuracy–Efficiency Trade-off:**

In real-world operation, protection relays isolate faults within milliseconds, ensuring immediate system protection. The subsequent fault diagnosis process is typically conducted after system stabilization using uploaded SCADA data, alarm signals, and waveform information. Although the ResNet baseline achieves lower inference latency, both models operate within the sub-second range (hundreds of milliseconds). **In post-event diagnostic scenarios, such latency differences are unlikely to influence operational workflows or decision timelines.** In practice, human operators often require several minutes to analyze diagnostic results and implement corrective actions, further reducing the practical impact of sub-second inference differences. More importantly, **the proposed method consistently achieves the highest accuracy across all evaluated tasks**, with improvements of up to approximately 3% over ResNet. In **safety-critical infrastructures such as substations, even moderate gains in fault location and classification accuracy can reduce the risk of misdiagnosis or missed faults, thereby improving maintenance decision quality and overall system reliability**. Therefore, within the considered deployment context, the additional computational cost is regarded as acceptable given the consistent accuracy advantages.

In summary, **while computational efficiency is an important consideration, the proposed model maintains sub-second inference latency while consistently delivering the highest diagnostic accuracy.** We acknowledge that the original manuscript did not sufficiently discuss this trade-off. In the revised version, **we have added a dedicated discussion paragraph clarifying the deployment assumptions and explicitly analyzing the balance between diagnostic accuracy and computational efficiency** in practical substation applications. This addition improves the clarity of the model's positioning and its applicability under different operational constraints.

The corresponding revisions in the Manuscript:

Results (Line 206–218)

Comparison of fault diagnosis models

……

The proposed model consistently outperforms all baseline approaches under varying levels of noise, sampling loss, and sensor failure (Tables 1–3, Fig. 1d–f, Supplementary Fig. 3–5, Supplementary Data). While it incurs moderate additional computational overhead (Supplementary Table 7), the overall training and inference costs remain within a practically deployable range. From an operational perspective, the observed

accuracy–efficiency trade-off should be interpreted in the context of practical substation workflows. Protection relays typically isolate faults within milliseconds, and subsequent diagnostic analysis is conducted after system stabilization using monitoring data collected from multiple sources. Under such post-event conditions, both the proposed model and the baseline methods operate within a sub-second inference range, which is sufficient for practical deployment. Given the safety-critical nature of substations, even moderate improvements in diagnostic accuracy can enhance operational reliability and reduce the risk of misdiagnosis or missed faults. Therefore, while computational efficiency remains an important consideration, the additional resource requirements of the proposed model are considered acceptable in applications where diagnostic robustness and precision are prioritized.
